# Genome identification of the LRR-RLK gene family in maize (*Zea mays*) and expression analysis in response to *Fusarium verticillioides* infection

**DOI:** 10.1186/s12870-025-06495-w

**Published:** 2025-04-25

**Authors:** Yiao Gao, Qing Qu, Ning Liu, Manli Sun, Xinfang Liu, Zhiyan Cao, Jingao Dong

**Affiliations:** 1 State Key Laboratory of North China Crop Improvement and Regulation, College of Plant Protection, Hebei Agricultural University, Baoding, 071001 China; 2 Hebei North University, Zhangjiakou, 075000 China; 3 Corn Research Institute, Liaoning Academy of Agricultural Sciences, Shenyang, 110161 China

**Keywords:** Maize *LRR-RLK*, *F. verticillioides*, Gene family, Bioinformatics expression analysis

## Abstract

**Background:**

Plant leucine-rich repeat receptor-like kinases (LRR-RLKs) are a ubiquitous class of proteins in plants. These receptors are primarily responsible for recognizing pathogen-associated molecular patterns (PAMPs) and are crucial for regulating plant growth, development, and immune responses. *Fusarium verticillioides*, a significant maize pathogen, causes diseases such as ear rot and stalk rot. However, the expression patterns of *LRR-RLK* in maize following *F. verticillioides* infection remain unclear.

**Results:**

A total of 205 maize *LRR-RLK* gene family members from 15 subfamilies were identified. The gene structures, physicochemical properties, and conserved motifs of these LRR-RLKs were thoroughly analyzed. Co-expression analysis of the *LRR-RLK* genes suggested that the gene family may have expanded through gene duplication, with relatively high co-expression observed in closely related species. To explore their expression patterns, we conducted comprehensive tissue expression profiling, revealing significant variation in expression levels across different tissues. Using transcriptome sequencing, we obtained the expression profiles of *LRR-RLK* genes at different time points after *F. verticillioides* infection in maize. The expression levels of these genes exhibited significant changes following inoculation. Notably, genes such as *Zm00001d027645*, *Zm00001d032116*, *Zm00001d032244*, *Zm00001d030323*, *Zm00001d031427*, *Zm00001d030981*, *Zm00001d031201*, *Zm00001d032344*, and *Zm00001d032745* showed marked alterations, indicating their potential involvement in resistance to *F. verticillioides* infection.

**Conclusions:**

In this study, we systematically identified members of the *LRR-RLK* gene family in maize and characterized the biological information of selected family members. Additionally, our data revealed that certain *LRR-RLK* family members in maize responded to *F. verticillioides* infection, with their expression levels being significantly up-regulated.

**Supplementary Information:**

The online version contains supplementary material available at 10.1186/s12870-025-06495-w.

## Introduction

Receptor-like kinases (RLKs) are classified based on their extracellular domains into several types, including leucine-rich repeat (LRR) RLKs, epidermal growth factor-like RLKs, S-domain RLKs, lectin-like RLKs, PR5K-type RLKs, and tumor necrosis factor-like RLKs [1]. Among these, LRR-RLKs represent the largest subfamily of RLKs [2]. In plants, LRR-RLKs are protein molecules comprising three main domains: a leucine-rich repeat (LRRs) motif-rich extracellular domain, a transmembrane domain (TMs), and a kinase domain (KDs) [3]. The extracellular domain specifically binds to extracellular signal molecules, activating the intracellular kinase domain. This activation leads to phosphorylation and autophosphorylation, facilitating the transmission of extracellular signals across the membrane into the cell, thereby triggering plant responses [4, 5]. The LRR domain typically consists of 20–30 amino acids, forming tandem repeat domains with at least eight distinct numbers and distributions of LRR motifs [6]. Structurally, each LRR domain adopts an α-helix and β-fold conformation, and multiple LRR domains arrange in parallel to form a horseshoe-shaped structure, which plays a critical role in mediating protein‒protein interactions [7].

Members of the *LRR-RLK* gene family play crucial roles in plant growth and development, immune signaling, and responses to biotic and abiotic stresses [8–10]. These proteins are involved in recognizing and responding to diverse environmental stimuli, such as pathogen recognition and nutrient sensing, and subsequently transmitting signals downstream through various pathways. For instance, in Arabidopsis (*Arabidopsis thaliana (L.) heynh*), the CLAVATA1 (CLV1) gene is associated with the development of the stem and floral apical meristem. CLV1 dimerizes upon binding to the receptor-like protein CLAVATA2 (CLV2) and recognizes the small peptide CLAVATA3 (CLV3) as a ligand. This interaction regulates the expression of downstream transcription factors, which are critical for promoting plant growth, differentiation, and flowering time regulation [11, 12]. Similarly, in rice (*Oryza sativa L*.), knockout mutants of the OsRK2 gene exhibit reduced tolerance to salt and drought stress, while overexpression lines in *Arabidopsis* show enhanced tolerance. OsRK2 activates downstream genes related to salt and drought stress by phosphorylating the zinc finger transcription factor ZOS3-18 (OsZF1), thereby improving plant tolerance to these stresses [13]. Research on *LRR-RLK* genes under biological stress is extensive. As a pattern recognition receptor, flagellin-sensitive 2 (FLS2) recognizes and binds flagellin 22 (flg22), transmitting and amplifying immune signals through MAPK phosphorylation. Downstream of FLS2, WRKY22 and WRKY29 proteins activate the expression of defense-related genes [14]. In Arabidopsis, BRI1-associated receptor kinase 1 (BAK1) interacts with BR insensitive 1 (BRI1) to form a complex, enhancing signal output via downstream phosphorylation and regulating the brassinosteroid (BRs) signaling pathway [15]. In tomato (*Solanum lycopersicum L*.), somatic embryogenesis receptor-like kinases 1 (SERK1) and 3 (SERK3) form an induction complex with FLS2, participating in the PTI response [16]. In rice, SPL36 encodes a receptor kinase that regulates defense genes WRKY53, BIMK2, and AOS2, conferring resistance to *Xanthomonas oryzae* infection [17]. Additionally, in potato (*Solanum tuberosum L*.), the elastin-laminin receptor (ELR) interacts with the suppressor of BIR1-1 (SOBIR1) to form a dimer. Upon induction by the *Phytophthora infestans* elicitor INF1, the ELR-SOBIR1 complex recruits SERK3, activating downstream resistance-related signaling pathways [18]. In summary, *LRR-RLK* genes play crucial roles in plant responses to environmental stimuli, growth regulation, and immune processes, making them a significant focus in plant biology and genome research.

Maize is a vital global grain, feed, and economic crop, and it is the largest grain crop in China [19]. Currently, maize cultivation ranks first among the four major food crops, playing a crucial role in human production and development. Its cultivation is strategically significant for ensuring stable national food production and sustainable agricultural supply. However, in recent years, the increasing severity of maize ear rot has posed a significant threat to maize yield and food security. The functional study of *LRR-RLK* genes is essential for disease resistance breeding, plant biotechnology, and disease control. By studying *LRR-RLK* genes in plants, we can understand how plants recognize pathogen molecules and regulate immune responses, thereby improving crop varieties and enhancing disease resistance. LRR-RLKs are key proteins that respond to various stresses, making the study of their gene families imperative. Previous studies have shown that the maize *LRR-RLK* family gene *ZmRLK7* is involved in plant growth and development [20]. However, systematic identification and analysis of *LRR-RLK* family genes remain limited, particularly the expression patterns of *LRR-RLK* in maize following *F. verticillioides* infection.

Bioinformatics methods were employed to identify and classify the *LRR-RLK* family genes in maize, followed by an analysis of their gene structure, chromosomal localization, collinearity, and tissue expression patterns. Additionally, transcriptome data of *LRR-RLK* family genes were analyzed at different time points after inoculation with *F. verticillioides*. qRT‒PCR was utilized to determine the expression patterns of selected *LRR-RLK* family genes in response to *F. verticillioides* infection, identifying genes associated with maize antifungal resistance. These findings provide a foundation for further exploration of the biological functions of *LRR-RLK* family genes and serve as a reference for in-depth analysis of the molecular mechanisms by which *LRR-RLK* family genes regulate plant disease resistance.

## Results

### Identification and bioinformatics analysis of the *LRR-RLK* gene in maize

Through sequence alignment, 205 members of the *LRR-RLK* family were identified in maize (Table S1). Analysis of the encoded proteins revealed that the number of amino acids ranged from 592 (*Zm00001d051094*) to 1569 (*Zm00001d007776*), molecular weights ranged from 64,898.81 (*Zm00001d051094*) to 178,734.75 (*Zm00001d007776*), and isoelectric points ranged from 5.05 (*Zm00001d0336775*) to 9.5 (*Zm00001d023548*) (Table S2). Approximately 72.22% of the LRR-RLK proteins had isoelectric points below 7, indicating that most are acidic proteins. Subcellular localization is critical for understanding gene function. Cell PLoc analysis showed that all genes are located on the cell membrane, suggesting that LRR-RLK genes may play a significant role in signal transduction.

### Phylogenetic tree analysis of the *LRR-RLK* gene

**Fig. 1:**
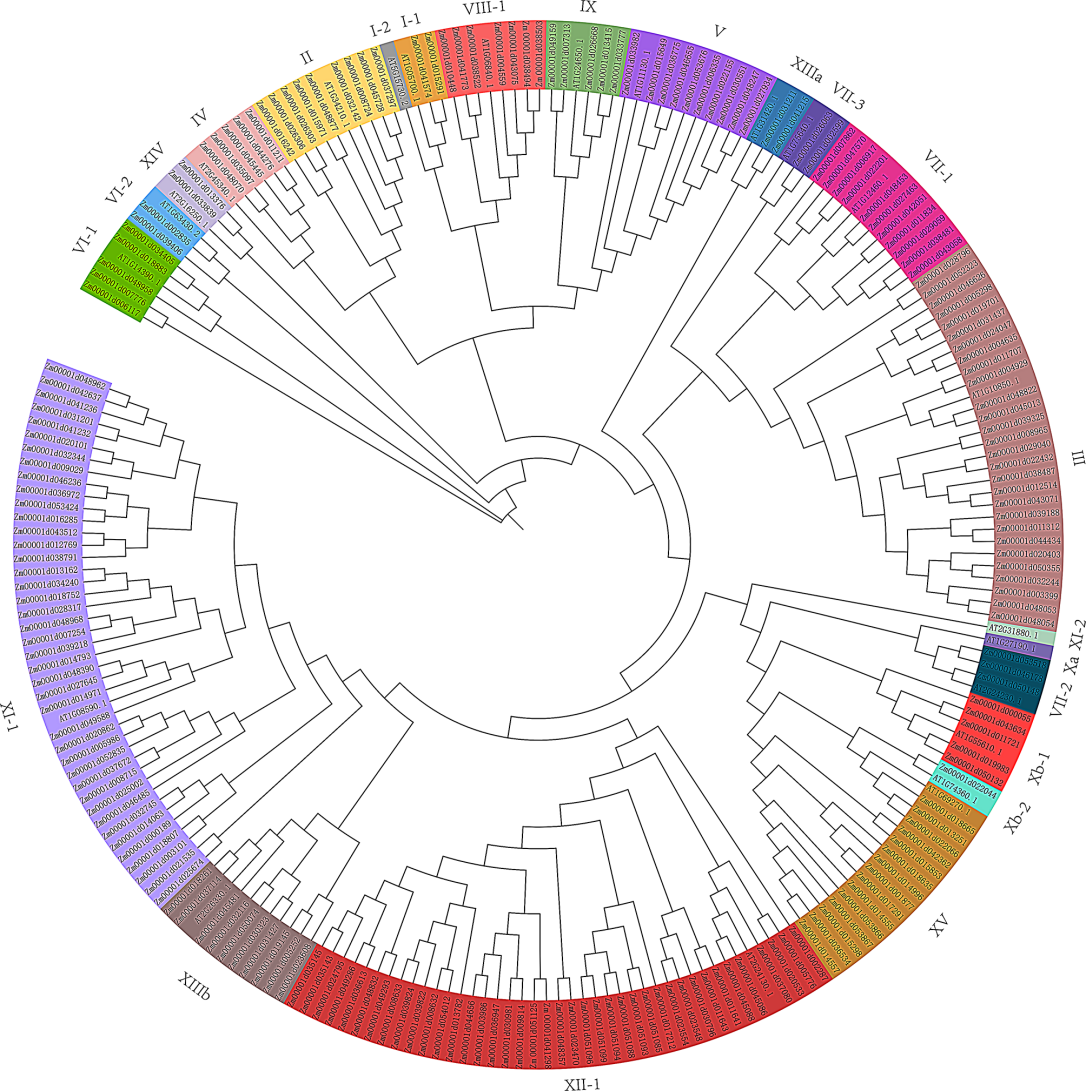
Phylogenetic tree of the maize *LRR-RLK*. Note. The phylogenetic tree was constructed using MEGA11 software on the basis of the LRR-RLK protein sequences, with the distance model set to the maximum likelihood method. Subfamilies are marked with different colours

Phylogenetic analysis provides insights into gene evolutionary relationships. Using MEGA11 software, a phylogenetic tree was constructed from 205 LRR-RLK protein sequences in maize and representative LRR-RLK sequences from Arabidopsis subfamilies (Fig. 1). The analysis classified the *LRR-RLK* gene family into 15 distinct subfamilies (Clades I-XV), further divided into 20 subgroups. Subfamily XI-1, with 41 members, is the largest in maize, followed by Subfamily XII-1 (40 members) and Subfamily III (28 members). These three subfamilies likely play significant roles in the maize life cycle. In contrast, the Xb-2 subfamily has only one member, making it the fewest.

### Conserved motifs, domain, gene structure, and transmembrane domain of LRR-RLK

**Fig. 2:**
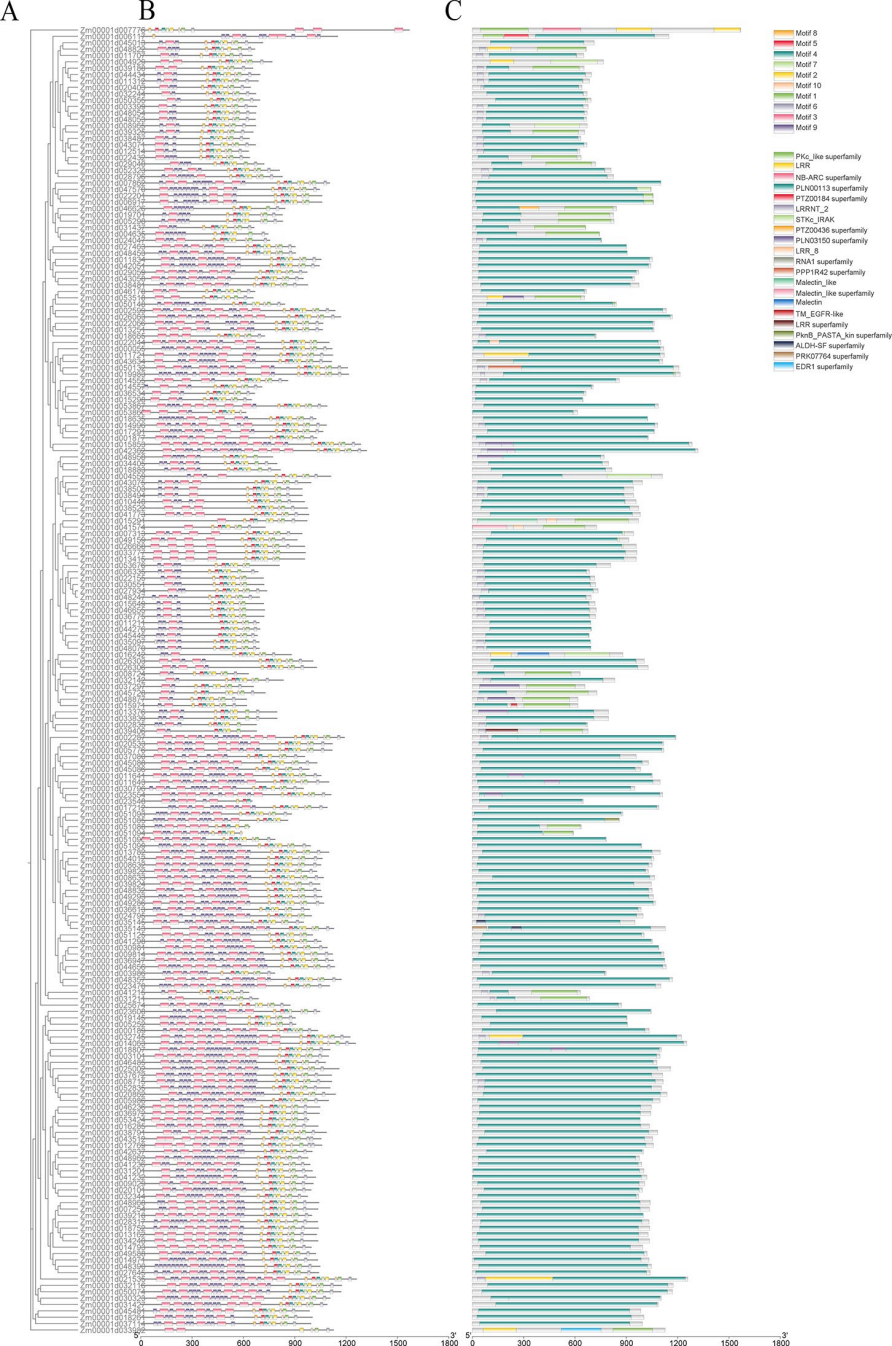
Detailed structure of LRR-RLK family proteins. (**A)** Phylogenetic tree, (**B)** conserved motif, (**C)** conserved domain

Motifs within the same subfamily are highly similar, consistent with the phylogenetic analysis results (Fig. 2A). A motif, often referred to as a super secondary structure, is composed of protein secondary structural units and plays a critical role in protein function and structure. Certain motifs are universally present among LRR-RLK members (Fig. 2B). For instance, each member of the LRR-RLK family possesses motif 3. However, the types and distribution of motifs vary to different extents among members of different subfamilies. This variation may lead to non-functionalization, sub-functionalization, or neofunctionalization of these *LRR-RLK* genes. Conserved domain analysis revealed that over 96% of LRR-RLK members contain the PLN00113 superfamily (Fig. 2C). However, PTZ00184 superfamily is present in only one family member. These findings highlight the evolutionary unity within subfamilies and the divergence between different subfamilies.

The gene structures of all family members were analyzed (Figure S1). As shown in Figure S1, the number of exons and introns varied significantly among subfamilies. Subfamilies I, V, VI, and VIII contain more exons and introns, while subfamilies IX, X, and XI have fewer. Notably, approximately 83% of genes in subfamily X possess only one exon and lack introns, suggesting functional diversity among subfamilies. In contrast, genes within the same subfamily exhibit similar exon-intron structures. For instance, all members of subfamily IX have two exons and one intron, while subfamily IV members uniformly have four exons and three introns.

To better characterize LRR-RLK protein structures, transmembrane domains of all members were analyzed using TMHMM online software (Figure S2). The results revealed that approximately 96% of family members contain transmembrane domains, with most members possessing 1–2 domains. Only three proteins—Zm00001d052323, Zm00001d038481, and Zm00001d018261—contain three transmembrane domains, while Zm00001d045086 uniquely contains four.

### Chromosome mapping of the *LRR-RLK* gene family

**Fig. 3:**
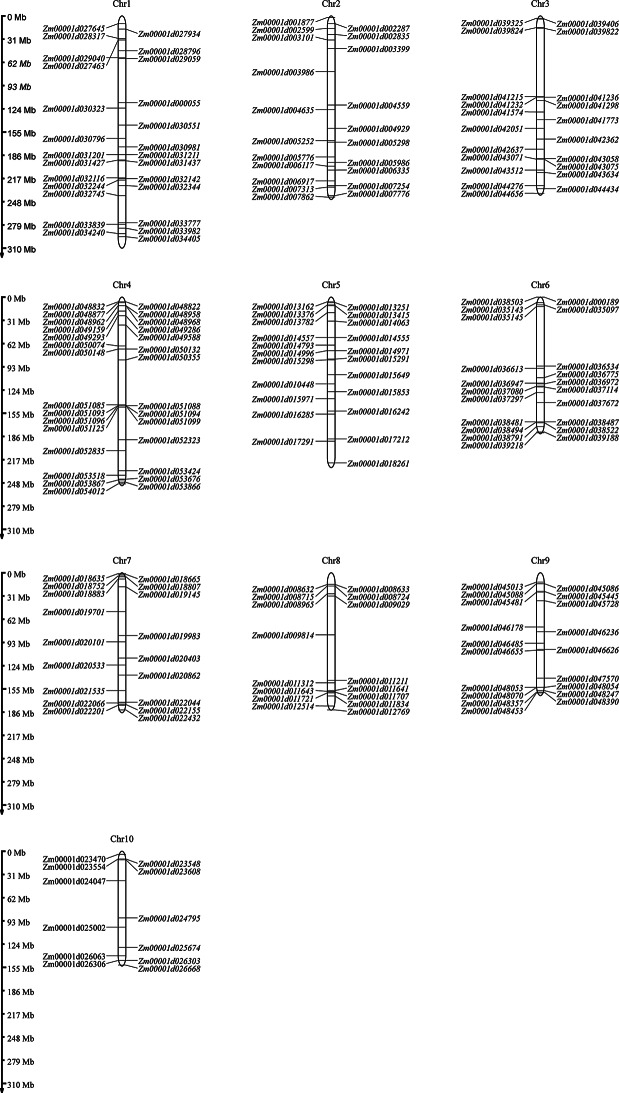
Distribution of *LRR-RLK* genes on chromosomes in maize. Chromosome size is indicated by relative length. The scale bar represents megabases (Mb). The physical locations of *LRR-RLK* are drawn on each chromosome

The 205 members of the maize LRR-RLK gene family are distributed across all 10 chromosomes, though unevenly (Fig. 3). Chromosome 4 (Chr4) has the highest number of genes (29), followed by Chr1 with 26 genes. Chr5 contains 22 genes, while Chr2, Chr3 and Chr6 each have 21 genes. Chr9, Chr7 and Chr8 contain 19, 18, and 16 genes, respectively. Chr10 has the fewest genes, with only 12.

### Collinearity analysis and fragment duplication of the *LRR-RLK* gene

**Fig. 4:**
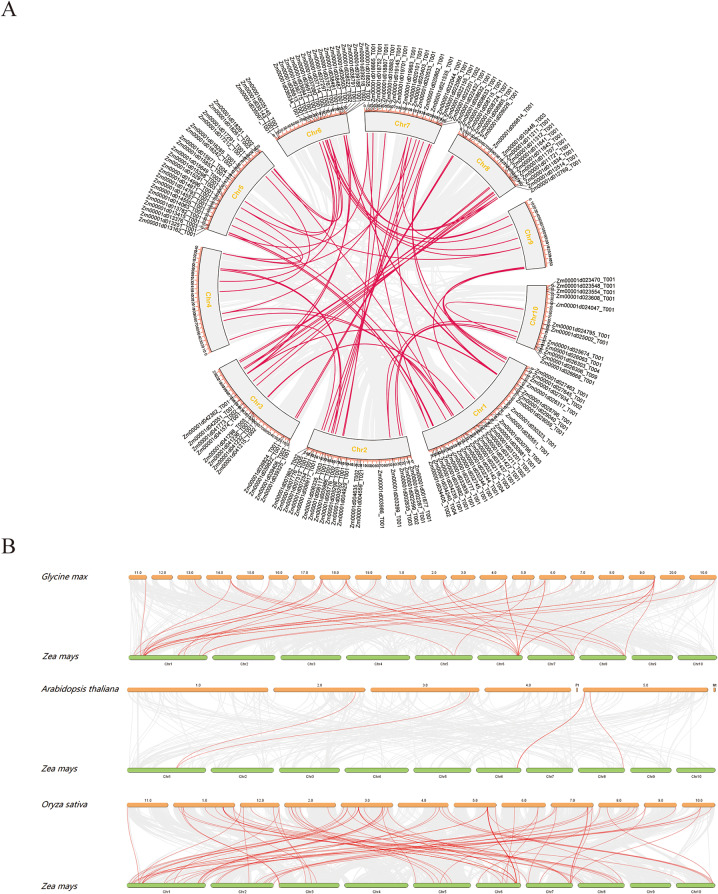
Collinearity analysis of *LRR-RLK* gene family. (**A**) Intraspecific collinearity. The blue font represents tandem repeats. Red lines represent segmental duplication. (**B**) Interspecific collinearity. The figure illustrates the collinearity of *Zea mays* with *Glycine max*, *Arabidopsis thaliana*, and *Oryza sative*. Short rods of varying lengths depict chromosomes, while red lines indicate homologous pairs among the species

Using TBtools, we performed intraspecies collinearity analysis on maize *LRR-RLK* family genes. The results identified 29 fragment repeats and 1 pair of tandem repeats in maize (Fig. 4A). The tandem repeat is located at *Zm00001d031427-T001* on chromosome 1. Fragment duplication events were observed on all chromosomes, with over 25% of genes involved in duplication. Chromosome 1 had the highest number of duplicated genes, totaling 16 pairs. These findings suggest that the expansion of the *LRR-RLK* gene family likely resulted from frequent fragment duplications, underscoring the importance of these genes in maize growth and development. Additionally, interspecies collinearity analysis between maize and soybean (*Glycine max (L.) Merr.*), Arabidopsis and rice (*Oryza sativaL*.) revealed 33 pairs of homologous genes between maize and soybean (Fig. 4B), distributed on maize chromosomes 1, 5, 6, 7, and 8, with chromosome 1 having the most. Maize and rice share 70 pairs of homologous genes, present on all chromosomes except 4 and 9. In contrast, maize and Arabidopsis share only four pairs of homologous genes, reflecting maize’s closer evolutionary relationship with monocotyledonous plants like rice and soybean than with dicotyledonous plants like Arabidopsis. These results highlight potential evolutionary divergences among species.

### Prediction of the maize LRR-RLK protein interaction network

**Fig. 5:**
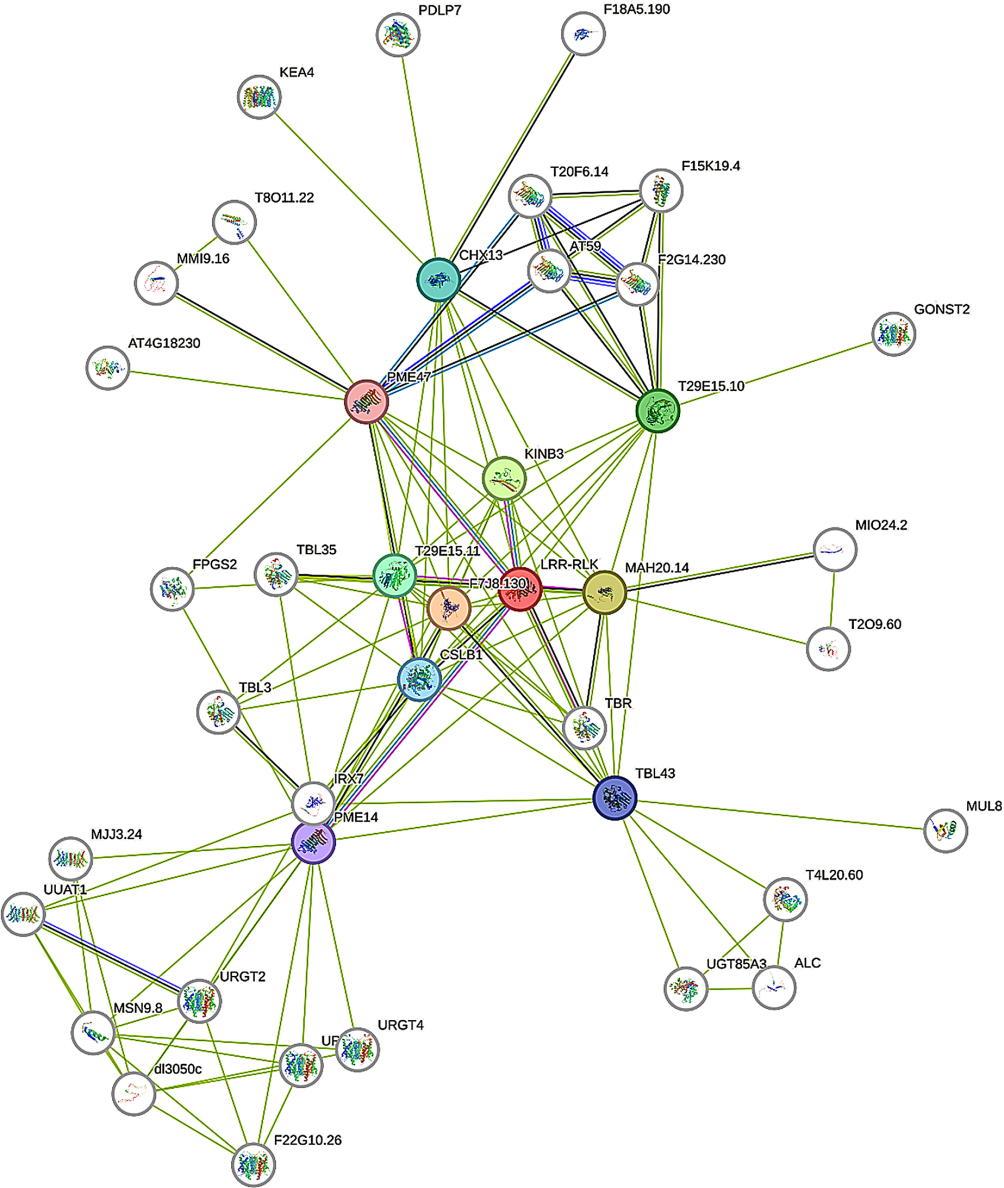
Protein–protein interaction network of the LRR-RLK proteins. The colored nodes represent proteins that interact directly with the LRR-RLK, and the white nodes represent proteins that interact indirectly. The three-dimensional structural diagram of the protein is shown inside the nodes, and the different colored lines between the nodes indicate the type of interaction evidence

Using the STRING 12.0 online database, the interaction network of LRR-RLK proteins was analyzed based on the Arabidopsis homologous protein model (Fig. 5). The results identified numerous proteins interacting with LRR-RLK proteins. For instance, the calcium-binding EF hand-shaped domain family protein (MAH20.14) regulates biological functions by binding calcium ions [21]. The glycoside hydrolase 28 family protein (T29E15.10) belongs to the polysaccharide galacturonate family [22]. Cellulose synthase-like protein (CSLB1), localized in the Golgi apparatus, polymerizes the backbone of noncellulose polysaccharides (e.g., hemicellulose) in plant cell walls [23]. Pectin esterases (PME14 and PME47) modify the cell wall via pectin demethylation [24]. Additionally, the cation/H⁺ cotransporter 13 (CHX13) acts as a high-affinity potassium transporter, facilitating K⁺ acquisition and functioning as a K⁺/H⁺ cotransporter [25]. These findings highlight the diverse roles of LRR-RLK proteins, including their involvement in cell wall synthesis and the calcium ion signaling pathway.

### Analysis of tissue expression patterns of *LRR-RLK*

**Fig. 6:**
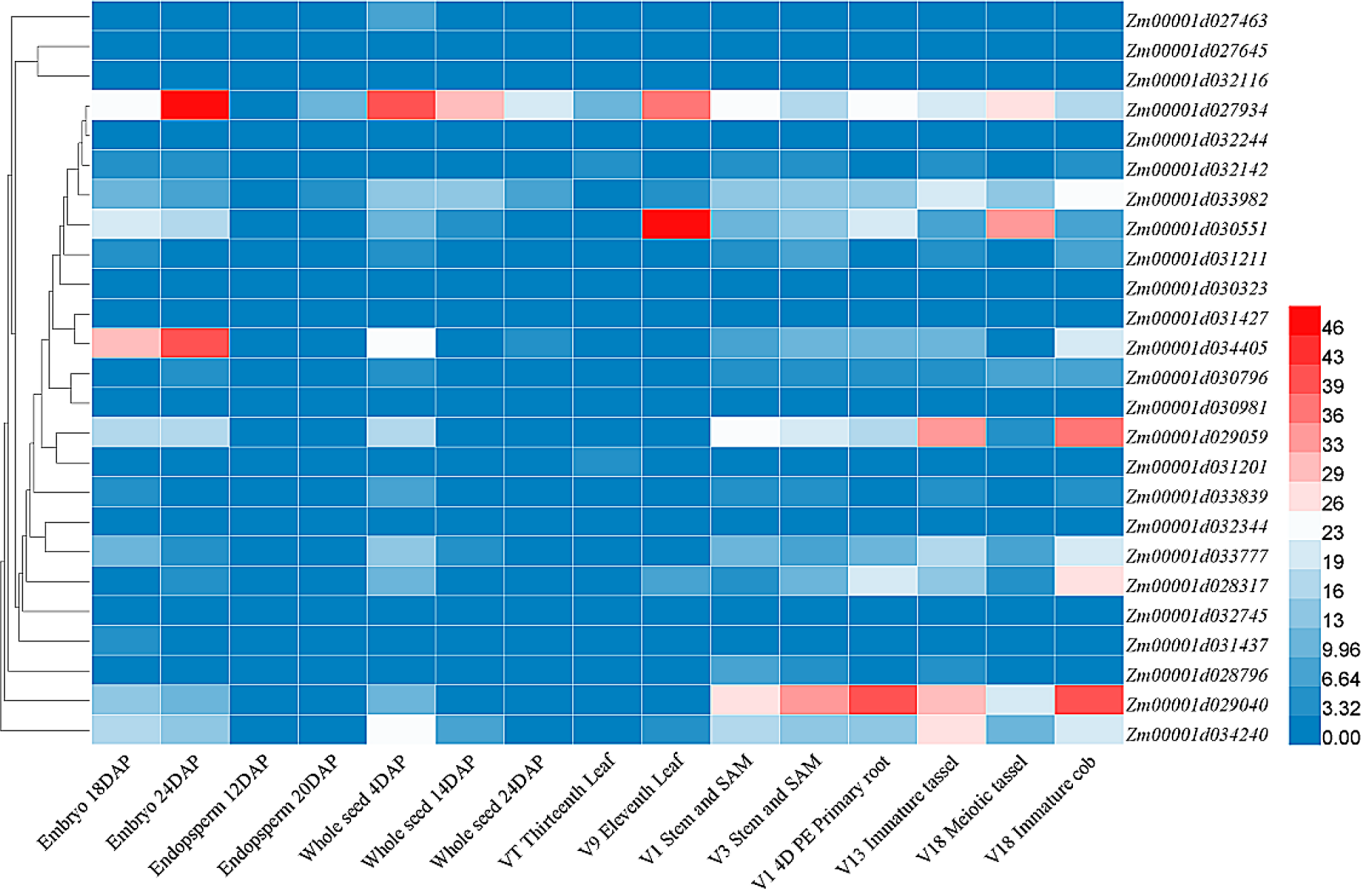
Expression patterns of 25 *LRR-RLK* family members in different developmental stages and tissues. The different colours indicate the expression levels of the *LRR-RLK* genes in maize. The expression abundance of each transcript is represented by the normalized fragments per kilobase pair per million (FPKM) value and displayed as colored boxes from blue (lower expression) to red (higher expression)

Gene expression varies significantly across tissues, and understanding these patterns is crucial for exploring gene function. Using the qTeller tool in the MaizeGDB database, tissue expression data for maize *LRR-RLK* genes were obtained. A total of 25 *LRR-RLK* genes had detectable expression data (Fig. 6). In the heatmap, dark blue regions indicate low expression, while deep red regions represent high expression. The gene *Zm00001d027934* showed high expression in all tissues, suggesting it may function throughout maize growth and development. *Zm00001d030551* exhibited high expression in leaves and meiotic tassel, indicating potential roles in these tissues. Interestingly, genes *Zm00001d033982*, *Zm00001d034405*, *Zm00001d034405*, *Zm00001d029059*, *Zm00001d033777*, *Zm00001d028317*, *Zm00001d029040*, and *Zm00001d034240* were expressed to a different extent in embryo, whole seeds, stem and SAM, primary root, immature tassel and meiotic tassel, suggesting that these eight genes may be functionally similar. There were also some genes, such as *Zm00001d027645*, *Zm00001d032116*, *Zm00001d032244*, *Zm00001d030323*, *Zm00001d031427*, *Zm00001d030981*, *Zm00001d031201*, *Zm00001d032344* and *Zm00001d032745* were expressed at relatively low levels in all tissues.

### Expression profile of the *LRR-RLK* gene under *F. verticillioides* stress

**Fig. 7:**
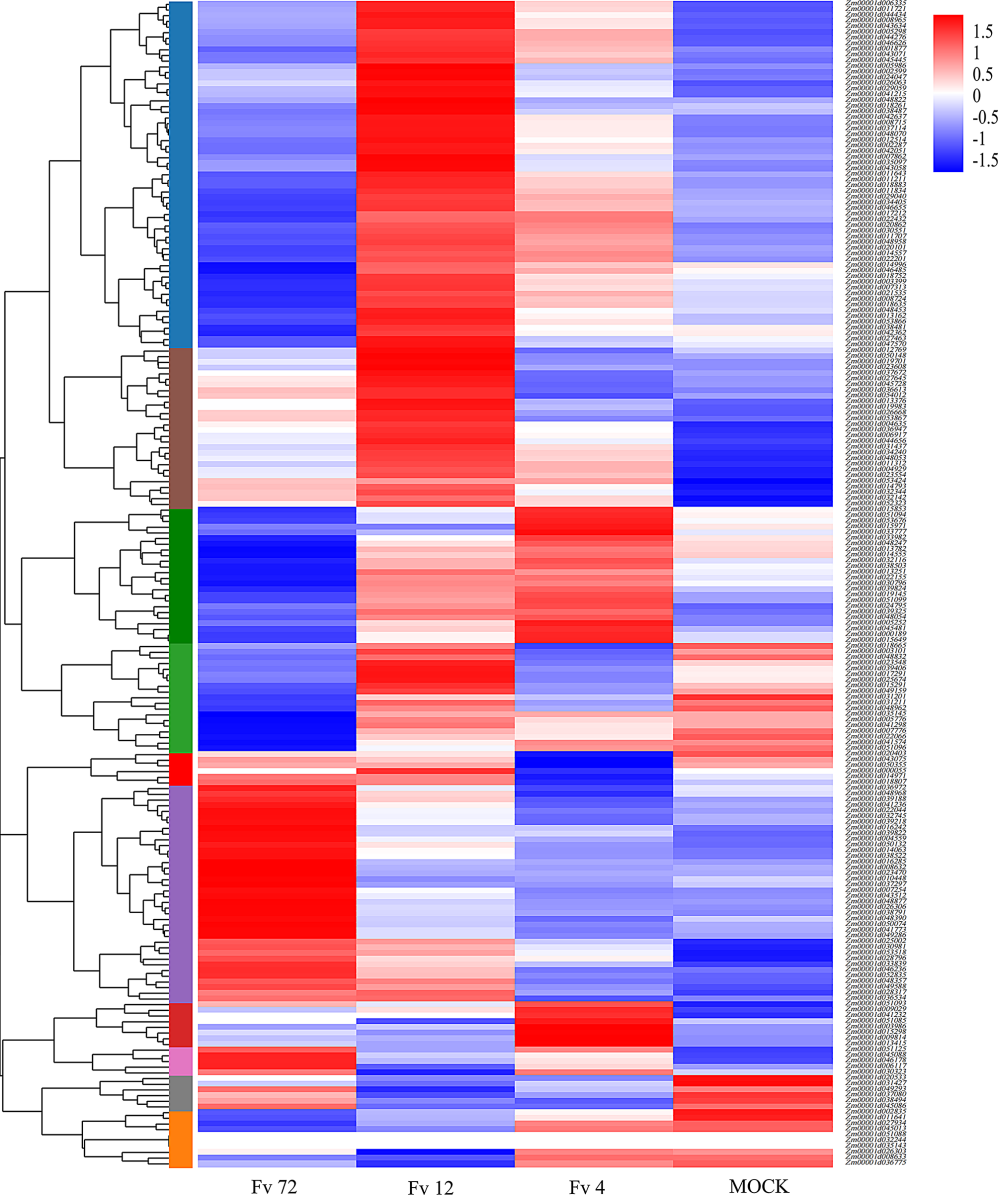
Heatmap of the expression profiles of the *LRR-RLK* gene family in maize at different times after inoculation with *F. verticillioides*. Each column in the figure represents a sample, and each row represents a gene. Each column corresponds to a sample, and each row represents a gene. Colors indicate standardized gene expression values (log10-transformed FPKM), with red and blue denoting high and low transcript abundance, respectively

In this study, transcriptome sequencing was performed on maize seed samples inoculated with *F. verticillioides* at 0 h (mock), 4 h (Fv4), 12 h (Fv12), and 72 h (Fv72). The expression levels of the *LRR-RLK* family genes were calculated using FPKM values at each time point (Fig. 7, Table S4). The gene family members were categorized into three expression patterns. The first pattern showed a gradual decrease or no significant change in expression after inoculation. For example, the expression levels of *Zm00001d027934* under mock, Fv4, Fv12, and Fv72 conditions were 27.566667, 27.966667, 23.96, and 22.41, respectively. There was no significant change at 4 h post-inoculation, but a gradual decline was observed after 12 h. The second pattern exhibited an overall upward trend in expression over time. For instance, the expression levels of *Zm00001d033839* at the four time points were 1.416667, 1.793333, 2.153333, and 2.766667, respectively, demonstrating a gradual increase. The third pattern is characterized by a downregulation or unchanged gene expression shortly after inoculation, followed by a significant upregulation at 72 h post-inoculation. For instance, the expression level of *Zm00001d038494* was measured at 0.74 without inoculation, decreased to 0.56 at 4 h post-inoculation, rose to 0.626667 at 12 h, and further increased to 0.863333 by 72 h. Notably, genes such as *Zm00001d027645*, *Zm00001d032116*, *Zm00001d032244*, *Zm00001d030323*, *Zm00001d031427*, *Zm00001d030981*, *Zm00001d031201*, and *Zm00001d032344*, which had low expression in various tissues and before inoculation, showed significant upregulation at 12–72 h post-inoculation. This suggests their potential role in resisting *F. verticillioides* infection.

### qRT‒PCR validation of *LRR-RLK* gene expression under *F. verticillioides* stress

**Fig. 8:**
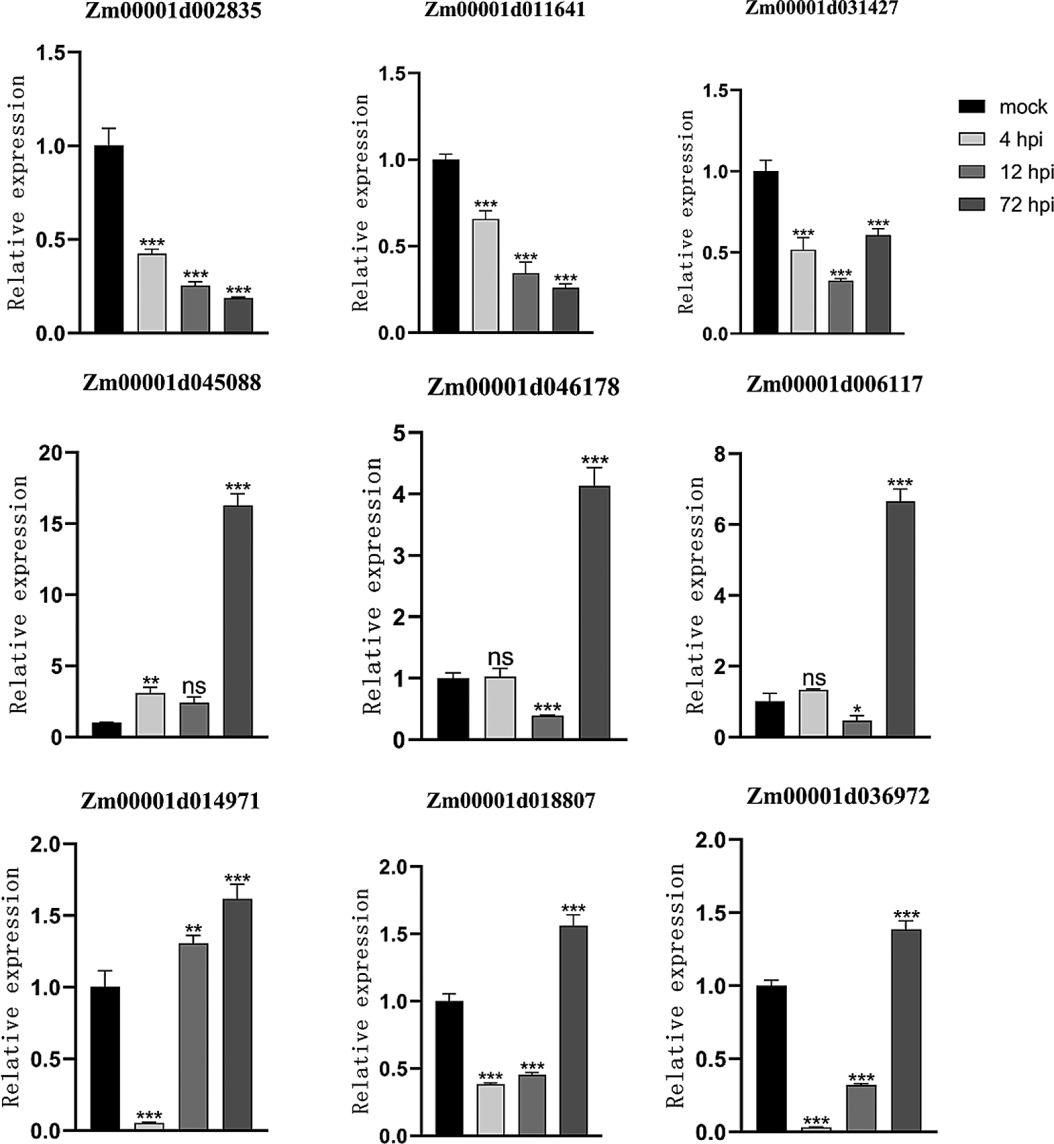
Gene expression of parts of the LRR-RLK gene family in maize at different times after inoculation with *F. verticillioides*. Nine *LRR-RLK* members were detected by RT‒qPCR at no inoculation and after inoculation for 4, 12 and 72 h. The data are presented as the means (± SE) of three biological replicates (ns > 0.05, * *p* ≤ 0.05, ** *p* ≤ 0.005, *** *p* ≤ 0.001)

Based on transcriptome sequencing results, *LRR-RLK* family members with significant expression differences were selected for qRT‒PCR analysis at 4, 12and 72 h post-inoculation. The expression trends aligned with the transcriptome data (Fig. 8). Specifically, the expression levels of *Zm00001d002835* and *Zm00001d011641* gradually decreased over time. *Zm00001d031427*, *Zm00001d045088*, *Zm00001d046178* and *Zm00001d006117* showed elevated expression after 4 h of inoculation, which decreased slightly after 12 h of inoculation but increased significantly again after 72 h of inoculation. Additionally, *Zm00001d014971*, *Zm00001d018807*, and *Zm00001d036972* exhibited a significant decrease at 4 h but gradually increased thereafter.

## Discussion

Plants face various biotic and abiotic stresses during growth, significantly impacting their development [26]. To combat these stresses, plants have evolved defense mechanisms, including receptor-like kinases (RLKs) that sense stimuli and transmit signals to initiate stress responses. Among RLKs, leucine-rich repeat receptor-like kinases (LRR-RLKs) form the largest subclass and play critical roles in plant growth, development, and stress responses. Extensive research has focused on whole-genome identification and analysis of *LRR-RLK* gene families. To date, *LRR-RLK* genes have been systematically identified and analyzed in over 31 plant species. For example, 225 *LRR-RLK* genes were identified in Arabidopsis and classified into 15 subfamilies [2], while rice contains 309 *LRR-RLK* genes grouped into 5 subfamilies [27]. Similarly, 303 *LRR-RLK* genes were identified in Chinese cabbage *(Brassica rapa*) and classified into 15 subfamilies [28], and 234 *LRR-RLK* genes were found in tomato, divided into 10 subfamilies [29]. In apple (*Malus pumila Mill*.), 378 *LRR-RLK* genes were identified and classified into 15 subfamilies [30]. This study identified 205 maize *LRR-RLK* genes, classified into 15 subfamilies based on the Arabidopsis classification system. These genes exhibit diverse characteristics and are widely distributed across maize chromosomes. Gene structure analysis revealed significant variations in exon and intron numbers, suggesting functional divergence among subfamilies despite their shared classification [26]. Subfamily III contains the most genes, followed by subfamilies VI and II. Members within the same subfamily share similar domain compositions, indicating potential functional similarities or synergistic roles in pathogen resistance. Species collinearity analysis highlighted a close evolutionary relationship between maize *LRR-RLK* genes and those in rice and soybean, likely due to their shared monocotyledonous ancestry. Intraspecific collinearity analysis identified a tandemly duplicated gene (*Zm00001d018261-T001*) and multiple genes generated by chromosomal segment duplications. For instance, *Zm00001d041298-T003* on Chr3 and *Zm00001d031427-T001* on Chr1 exhibit synteny, supporting the hypothesis that gene duplication drives the expansion of the *LRR-RLK* family.

Protein interaction analysis revealed that LRR-RLK proteins not only promote cell wall synthesis but also interact with calcium-binding EF-hand domain proteins, implicating them in calcium signaling [31]. Calcium ions (Ca²⁺) are closely linked to plant immunity. For instance, the NBS-LRR gene CcRNL1 in Cinnamomum camphora (*Cinnamomum camphora L. Resl.*) is induced by powdery mildew, suggesting a role in resistance to this pathogen [32]. Similarly, in tobacco (*Nicotiana benthamiana*), most LRR-RLK genes are differentially expressed after *Verticillium dahliae* infection, activating the MAPK pathway [33]. Additionally, NbBRI1 regulates H₂O₂ and NO production, participating in BRs-mediated defense signaling [34]. In this study, maize *LRR-RLK* family genes responded to *F. verticillioides* infection, exhibiting three distinct expression patterns at 4, 12and 72 h post-inoculation. The first pattern showed a consistent increase in expression over time, suggesting these genes actively participate in resistance to *F. verticillioides*. LRR-RLKs are known to play roles in hormone signaling pathways, such as BRs [9] and abscisic acid (ABA) [35], and may similarly function in this context. The second pattern featured unchanged or decreased expression at 4 h but a gradual increase at 12 and 72 h. These genes may respond to *F. verticillioides* infection during the early stages, with protein activation occurring in the middle and later phases. Through signal transduction, defense-related genes, such as PR protein genes and phytohormone synthesis genes, are upregulated. This activates downstream signaling pathways, triggers the immune system, and contributes to the plant’s disease resistance response. In contrast, the third type of gene exhibits a gradual decrease or no significant change in expression over time after inoculation. These genes may not be involved in resistance to *F. verticillioides* but could play roles in other maize growth and developmental processes.

## Conclusions

A total of 205 maize *LRR-RLK* gene family members were identified, distributed across all 10 chromosomes. Each member contains LRR domains, transmembrane domains, and kinase domains, with all proteins localized to the cytoplasmic membrane. Phylogenetic analysis classified these genes into 15 groups and 20 subgroups. Gene duplication events were prevalent within the *LRR-RLK* family. Expression levels varied across tissues, and some genes showed upregulation in response to *F. verticillioides* infection.

## Materials and methods

### Identification and basic properties of the *LRR-RLK* family members in maize

LRR-RLK proteins contain kinase (KD), leucine-rich repeat (LRR), and transmembrane (TM) domains. Hidden Markov models (HMMs) for these domains, including Pkinase (PF00069), Pkinase-Tyr (PF07714), LRR-1 (PF00560), LRR-2 (PF07723), LRR-3 (PF07725), LRR-4 (PF12799), LRR-5 (PF13306), LRR-6 (PF13516), LRR-8 (PF13855), LRR-9 (PF14580), and LRV (PF01816), were downloaded from the Pfam database (http://pfam.xfam.org/). A homology search was conducted on the maize Zm-B73-REFERENCE-GRAMENE-4.0 protein database using MaizeGDB (https://maizegdb.org/) with an e-value threshold of < 1 × 10⁻¹⁰, and results were collected for further screening. The TAIR database v10.0 (http://www.arabidopsis.org/) provided Arabidopsis LRR-RLK amino acid sequences, which were used for a BLAST + v.2.6.0 similarity search against the maize protein database (e-value < 1 × 10⁻⁵, homology > 50%) [36]. The online software ExPASy ProtParam (http://web.expasy.org/protparam/) analyzed protein properties, including amino acid sequence length, molecular weight, and isoelectric point. Subcellular localization predictions were performed using the online software Cell-PLoc 2.0 (http://www.csbio.sjtu.edu.cn/bioinf/Cell-PLoc-2/).

### Evolutionary analysis of the LRR-RLK family members in maize

Amino acid sequences of representative Arabidopsis LRR-RLK subfamily members were retrieved from NCBI, and sequences of all maize LRR-RLK members were downloaded from MaizeGDB. Using the online software MEGA11 (http://www.megasoftware.net), a phylogenetic tree was constructed with the maximum likelihood method. The tree was visualized using the online software iTOL (https://itol.embl.de/).

### LRR-RLK gene structure, motif and transmembrane structure domain analysis in maize

The LRR-RLK protein sequences were submitted to the One Step Build a ML Tree function of TBtools to determine phylogenetic relationships. Using the online software MEME wrapper function to predict the conserved motifs of the LRR-RLK proteins with the maximum number of motifs 10, and e-value 10. The conserved domain information of the LRR-RLK proteins were analyzed using the online tool NCBI Batch CD-search (www.ncbi.nlm.nih.gov). Then, phylogenetic relationship information, conserved motif information, and domain information were submitted to the Gene Structure View function for visualization. The gene structure diagrams were plotted using the online software GSDS 2.0 (http://gsds.gao-lab.org/).Transmembrane domains of maize LRR-RLK proteins were predicted using the online software TMHMM server 2.0 (https://services.healthtech.dtu.dk/services/TMHMM-2.0/).

### Chromosomal localization of *LRR-RLKs* in maize

Maize genome annotation files were downloaded from MaizeGDB (https://maizegdb.org/). The chromosomal locations of all LRR-RLK family members were visualized and analyzed using the online software MG2C (http://mg2c.iask.in/mg2c_v2.1/).

### Prediction of maize LRR-RLK family protein interaction network

The LRR-RLK protein interaction network was predicted using the online software STRING 12.0 (https://cn.string-db.org/). Arabidopsis thaliana was selected as the model species, with a confidence threshold of 0.4 and a maximum of 10 interactions.

### Covariance analysis of maize *LRR-RLK*

Intraspecific and interspecific covariance of *LRR-RLK* genes was analyzed using the one-step MCScanX function in TBtools. Genome annotation files for Arabidopsis, rice, and soybean were obtained from Ensembl Plants (http://plants.ensembl.org/index.html). Intraspecies covariance parameters were set to default values, while interspecies covariance used an E-value threshold of 1 × 10⁻⁵, with other parameters at default settings.

### Analysis of tissue expression patterns of maize *LRR-RLK*

Expression data for *LRR-RLK* family members across different tissues were downloaded from qTeller MaizeGDB (https://qteller.maizegdb.org/). Cluster analysis was conducted using TBtools to generate expression profiles.

### Analysis of the expression pattern of genes related to *F. verticillioides* stress

#### Plant material

The maize B73 inbred line, known for its high yield, quality, and stress resistance, has been widely cultivated in China since the 1960s. Seeds used in this study were harvested from the experimental field at the Liaoning Academy of Agricultural Sciences (Shenyang, China). Plants were grown under 16 h of light and 8 h of darkness at 25 °C.

#### Inoculation of maize seeds with *F. verticillioides*

The activated *F. verticillioides Fv7600* on PDA medium was punched out of the discs with a 6 mm diameter punch, inoculated into sodium carboxymethylcellulose (CMC) liquid medium, and incubated at 25 °C for 3–5 days with darkness and shaking, then filtered through three layers of sterile gauze, and diluted into a spore suspension of 1 × 10^6^ spores/mL in sterile water for spare use. For inoculation, uniform and intact maize B73 seeds were surface-sterilized on a clean bench. Seeds were treated with 75% ethanol for 1 min, rinsed three times with sterile water, followed by 1% sodium hypochlorite for 3 min and another three rinses with sterile water. Seeds were then soaked in sterile water for 12 h. Scratched seeds were placed in a glass dish lined with filter paper, and 25 µL of the spore suspension was applied to the wound, ensuring contact for at least 10 s. Seeds were incubated in the dark at 25 °C, maintaining moisture throughout. Samples were collected at 4 h (Fv4), 12 h (Fv12), and 72 h (Fv72) post-inoculation [37]. Each experiment included ten maize seeds with three biological replicates. Samples were immediately frozen in liquid nitrogen and stored at -80 °C for RNA extraction.

#### RNA extraction

Maize seed samples were ground into powder using liquid nitrogen, and RNA was extracted following the protocol of the Tiangen Polysaccharide and Polyphenol Plant Total RNA Extraction Kit. Briefly, 0.1 g of maize seed powder was mixed with 1 mL of SL lysis buffer (provided in the kit) and vortexed vigorously. After centrifugation, the supernatant was transferred to the CS filtration column and centrifuged again. The supernatant from the collection tube was transferred to a new nuclease-free centrifuge tube. An appropriate volume of anhydrous ethanol was added to the supernatant, which was then transferred to the CR3 adsorption column. After centrifugation to remove waste liquid, DNase I solution was added to the center of the CR3 column. Deproteinization and wash buffers were sequentially added to the CR3 column, followed by centrifugation. Finally, RNase-Free ddH₂O was added dropwise to the CR3 column, and RNA was eluted by centrifugation. RNA integrity was assessed by agarose gel electrophoresis, while RNA concentration and quality were measured using a Thermo NanoDrop 2000 spectrophotometer.

#### RNA-seq and expression pattern analysis

Hierarchical cluster analysis of maize LRR-RLK family members was performed using the Majorbio analysis platform. RSEM software was employed to calculate group averages for analysis. All FPKM values in the graph were transformed by Log10, and the maize RefGen_v4 genome was used as the reference. Differential expression analysis was conducted using DESeq2, with significance defined as P-adjustment < 0.05 and the Benjamini-Hochberg (BH) method for multiple testing correction.

#### qRT‒PCR

Nine LRR-RLK genes exhibiting distinct expression patterns were selected for qRT-PCR validation. Reverse transcription was performed using the PrimeScript™ RT Reagent Kit with gDNA Eraser (Takara). The maize *UBQ9* gene served as the internal reference [38], and primers were designed using Primer 5.0 software (Table S3). The qRT-PCR reaction mixture consisted of 10 µL SYBR Green (US EVERBRIGHT), 0.4 µL of each primer, 8.2 µL ddH₂O, and 1 µL cDNA, totaling 20 µL. Reaction conditions included an initial denaturation at 95 °C for 2 min, followed by 45 cycles of 95 °C for 5 s and 60 °C for 30 s. Each treatment was performed in triplicate. Data were analyzed using the 2⁻ΔΔCT method, with statistical significance determined by SPSS 18.0 and visualized using GraphPad Prism 8.

## Electronic supplementary material

Below is the link to the electronic supplementary material.

Supplementary Material 1

Supplementary Material 2

Supplementary Material 3

Supplementary Material 4

Supplementary Material 5

Supplementary Material 6

Supplementary Material 7

## Data Availability

The transcriptome data are available in the NCBI SRA repository, SRA accession number is PRJNA735336. HMMs were downloaded from the Pfam database (http://pfam.xfam.org/). The TAIR database v10.0 (http://www.arabidopsis.org/) provided Arabidopsis LRR-RLK amino acid sequences. Amino acid sequences of all maize LRR-RLK members and Zm-B73-REFERENCE-GRAMENE-4.0 protein database were downloaded from MaizeGDB (https://maizegdb.org/). Genome annotation files for Arabidopsis, rice, and soybean were obtained from Ensembl Plants (http://plants.ensembl.org/index.html). Expression data for LRR-RLK family members across different tissues were downloaded from qTeller MaizeGDB (https://qteller.maizegdb.org/). Other datas used and analyzed during the current study are contained within the article or supplementary files.
