# Supplementary material for: Genome identification of the LRR-RLK gene family in maize (Zea mays) and expression analysis in response to Fusarium verticillioides infection
Source: BMC Plant Biol. 2025 Apr 25;25:524. doi: 10.1186/s12870-025-06495-w (PMC12023693; doi:10.1186/s12870-025-06495-w)
Supplement: Supplementary file 1 — Supplementary Material 1 [file 12870_2025_6495_MOESM1_ESM.pdf]

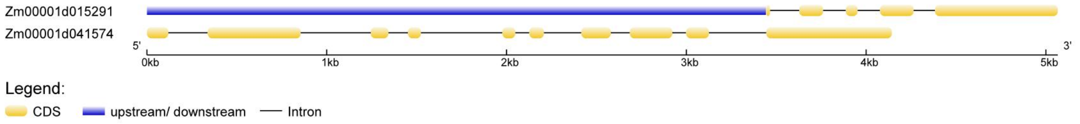

Gene structure of subfamily I.

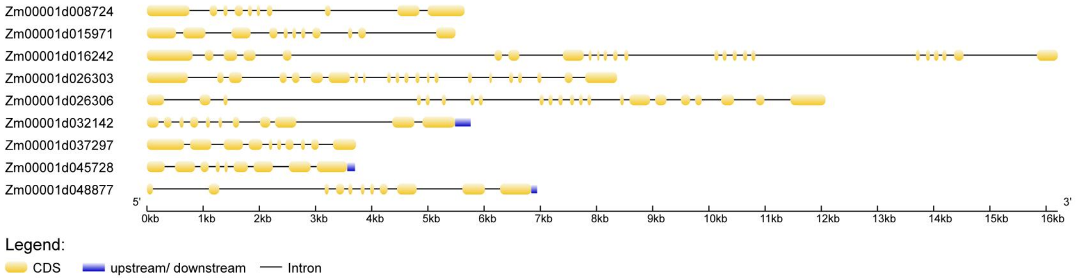

Gene structure of subfamily II.

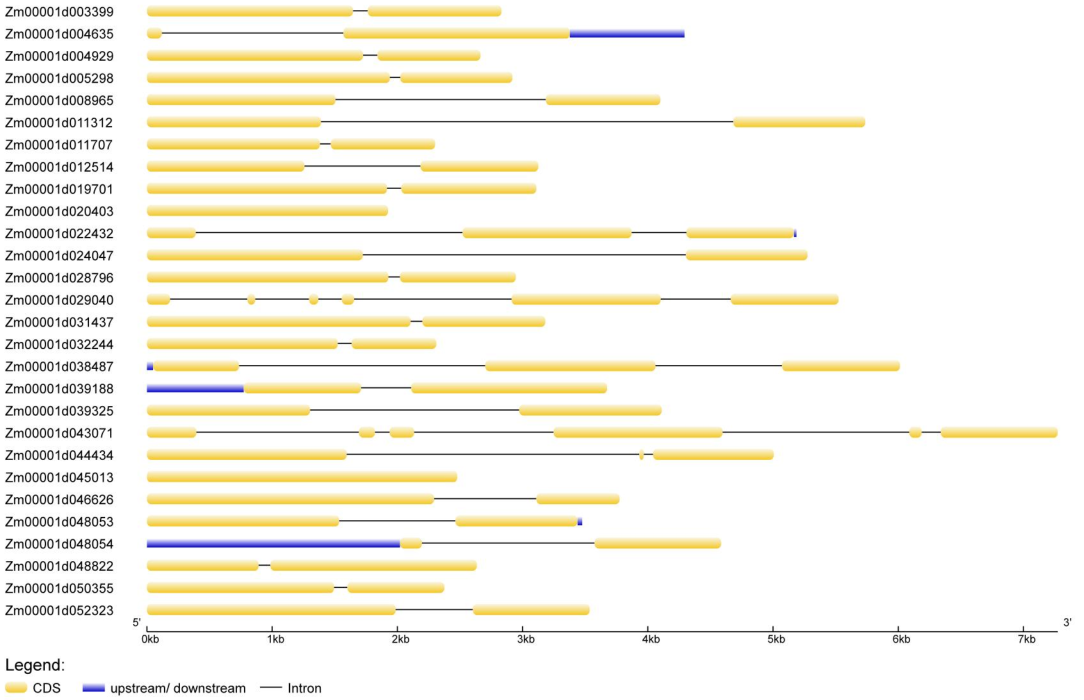

Gene structure of subfamily III.

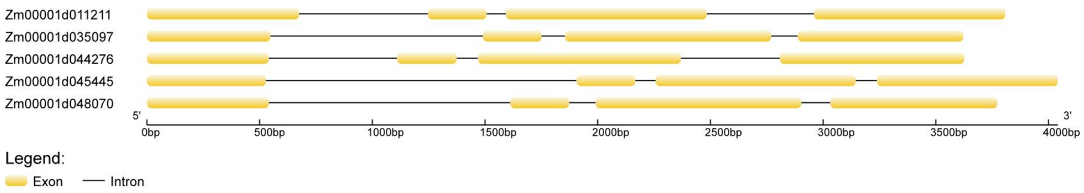

Gene structure of subfamily IV.

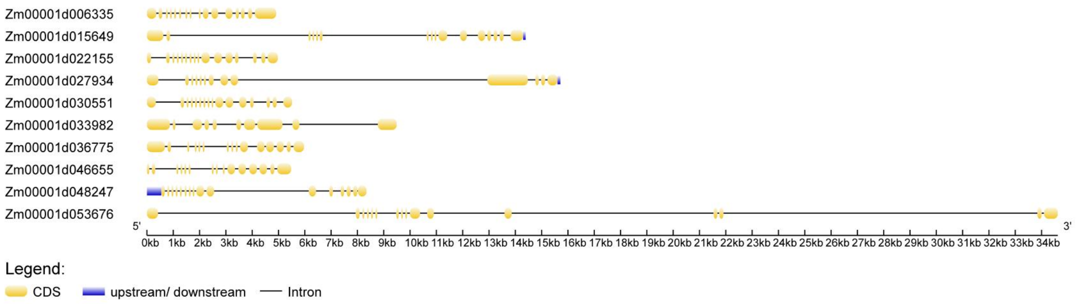

Gene structure of subfamily V.

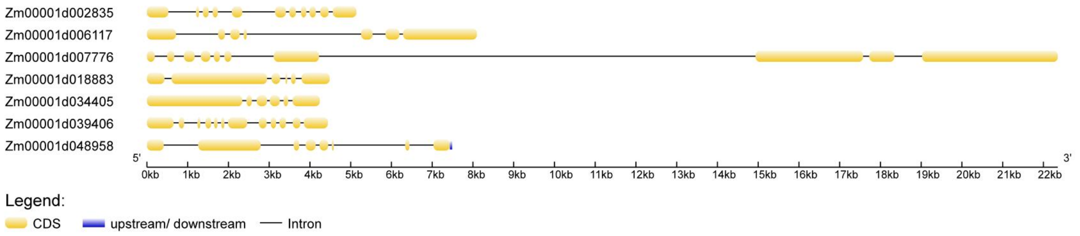

Gene structure of subfamily VI.

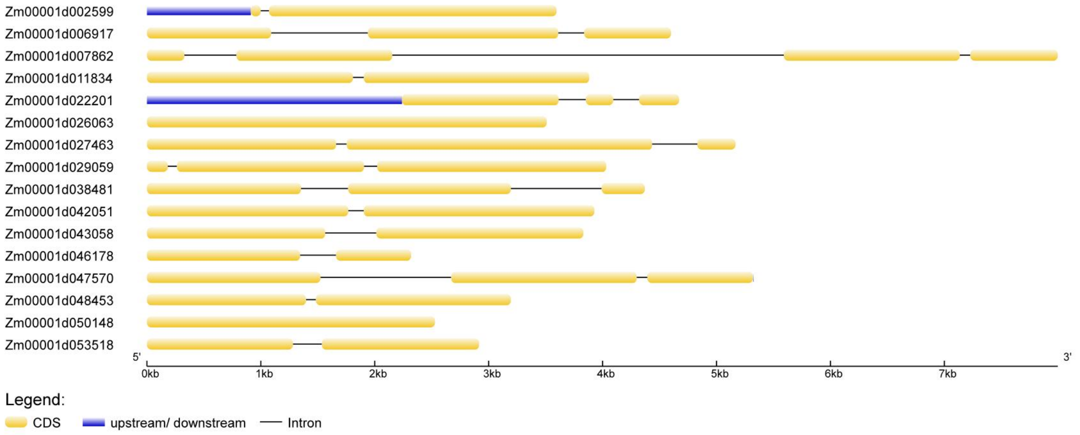

Gene structure of subfamily VII.

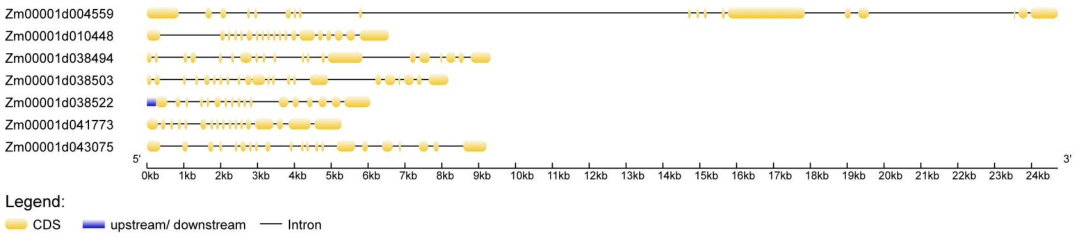

Gene structure of subfamily VIII.

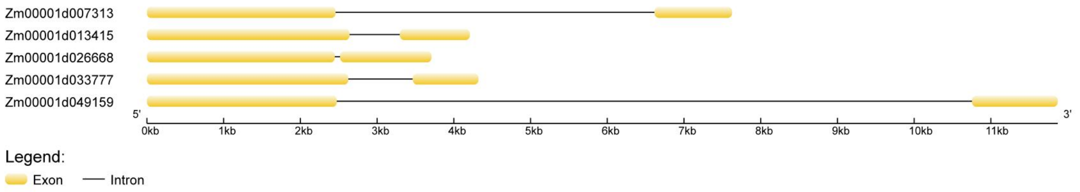

Gene structure of subfamily IX.

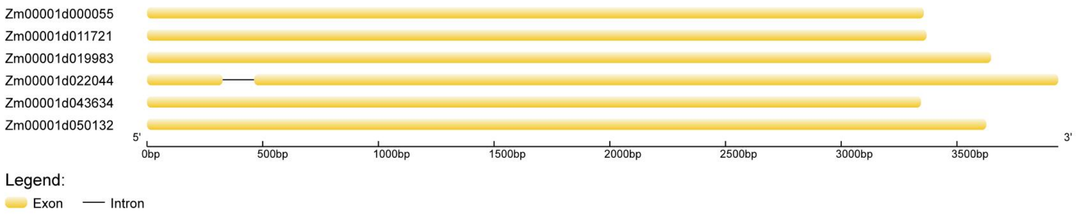

Gene structure of subfamily X.

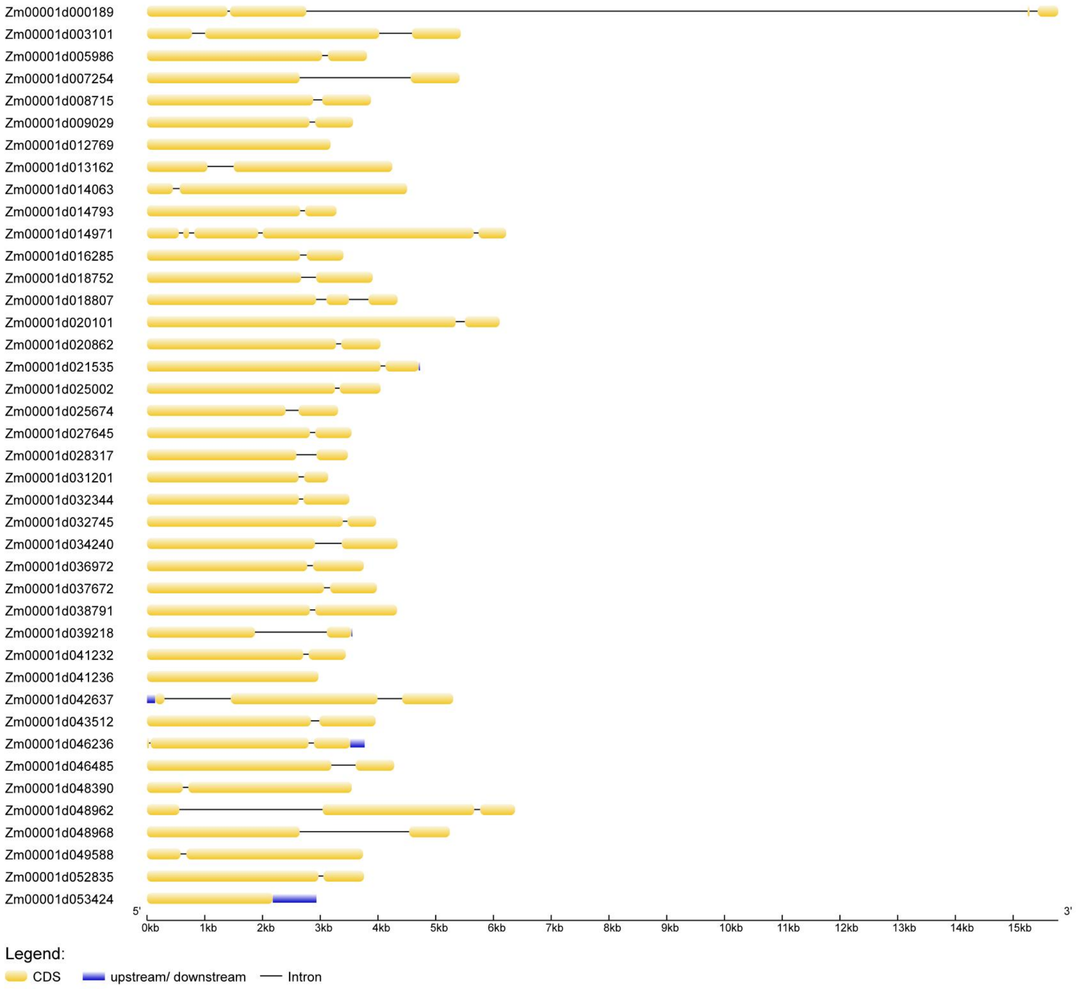

Gene structure of subfamily XI.

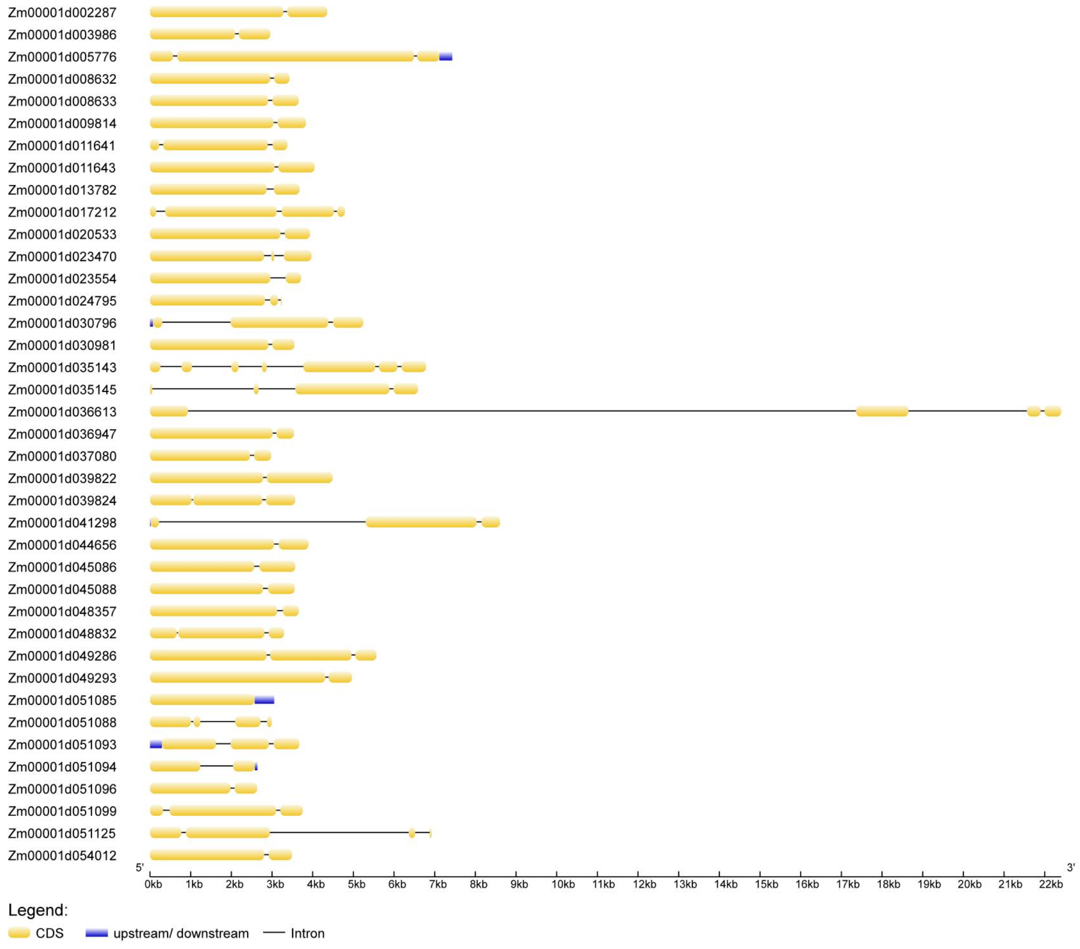

Gene structure of subfamily XII.

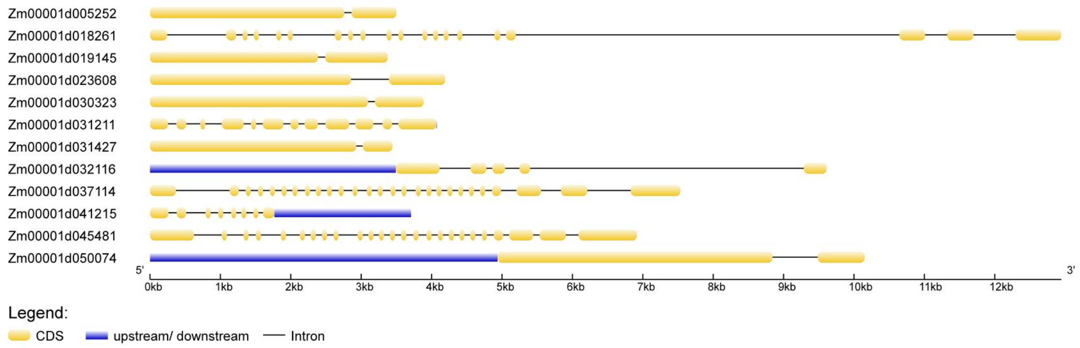

Gene structure of subfamily XIII.

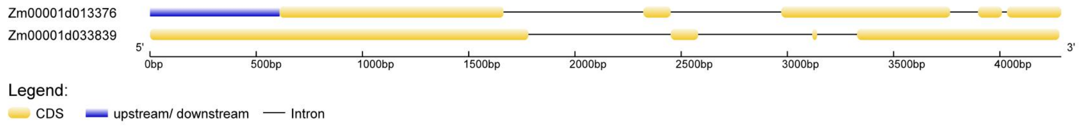

Gene structure of subfamily XIV.

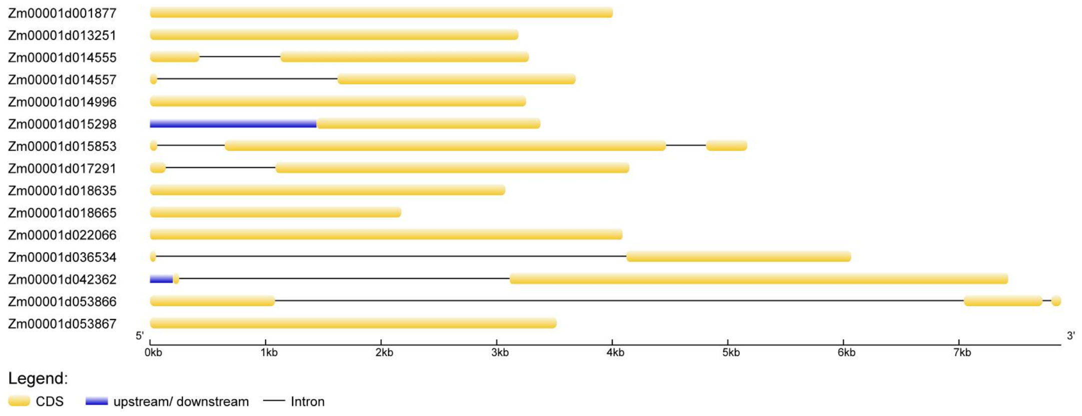

Gene structure of subfamily XV.
