# Supplementary material for: Genome identification of the LRR-RLK gene family in maize (Zea mays) and expression analysis in response to Fusarium verticillioides infection
Source: BMC Plant Biol. 2025 Apr 25;25:524. doi: 10.1186/s12870-025-06495-w (PMC12023693; doi:10.1186/s12870-025-06495-w)
Supplement: Supplementary file 2 — Supplementary Material 2 [file 12870_2025_6495_MOESM2_ESM.pdf]

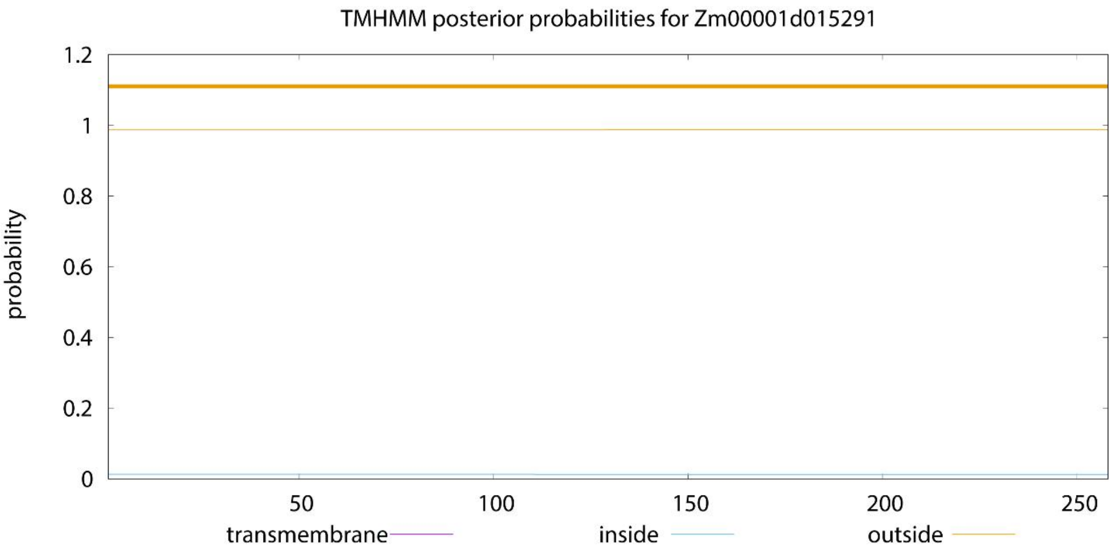

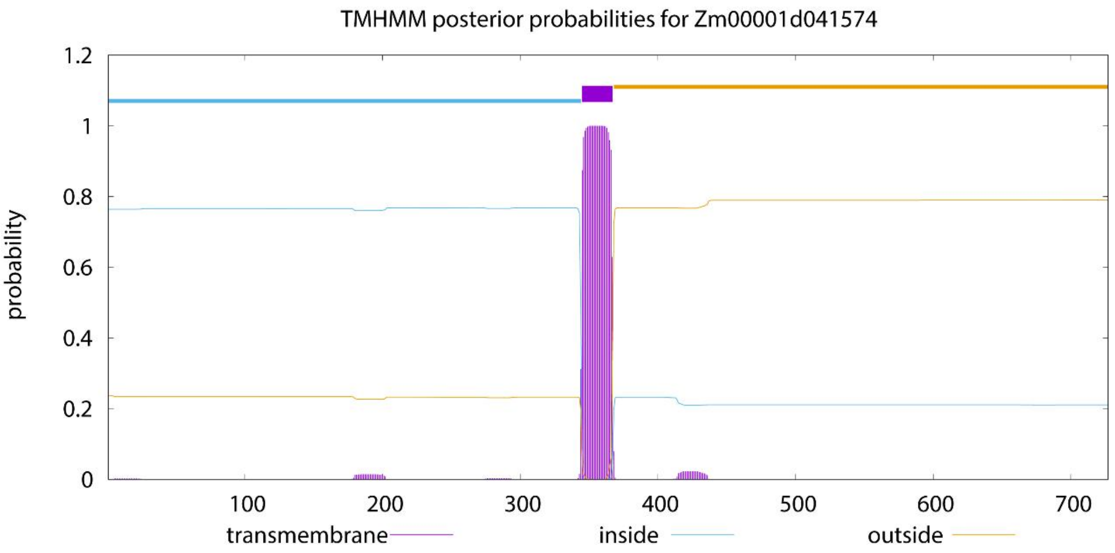

Transmembrane structure domain of subfamily I.

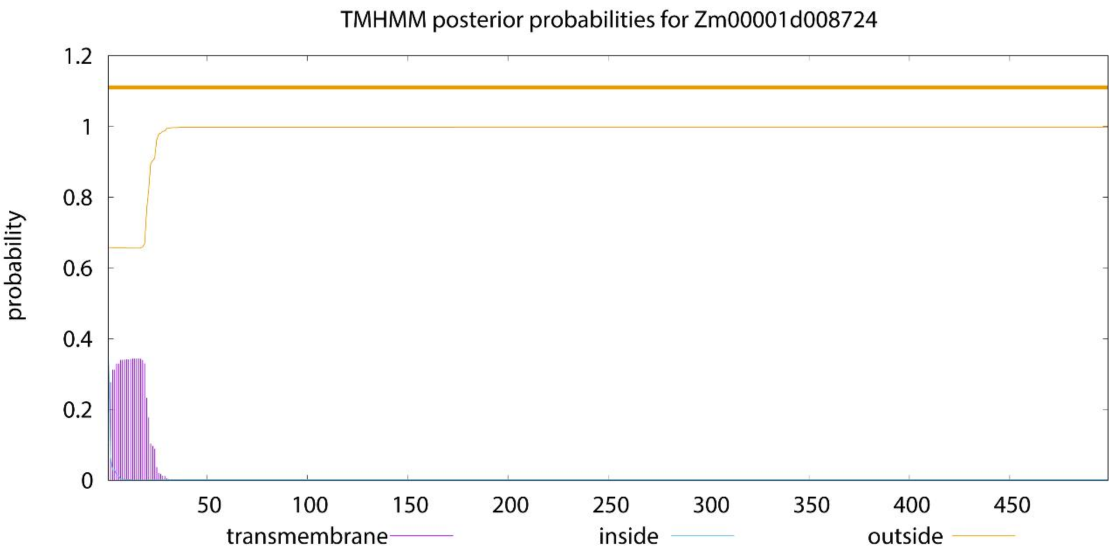

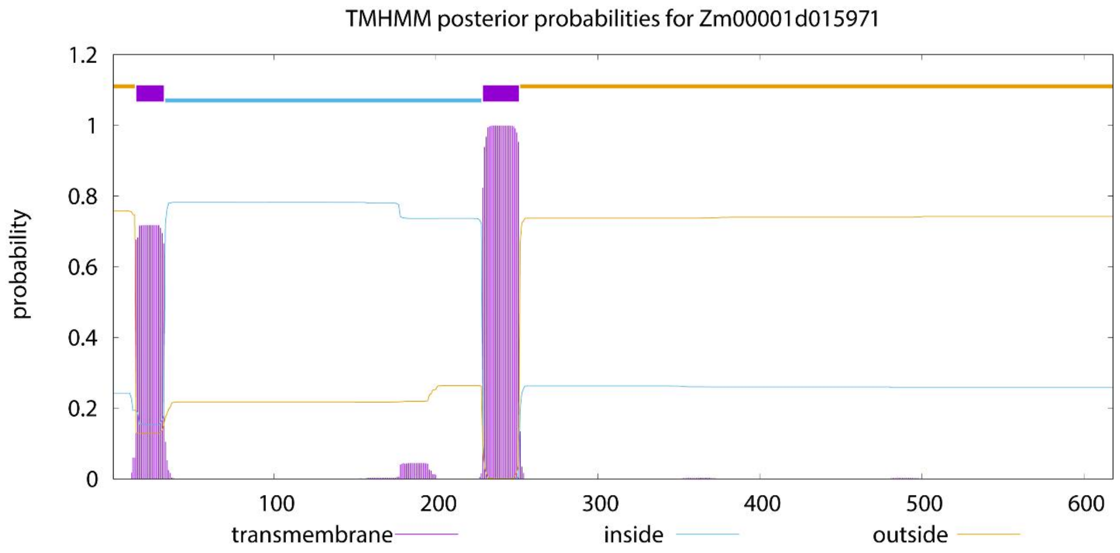
TMHMM posterior probabilities for Zm00001d015971
1.2
1
0.8
0.6
0.4
0.2
0
probability
100
200
300
400
500
600
transmembrane
inside
outside

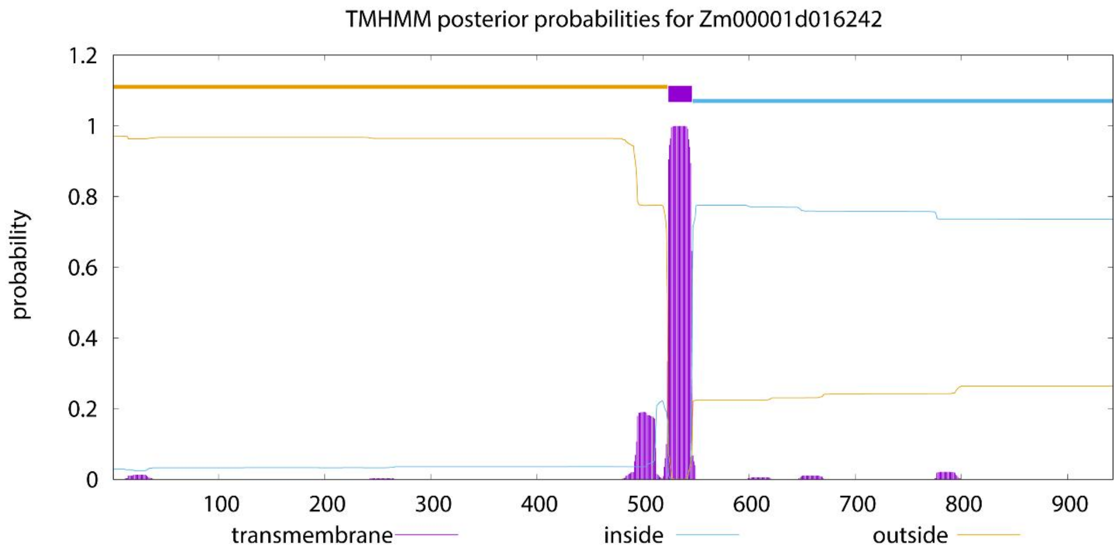
TMHMM posterior probabilities for Zm00001d016242
1.2
1
0.8
0.6
0.4
0.2
0
probability
100
200
300
400
500
600
700
800
900
transmembrane
inside
outside

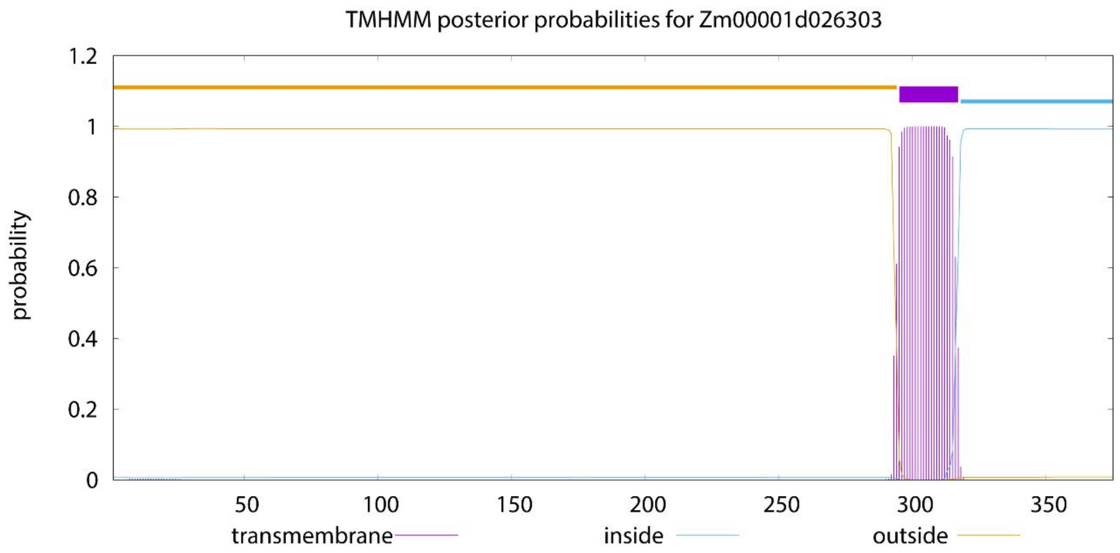
TMHMM posterior probabilities for Zm00001d026303
1.2
1
0.8
0.6
0.4
0.2
0
probability
50
100
150
200
250
300
350
transmembrane
inside
outside

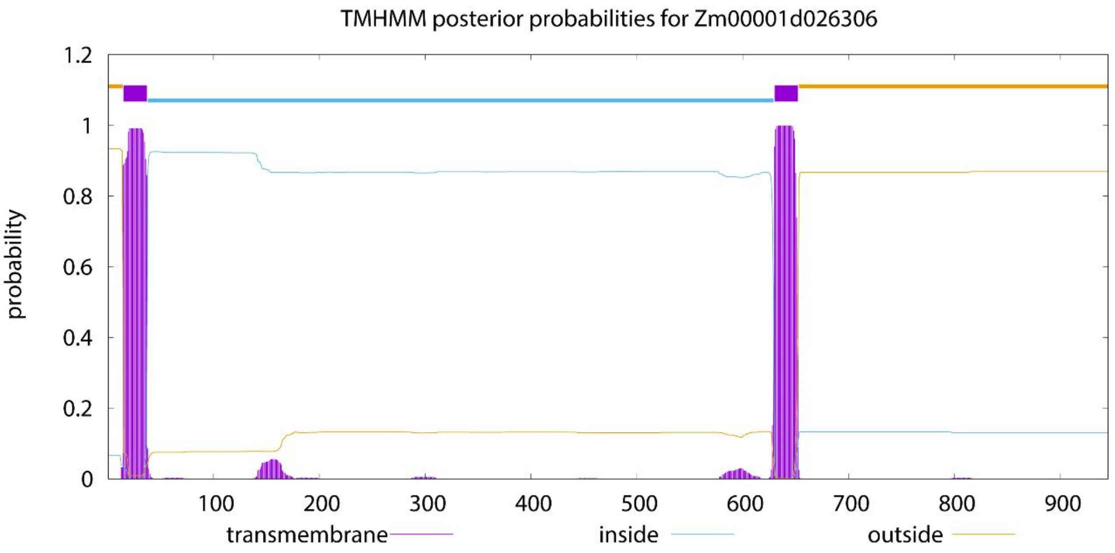
TMHMM posterior probabilities for Zm00001d026306
probability
1.2
1
0.8
0.6
0.4
0.2
0
100
200
300
400
500
600
700
800
900
transmembrane
inside
outside

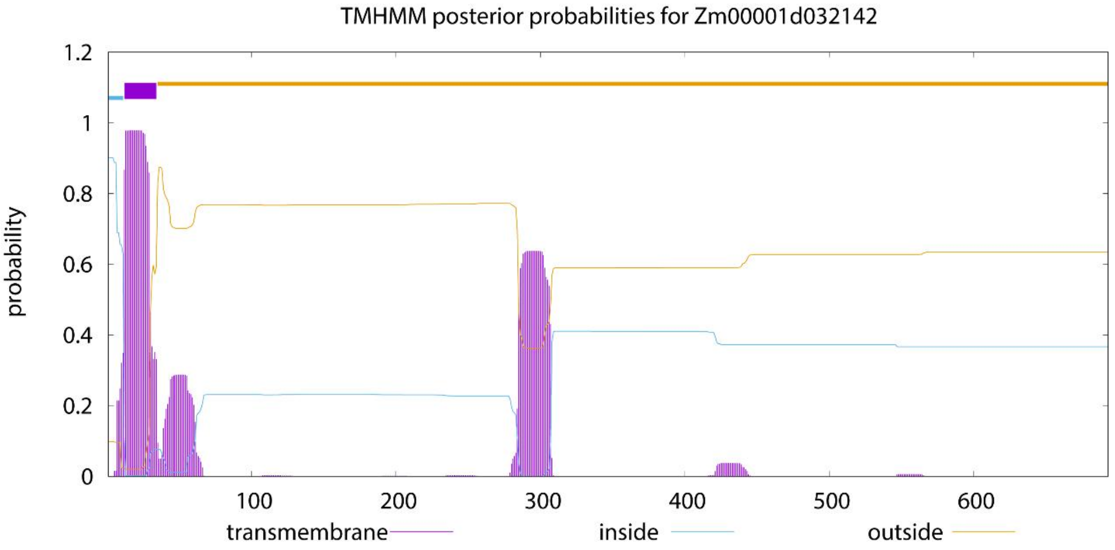
TMHMM posterior probabilities for Zm00001d032142
probability
1.2
1
0.8
0.6
0.4
0.2
0
100
200
300
400
500
600
transmembrane
inside
outside

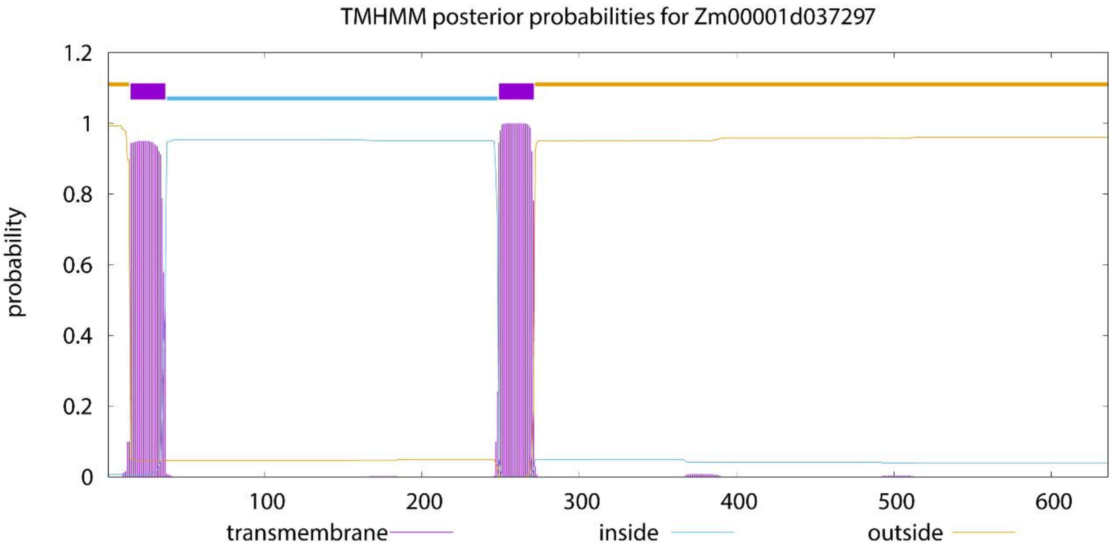
TMHMM posterior probabilities for Zm00001d037297
probability
1.2
1
0.8
0.6
0.4
0.2
0
100
200
300
400
500
600
transmembrane
inside
outside

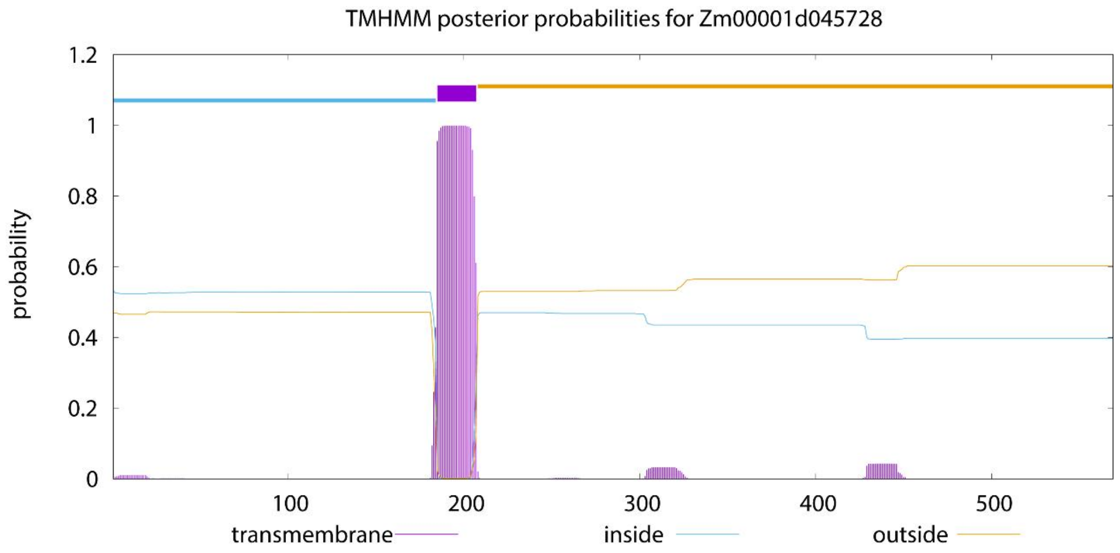

Transmembrane structure domain of subfamily II.

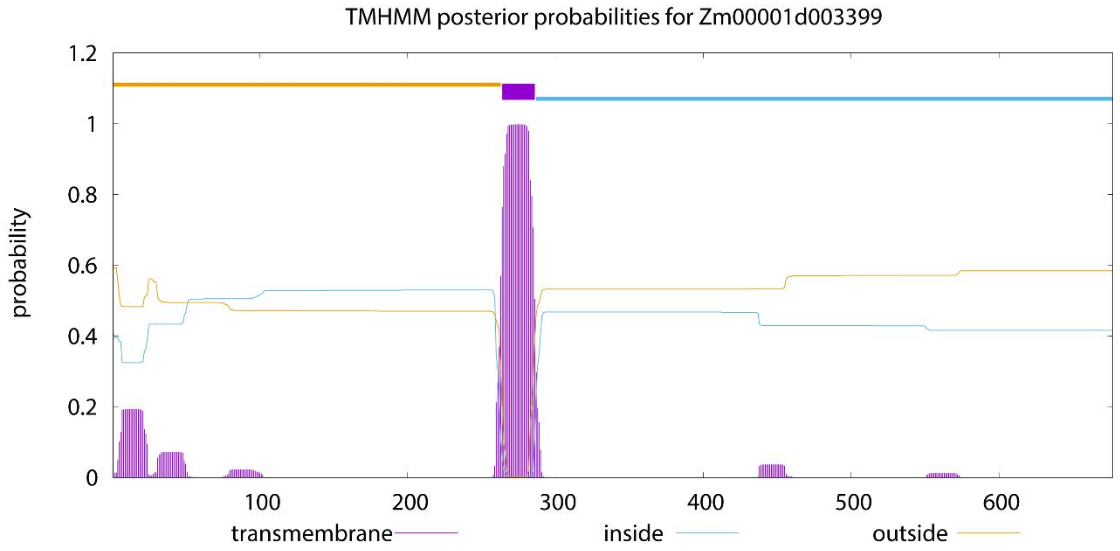

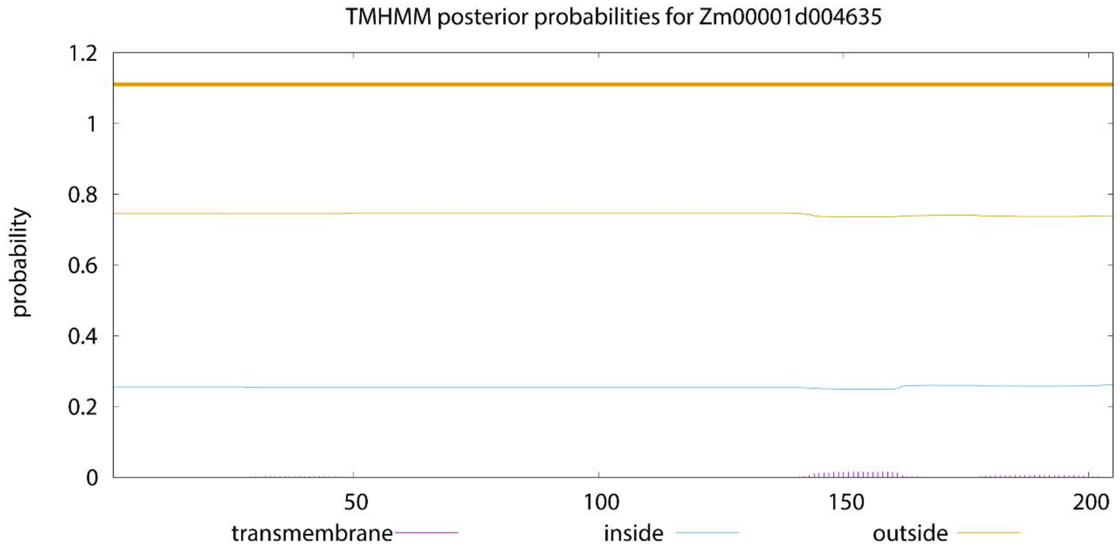

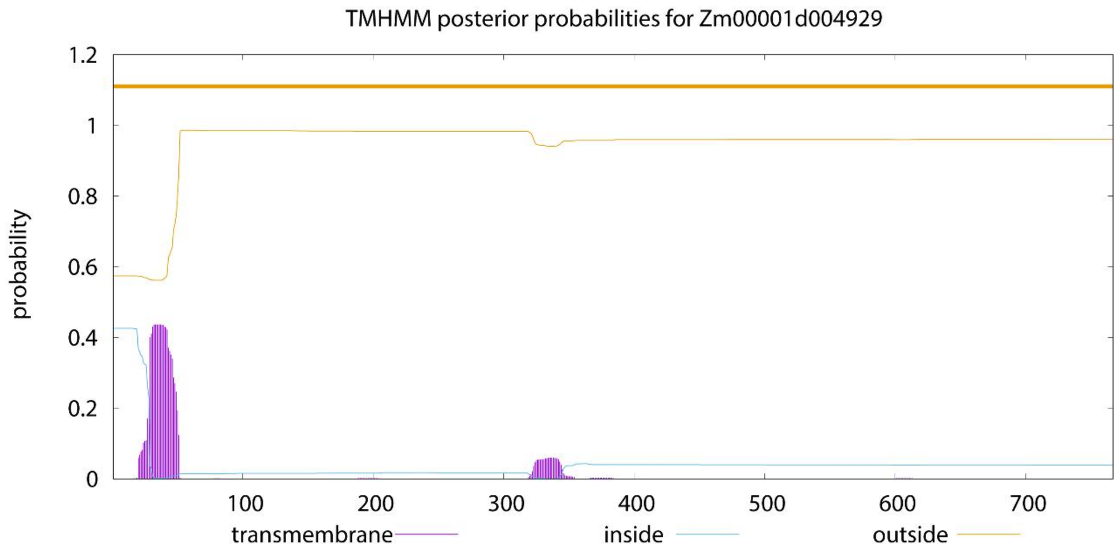
TMHMM posterior probabilities for Zm00001d004929
probability
1.2
1
0.8
0.6
0.4
0.2
0
100
200
300
400
500
600
700
transmembrane
inside
outside

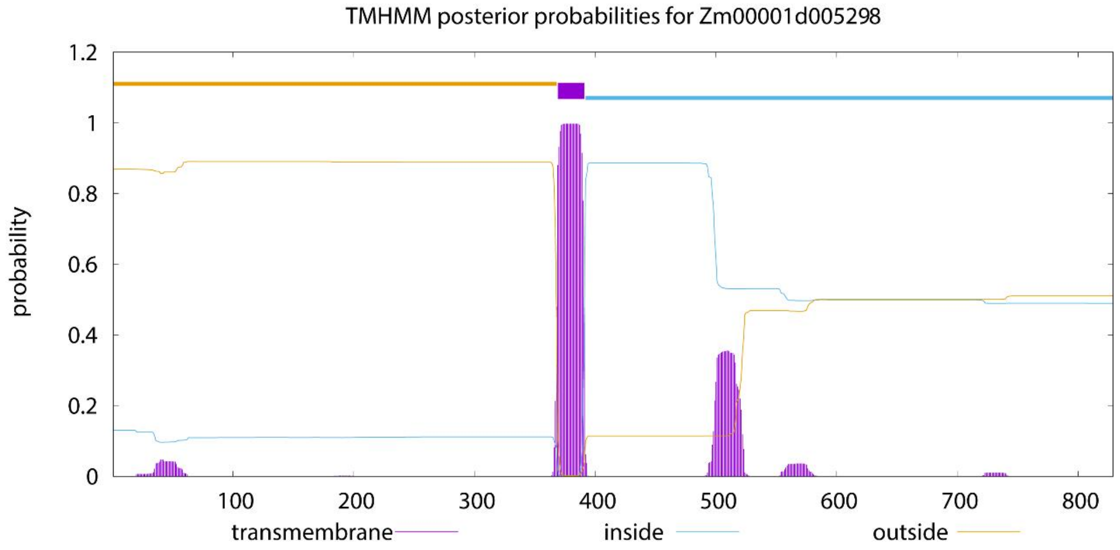
TMHMM posterior probabilities for Zm00001d005298
probability
1.2
1
0.8
0.6
0.4
0.2
0
100
200
300
400
500
600
700
800
transmembrane
inside
outside

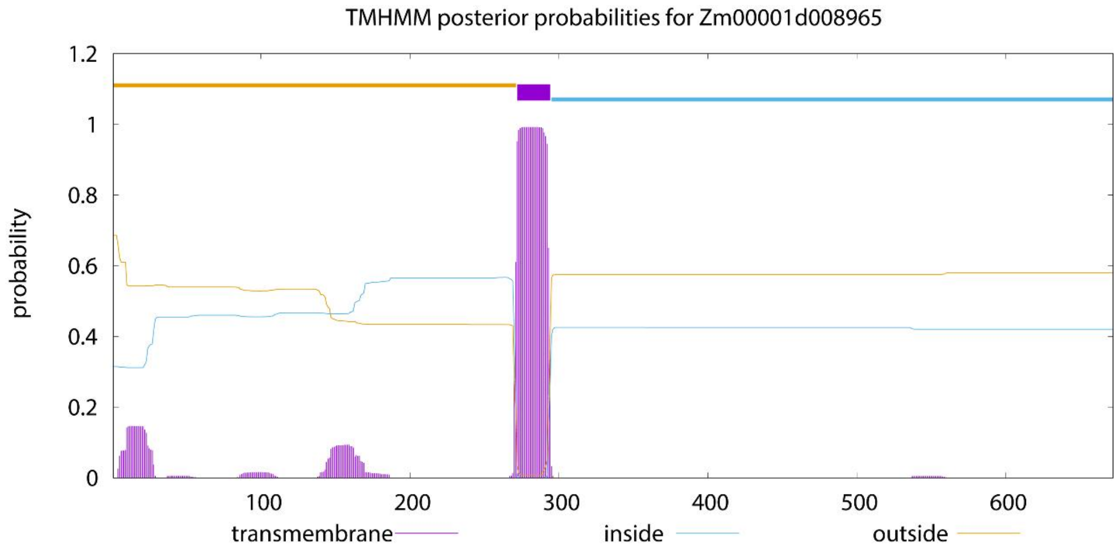
TMHMM posterior probabilities for Zm00001d008965
probability
1.2
1
0.8
0.6
0.4
0.2
0
100
200
300
400
500
600
transmembrane
inside
outside

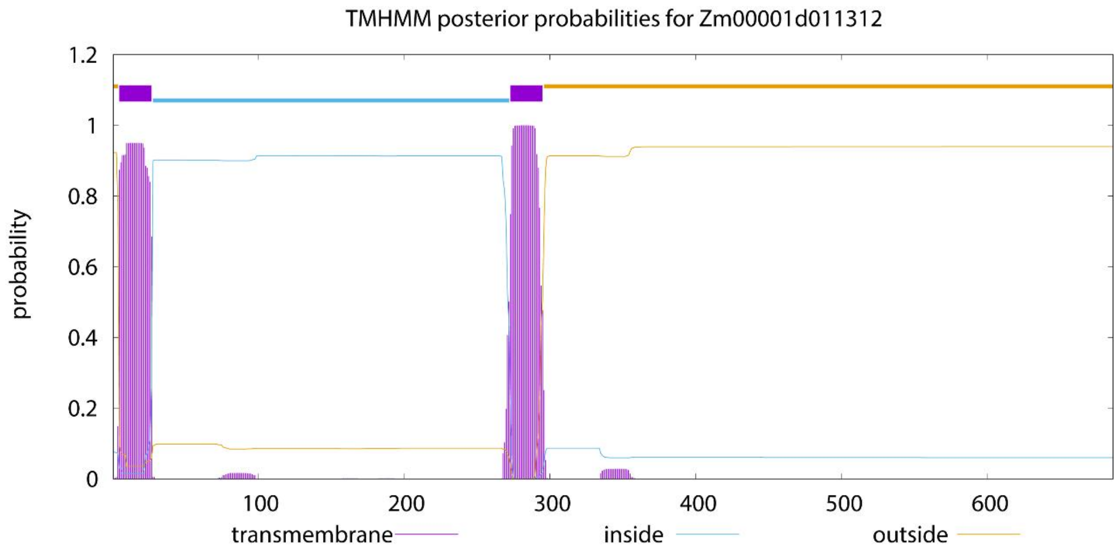
TMHMM posterior probabilities for Zm00001d011312
probability
1.2
1
0.8
0.6
0.4
0.2
0
100
200
300
400
500
600
transmembrane
inside
outside

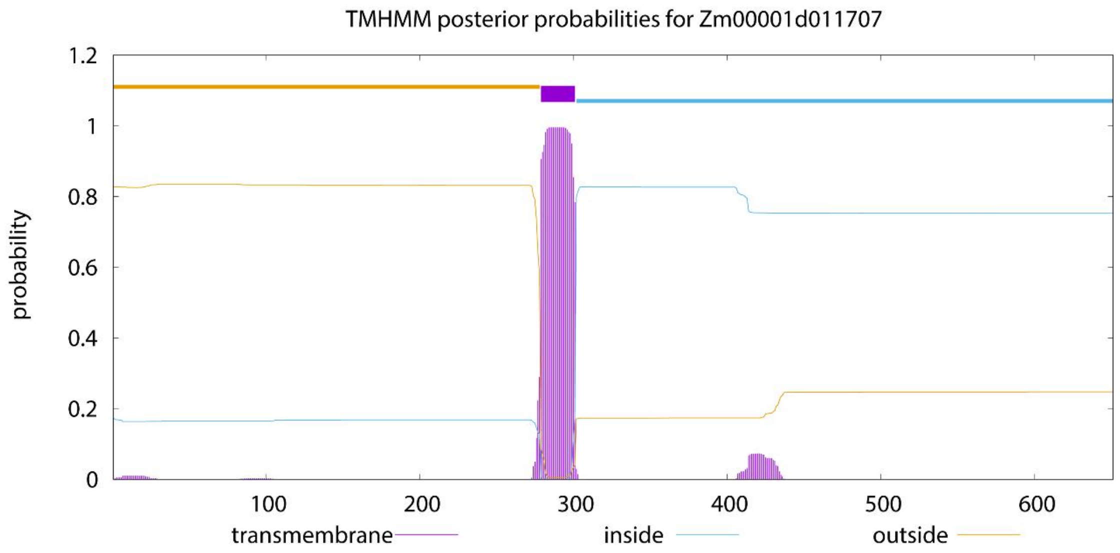
TMHMM posterior probabilities for Zm00001d011707
probability
1.2
1
0.8
0.6
0.4
0.2
0
100
200
300
400
500
600
transmembrane
inside
outside

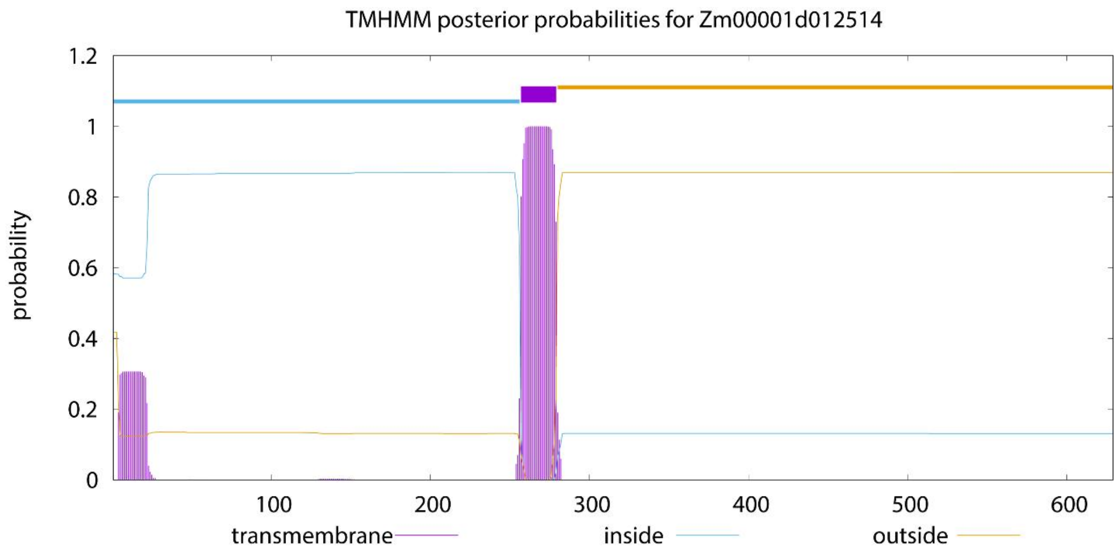
TMHMM posterior probabilities for Zm00001d012514
probability
1.2
1
0.8
0.6
0.4
0.2
0
100
200
300
400
500
600
transmembrane
inside
outside

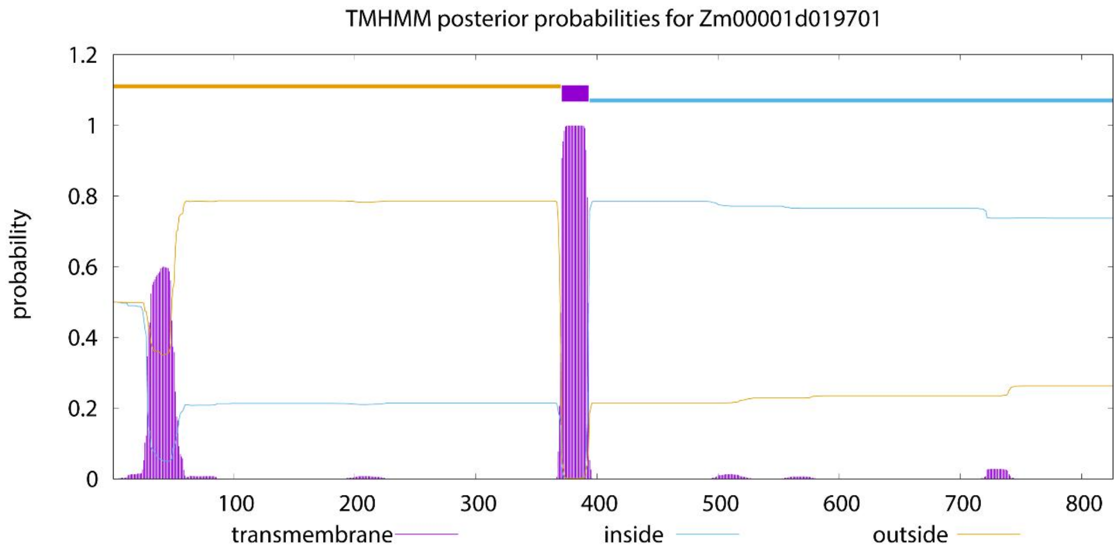
TMHMM posterior probabilities for Zm00001d019701
probability
transmembrane
inside
outside

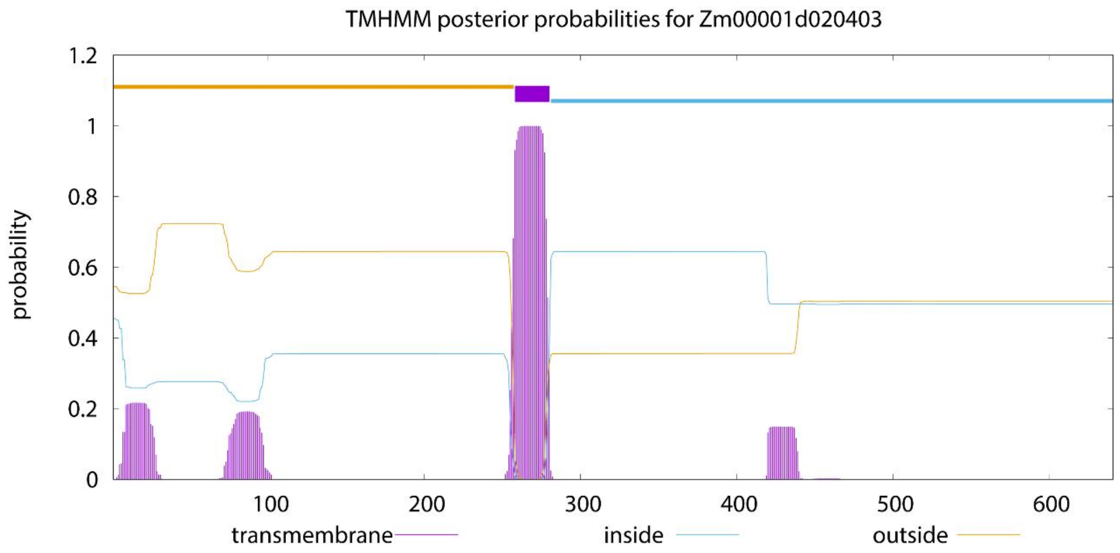
TMHMM posterior probabilities for Zm00001d020403
probability
transmembrane
inside
outside

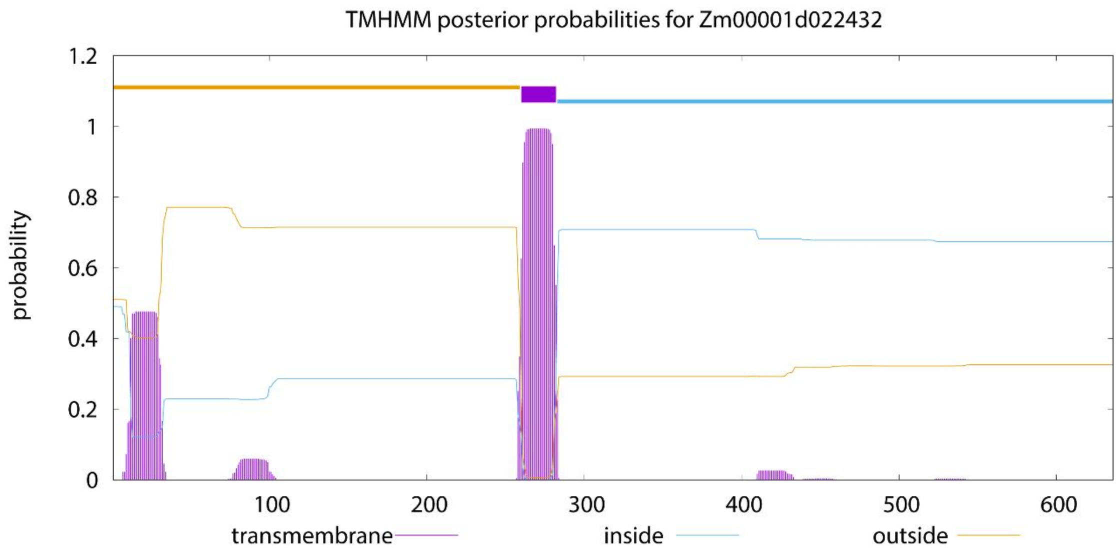
TMHMM posterior probabilities for Zm00001d022432
probability
transmembrane
inside
outside

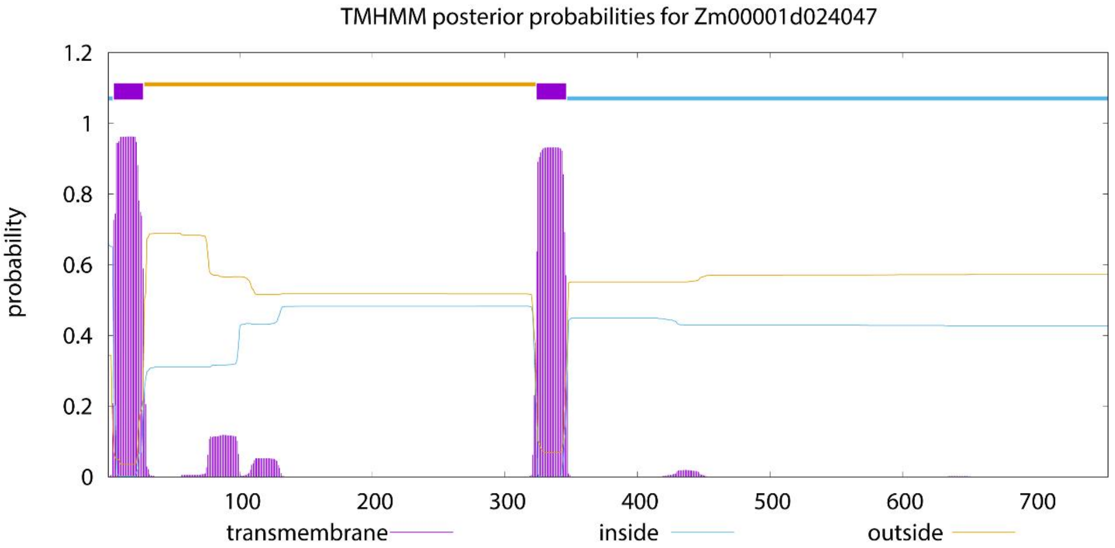
TMHMM posterior probabilities for Zm00001d024047
probability
1.2
1
0.8
0.6
0.4
0.2
0
100
200
300
400
500
600
700
transmembrane
inside
outside

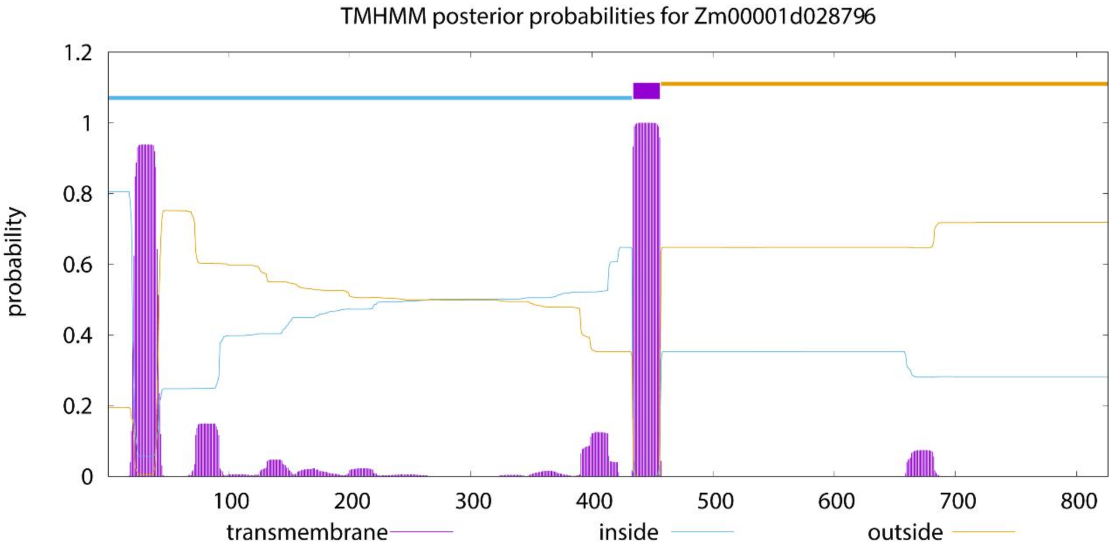
TMHMM posterior probabilities for Zm00001d028796
probability
1.2
1
0.8
0.6
0.4
0.2
0
100
200
300
400
500
600
700
800
transmembrane
inside
outside

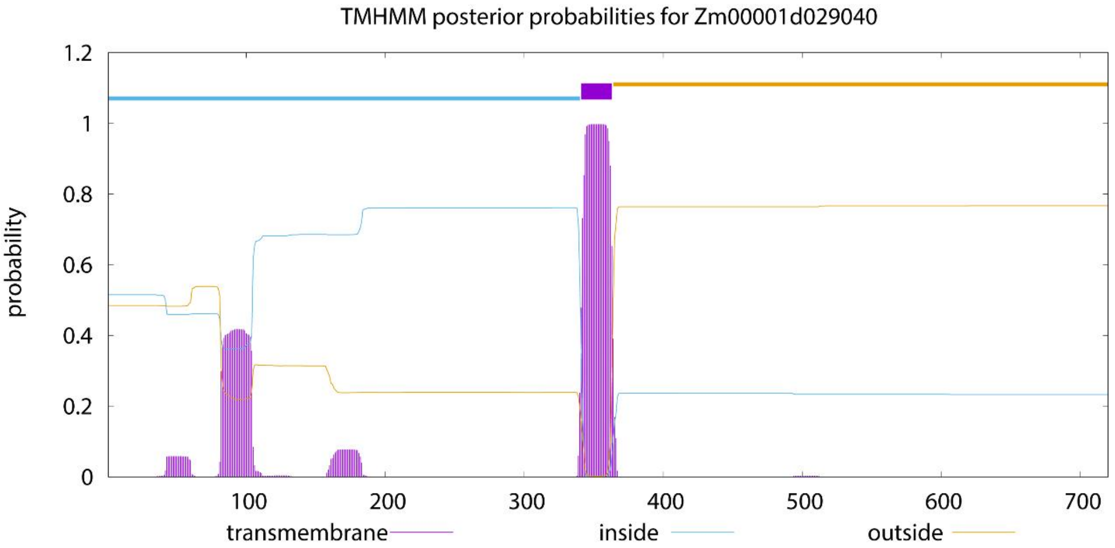
TMHMM posterior probabilities for Zm00001d029040
probability
1.2
1
0.8
0.6
0.4
0.2
0
100
200
300
400
500
600
700
transmembrane
inside
outside

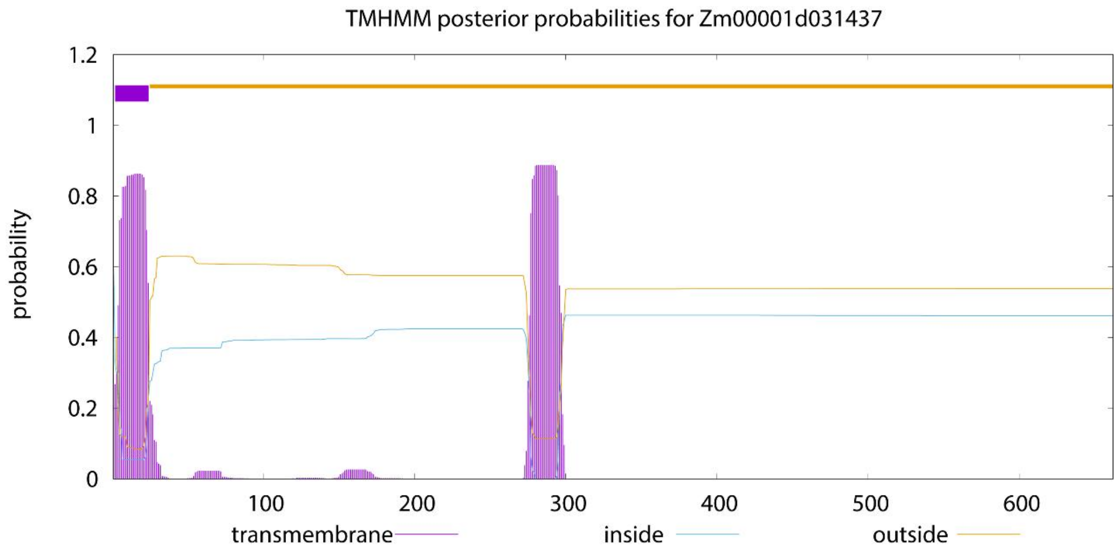
TMHMM posterior probabilities for Zm00001d031437
1.2
1
0.8
0.6
0.4
0.2
0
probability
100
200
300
400
500
600
transmembrane
inside
outside

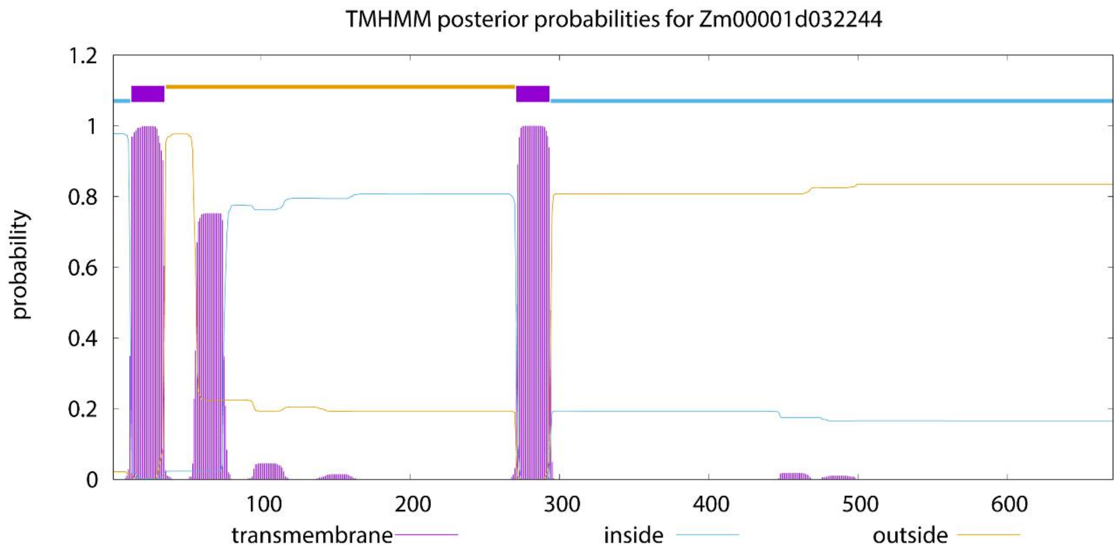
TMHMM posterior probabilities for Zm00001d032244
1.2
1
0.8
0.6
0.4
0.2
0
probability
100
200
300
400
500
600
transmembrane
inside
outside

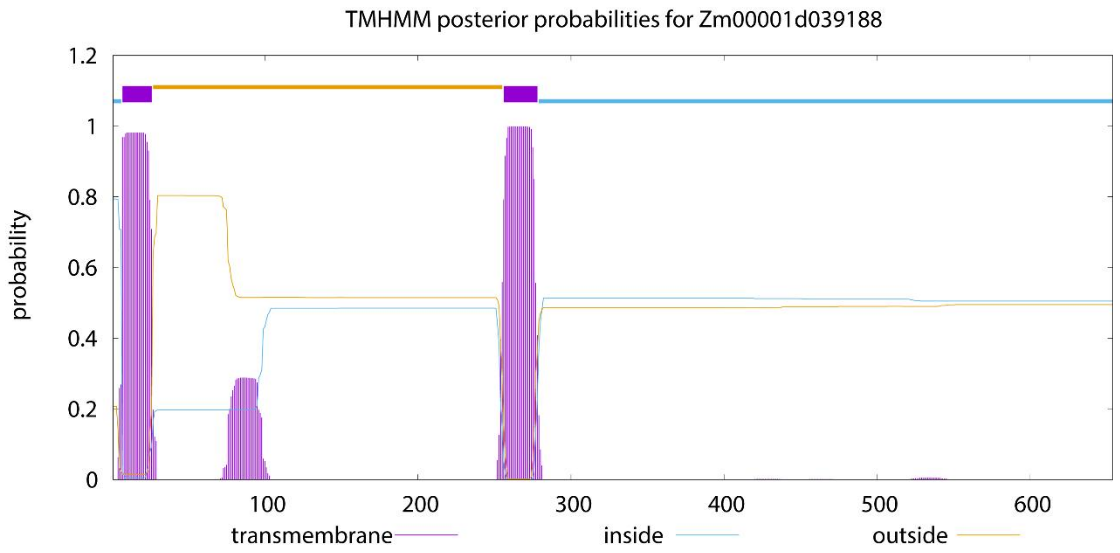
TMHMM posterior probabilities for Zm00001d039188
1.2
1
0.8
0.6
0.4
0.2
0
probability
100
200
300
400
500
600
transmembrane
inside
outside

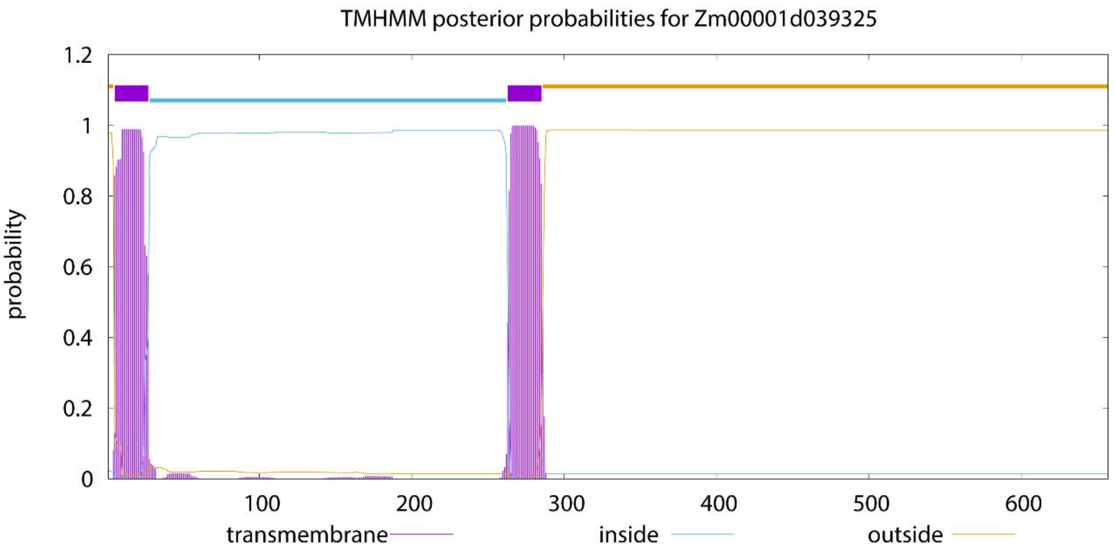
TMHMM posterior probabilities for Zm00001d039325
probability
1.2
1
0.8
0.6
0.4
0.2
0
100
200
300
400
500
600
transmembrane
inside
outside

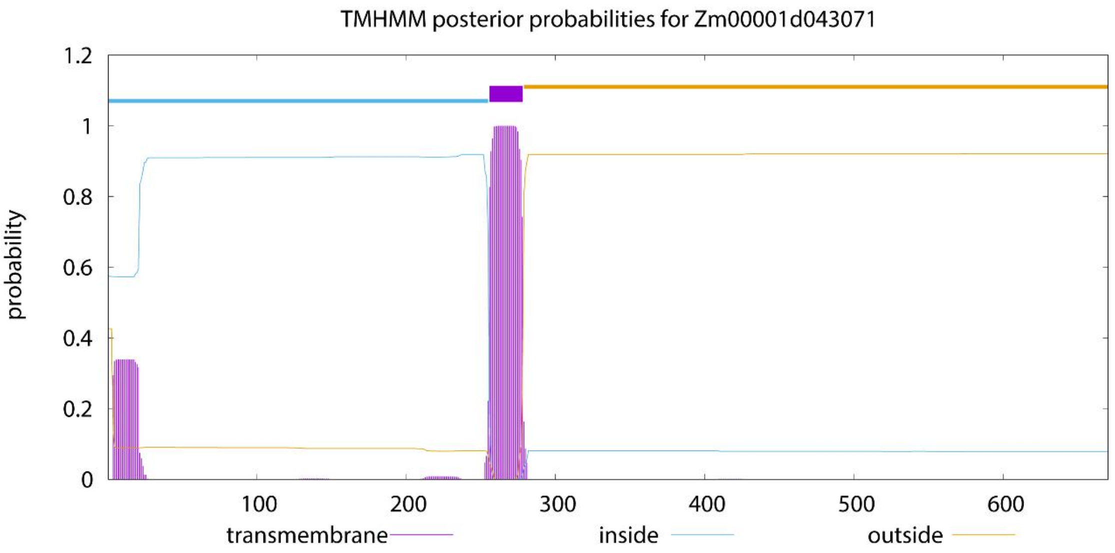
TMHMM posterior probabilities for Zm00001d043071
probability
1.2
1
0.8
0.6
0.4
0.2
0
100
200
300
400
500
600
transmembrane
inside
outside

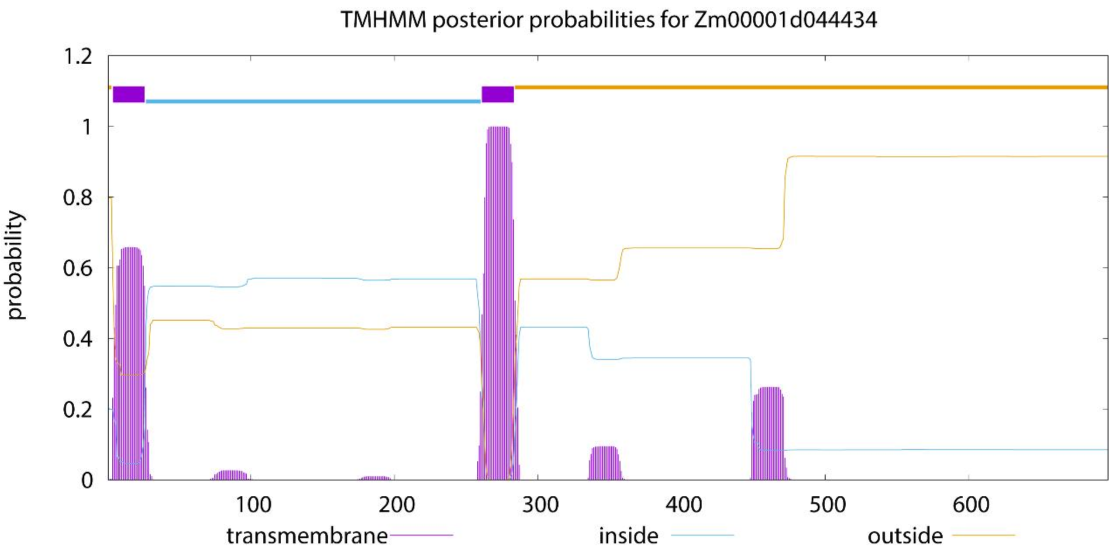
TMHMM posterior probabilities for Zm00001d044434
probability
1.2
1
0.8
0.6
0.4
0.2
0
100
200
300
400
500
600
transmembrane
inside
outside

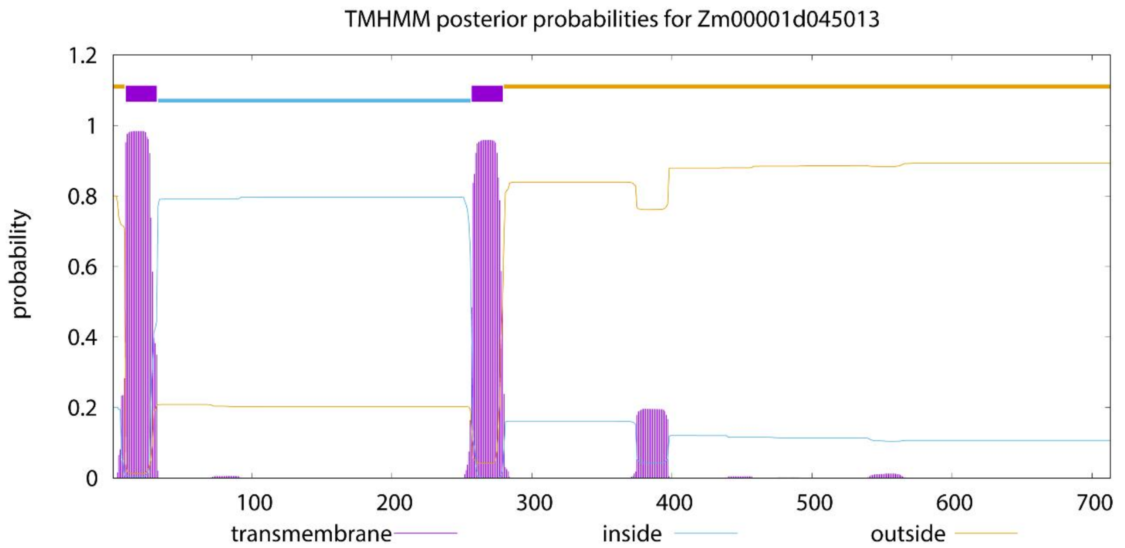
TMHMM posterior probabilities for Zm00001d045013
probability
transmembrane
inside
outside

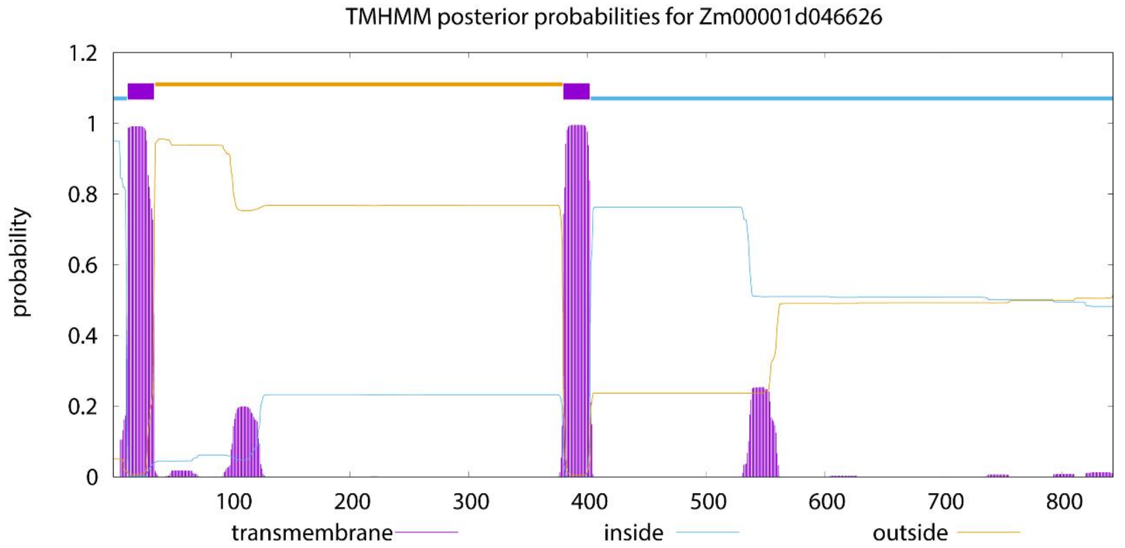
TMHMM posterior probabilities for Zm00001d046626
probability
transmembrane
inside
outside

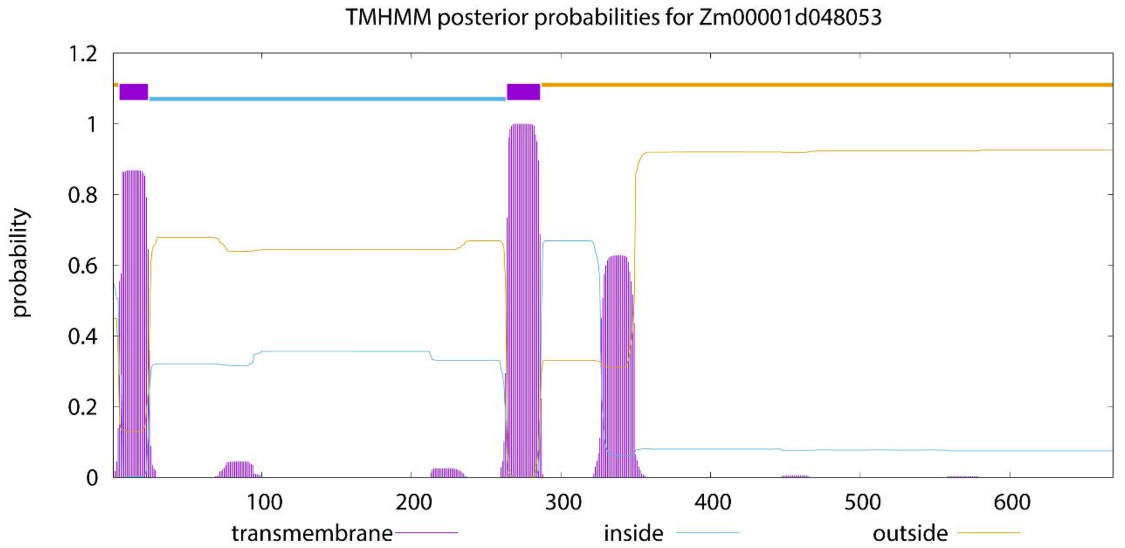
TMHMM posterior probabilities for Zm00001d048053
probability
transmembrane
inside
outside

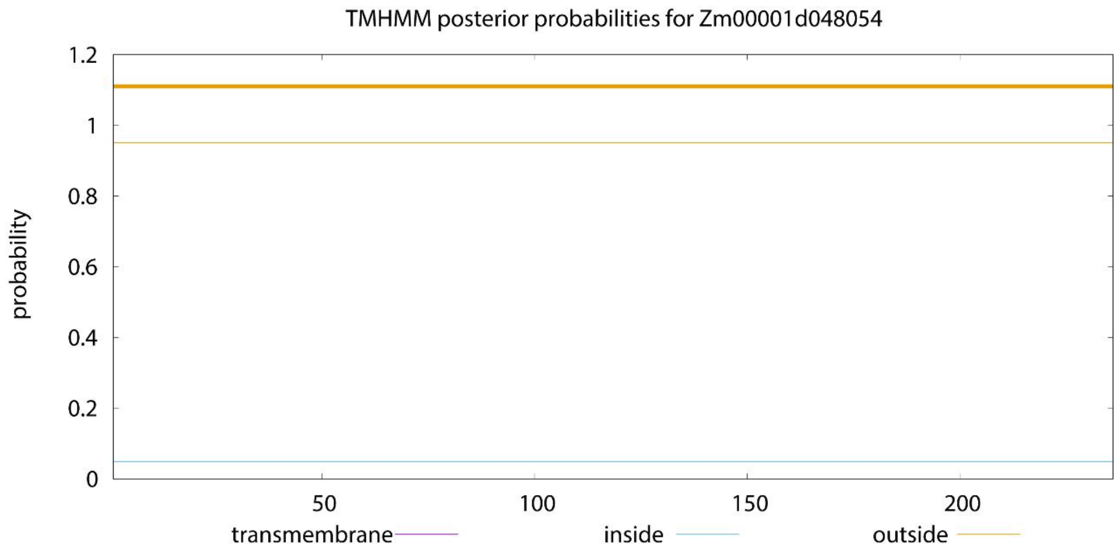
TMHMM posterior probabilities for Zm00001d048054
probability
1.2
1
0.8
0.6
0.4
0.2
0
50
100
150
200
transmembrane
inside
outside

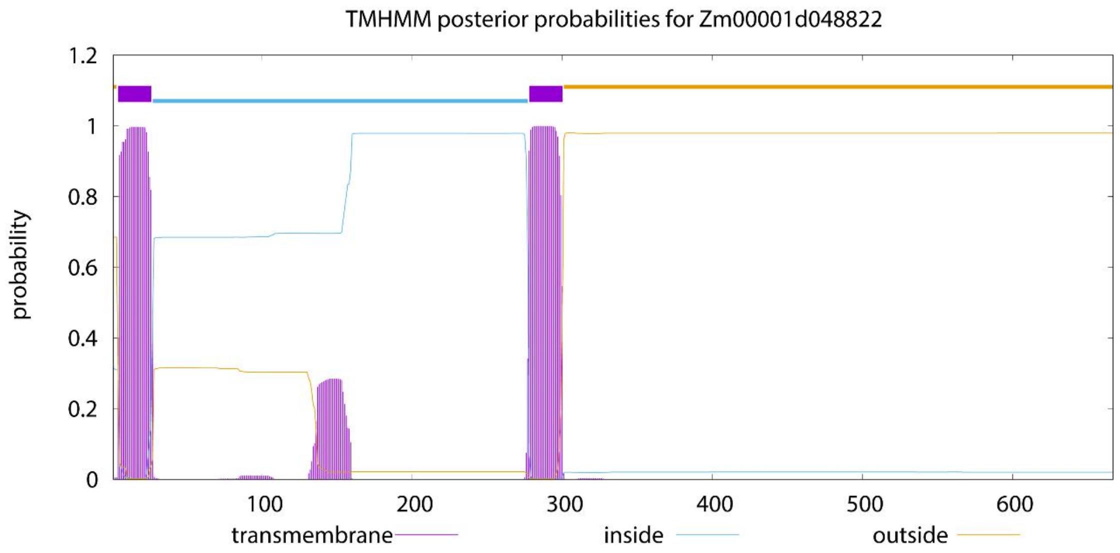
TMHMM posterior probabilities for Zm00001d048822
probability
1.2
1
0.8
0.6
0.4
0.2
0
100
200
300
400
500
600
transmembrane
inside
outside

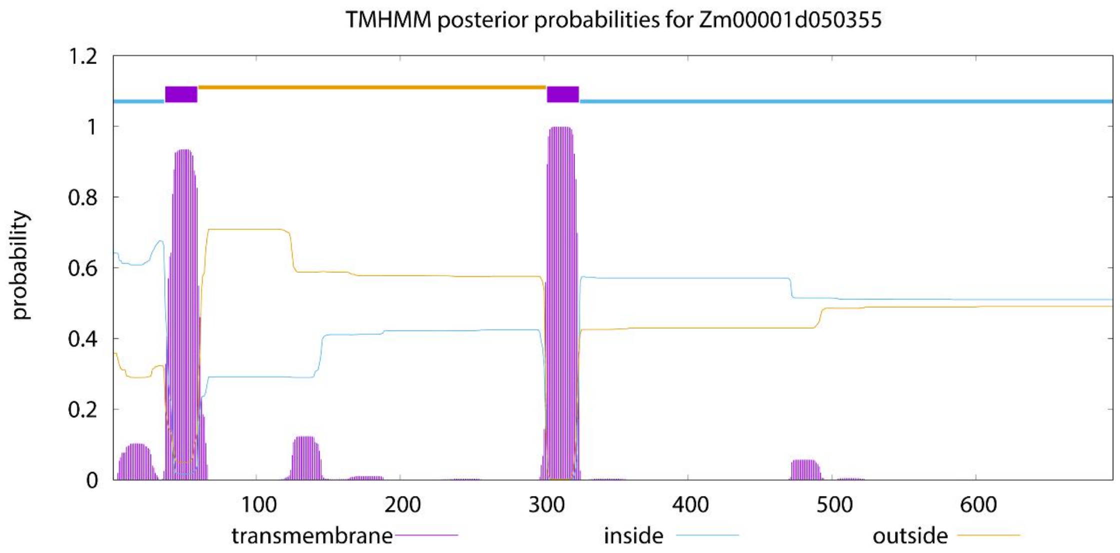
TMHMM posterior probabilities for Zm00001d050355
probability
1.2
1
0.8
0.6
0.4
0.2
0
100
200
300
400
500
600
transmembrane
inside
outside

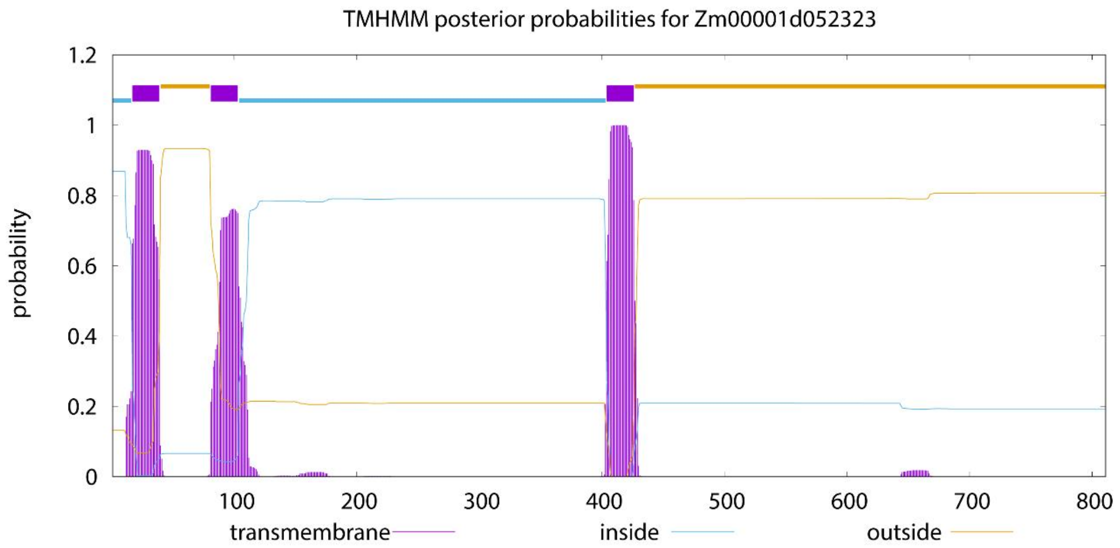

Transmembrane structure domain of subfamily III.

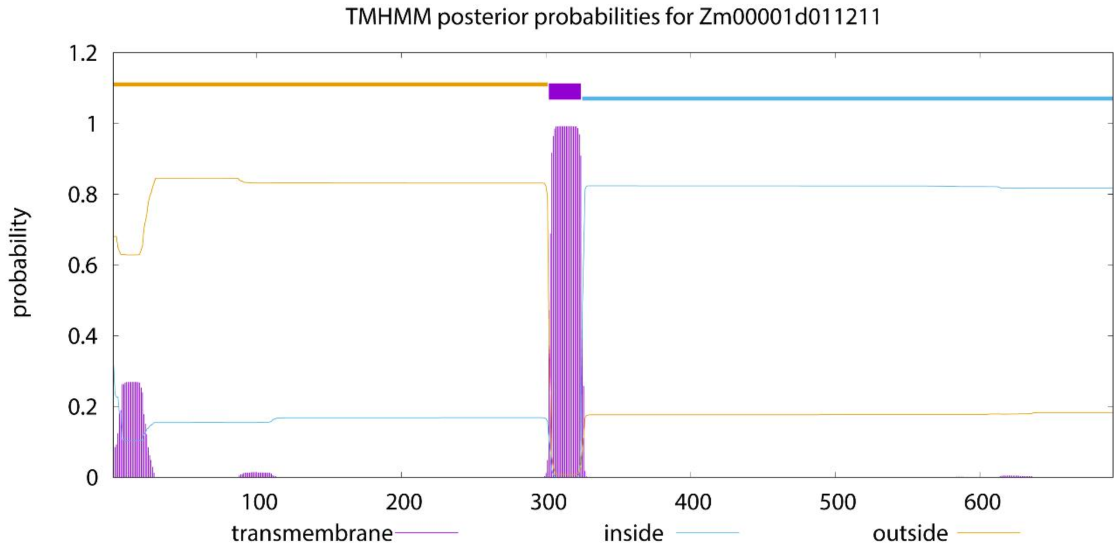

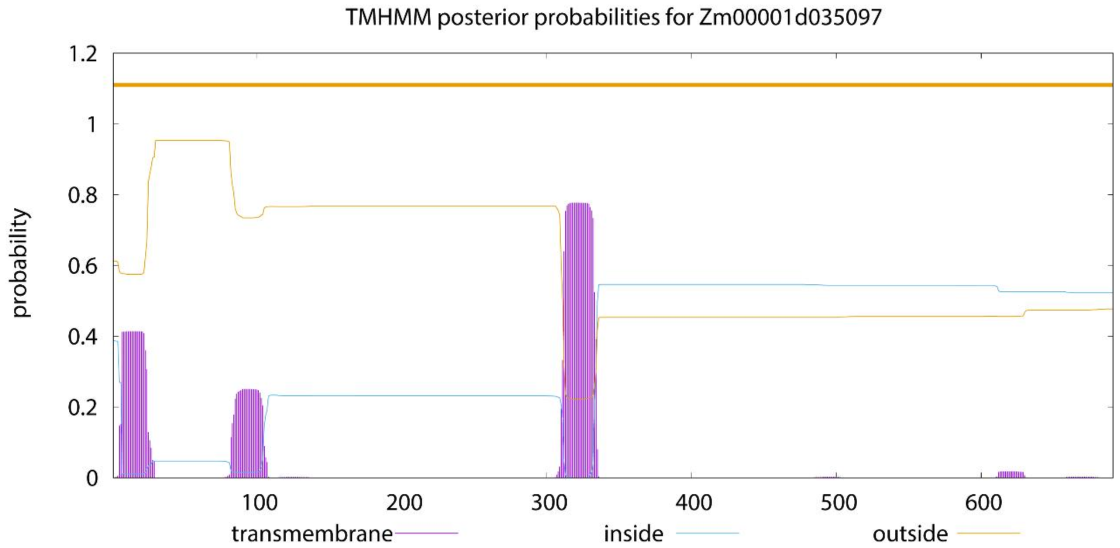

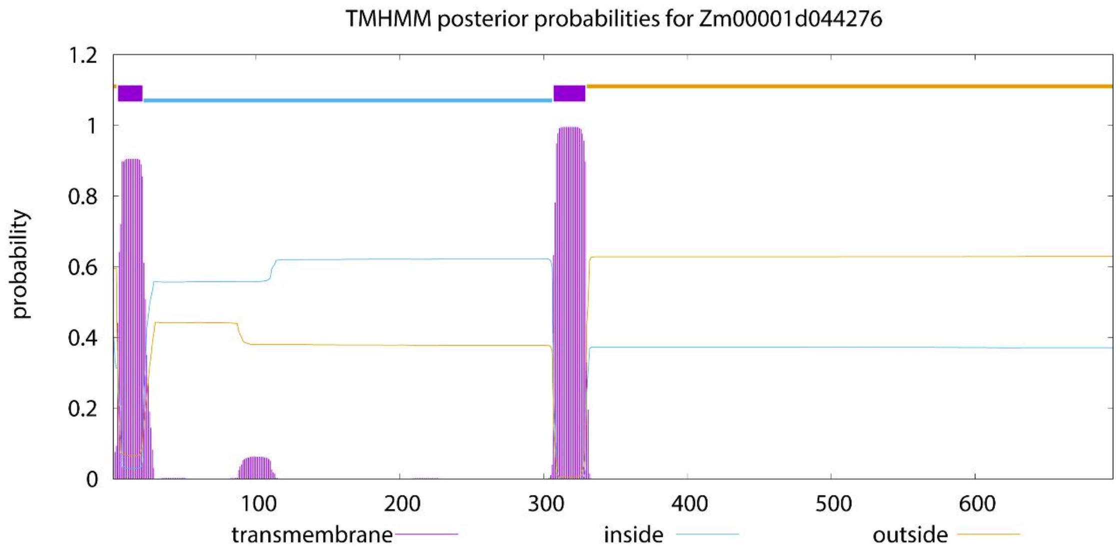

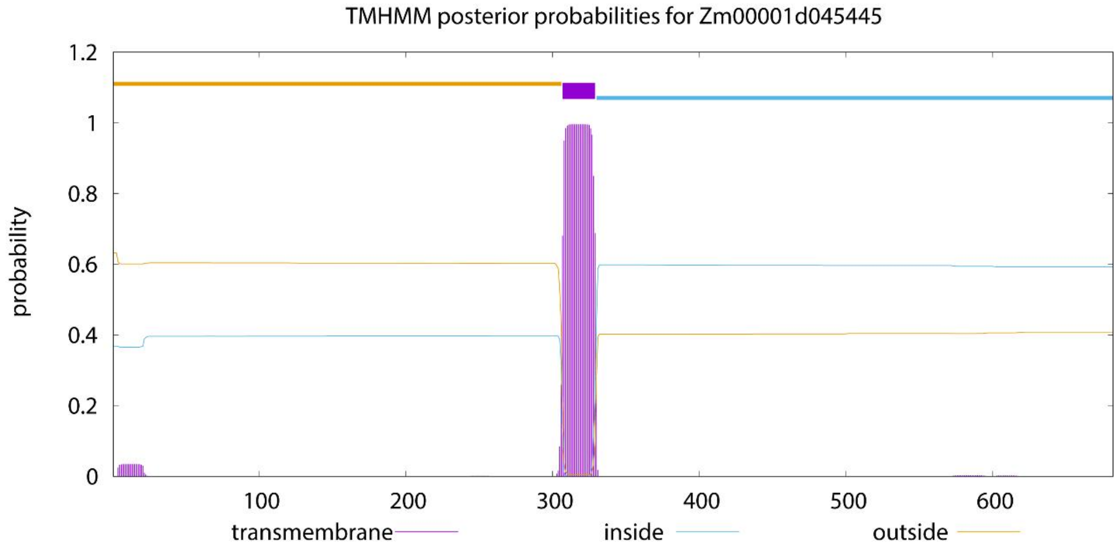

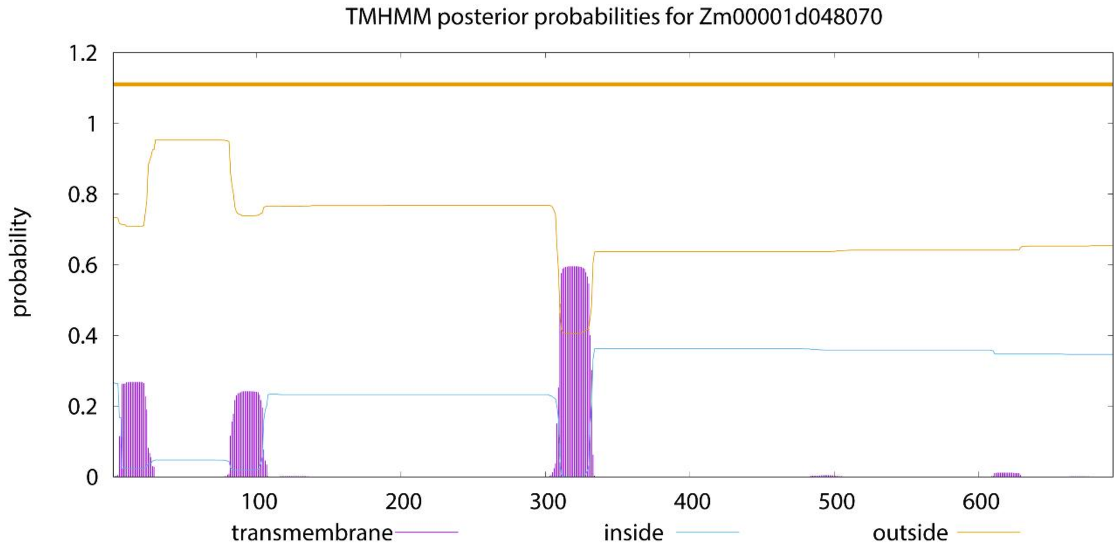

Transmembrane structure domain of subfamily IV.

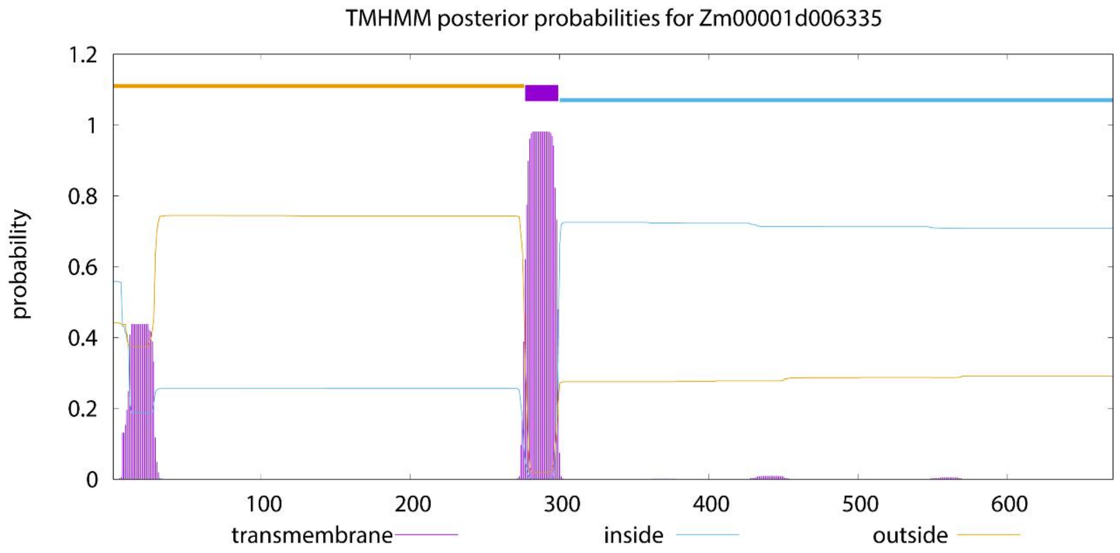
TMHMM posterior probabilities for Zm00001d006335
probability
1.2
1
0.8
0.6
0.4
0.2
0
100
200
300
400
500
600
transmembrane
inside
outside

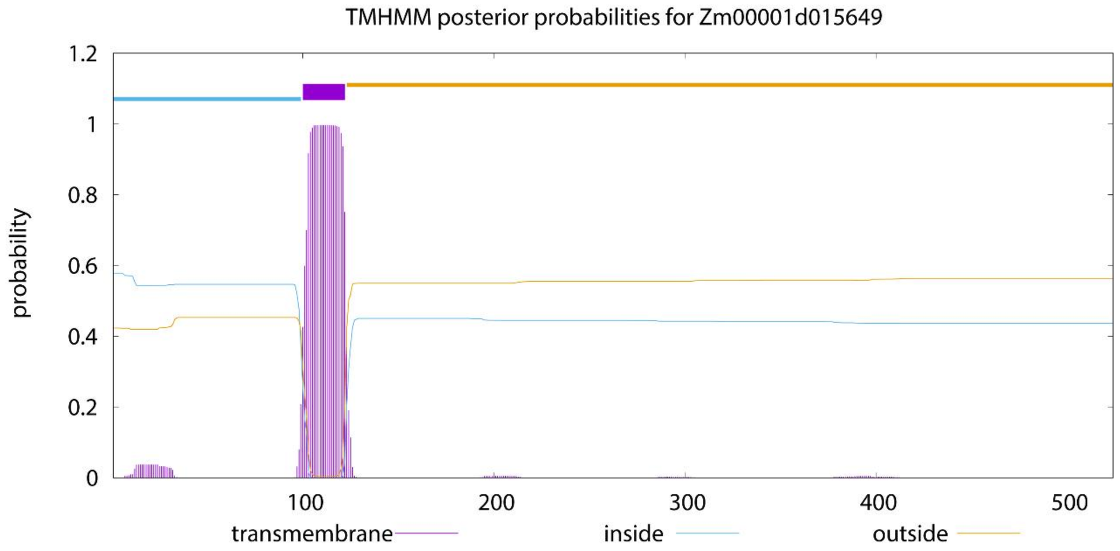
TMHMM posterior probabilities for Zm00001d015649
probability
1.2
1
0.8
0.6
0.4
0.2
0
100
200
300
400
500
transmembrane
inside
outside

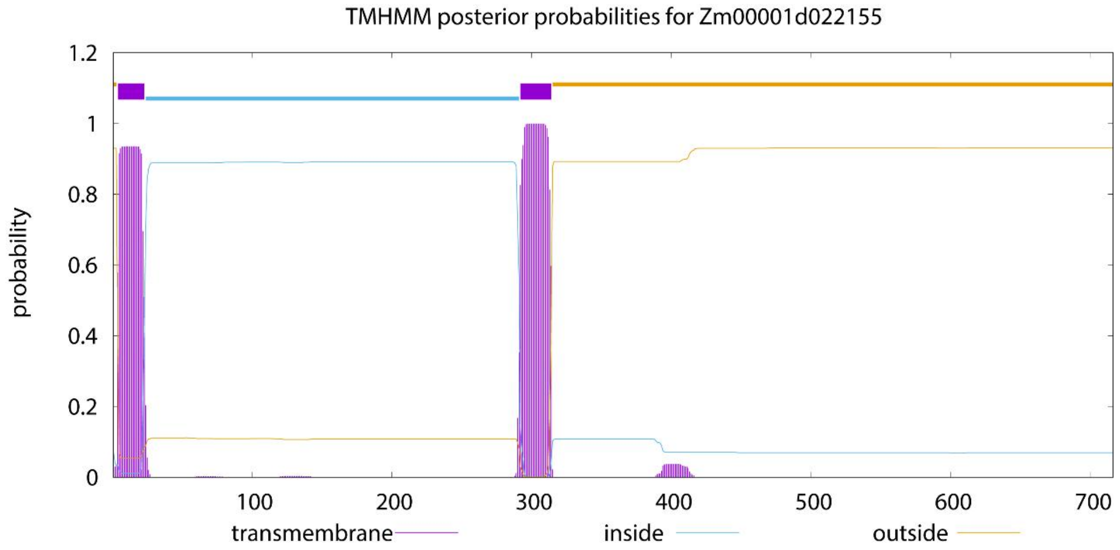
TMHMM posterior probabilities for Zm00001d022155
probability
1.2
1
0.8
0.6
0.4
0.2
0
100
200
300
400
500
600
700
transmembrane
inside
outside

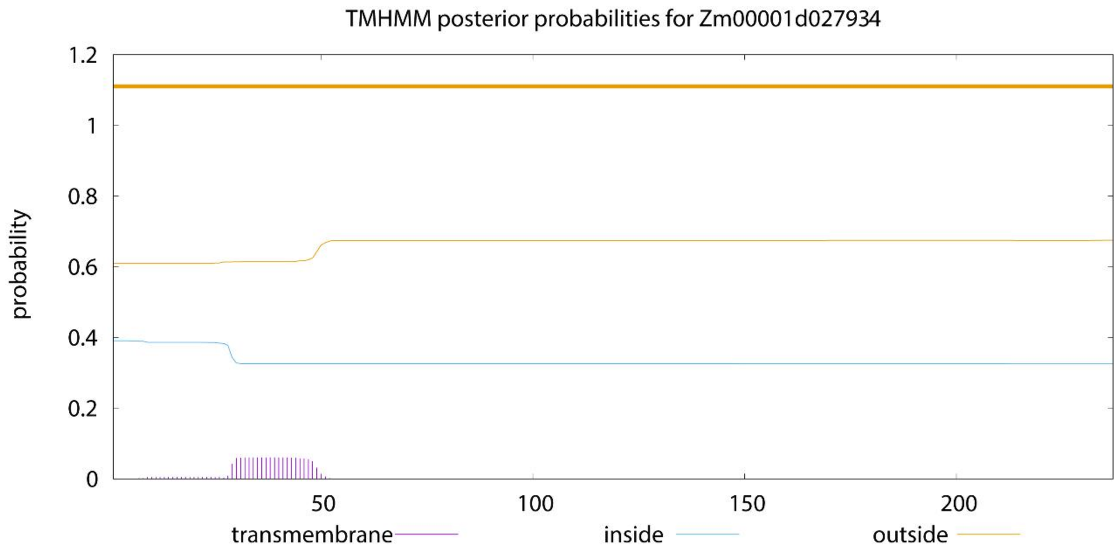
TMHMM posterior probabilities for Zm00001d027934
probability
1.2
1
0.8
0.6
0.4
0.2
0
50
100
150
200
transmembrane
inside
outside

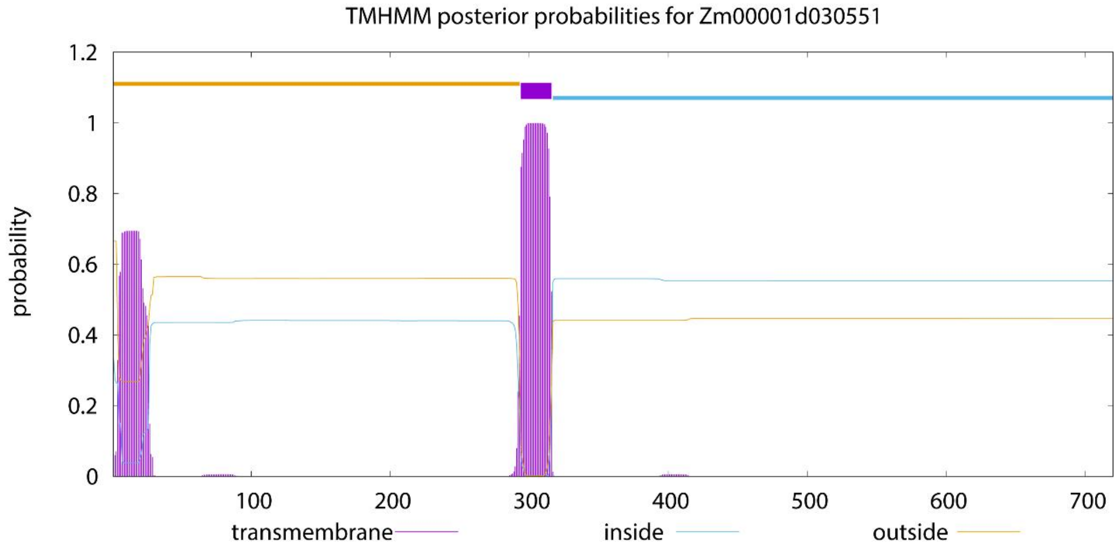
TMHMM posterior probabilities for Zm00001d030551
probability
1.2
1
0.8
0.6
0.4
0.2
0
100
200
300
400
500
600
700
transmembrane
inside
outside

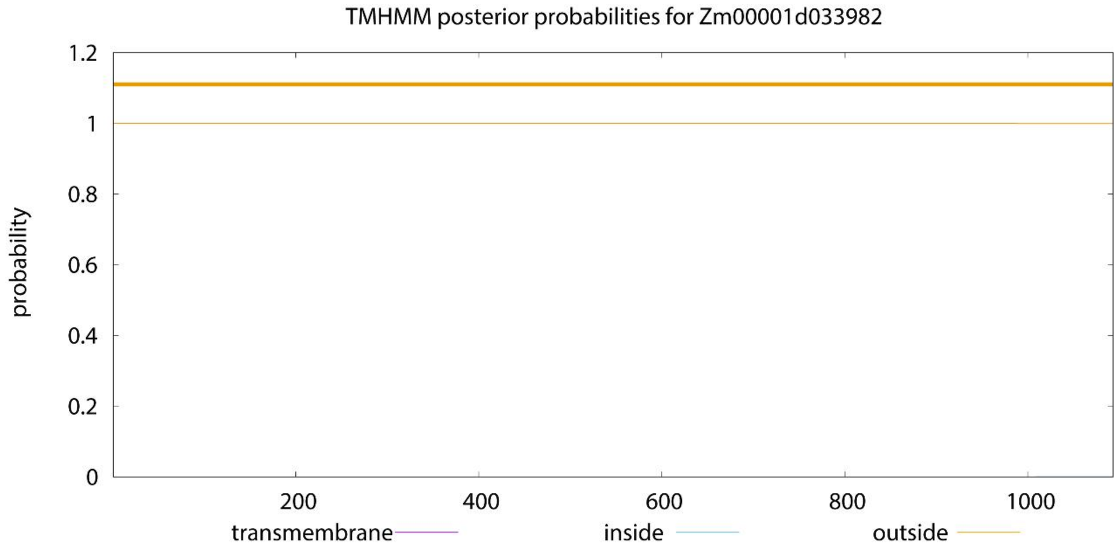
TMHMM posterior probabilities for Zm00001d033982
probability
1.2
1
0.8
0.6
0.4
0.2
0
200
400
600
800
1000
transmembrane
inside
outside

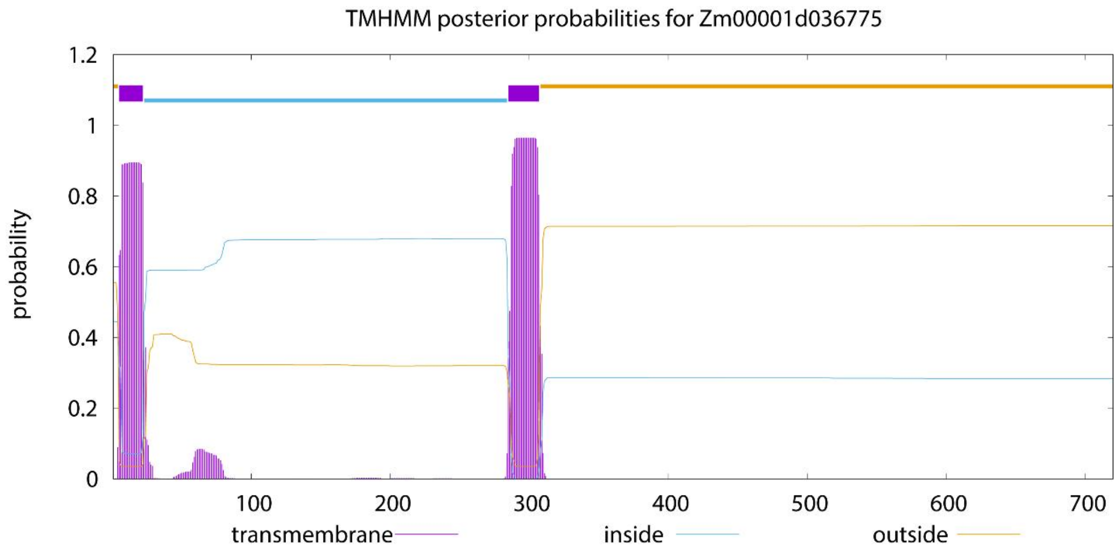
TMHMM posterior probabilities for Zm00001d036775
probability
1.2
1
0.8
0.6
0.4
0.2
0
100
200
300
400
500
600
700
transmembrane
inside
outside

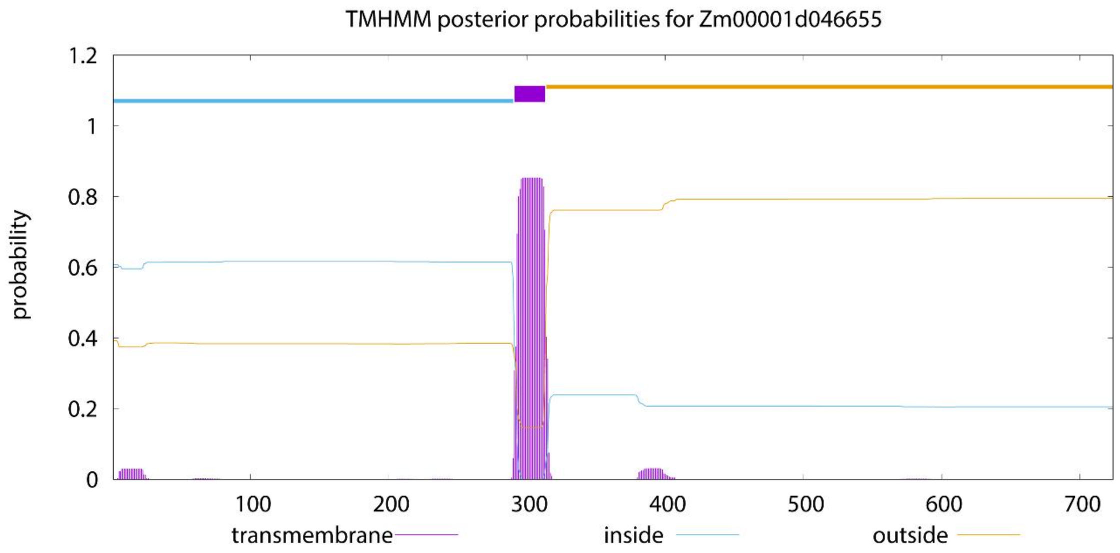
TMHMM posterior probabilities for Zm00001d046655
probability
1.2
1
0.8
0.6
0.4
0.2
0
100
200
300
400
500
600
700
transmembrane
inside
outside

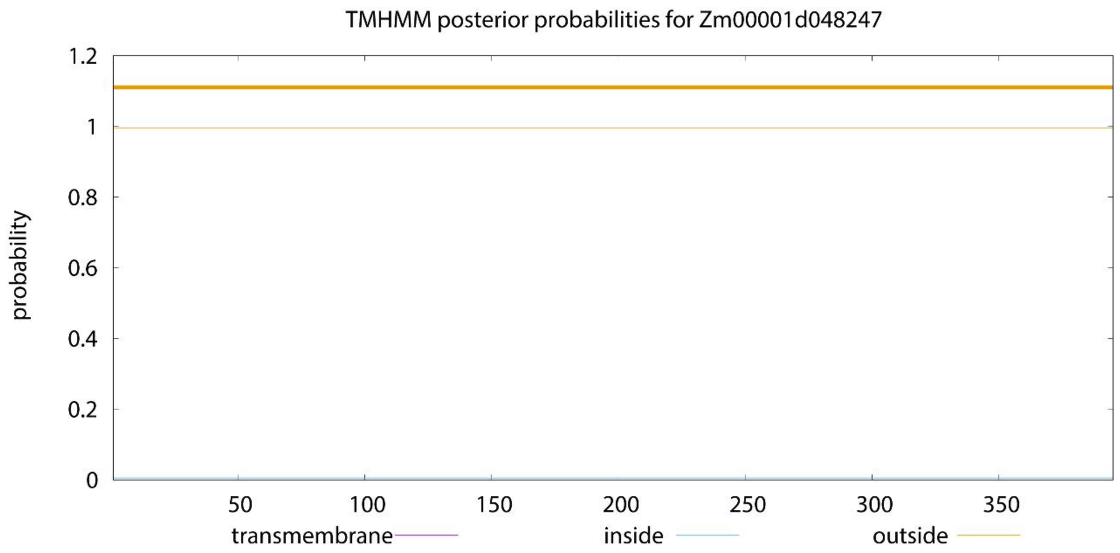
TMHMM posterior probabilities for Zm00001d048247
probability
1.2
1
0.8
0.6
0.4
0.2
0
50
100
150
200
250
300
350
transmembrane
inside
outside

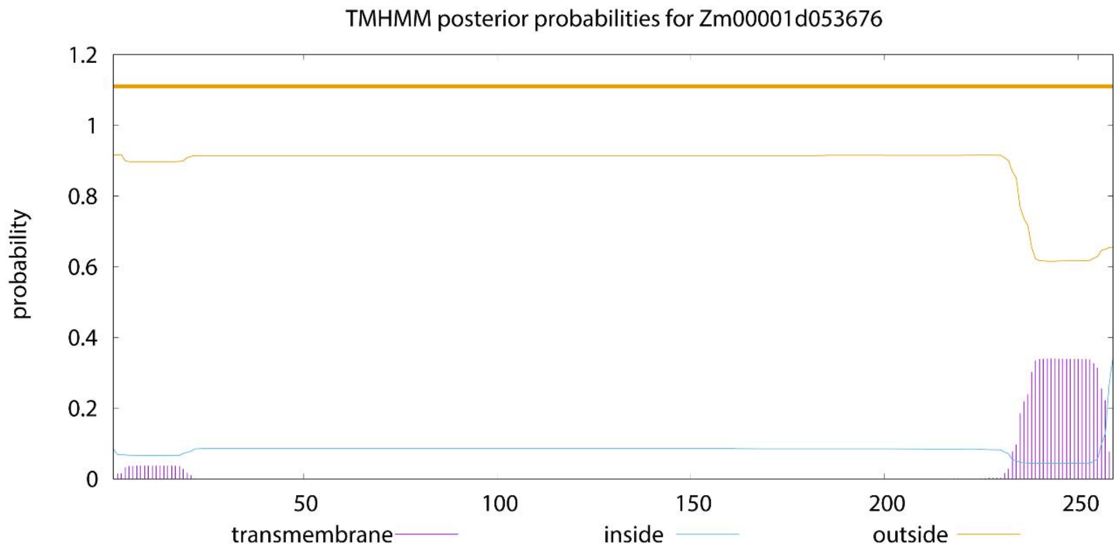

Transmembrane structure domain of subfamily V.

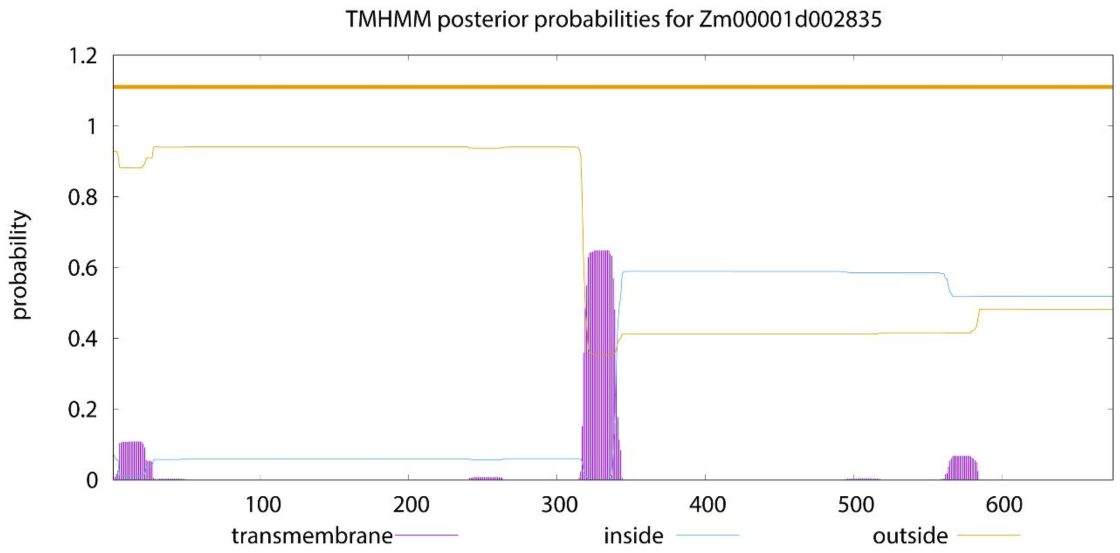

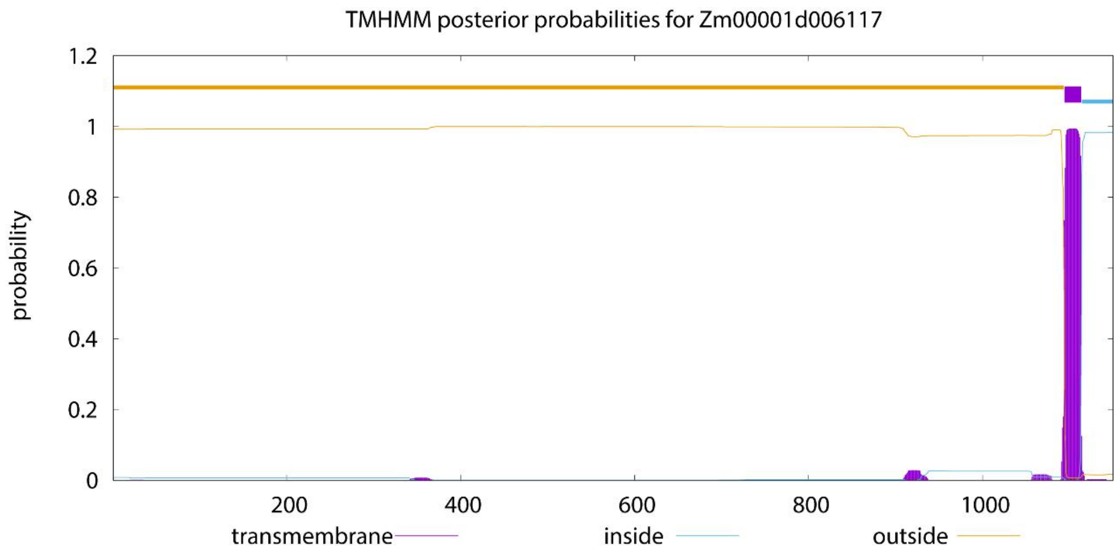

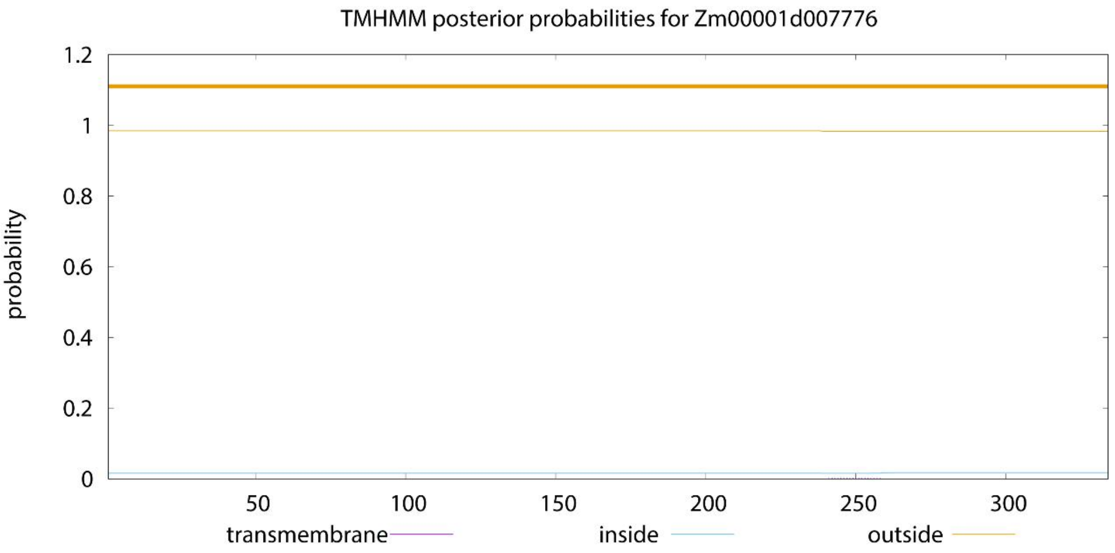
TMHMM posterior probabilities for Zm00001d007776
probability
1.2
1
0.8
0.6
0.4
0.2
0
50
100
150
200
250
300
transmembrane
inside
outside

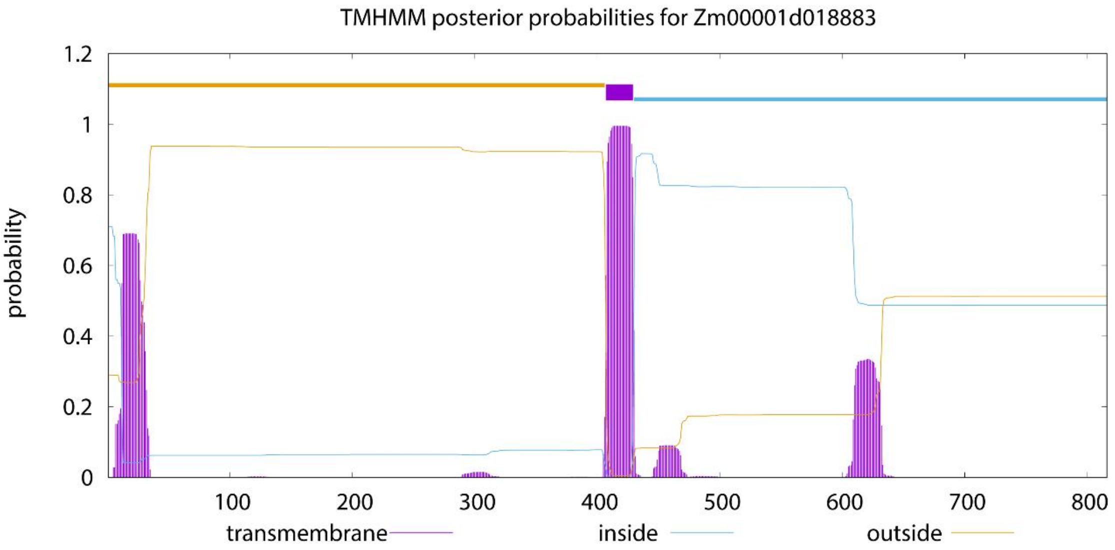
TMHMM posterior probabilities for Zm00001d018883
probability
1.2
1
0.8
0.6
0.4
0.2
0
100
200
300
400
500
600
700
800
transmembrane
inside
outside

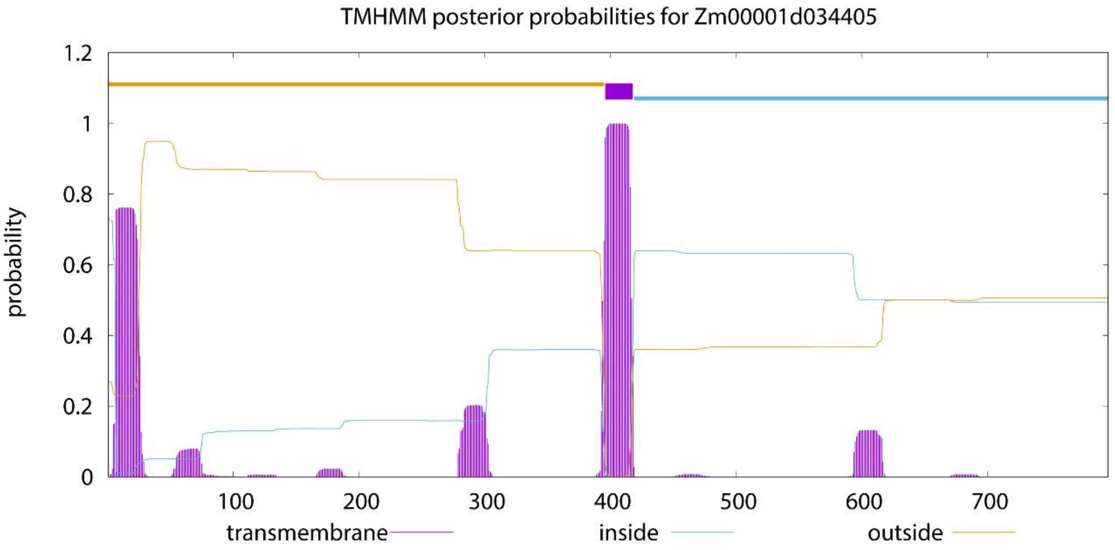
TMHMM posterior probabilities for Zm00001d034405
probability
1.2
1
0.8
0.6
0.4
0.2
0
100
200
300
400
500
600
700
transmembrane
inside
outside

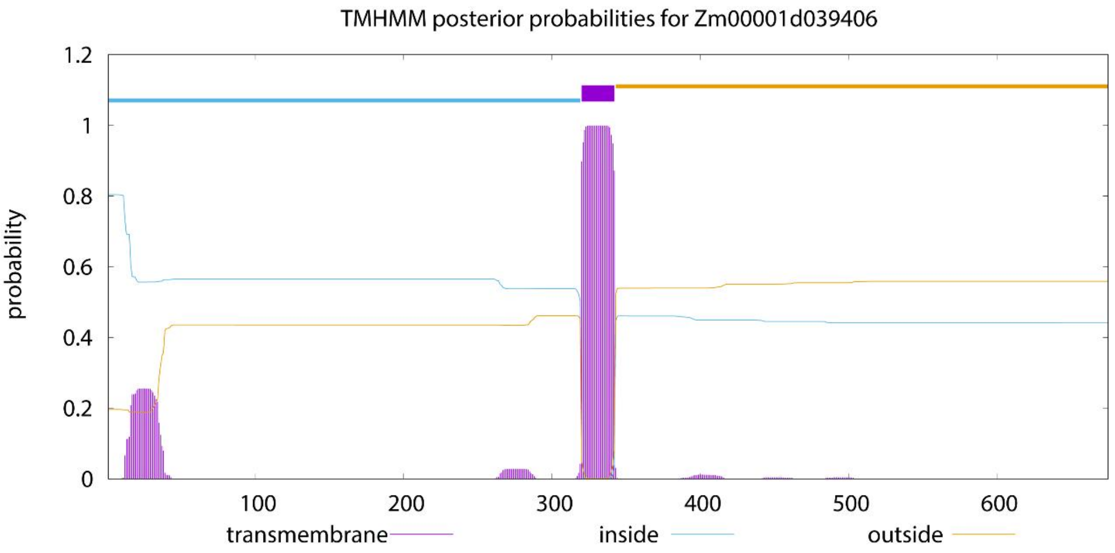

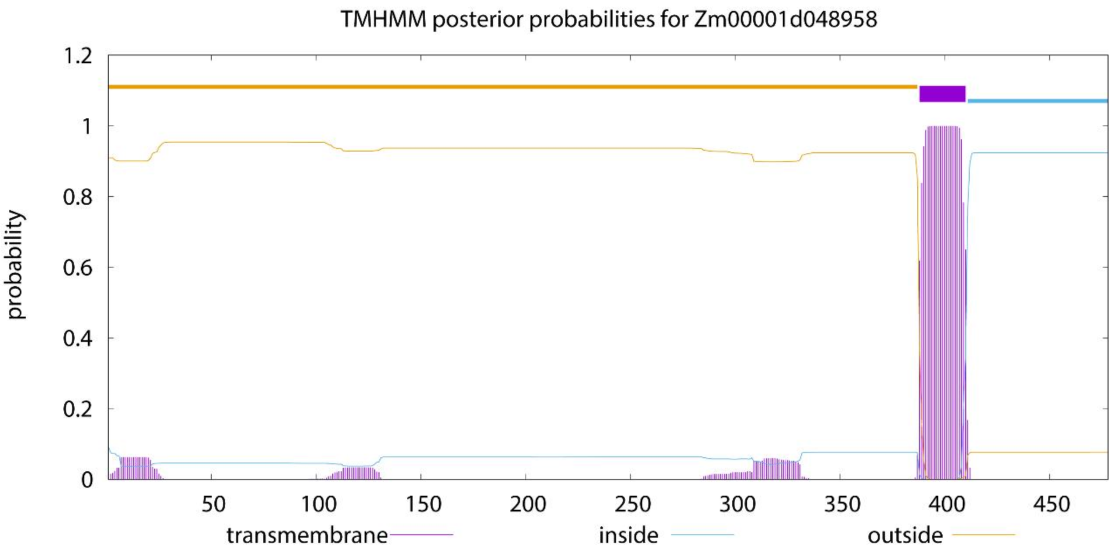

Transmembrane structure domain of subfamily VI.

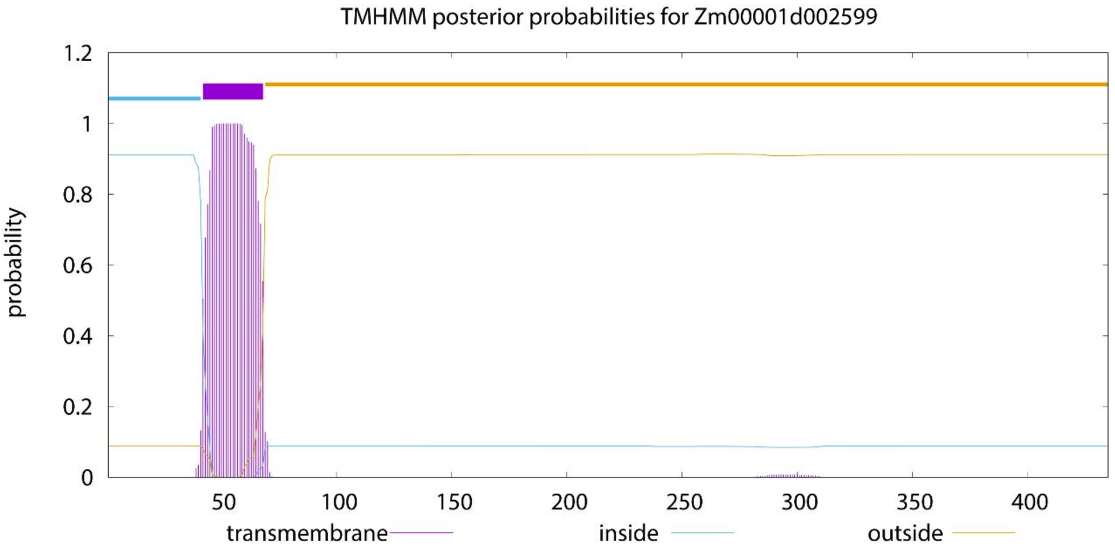

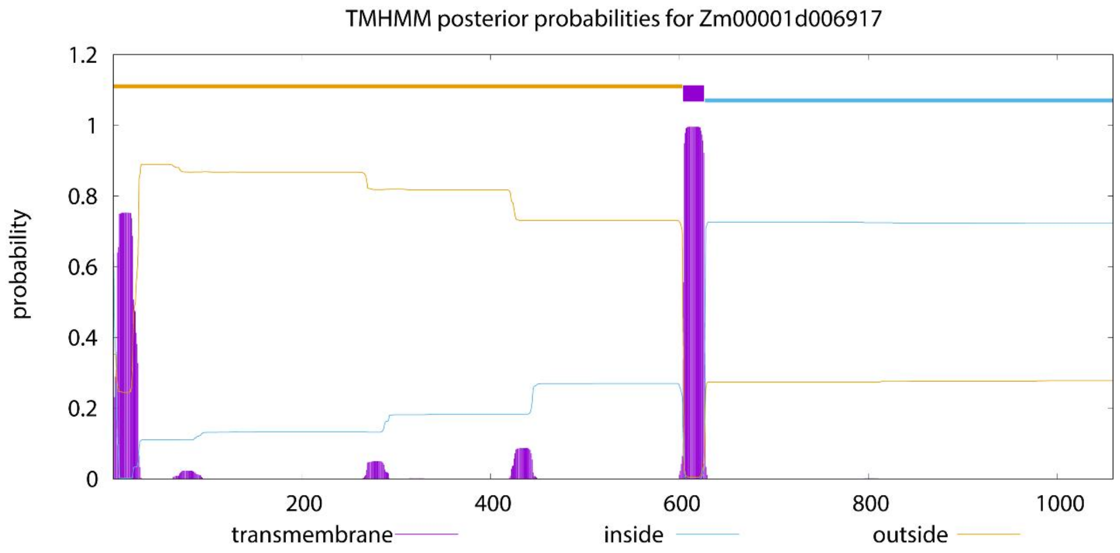
TMHMM posterior probabilities for Zm00001d006917
1.2
1
0.8
0.6
0.4
0.2
0
probability
200
400
600
800
1000
transmembrane
inside
outside

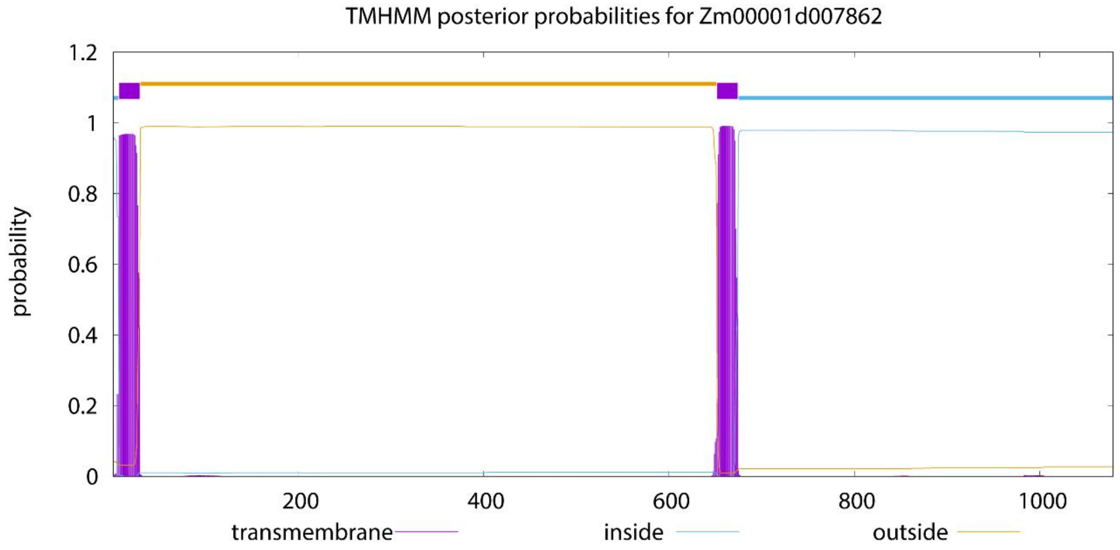
TMHMM posterior probabilities for Zm00001d007862
1.2
1
0.8
0.6
0.4
0.2
0
probability
200
400
600
800
1000
transmembrane
inside
outside

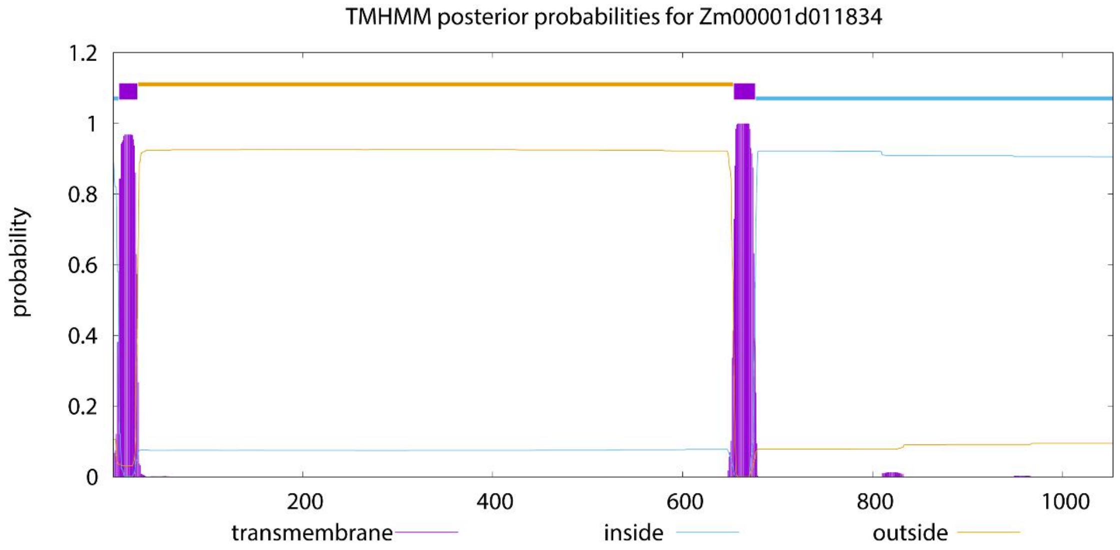
TMHMM posterior probabilities for Zm00001d011834
1.2
1
0.8
0.6
0.4
0.2
0
probability
200
400
600
800
1000
transmembrane
inside
outside

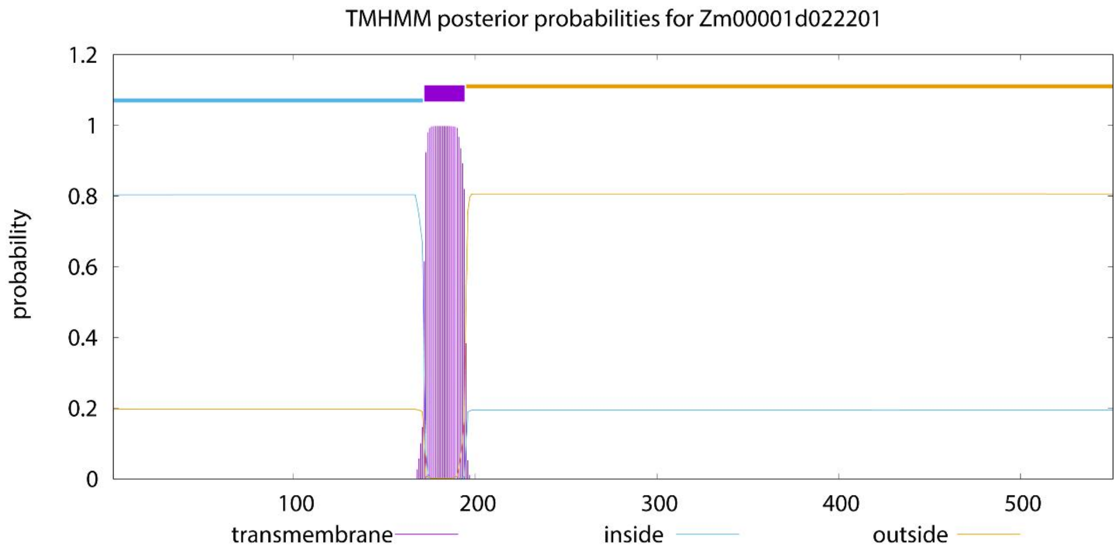
TMHMM posterior probabilities for Zm00001d022201
probability
1.2
1
0.8
0.6
0.4
0.2
0
100
200
300
400
500
transmembrane
inside
outside

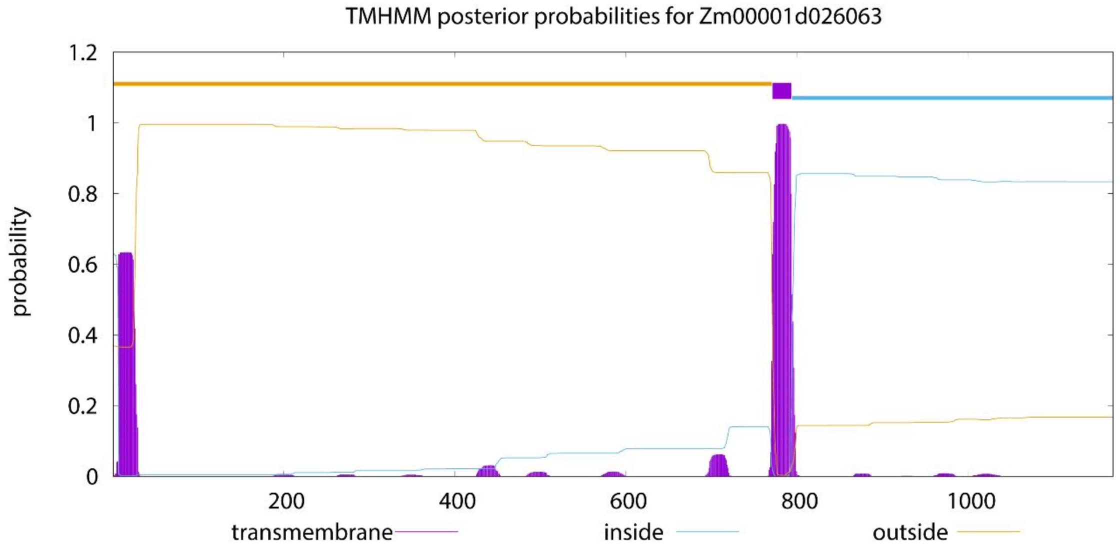
TMHMM posterior probabilities for Zm00001d026063
probability
1.2
1
0.8
0.6
0.4
0.2
0
200
400
600
800
1000
transmembrane
inside
outside

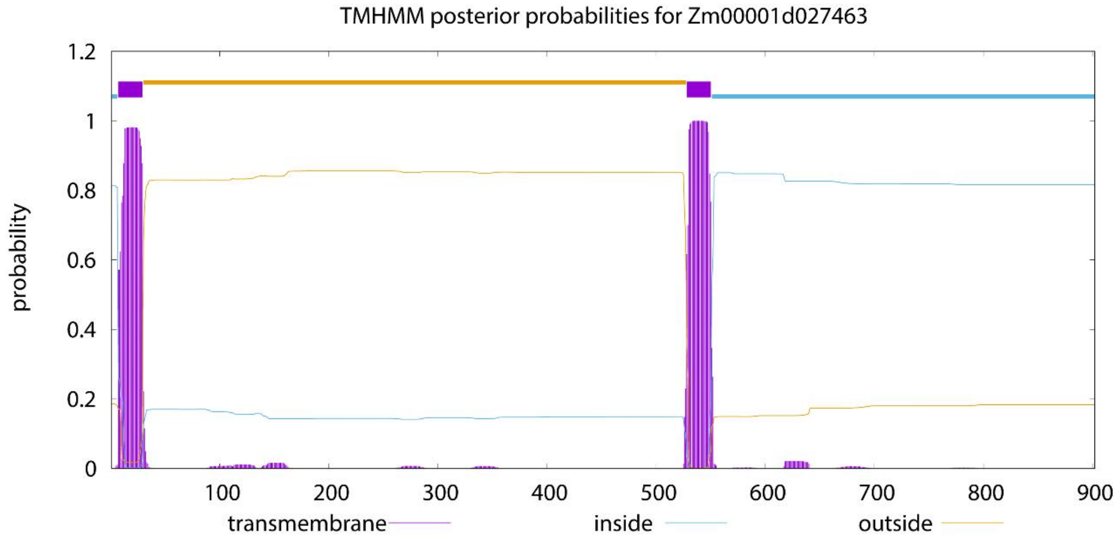
TMHMM posterior probabilities for Zm00001d027463
probability
1.2
1
0.8
0.6
0.4
0.2
0
100
200
300
400
500
600
700
800
900
transmembrane
inside
outside

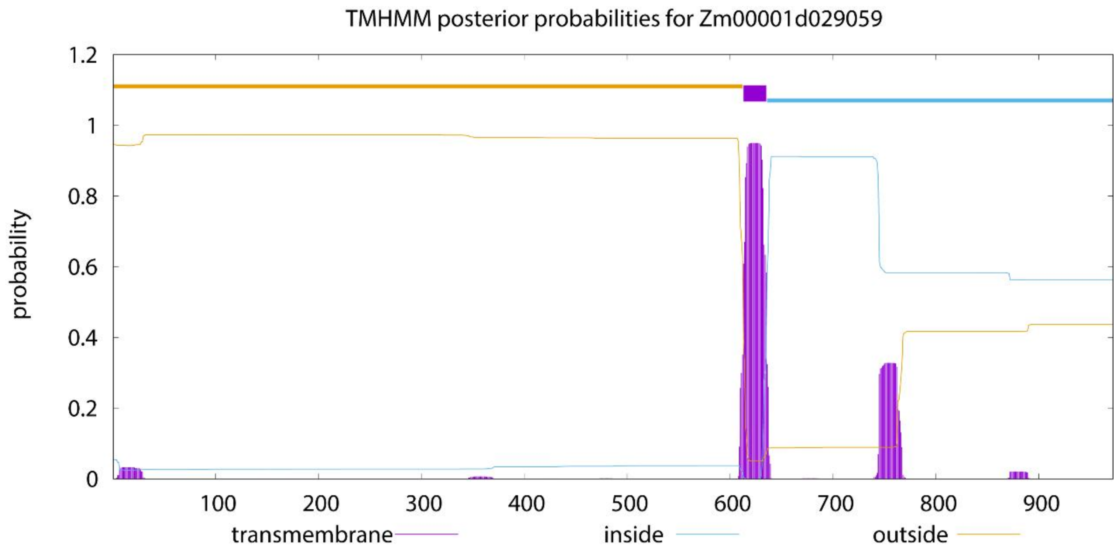
TMHMM posterior probabilities for Zm00001d029059
1.2
1
0.8
0.6
0.4
0.2
0
probability
100
200
300
400
500
600
700
800
900
transmembrane
inside
outside

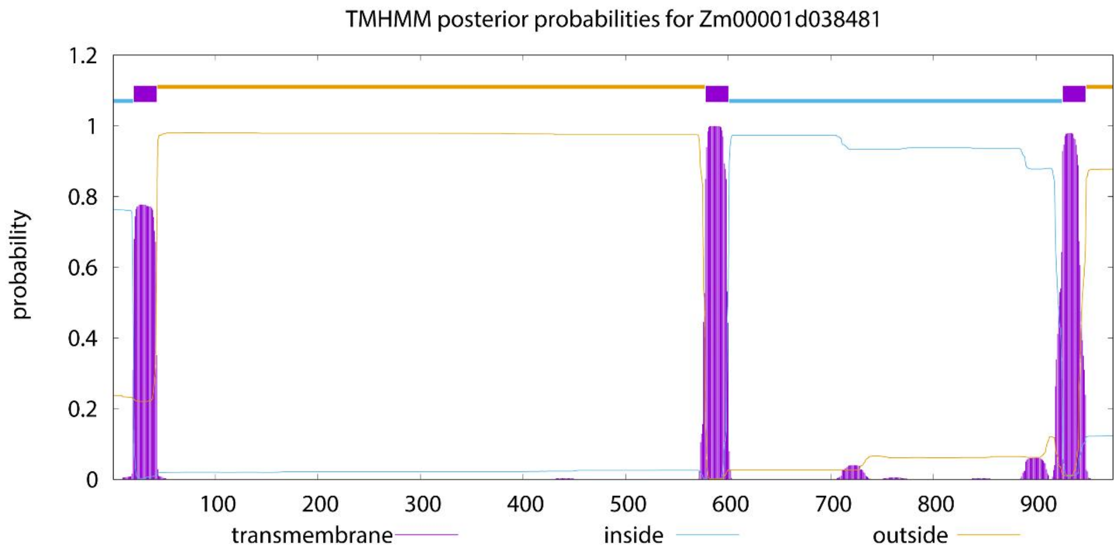
TMHMM posterior probabilities for Zm00001d038481
1.2
1
0.8
0.6
0.4
0.2
0
probability
100
200
300
400
500
600
700
800
900
transmembrane
inside
outside

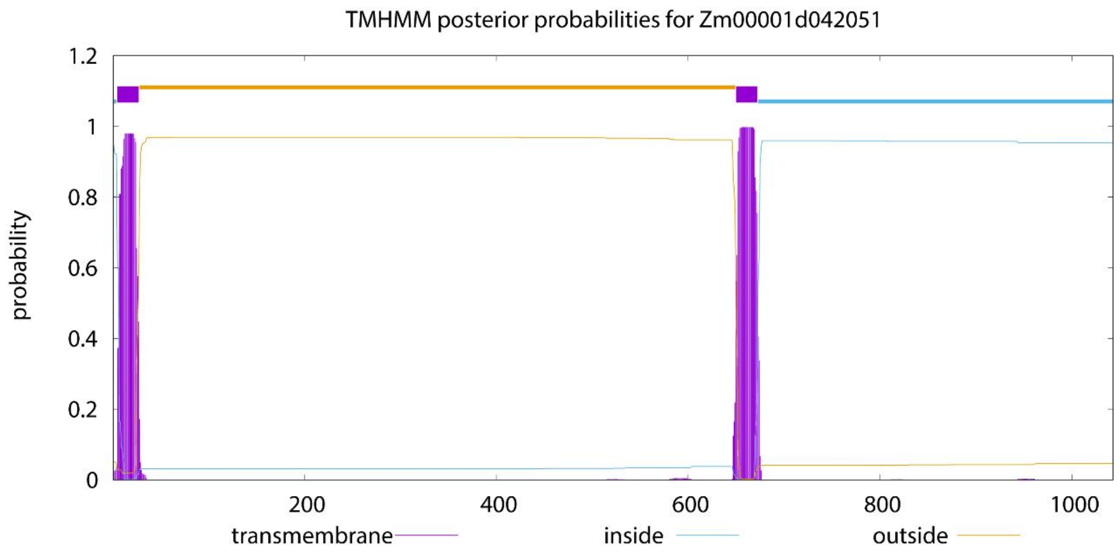
TMHMM posterior probabilities for Zm00001d042051
1.2
1
0.8
0.6
0.4
0.2
0
probability
200
400
600
800
1000
transmembrane
inside
outside

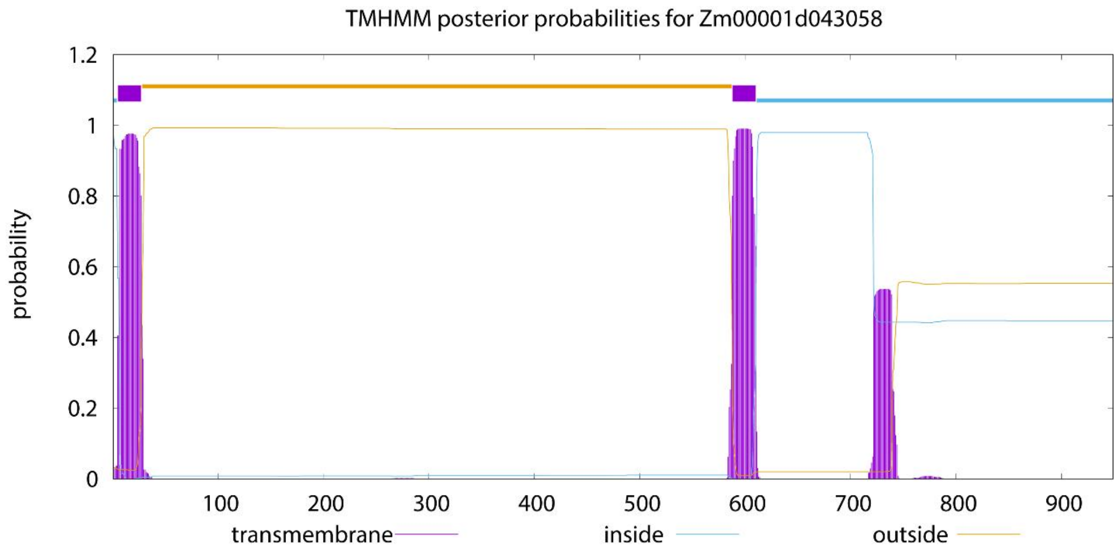
TMHMM posterior probabilities for Zm00001d043058
probability
1.2
1
0.8
0.6
0.4
0.2
0
100
200
300
400
500
600
700
800
900
transmembrane
inside
outside

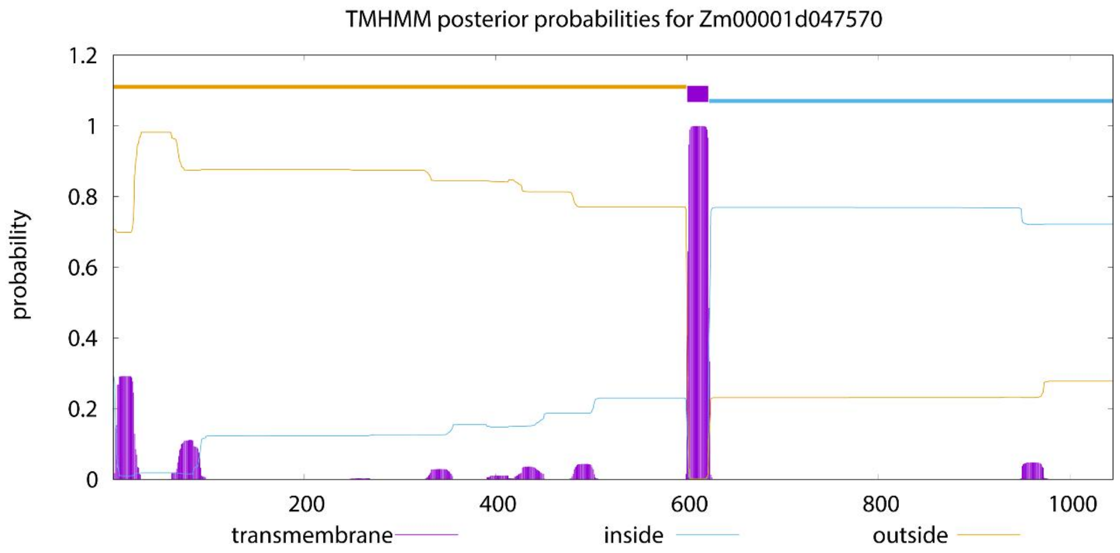
TMHMM posterior probabilities for Zm00001d047570
probability
1.2
1
0.8
0.6
0.4
0.2
0
200
400
600
800
1000
transmembrane
inside
outside

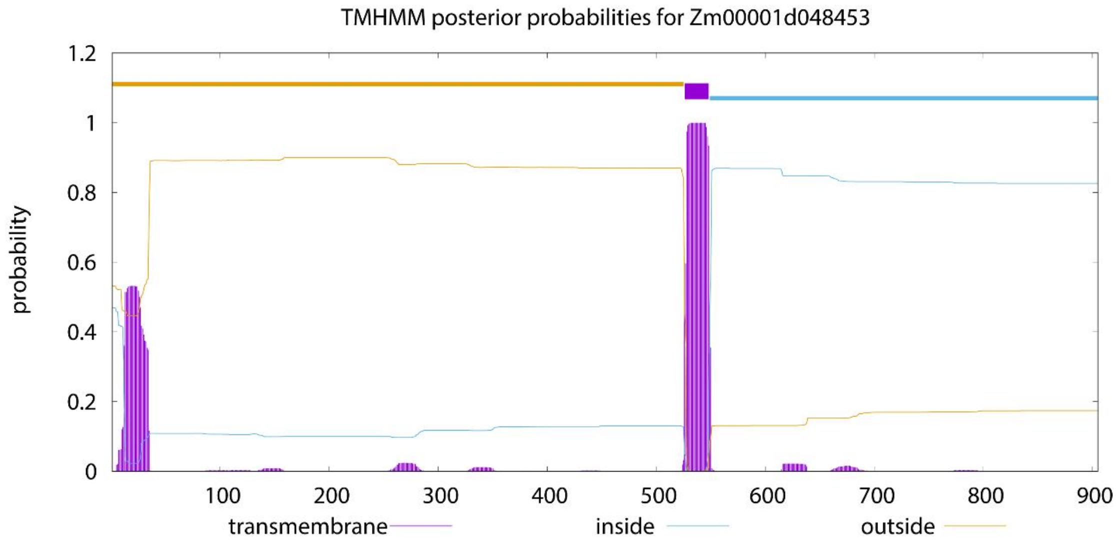
TMHMM posterior probabilities for Zm00001d048453
probability
1.2
1
0.8
0.6
0.4
0.2
0
100
200
300
400
500
600
700
800
900
transmembrane
inside
outside

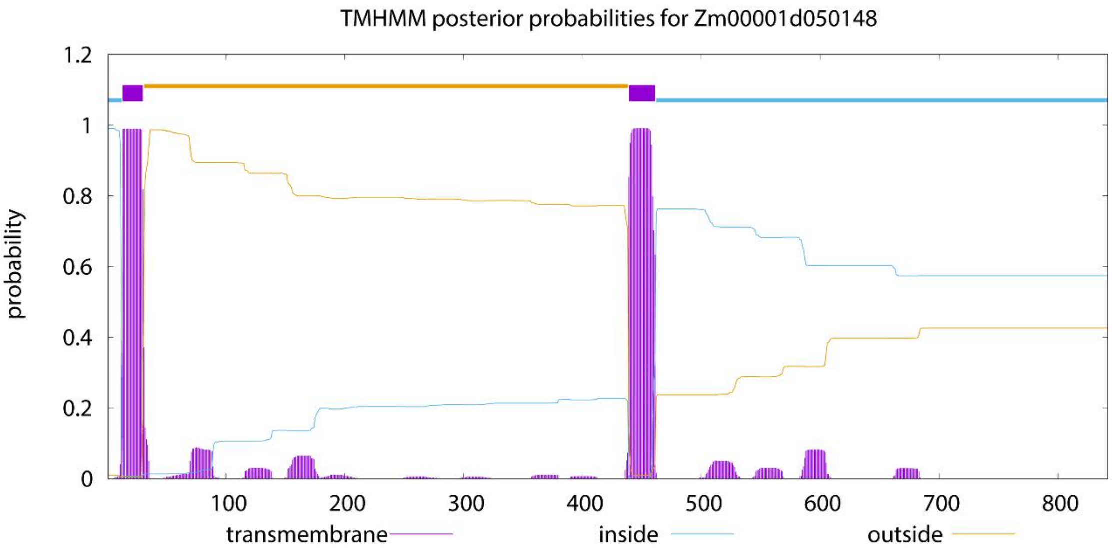

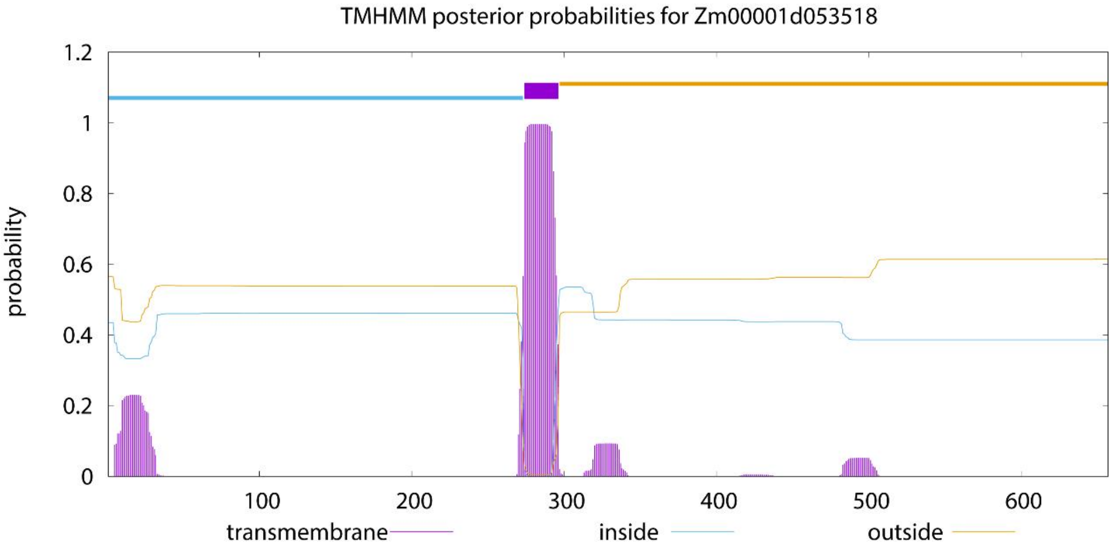

Transmembrane structure domain of subfamily VII.

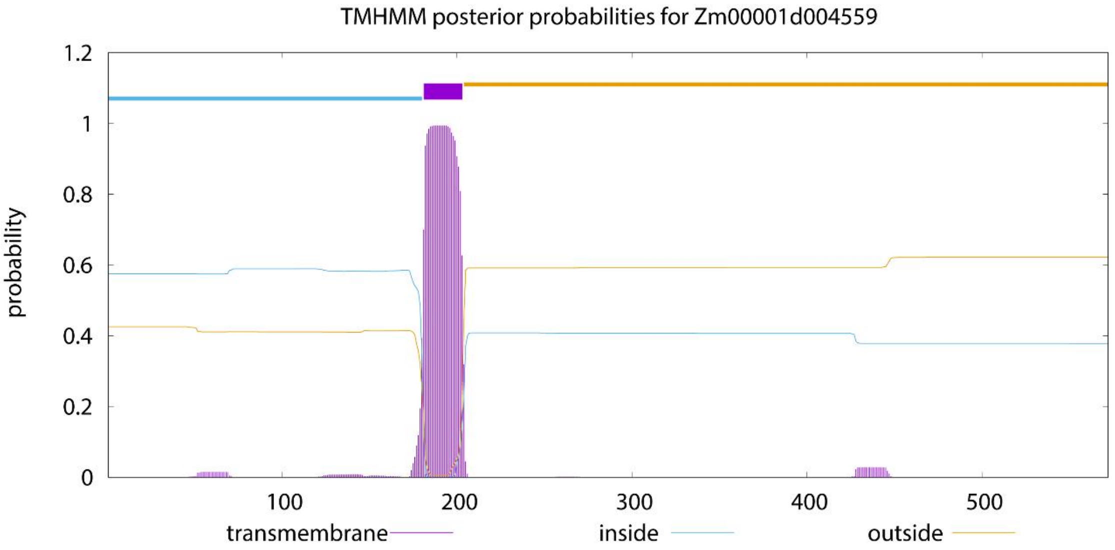

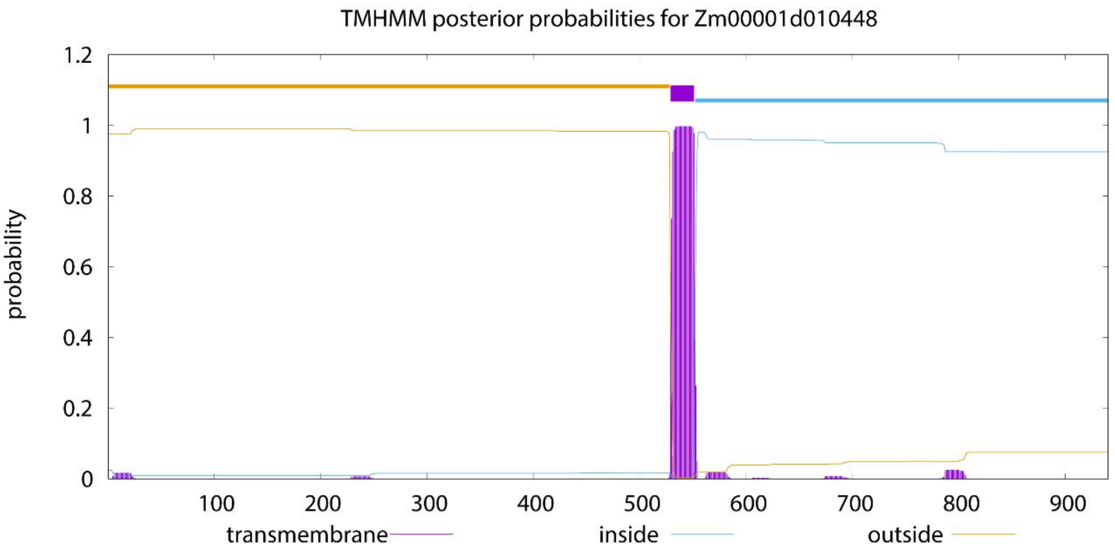
TMHMM posterior probabilities for Zm00001d010448
probability
1.2
1
0.8
0.6
0.4
0.2
0
100
200
300
400
500
600
700
800
900
transmembrane
inside
outside

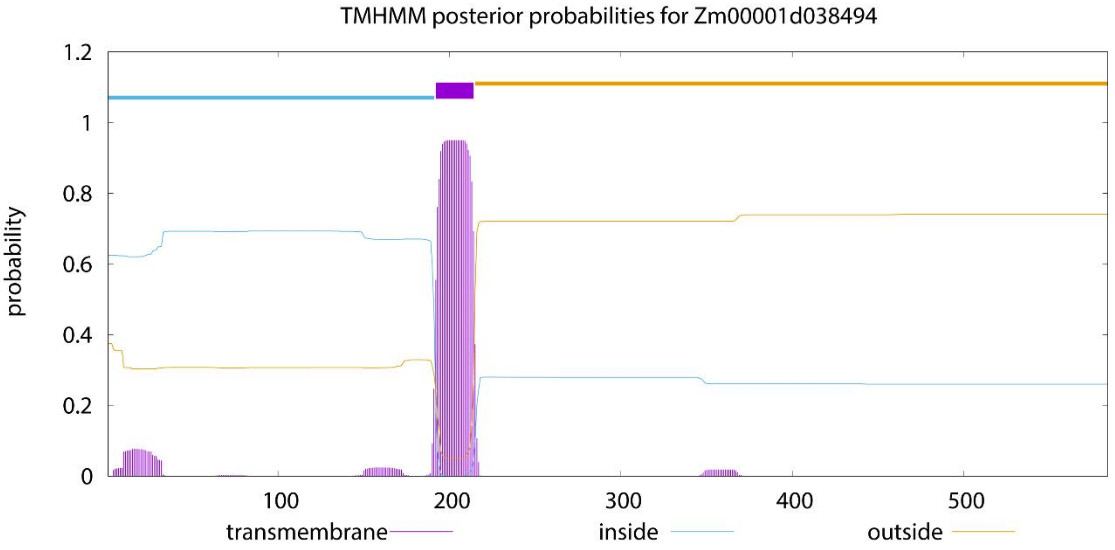
TMHMM posterior probabilities for Zm00001d038494
probability
1.2
1
0.8
0.6
0.4
0.2
0
100
200
300
400
500
transmembrane
inside
outside

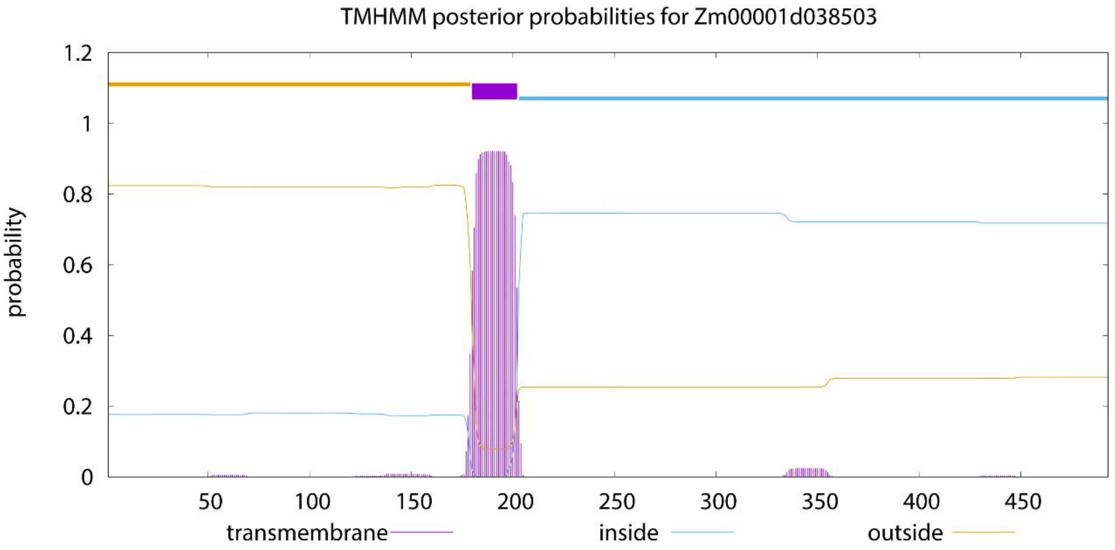
TMHMM posterior probabilities for Zm00001d038503
probability
1.2
1
0.8
0.6
0.4
0.2
0
50
100
150
200
250
300
350
400
450
transmembrane
inside
outside

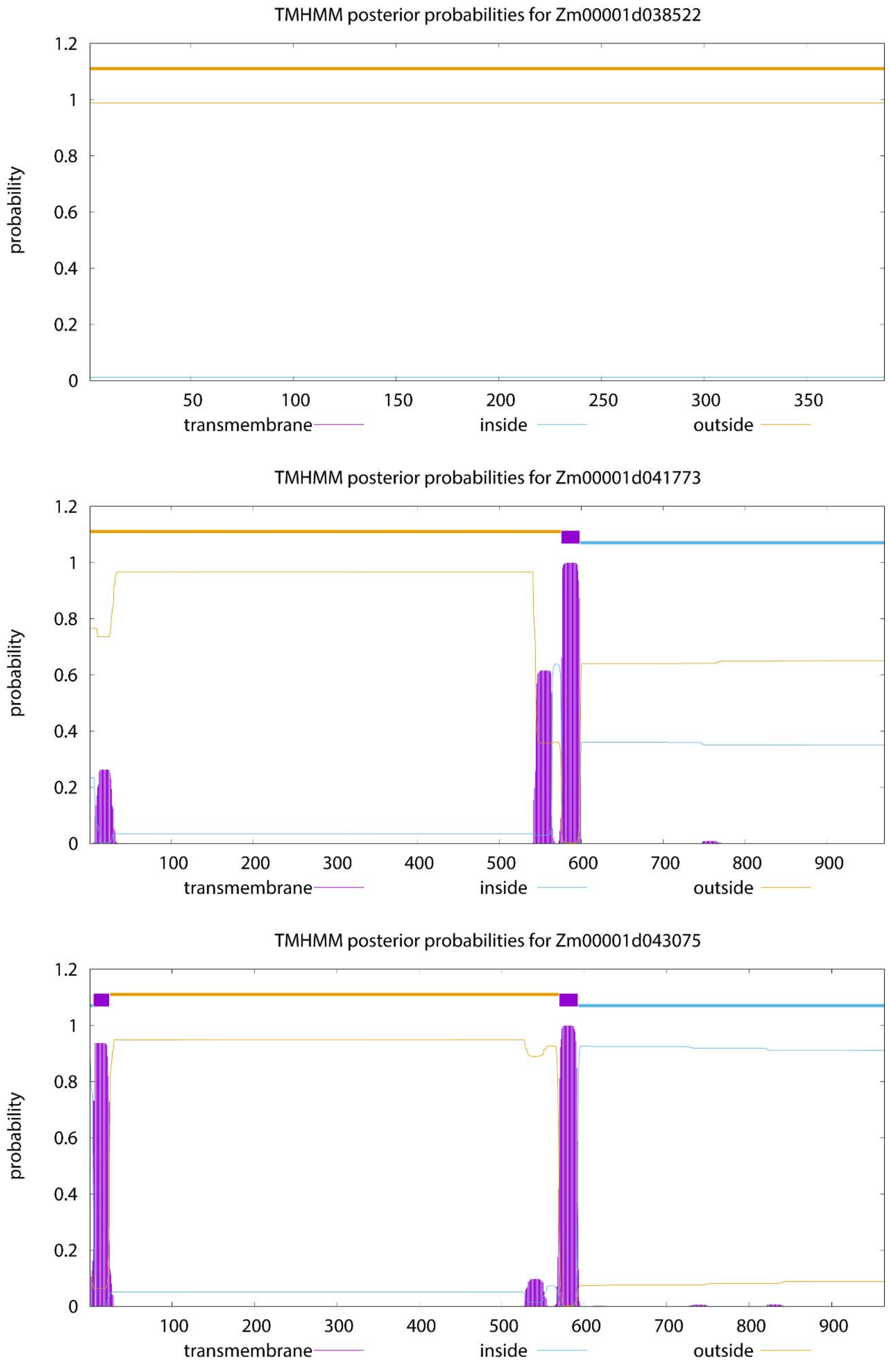

Transmembrane structure domain of subfamily VIII.

TMHMM posterior probabilities for Zm00001d007313

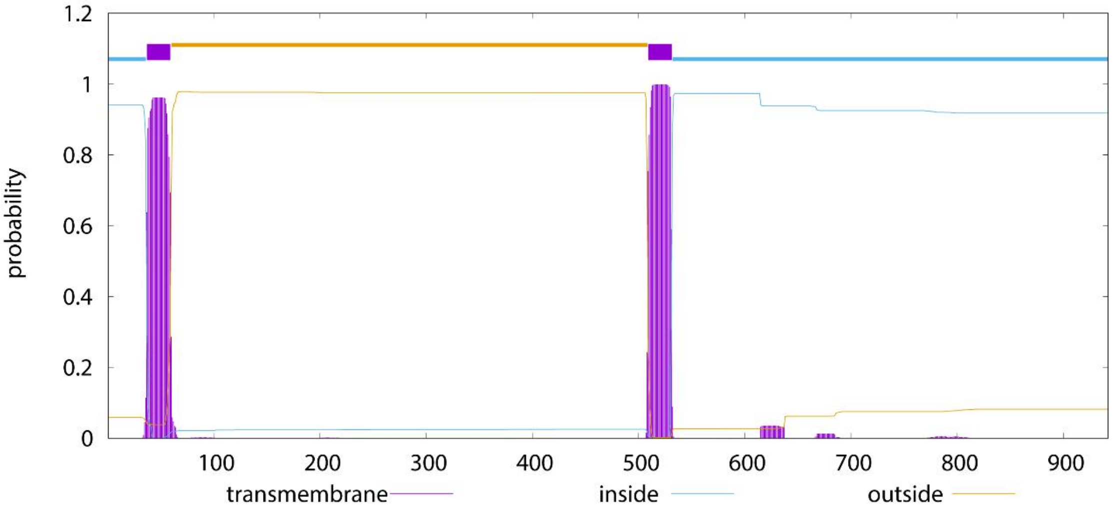

TMHMM posterior probabilities for Zm00001d013415

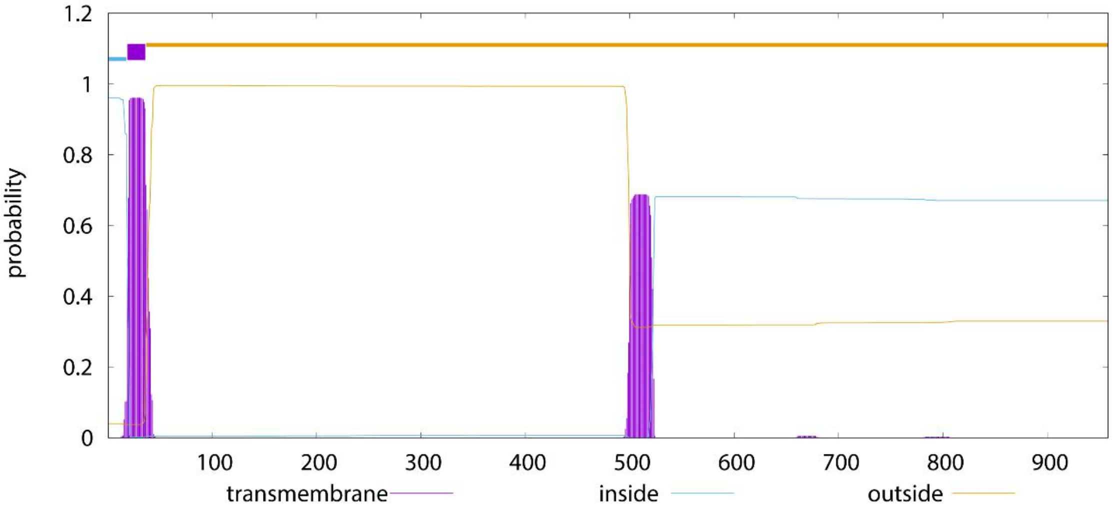

TMHMM posterior probabilities for Zm00001d026668

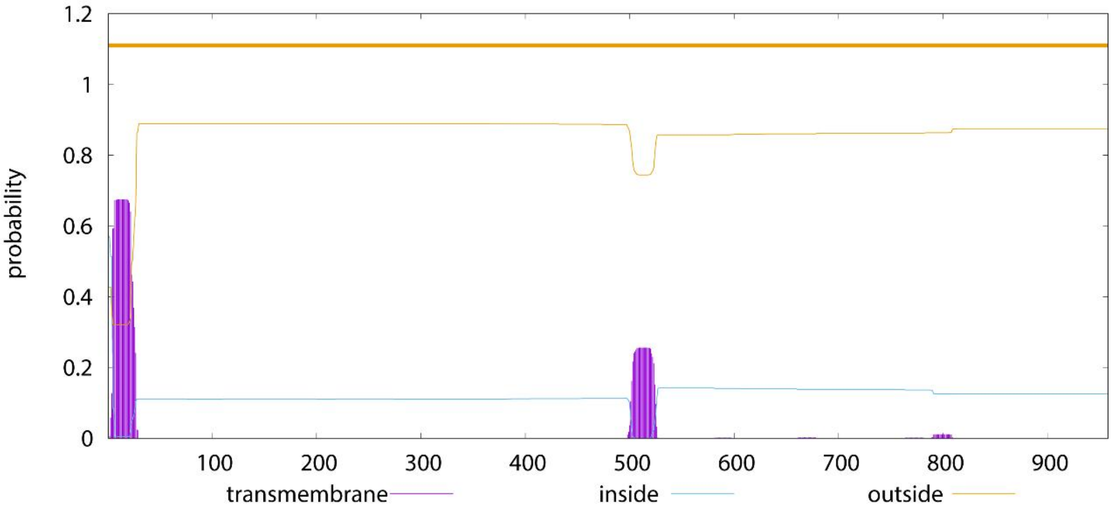

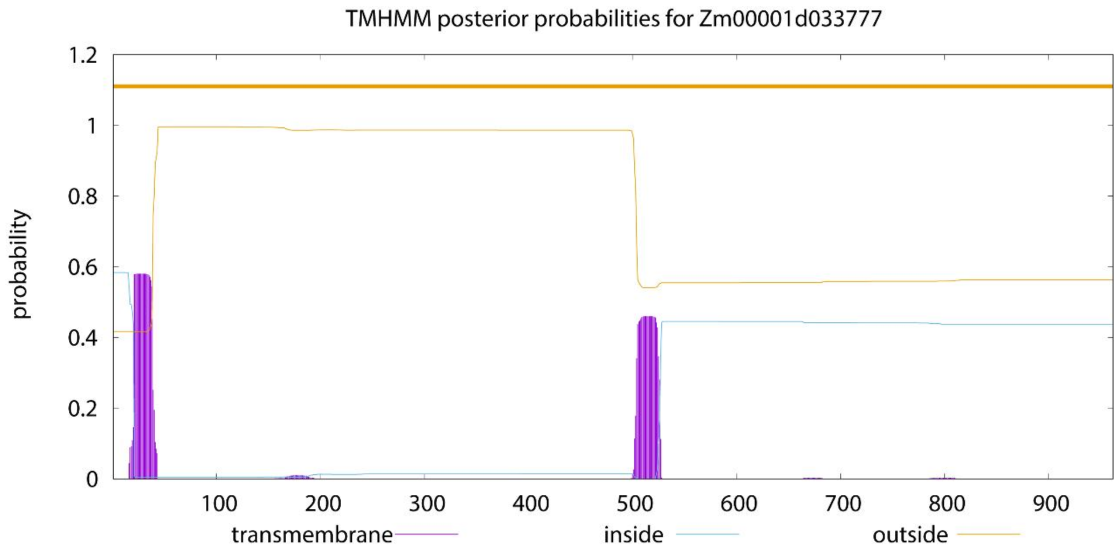

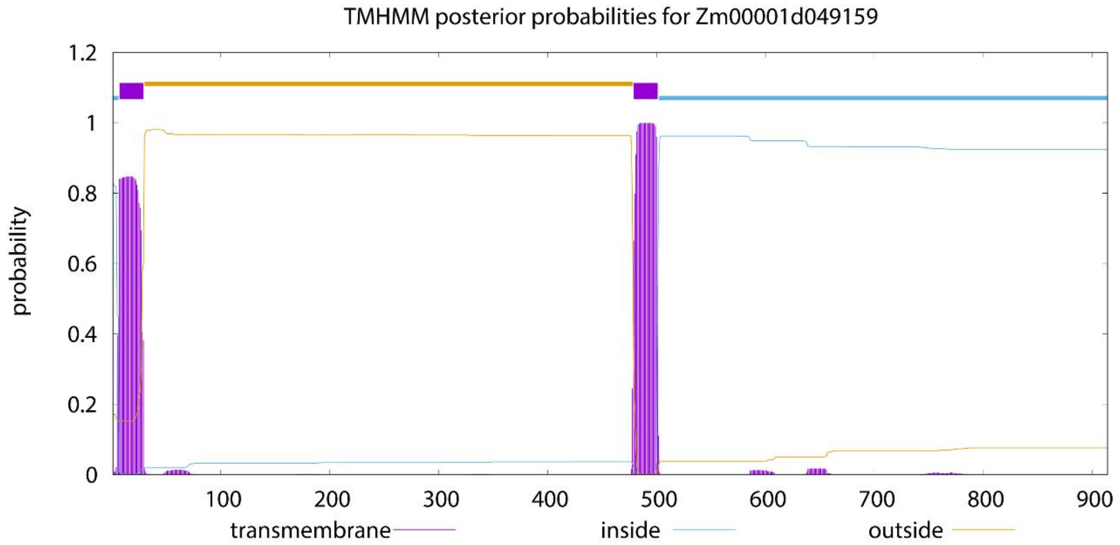

Transmembrane structure domain of subfamily IX.

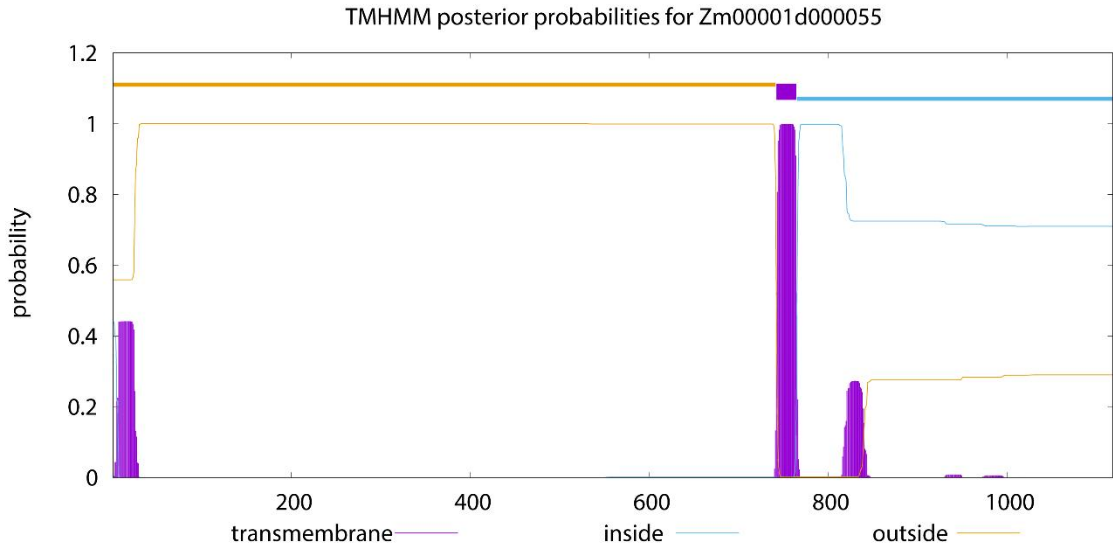

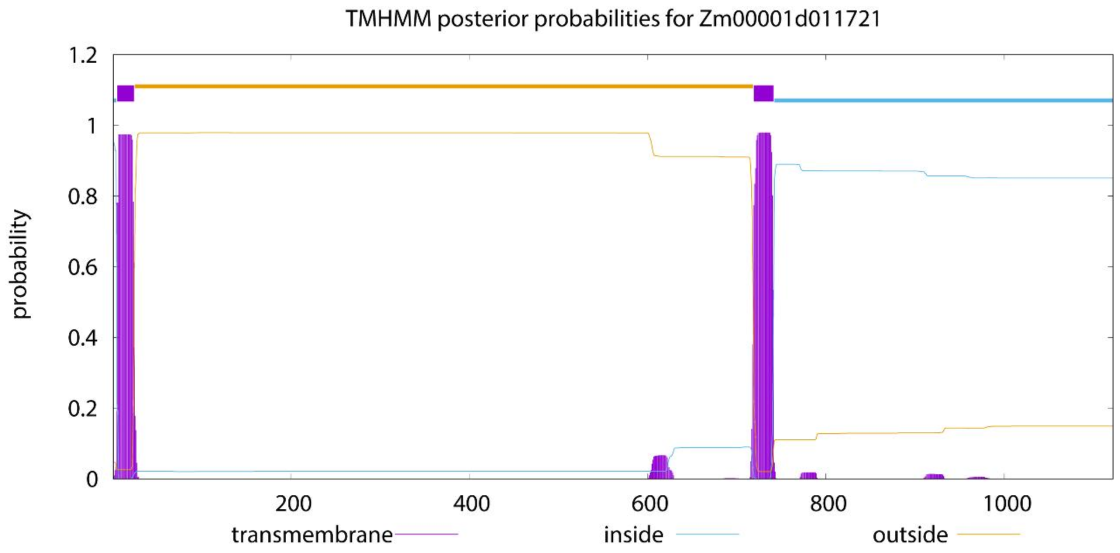
TMHMM posterior probabilities for Zm00001d011721
probability
1.2
1
0.8
0.6
0.4
0.2
0
200
400
600
800
1000
transmembrane
inside
outside

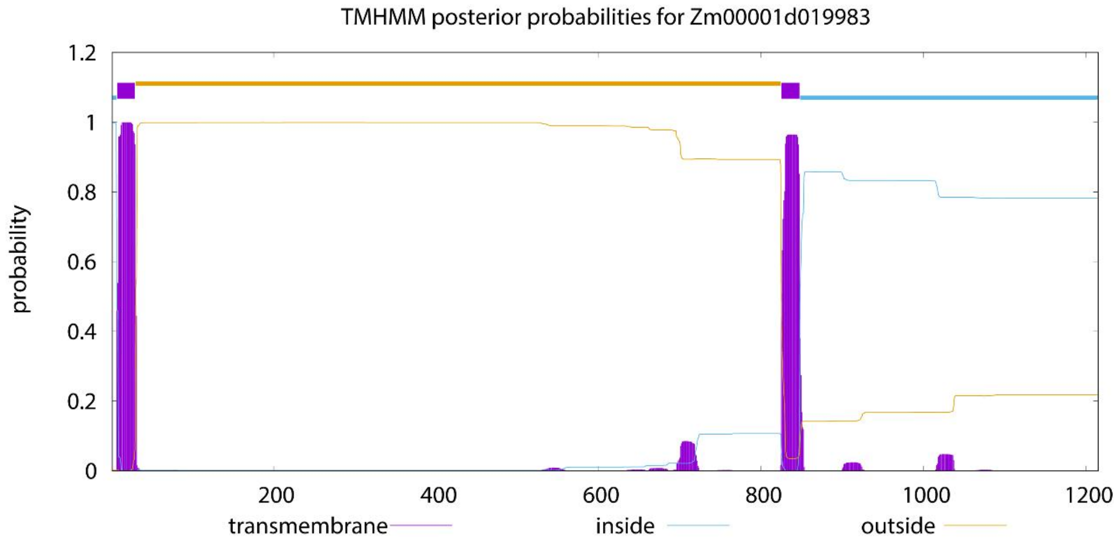
TMHMM posterior probabilities for Zm00001d019983
probability
1.2
1
0.8
0.6
0.4
0.2
0
200
400
600
800
1000
1200
transmembrane
inside
outside

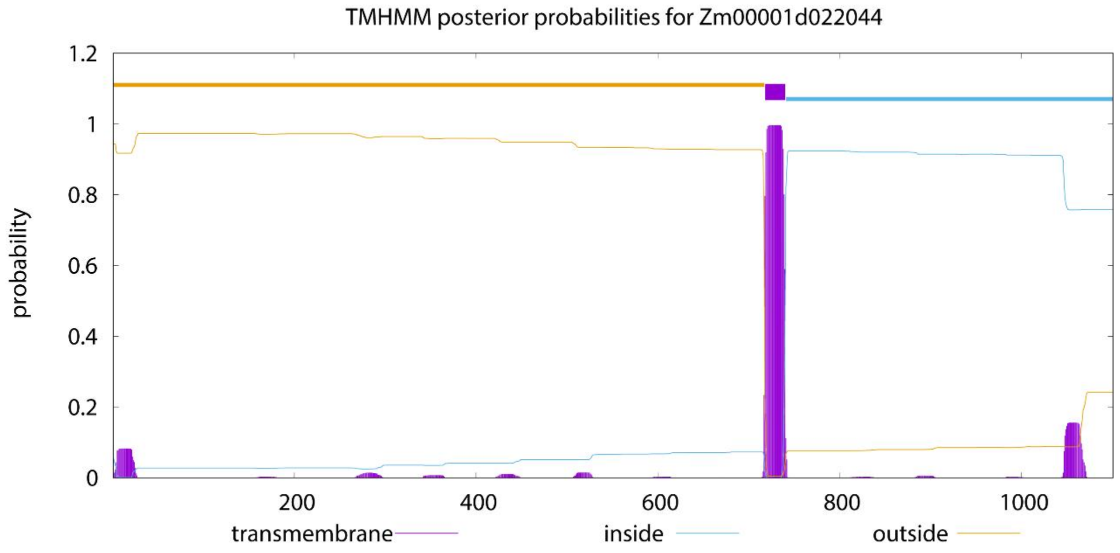
TMHMM posterior probabilities for Zm00001d022044
probability
1.2
1
0.8
0.6
0.4
0.2
0
200
400
600
800
1000
transmembrane
inside
outside

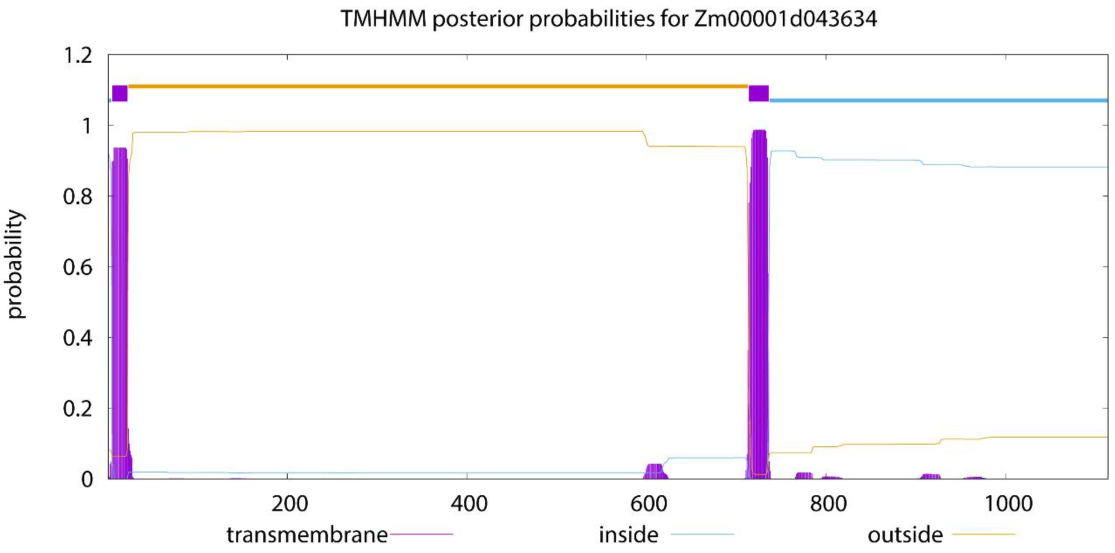

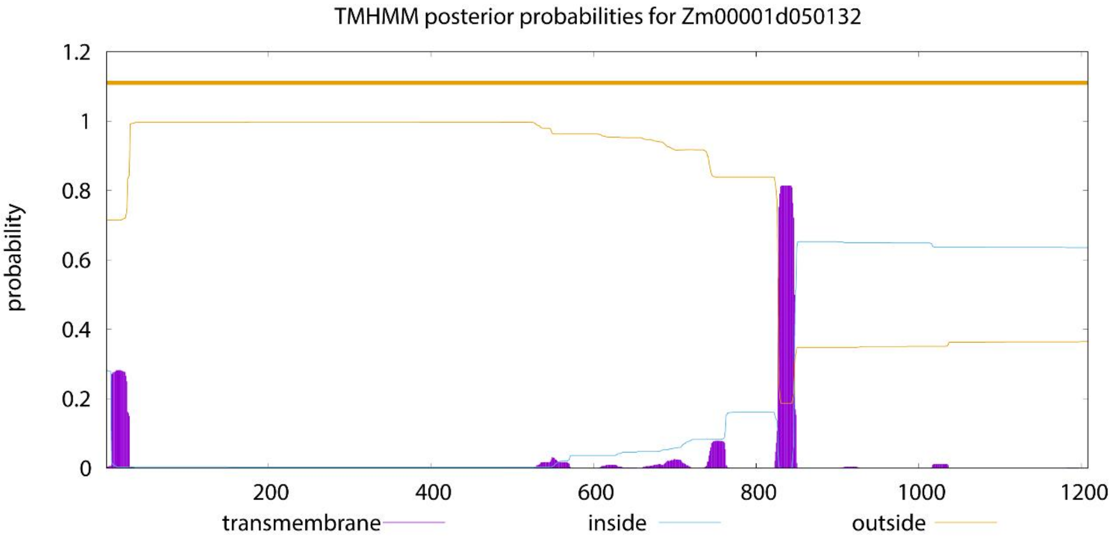

Transmembrane structure domain of subfamily X.

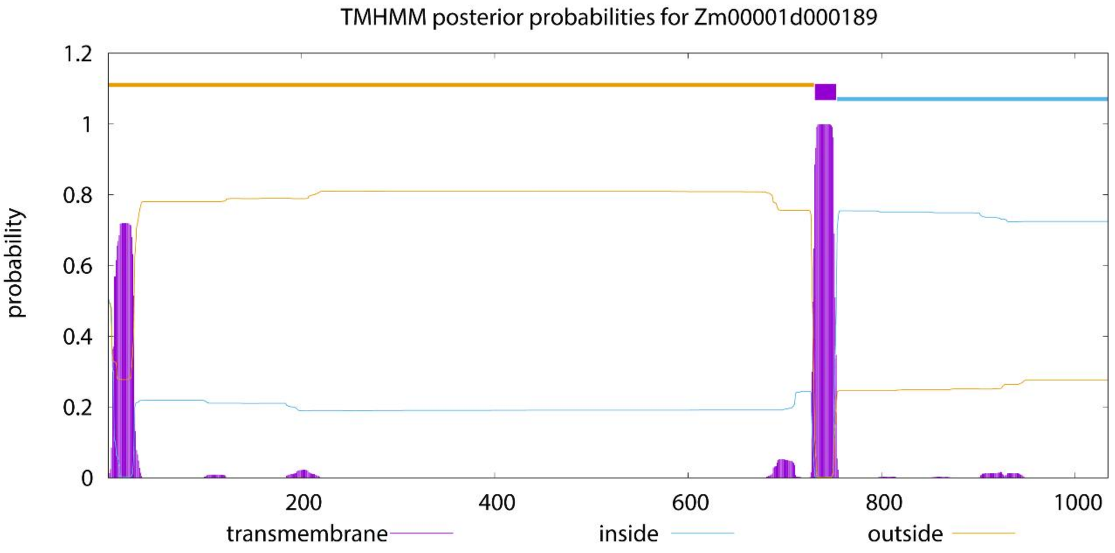

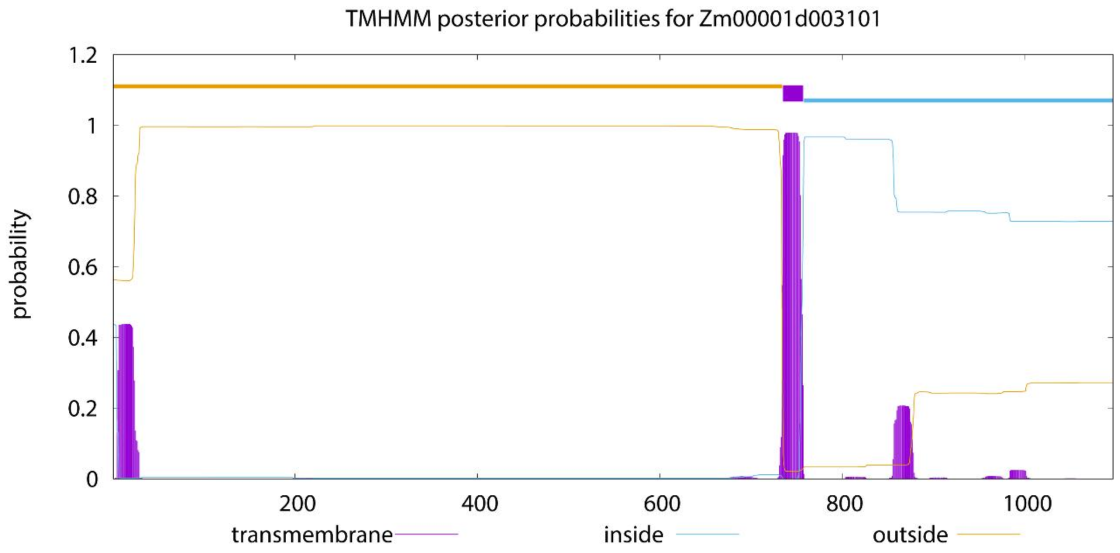
TMHMM posterior probabilities for Zm00001d003101
probability
1.2
1
0.8
0.6
0.4
0.2
0
200
400
600
800
1000
transmembrane
inside
outside

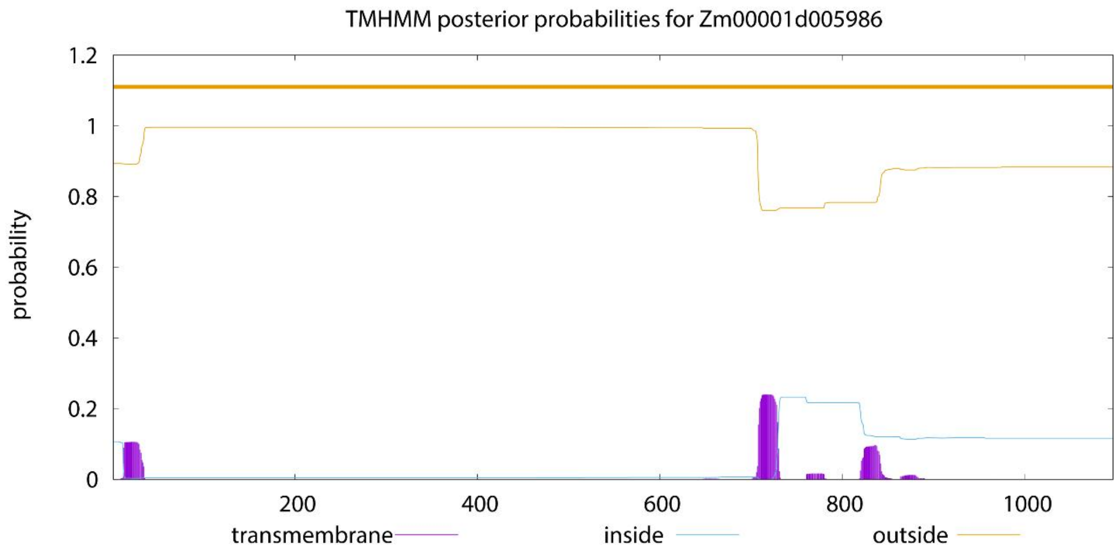
TMHMM posterior probabilities for Zm00001d005986
probability
1.2
1
0.8
0.6
0.4
0.2
0
200
400
600
800
1000
transmembrane
inside
outside

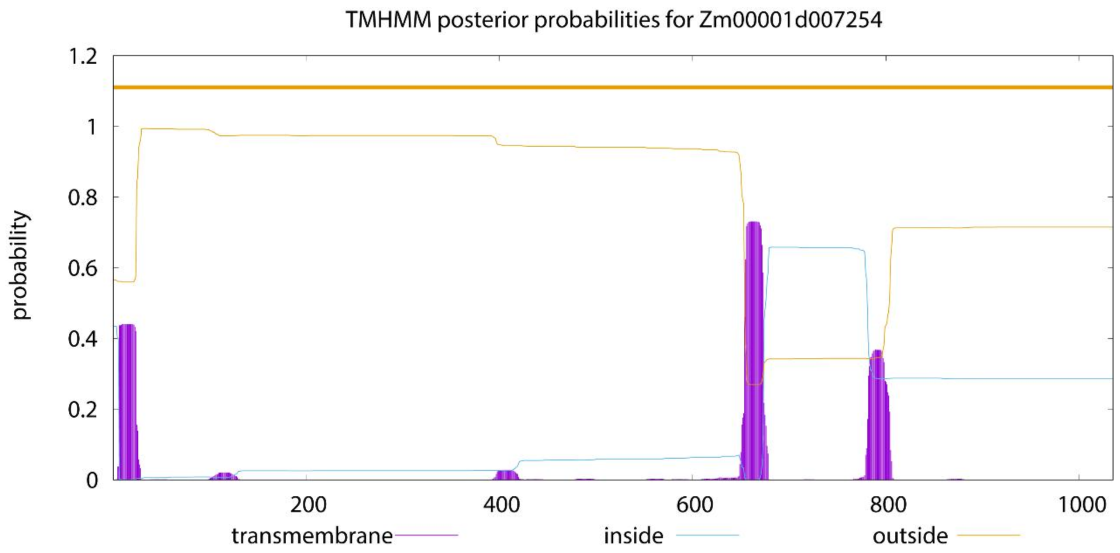
TMHMM posterior probabilities for Zm00001d007254
probability
1.2
1
0.8
0.6
0.4
0.2
0
200
400
600
800
1000
transmembrane
inside
outside

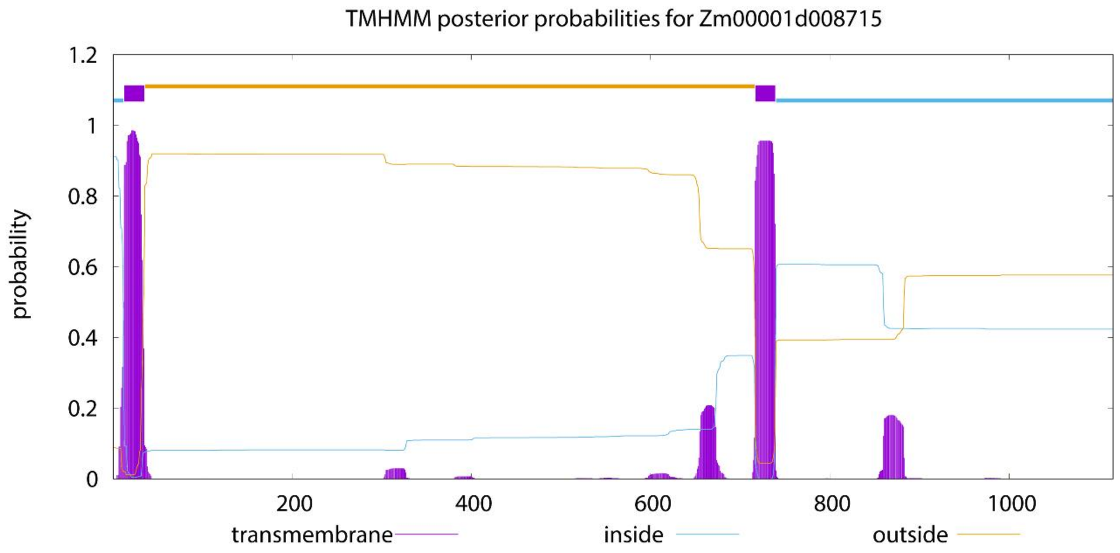
TMHMM posterior probabilities for Zm00001d008715
probability
transmembrane
inside
outside

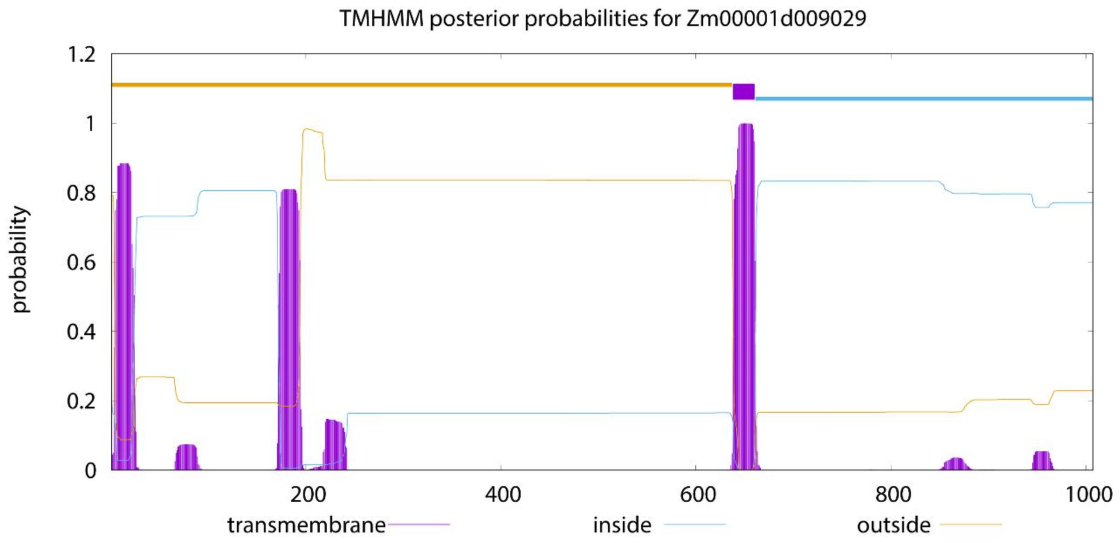
TMHMM posterior probabilities for Zm00001d009029
probability
transmembrane
inside
outside

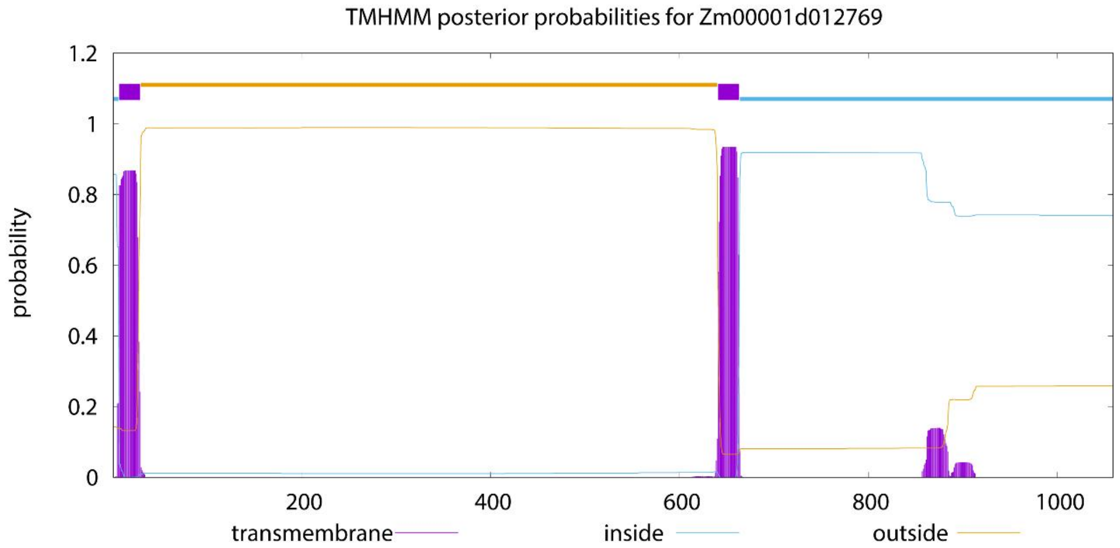
TMHMM posterior probabilities for Zm00001d012769
probability
transmembrane
inside
outside

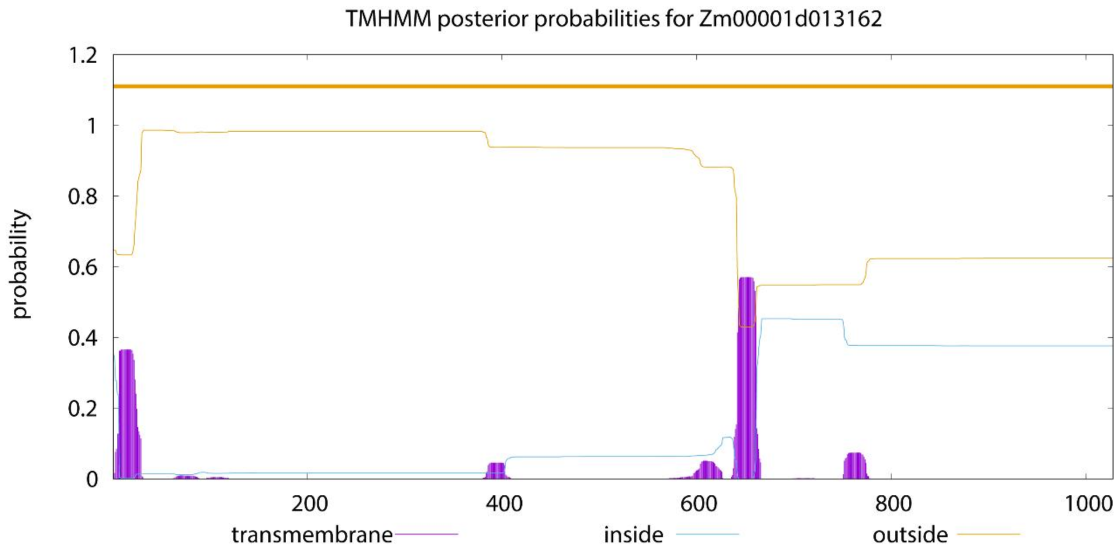
TMHMM posterior probabilities for Zm00001d013162
probability
transmembrane
inside
outside

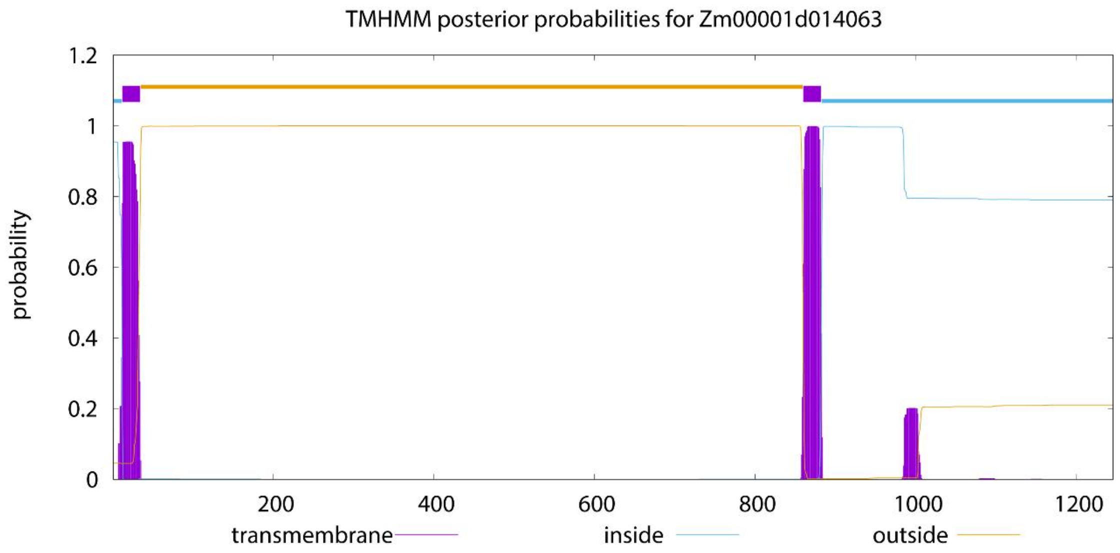
TMHMM posterior probabilities for Zm00001d014063
probability
transmembrane
inside
outside

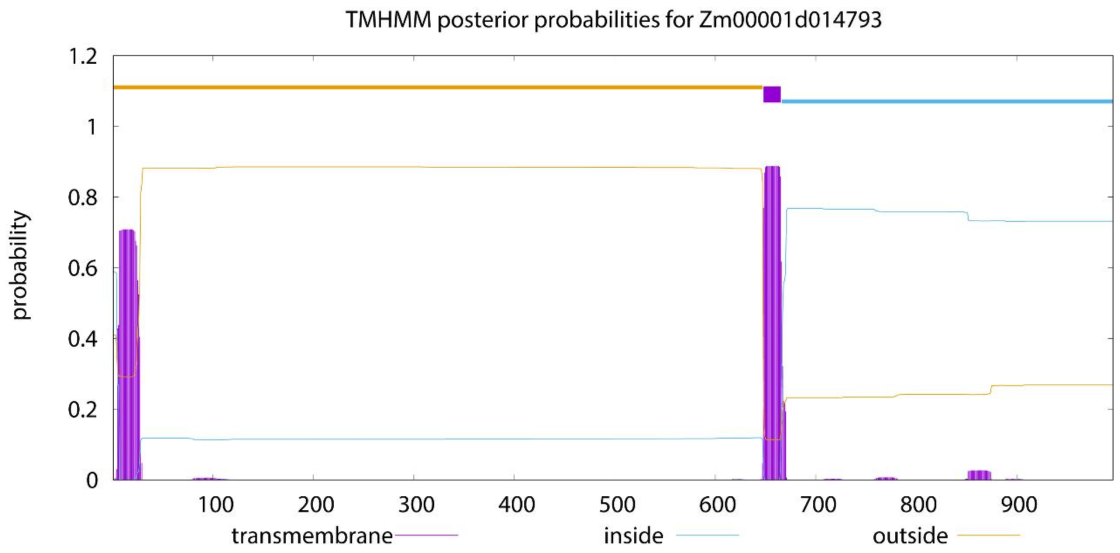
TMHMM posterior probabilities for Zm00001d014793
probability
transmembrane
inside
outside

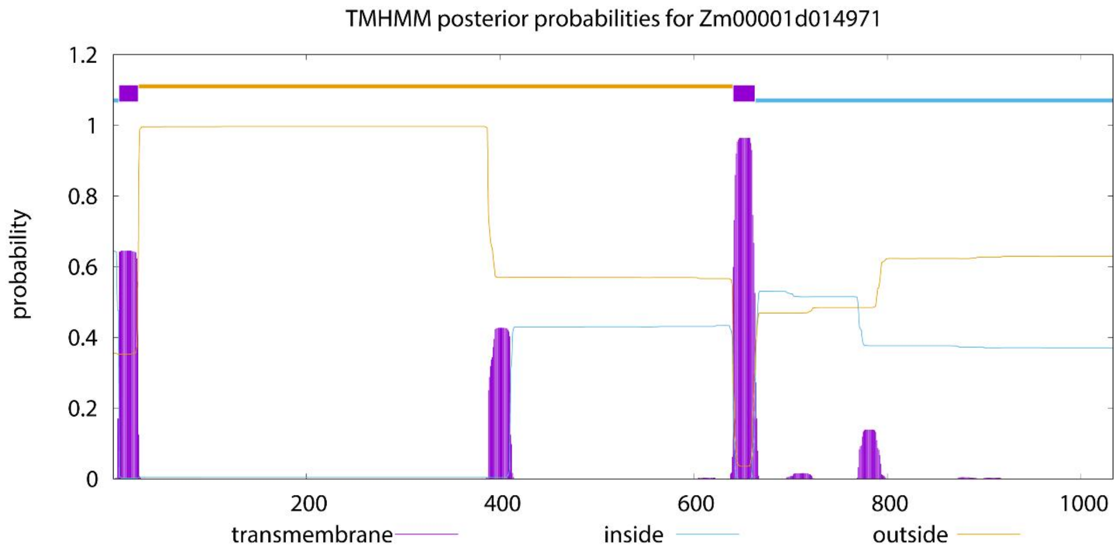
TMHMM posterior probabilities for Zm00001d014971
1.2
1
0.8
0.6
0.4
0.2
0
probability
200
400
600
800
1000
transmembrane
inside
outside

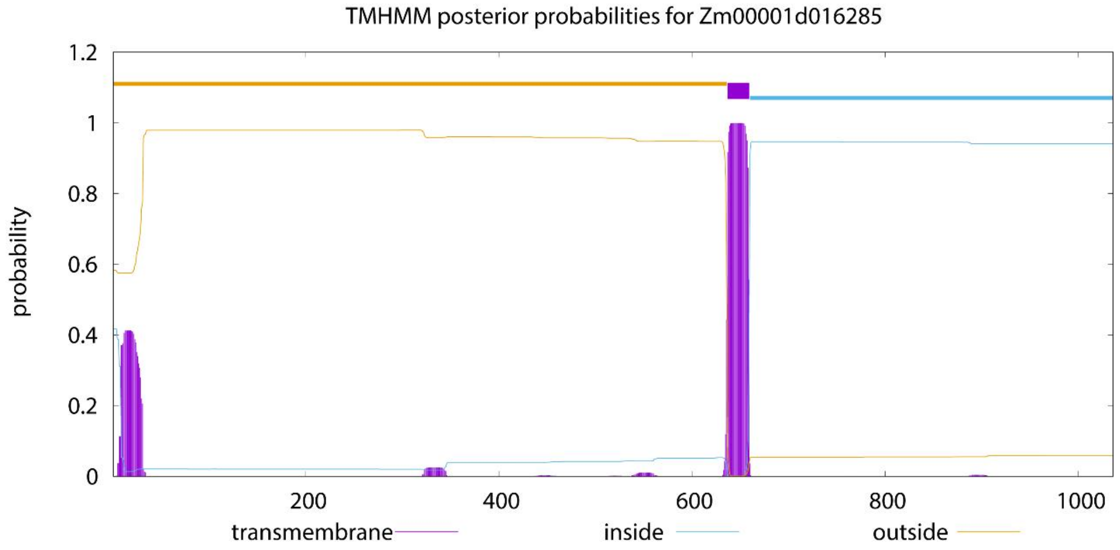
TMHMM posterior probabilities for Zm00001d016285
1.2
1
0.8
0.6
0.4
0.2
0
probability
200
400
600
800
1000
transmembrane
inside
outside

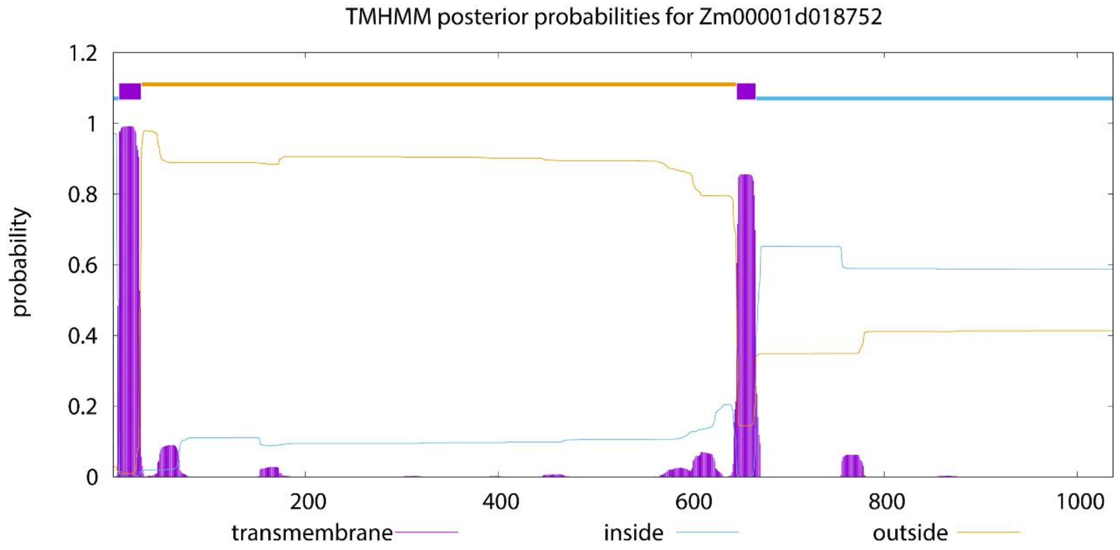
TMHMM posterior probabilities for Zm00001d018752
1.2
1
0.8
0.6
0.4
0.2
0
probability
200
400
600
800
1000
transmembrane
inside
outside

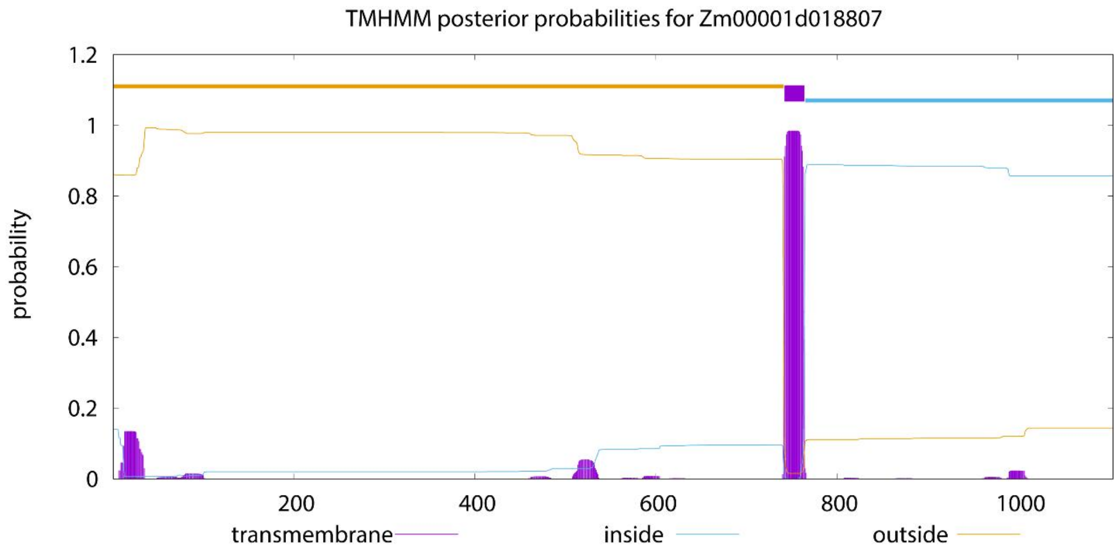
TMHMM posterior probabilities for Zm00001d018807
probability
1.2
1
0.8
0.6
0.4
0.2
0
200
400
600
800
1000
transmembrane
inside
outside

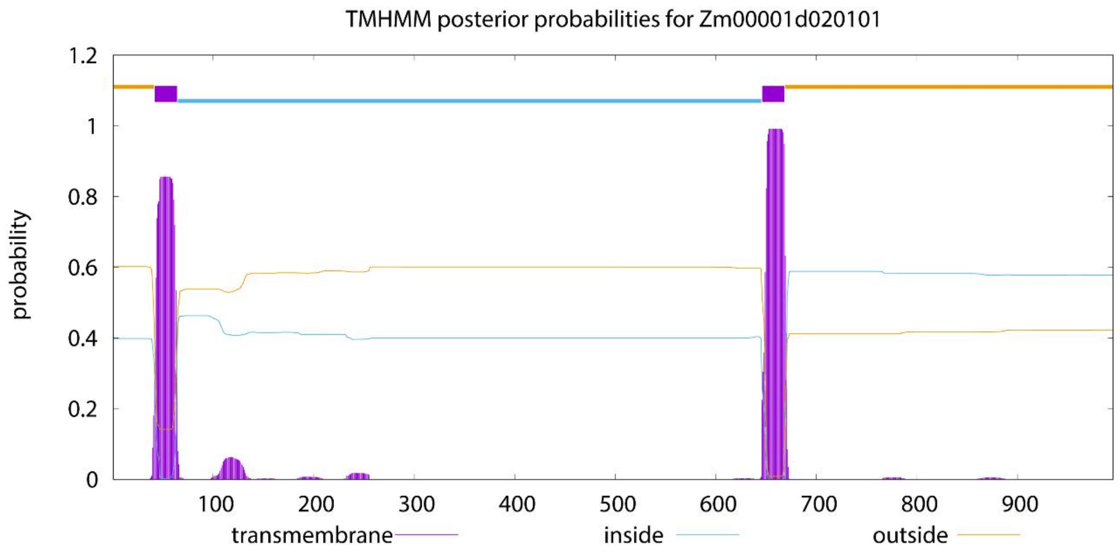
TMHMM posterior probabilities for Zm00001d020101
probability
1.2
1
0.8
0.6
0.4
0.2
0
100
200
300
400
500
600
700
800
900
transmembrane
inside
outside

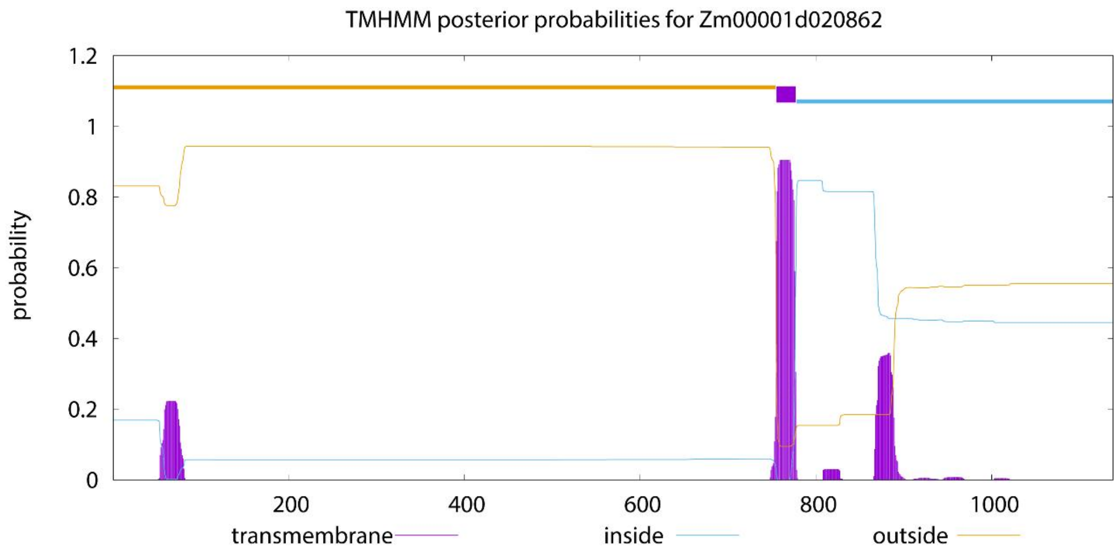
TMHMM posterior probabilities for Zm00001d020862
probability
1.2
1
0.8
0.6
0.4
0.2
0
200
400
600
800
1000
transmembrane
inside
outside

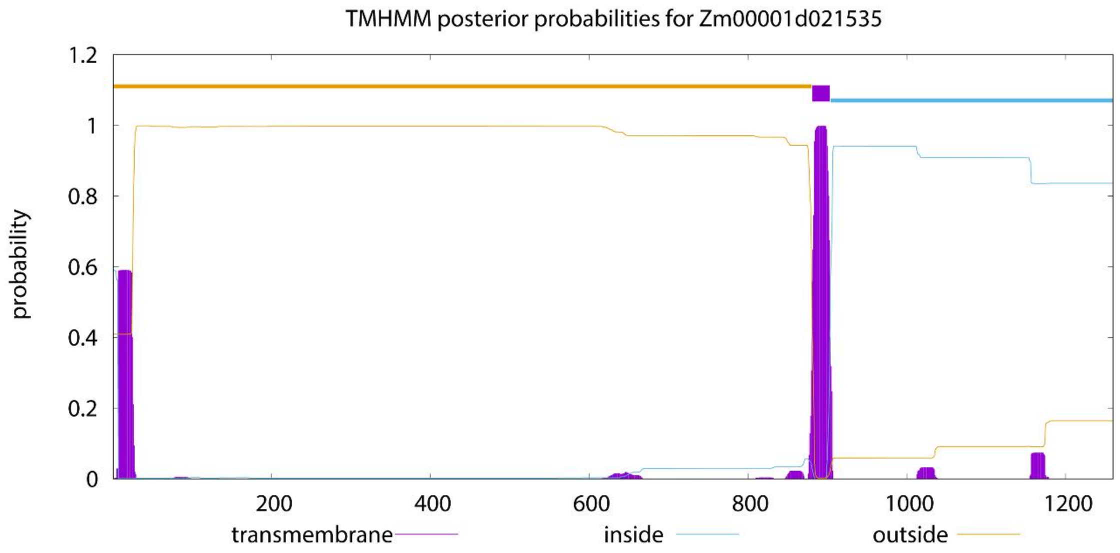
TMHMM posterior probabilities for Zm00001d021535
probability
transmembrane
inside
outside

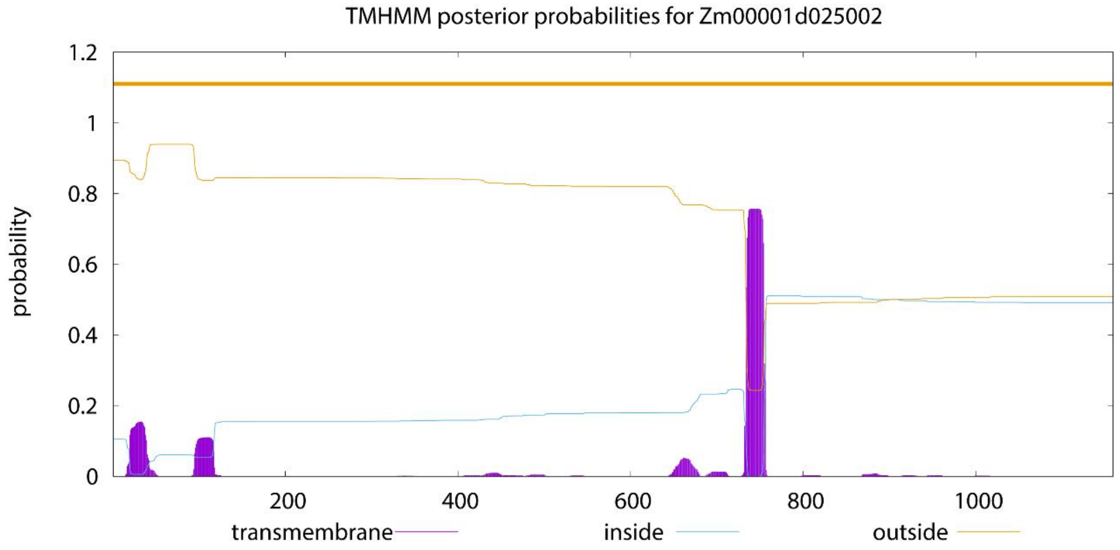
TMHMM posterior probabilities for Zm00001d025002
probability
transmembrane
inside
outside

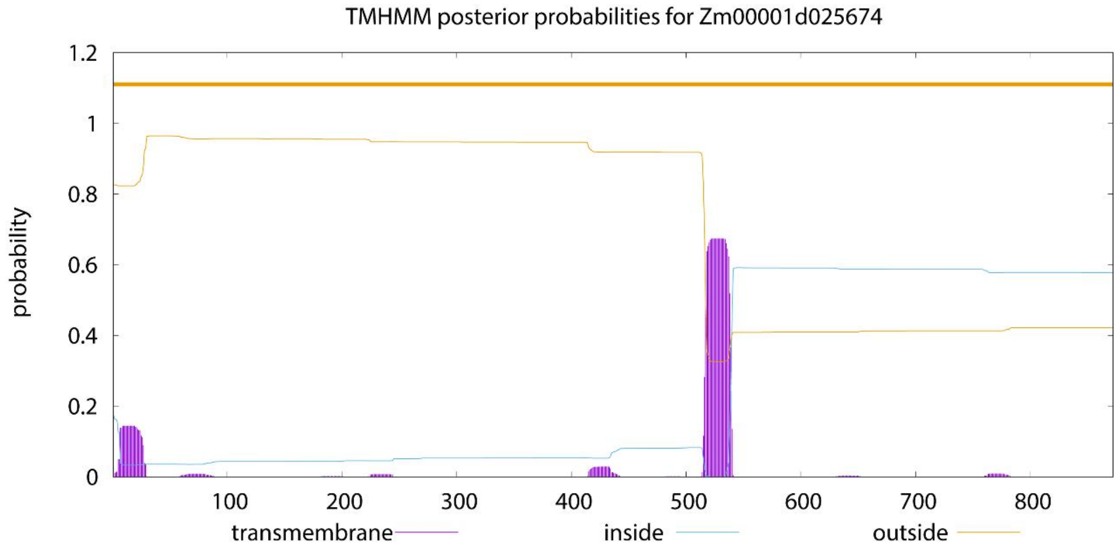
TMHMM posterior probabilities for Zm00001d025674
probability
transmembrane
inside
outside

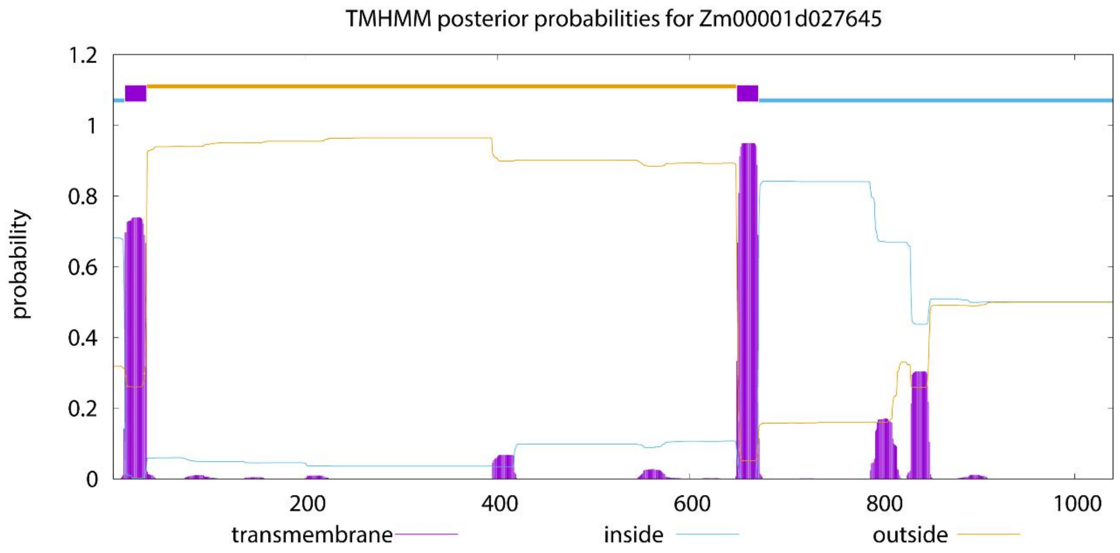

TMHMM posterior probabilities for Zm00001d027645
1.2
1
0.8
0.6
0.4
0.2
0
probability
200
400
600
800
1000
transmembrane
inside
outside

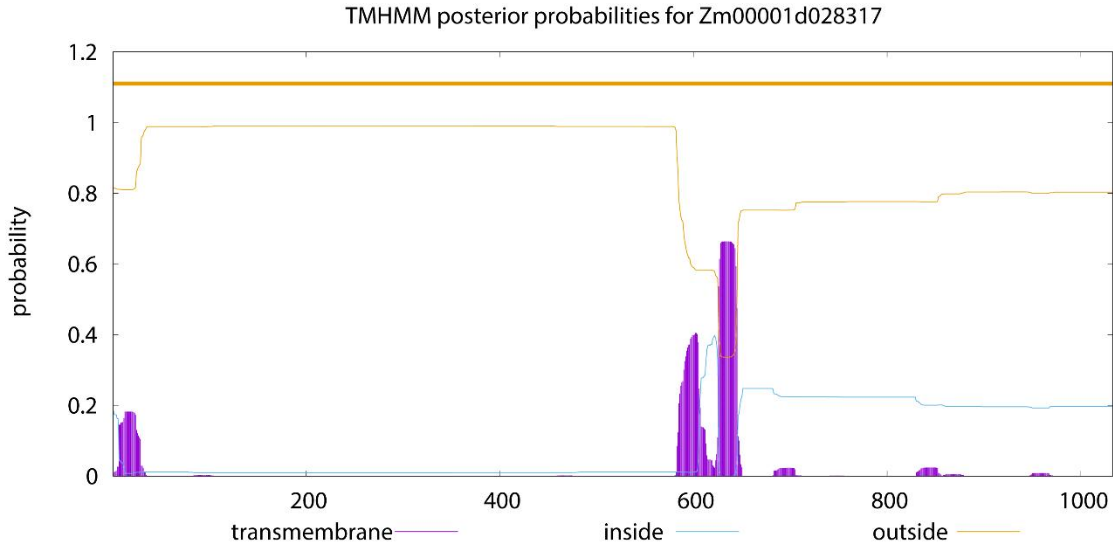

TMHMM posterior probabilities for Zm00001d028317
1.2
1
0.8
0.6
0.4
0.2
0
probability
200
400
600
800
1000
transmembrane
inside
outside

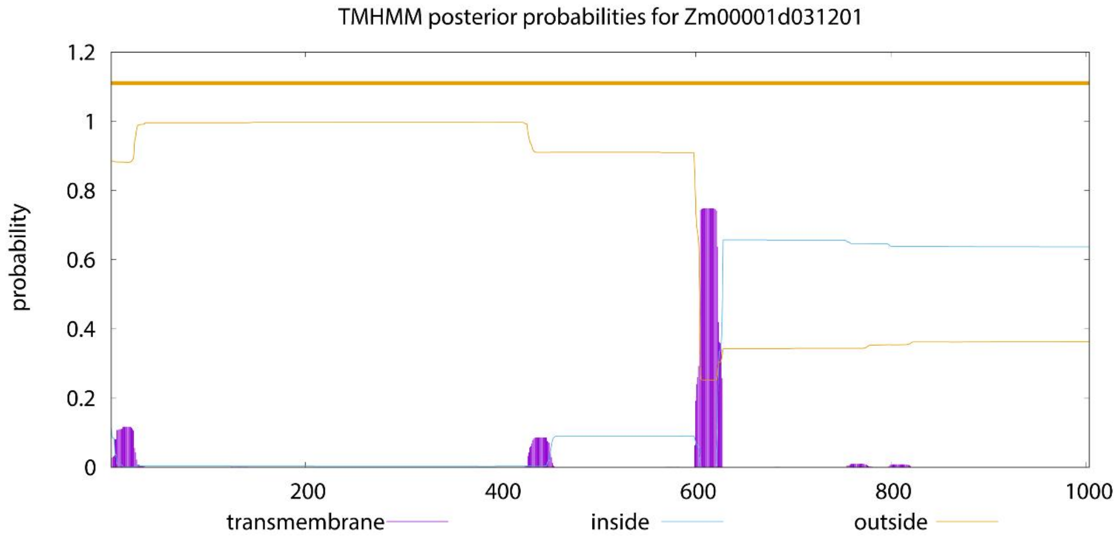

TMHMM posterior probabilities for Zm00001d031201
1.2
1
0.8
0.6
0.4
0.2
0
probability
200
400
600
800
1000
transmembrane
inside
outside

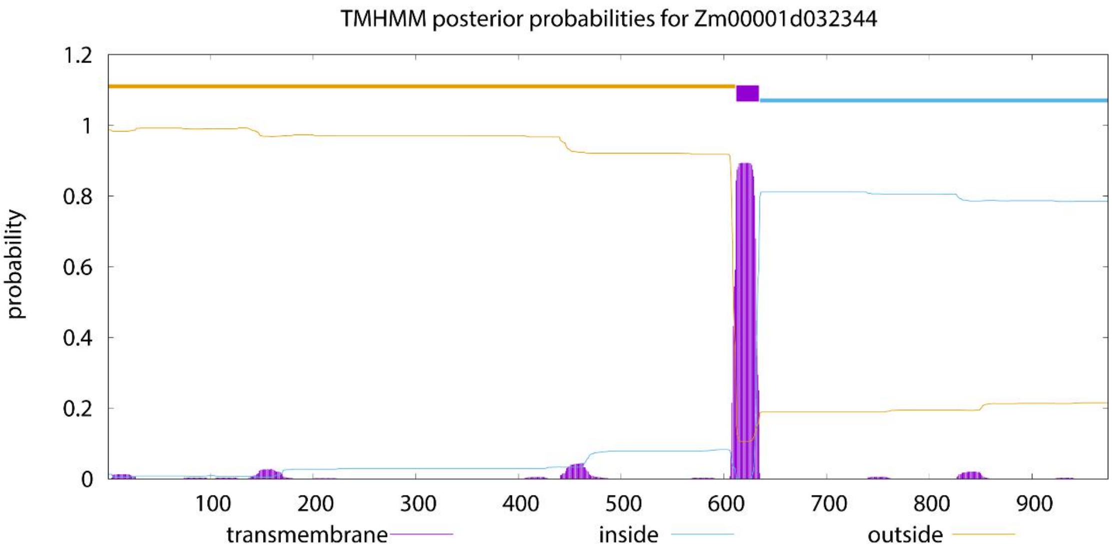
TMHMM posterior probabilities for Zm00001d032344
probability
1.2
1
0.8
0.6
0.4
0.2
0
100 200 300 400 500 600 700 800 900
transmembrane
inside
outside

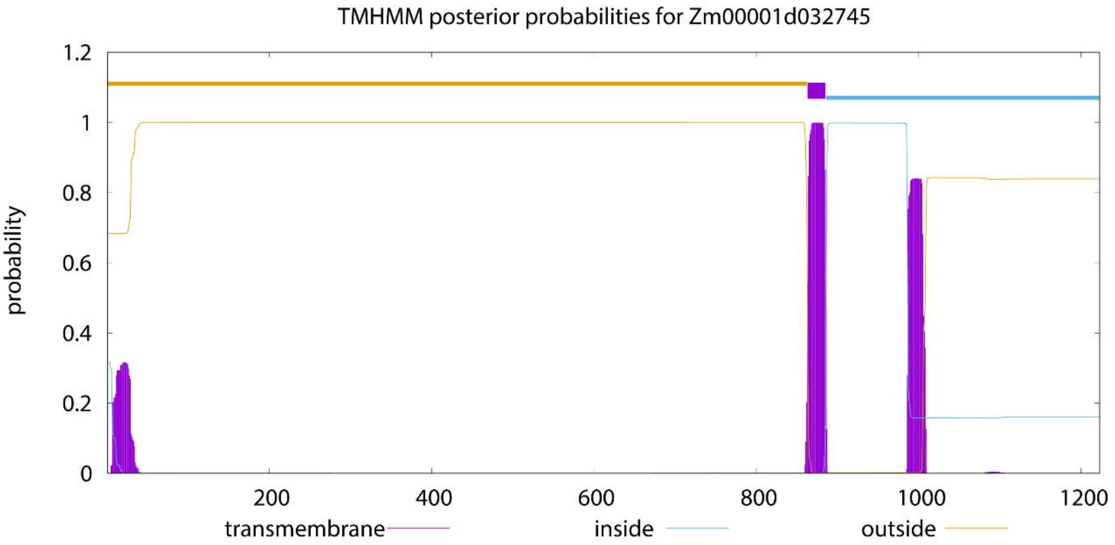
TMHMM posterior probabilities for Zm00001d032745
probability
1.2
1
0.8
0.6
0.4
0.2
0
200 400 600 800 1000 1200
transmembrane
inside
outside

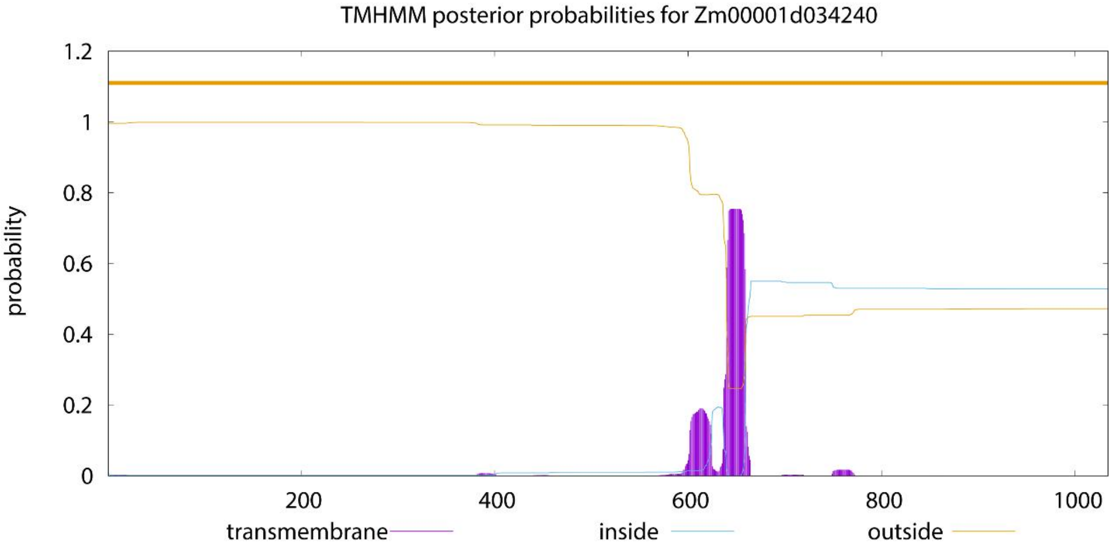
TMHMM posterior probabilities for Zm00001d034240
probability
1.2
1
0.8
0.6
0.4
0.2
0
200 400 600 800 1000
transmembrane
inside
outside

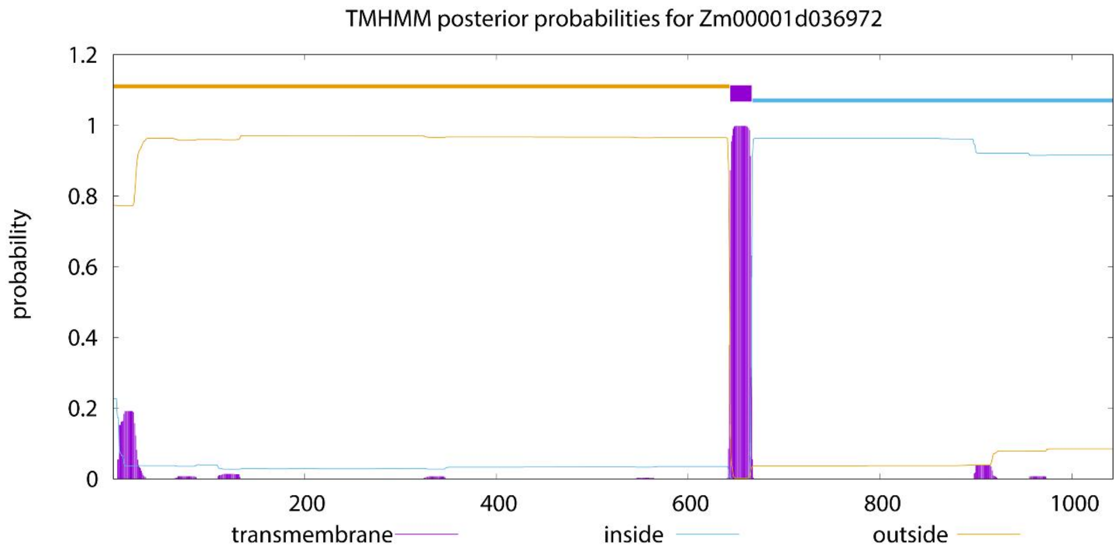
TMHMM posterior probabilities for Zm00001d036972
probability
transmembrane
inside
outside

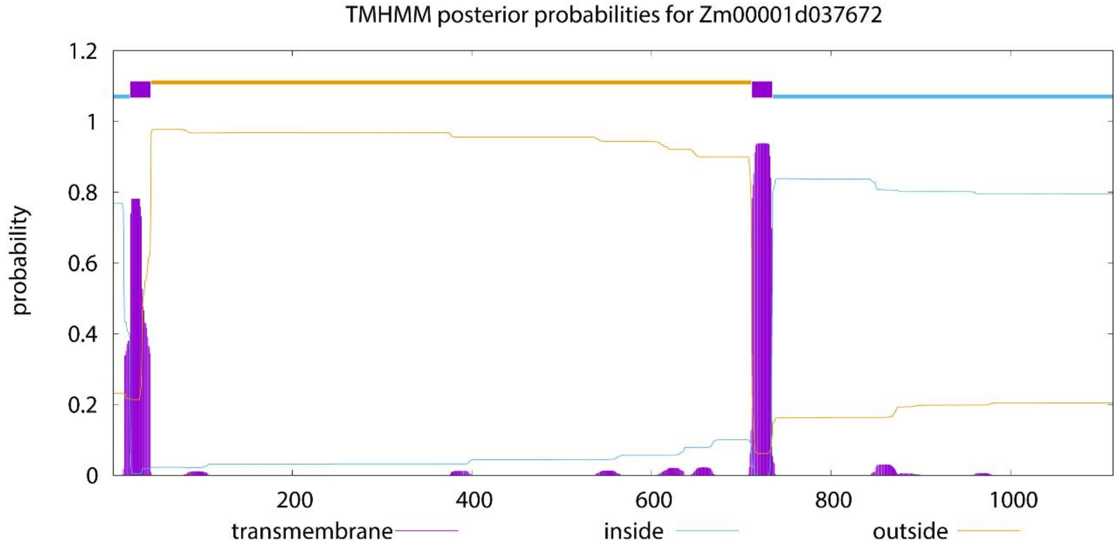
TMHMM posterior probabilities for Zm00001d037672
probability
transmembrane
inside
outside

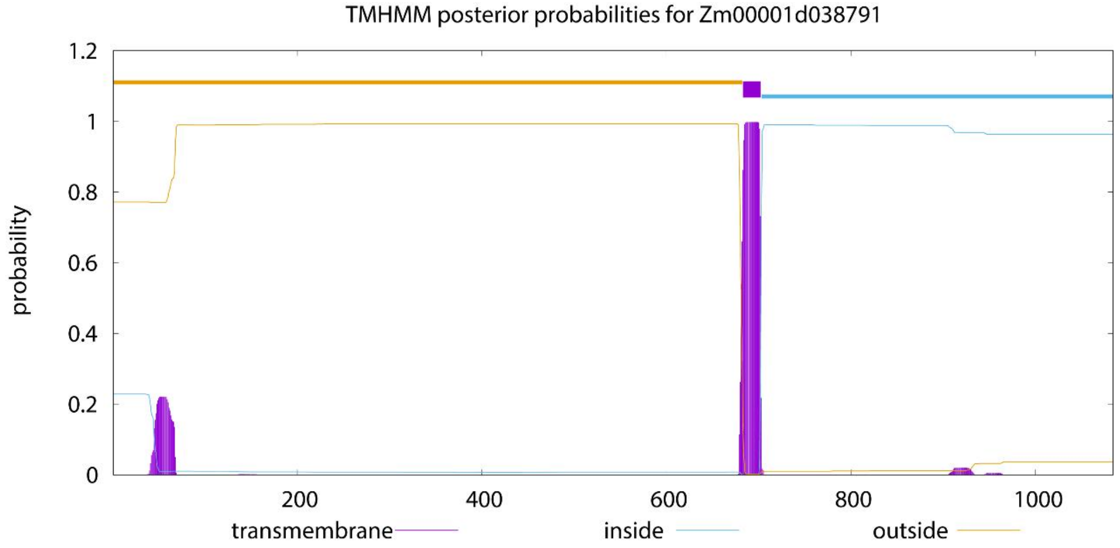
TMHMM posterior probabilities for Zm00001d038791
probability
transmembrane
inside
outside

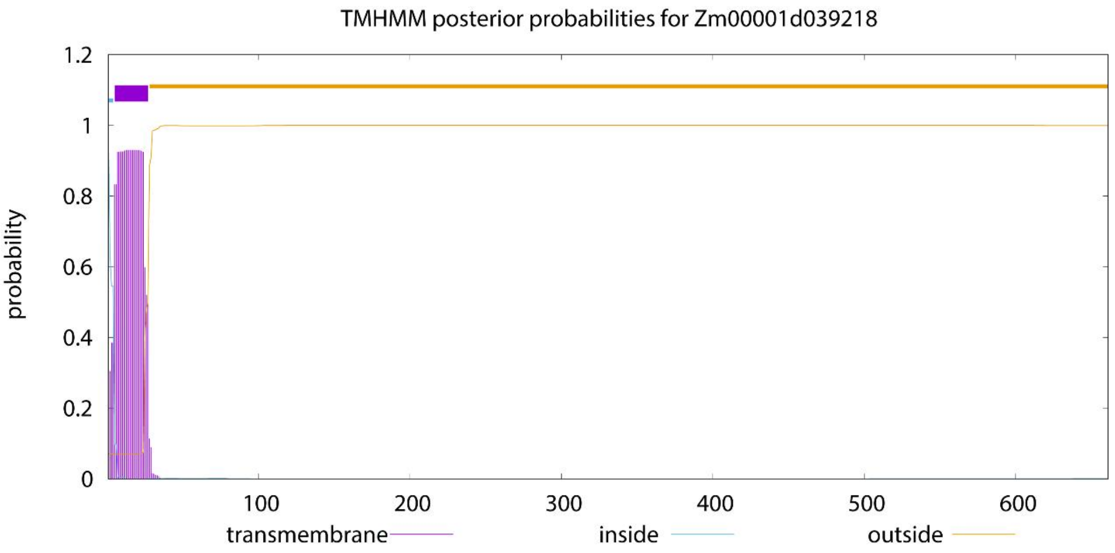

TMHMM posterior probabilities for Zm00001d039218
probability
1.2
1
0.8
0.6
0.4
0.2
0
100
200
300
400
500
600
transmembrane
inside
outside

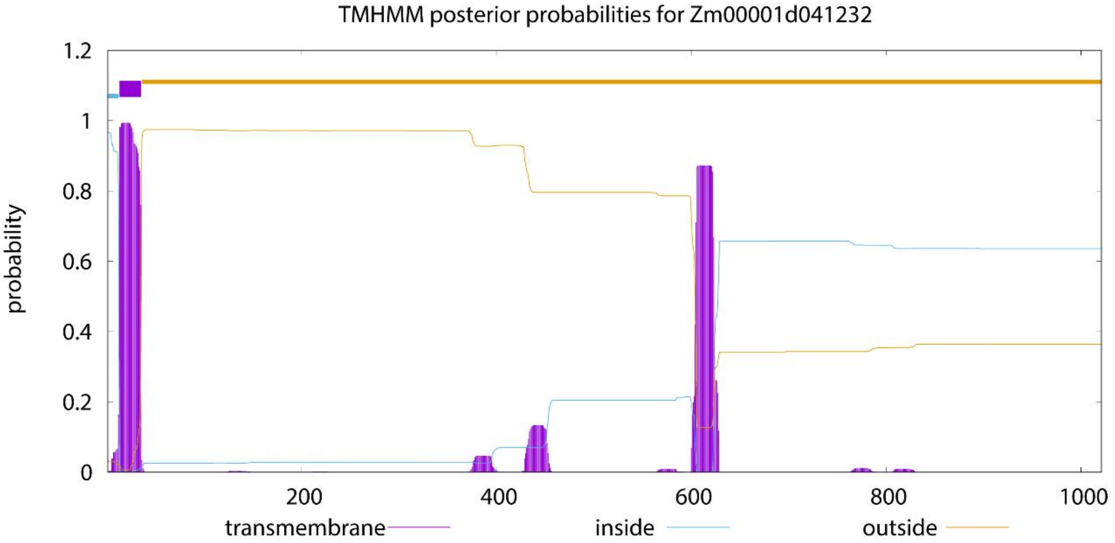

TMHMM posterior probabilities for Zm00001d041232
probability
1.2
1
0.8
0.6
0.4
0.2
0
200
400
600
800
1000
transmembrane
inside
outside

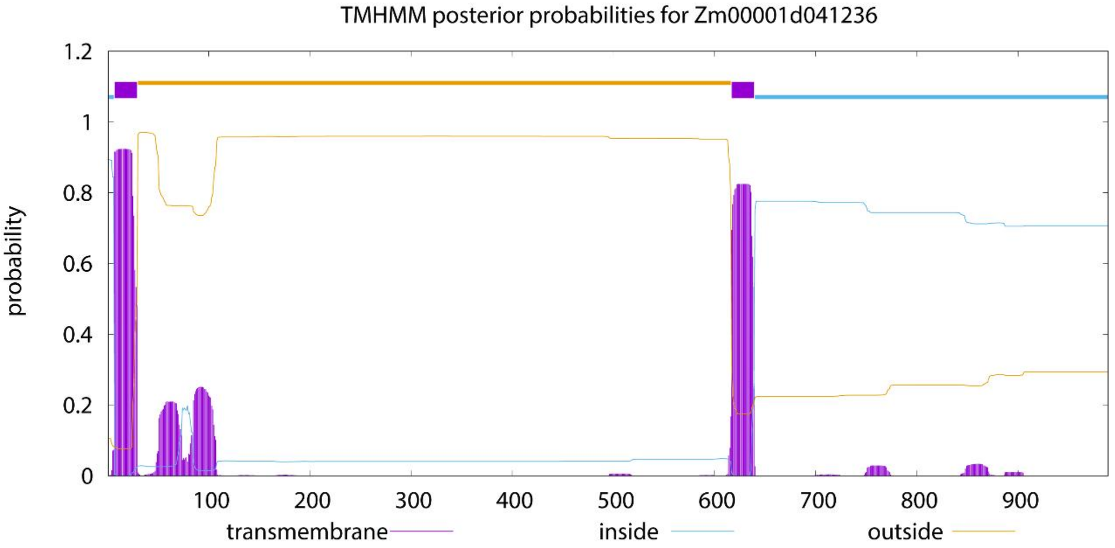

TMHMM posterior probabilities for Zm00001d041236
probability
1.2
1
0.8
0.6
0.4
0.2
0
100
200
300
400
500
600
700
800
900
transmembrane
inside
outside

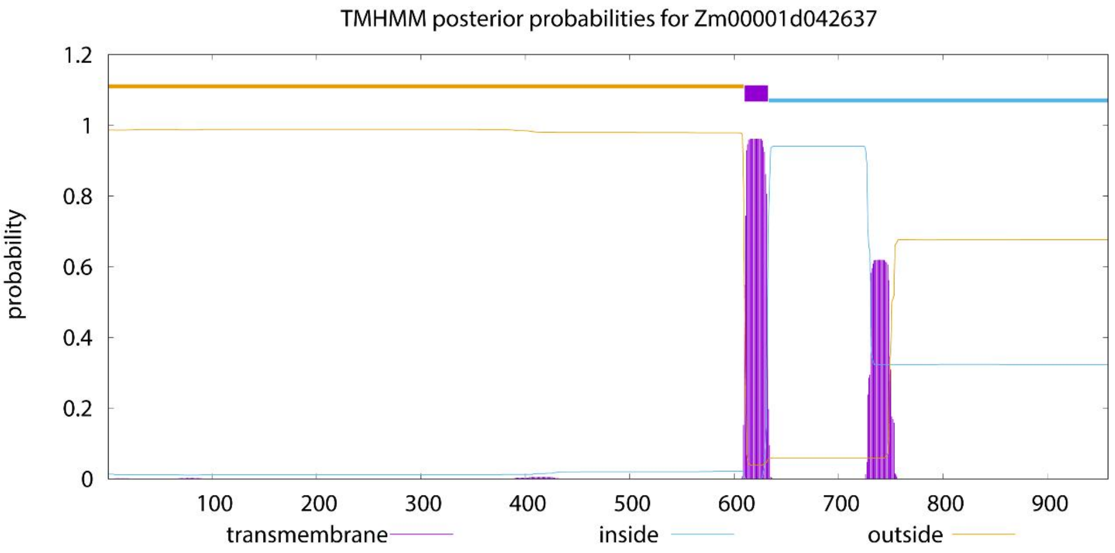
TMHMM posterior probabilities for Zm00001d042637
probability
1.2
1
0.8
0.6
0.4
0.2
0
100
200
300
400
500
600
700
800
900
transmembrane
inside
outside

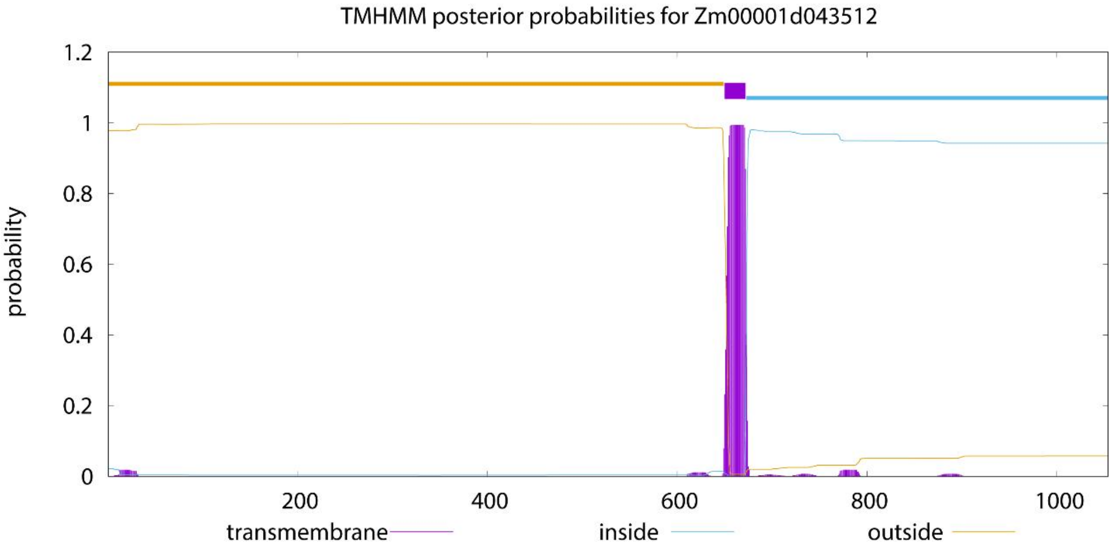
TMHMM posterior probabilities for Zm00001d043512
probability
1.2
1
0.8
0.6
0.4
0.2
0
200
400
600
800
1000
transmembrane
inside
outside

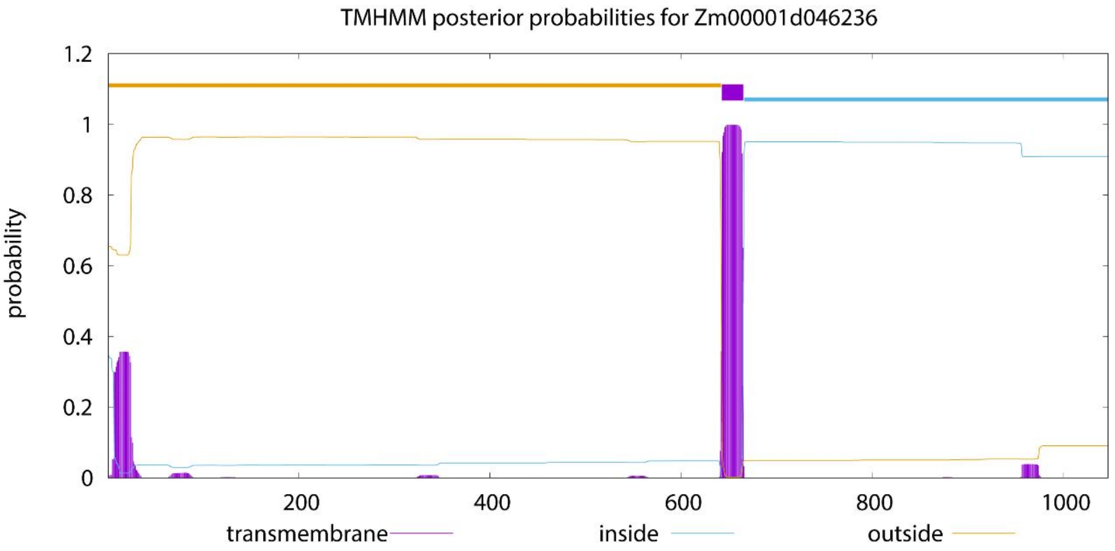
TMHMM posterior probabilities for Zm00001d046236
probability
1.2
1
0.8
0.6
0.4
0.2
0
200
400
600
800
1000
transmembrane
inside
outside

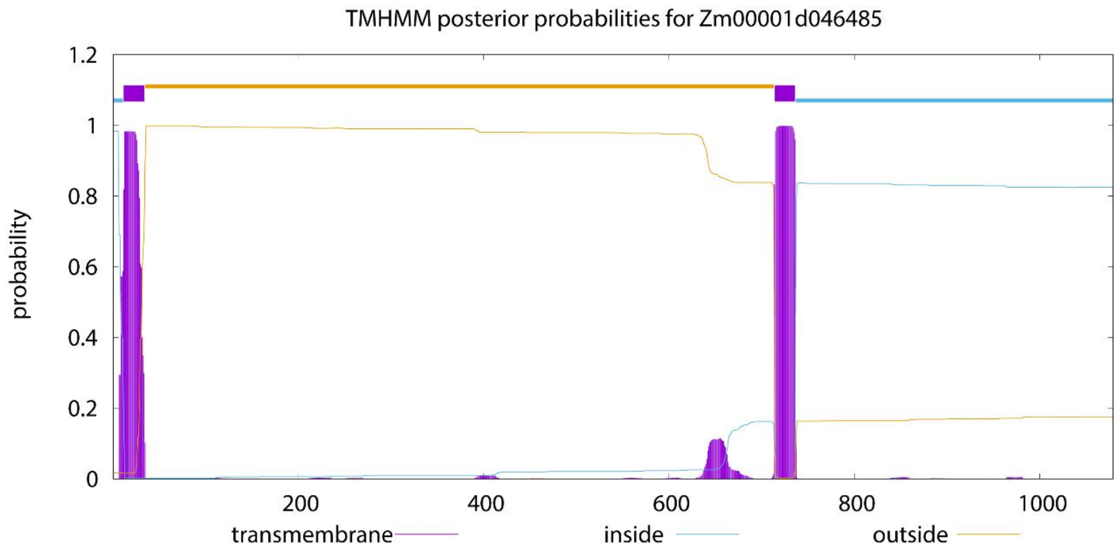
TMHMM posterior probabilities for Zm00001d046485
probability
1.2
1
0.8
0.6
0.4
0.2
0
200
400
600
800
1000
transmembrane
inside
outside

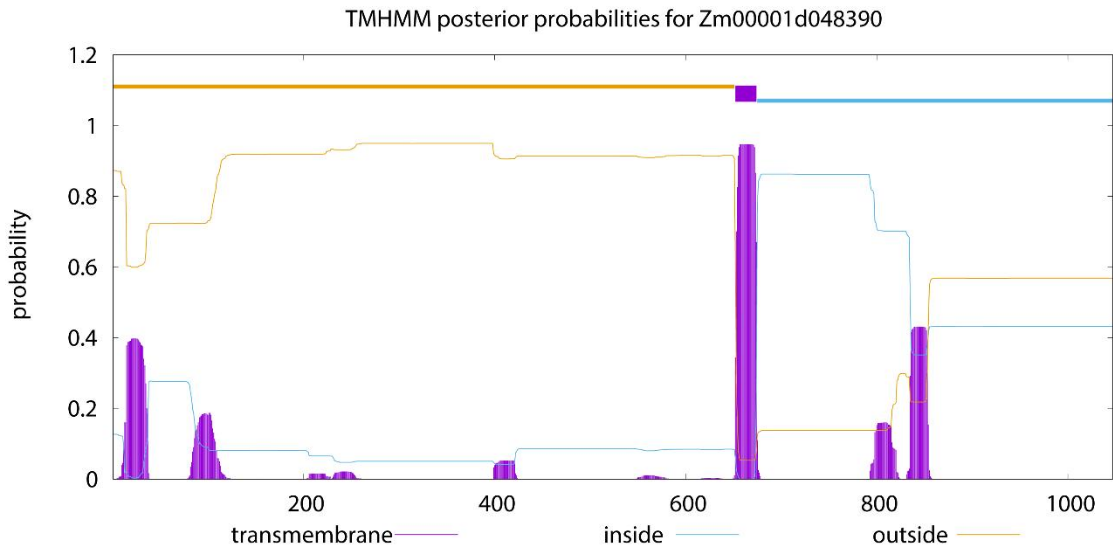
TMHMM posterior probabilities for Zm00001d048390
probability
1.2
1
0.8
0.6
0.4
0.2
0
200
400
600
800
1000
transmembrane
inside
outside

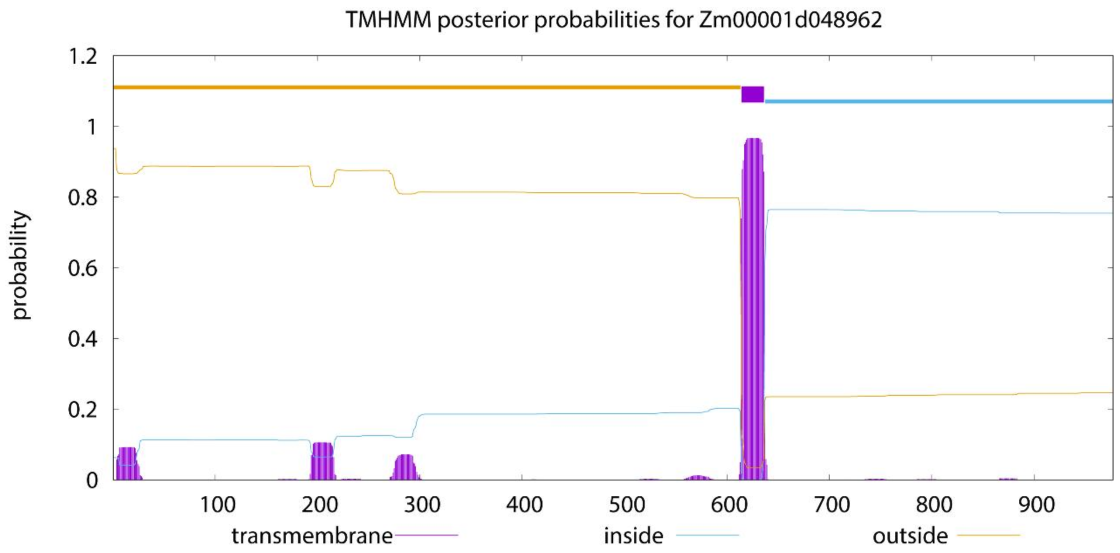
TMHMM posterior probabilities for Zm00001d048962
probability
1.2
1
0.8
0.6
0.4
0.2
0
100
200
300
400
500
600
700
800
900
transmembrane
inside
outside

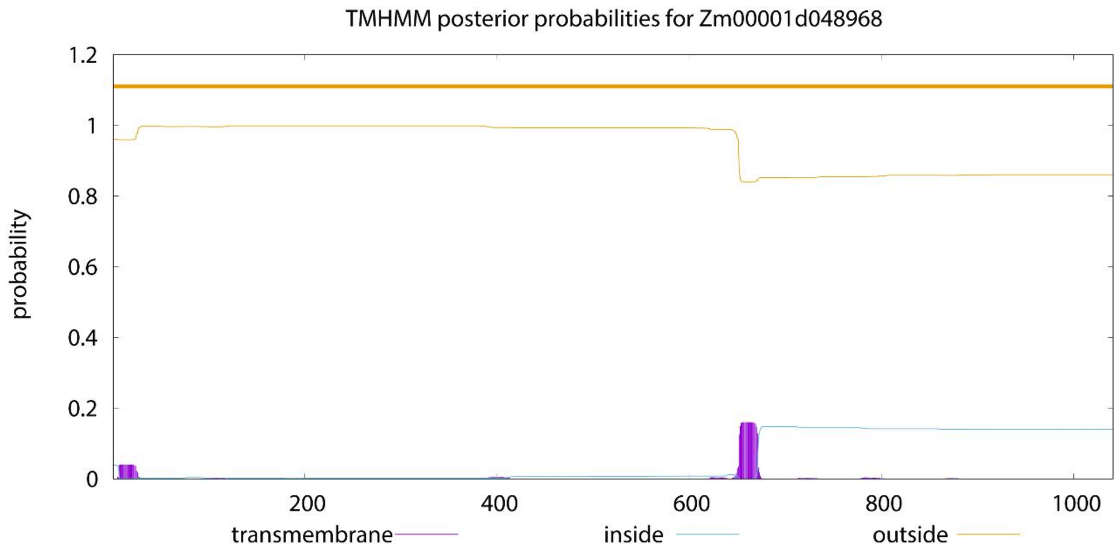
TMHMM posterior probabilities for Zm00001d048968
probability
transmembrane
inside
outside

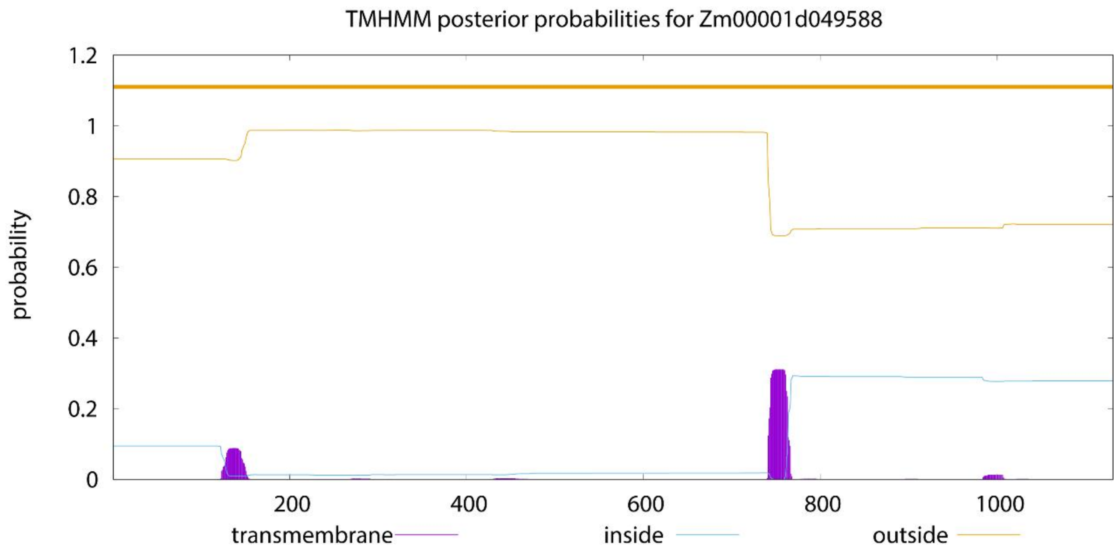
TMHMM posterior probabilities for Zm00001d049588
probability
transmembrane
inside
outside

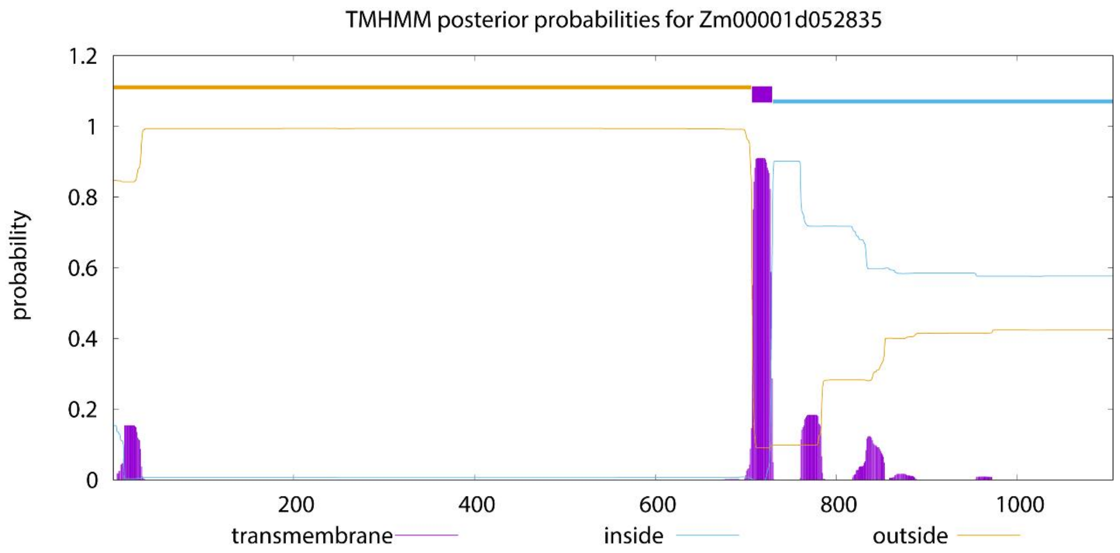
TMHMM posterior probabilities for Zm00001d052835
probability
transmembrane
inside
outside

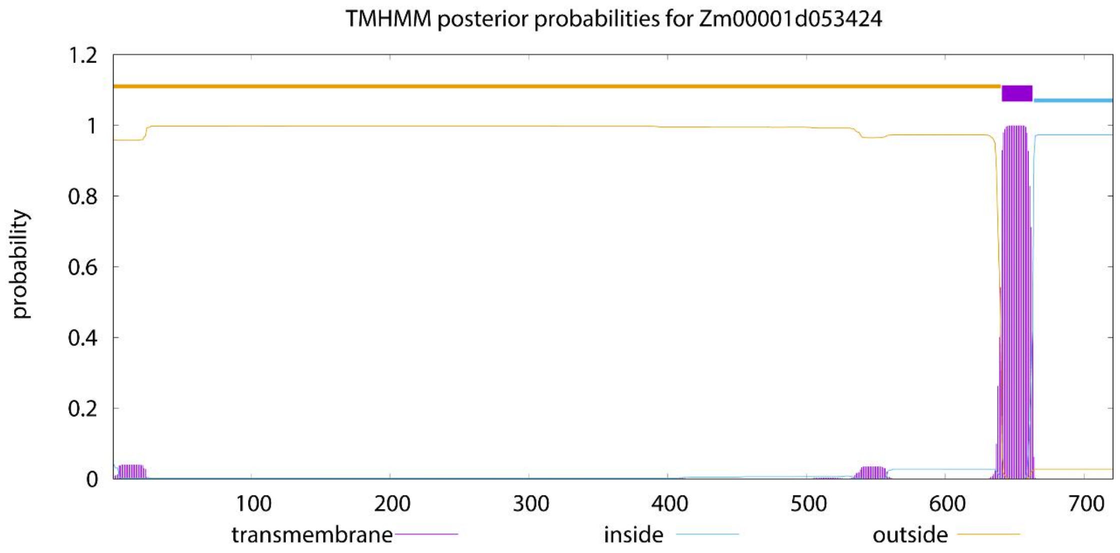

Transmembrane structure domain of subfamily XI.

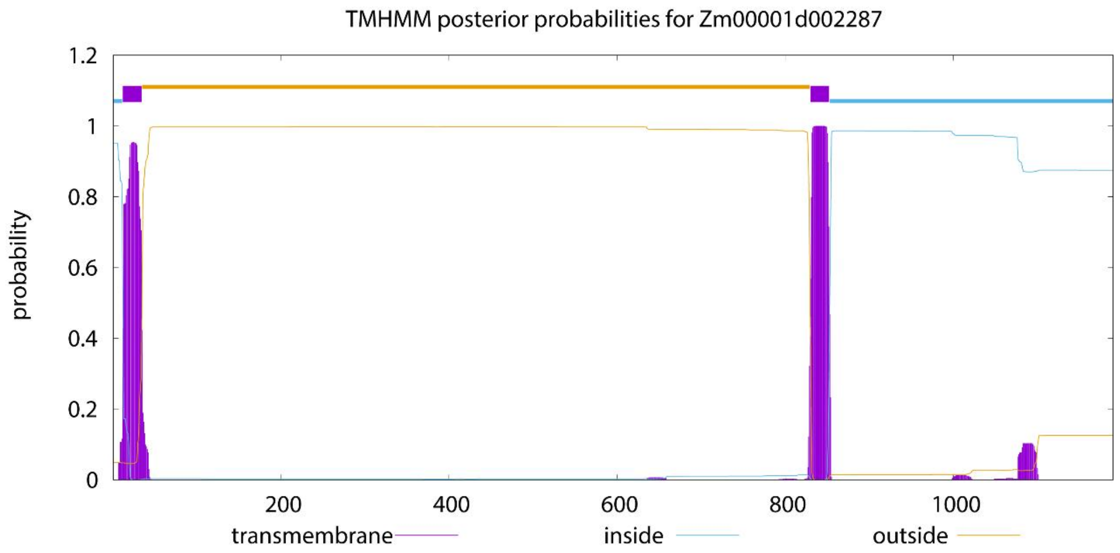

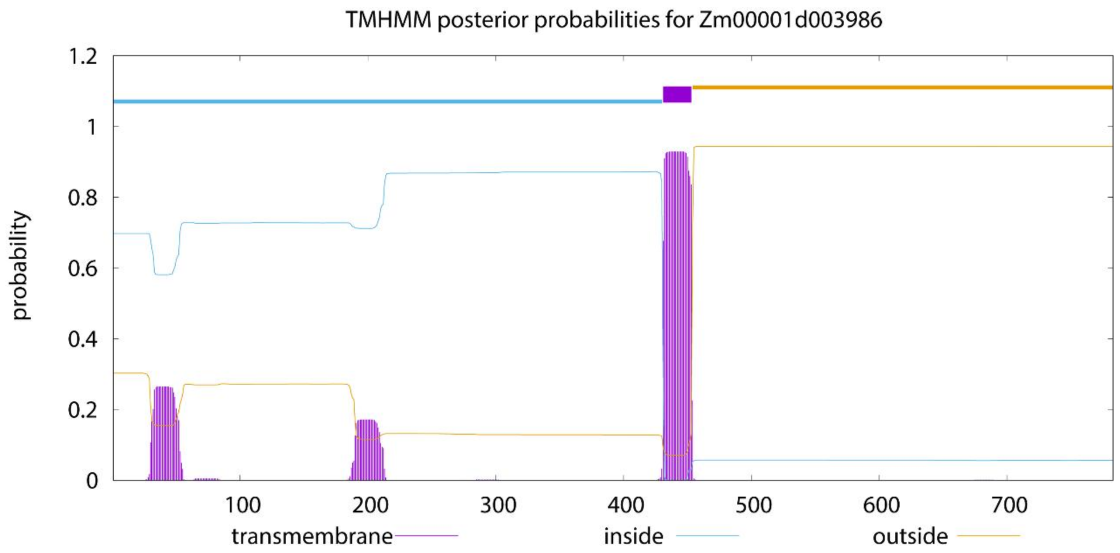

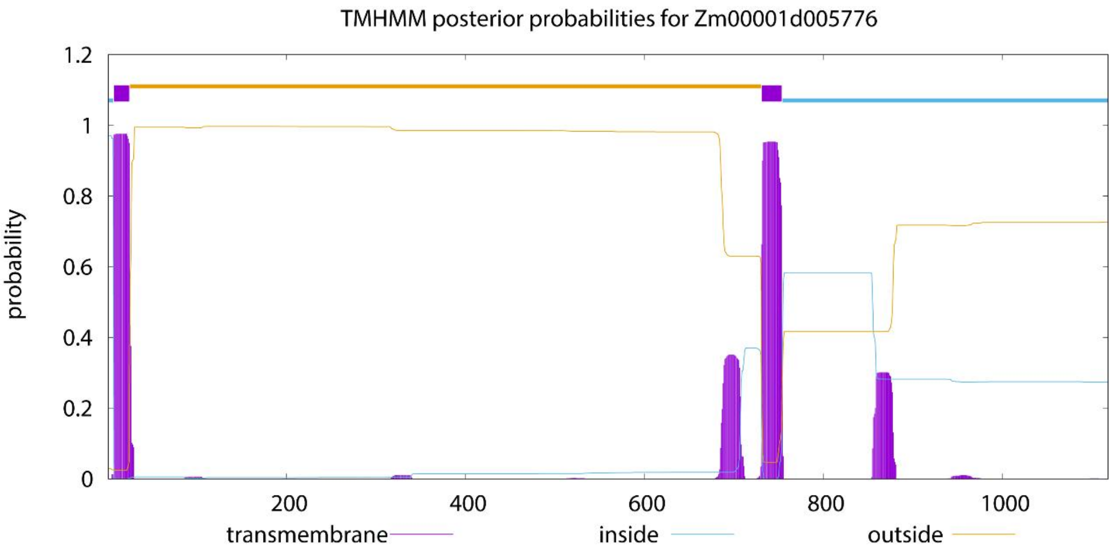
TMHMM posterior probabilities for Zm00001d005776
probability
1.2
1
0.8
0.6
0.4
0.2
0
200
400
600
800
1000
transmembrane
inside
outside

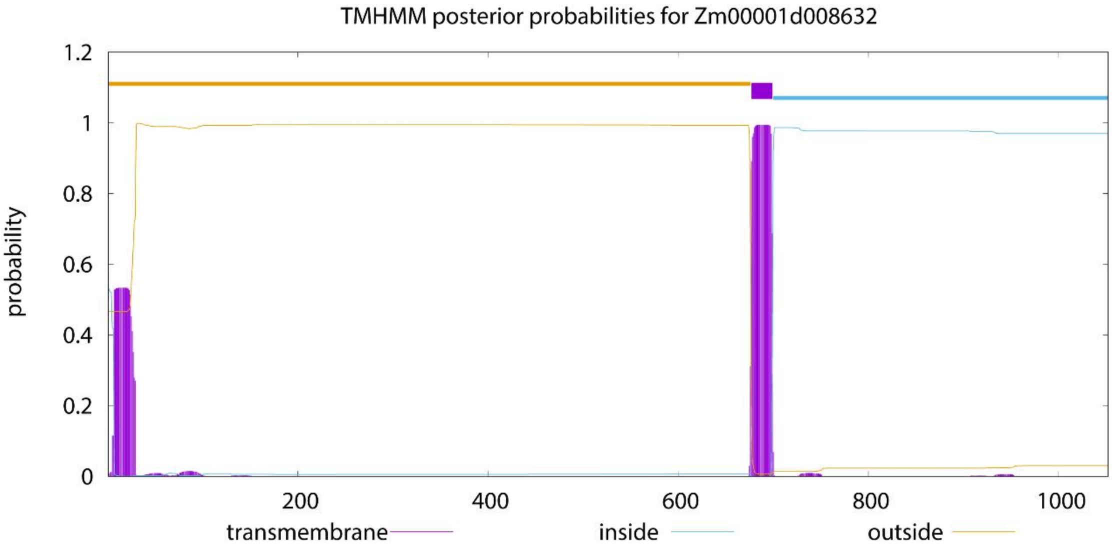
TMHMM posterior probabilities for Zm00001d008632
probability
1.2
1
0.8
0.6
0.4
0.2
0
200
400
600
800
1000
transmembrane
inside
outside

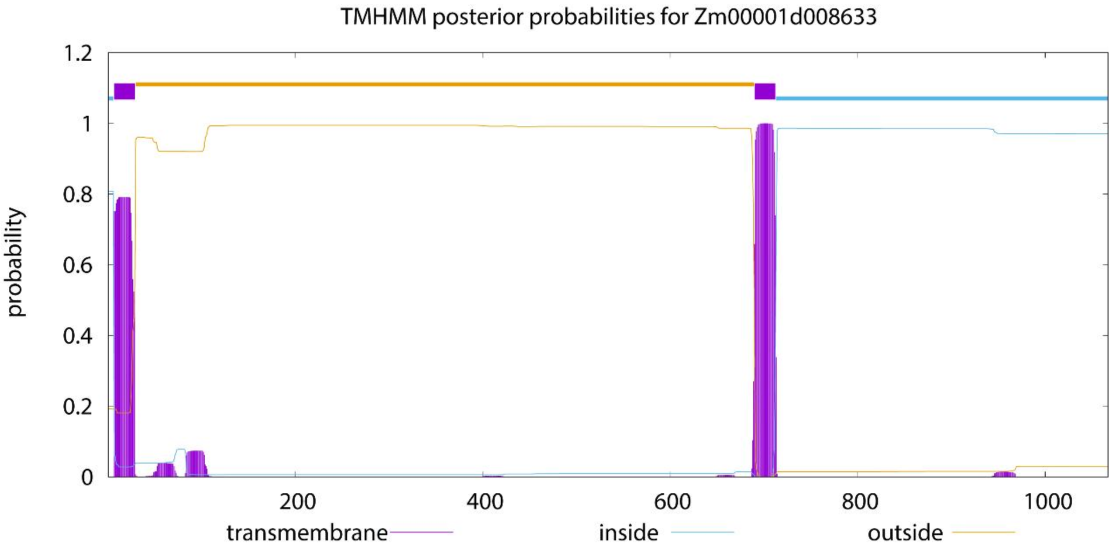
TMHMM posterior probabilities for Zm00001d008633
probability
1.2
1
0.8
0.6
0.4
0.2
0
200
400
600
800
1000
transmembrane
inside
outside

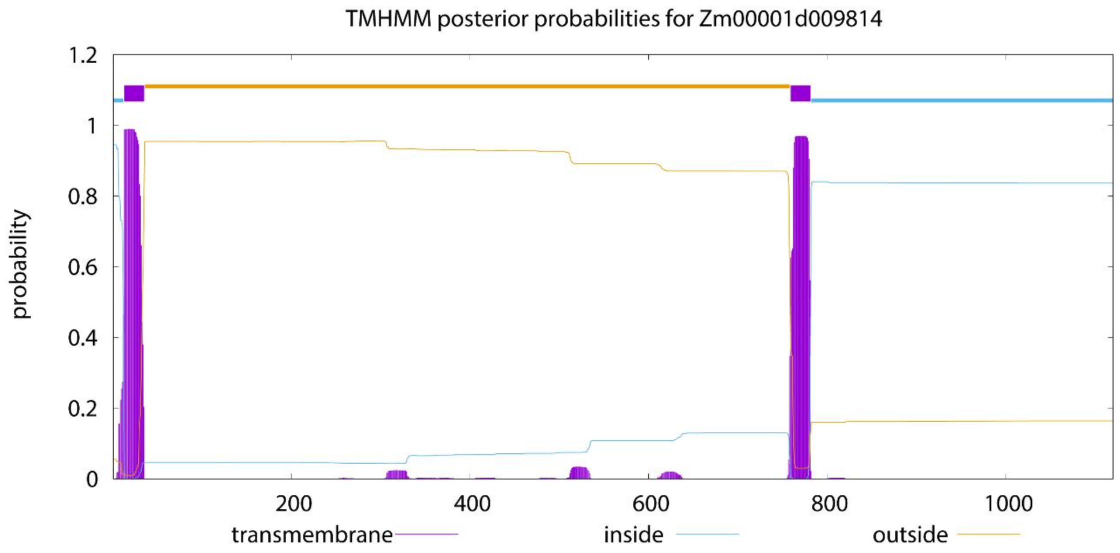
TMHMM posterior probabilities for Zm00001d009814
probability
1.2
1
0.8
0.6
0.4
0.2
0
200
400
600
800
1000
transmembrane
inside
outside

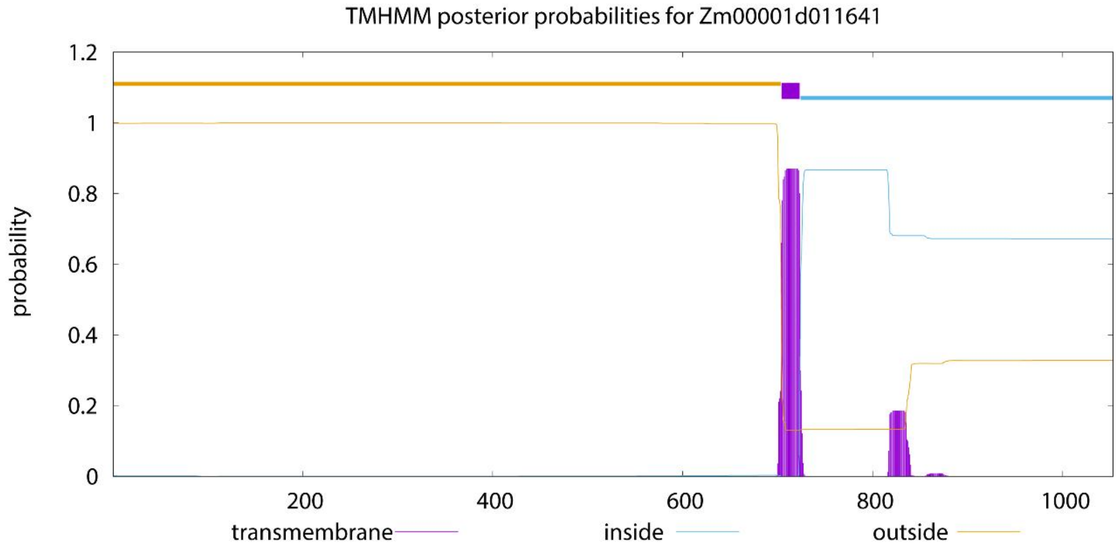
TMHMM posterior probabilities for Zm00001d011641
probability
1.2
1
0.8
0.6
0.4
0.2
0
200
400
600
800
1000
transmembrane
inside
outside

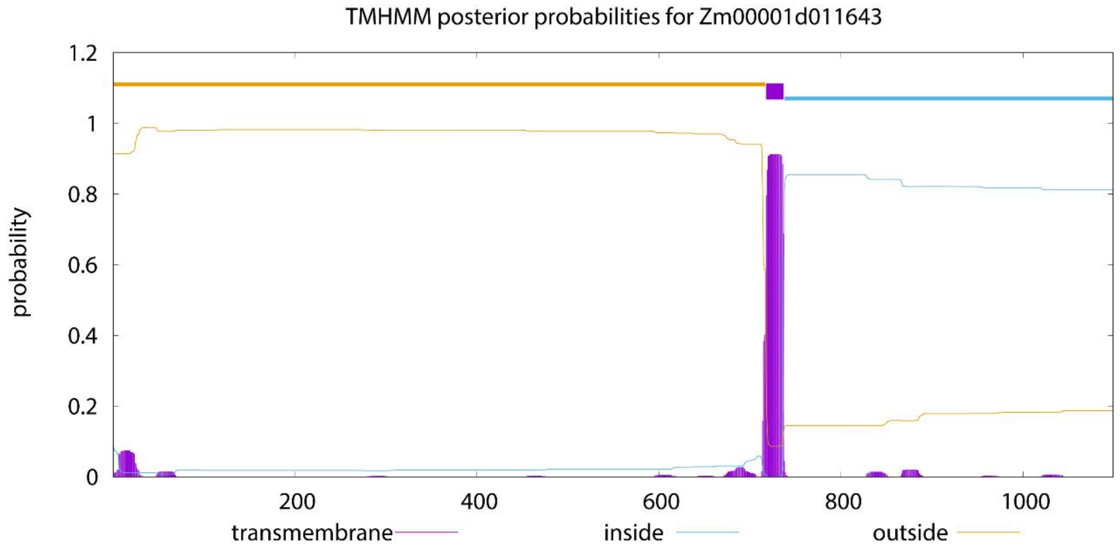
TMHMM posterior probabilities for Zm00001d011643
probability
1.2
1
0.8
0.6
0.4
0.2
0
200
400
600
800
1000
transmembrane
inside
outside

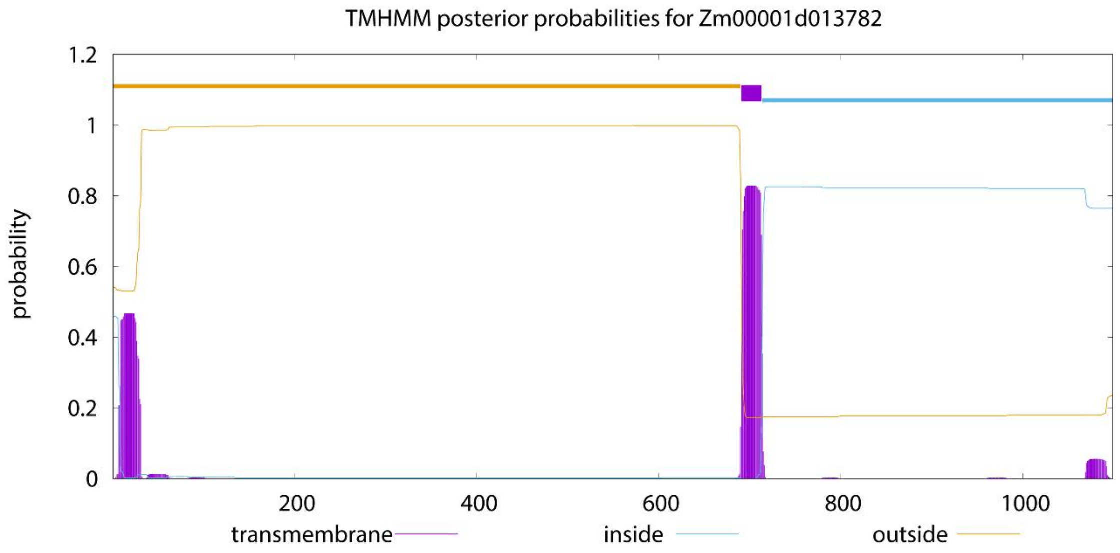
TMHMM posterior probabilities for Zm00001d013782
1.2
1
0.8
0.6
0.4
0.2
0
probability
200
400
600
800
1000
transmembrane
inside
outside

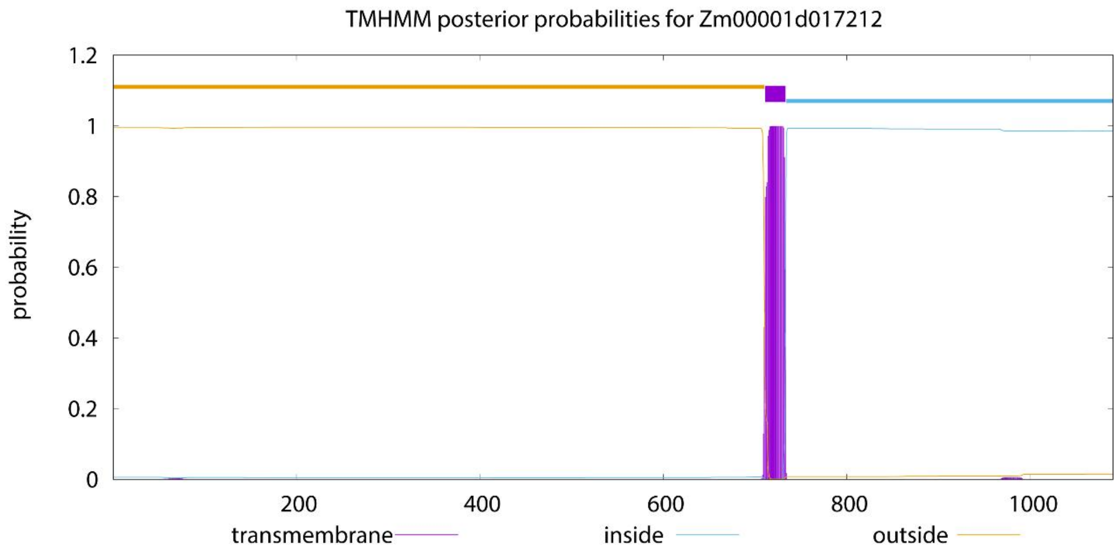
TMHMM posterior probabilities for Zm00001d017212
1.2
1
0.8
0.6
0.4
0.2
0
probability
200
400
600
800
1000
transmembrane
inside
outside

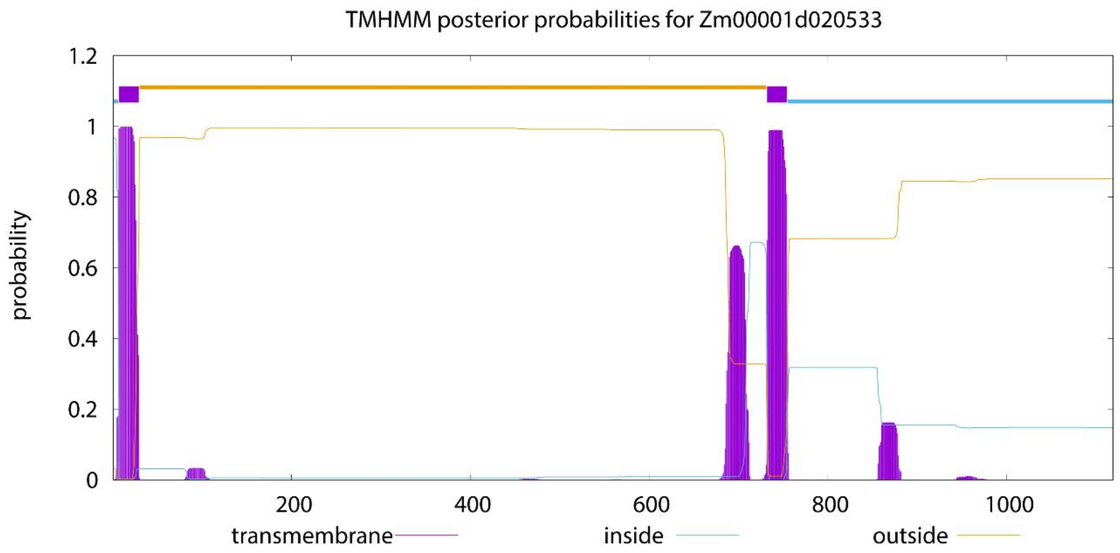
TMHMM posterior probabilities for Zm00001d020533
1.2
1
0.8
0.6
0.4
0.2
0
probability
200
400
600
800
1000
transmembrane
inside
outside

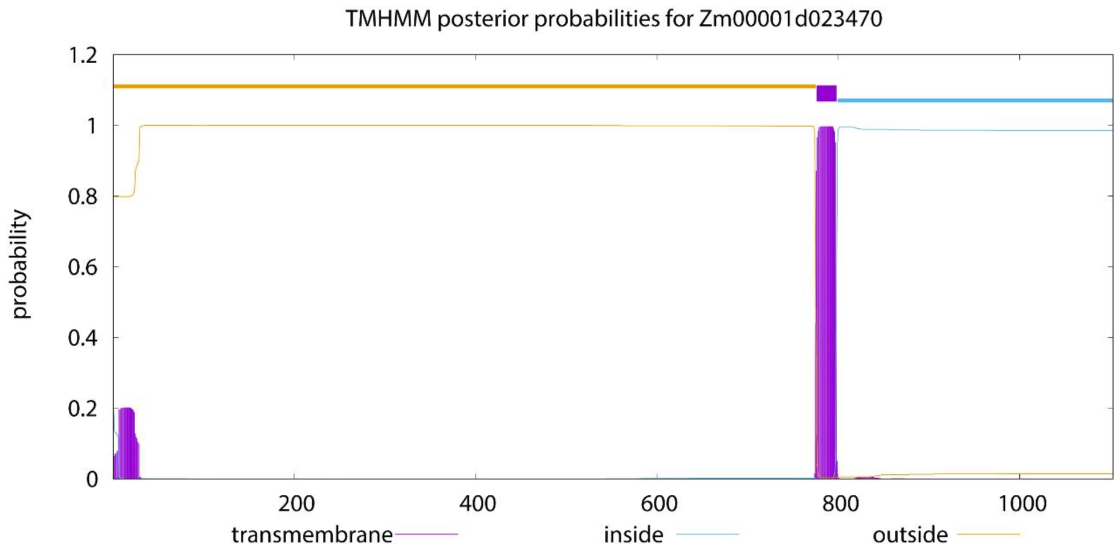

TMHMM posterior probabilities for Zm00001d023470
probability
1.2
1
0.8
0.6
0.4
0.2
0
200
400
600
800
1000
transmembrane
inside
outside

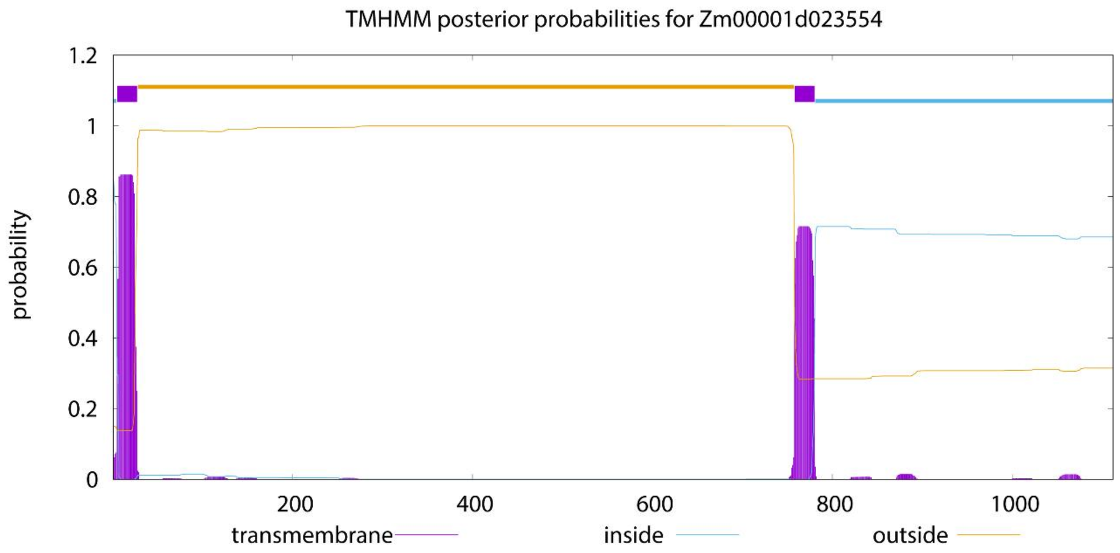

TMHMM posterior probabilities for Zm00001d023554
probability
1.2
1
0.8
0.6
0.4
0.2
0
200
400
600
800
1000
transmembrane
inside
outside

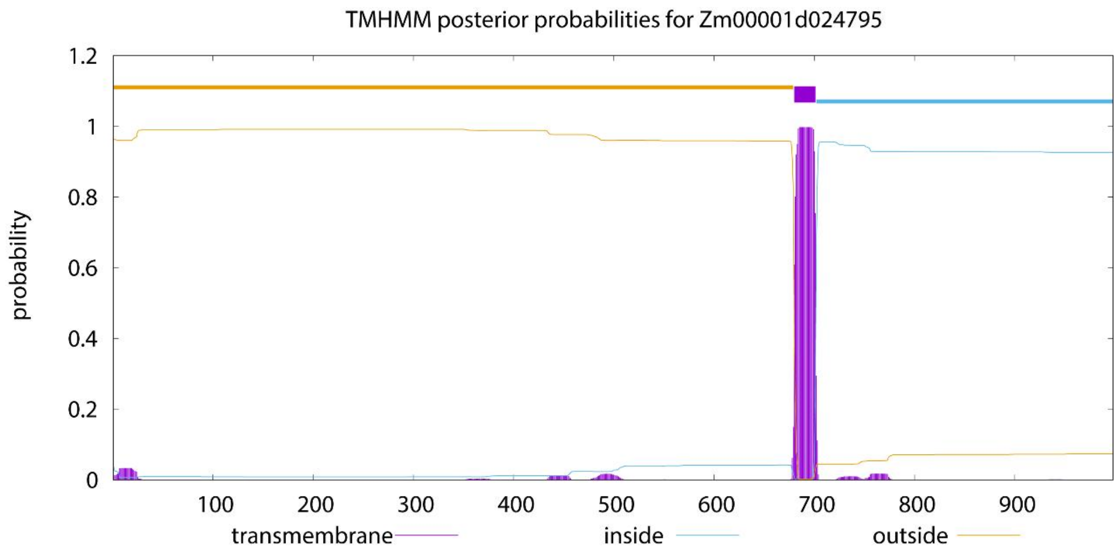

TMHMM posterior probabilities for Zm00001d024795
probability
1.2
1
0.8
0.6
0.4
0.2
0
100
200
300
400
500
600
700
800
900
transmembrane
inside
outside

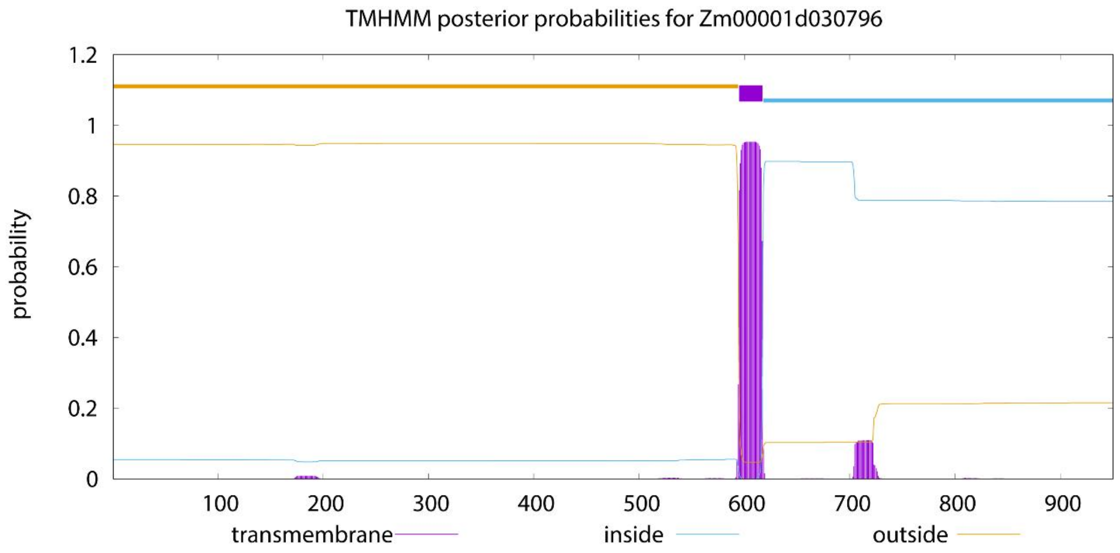
TMHMM posterior probabilities for Zm00001d030796
probability
1.2
1
0.8
0.6
0.4
0.2
0
100
200
300
400
500
600
700
800
900
transmembrane
inside
outside

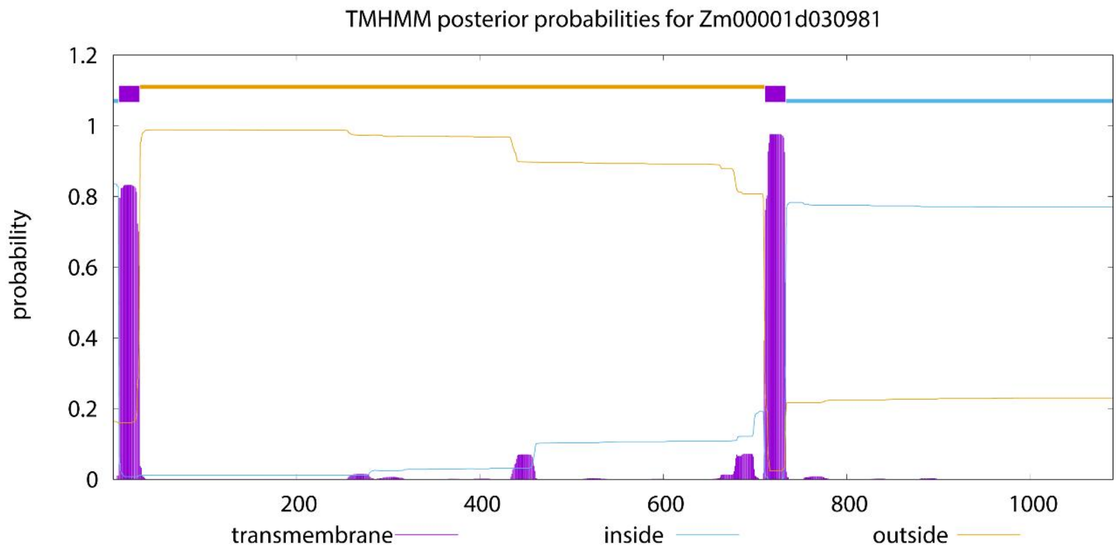
TMHMM posterior probabilities for Zm00001d030981
probability
1.2
1
0.8
0.6
0.4
0.2
0
200
400
600
800
1000
transmembrane
inside
outside

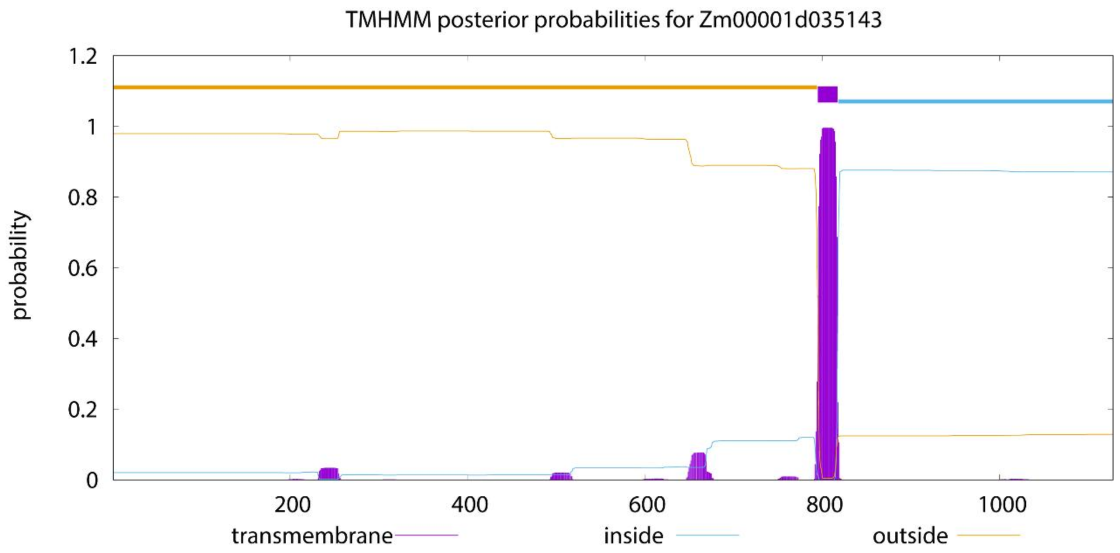
TMHMM posterior probabilities for Zm00001d035143
probability
1.2
1
0.8
0.6
0.4
0.2
0
200
400
600
800
1000
transmembrane
inside
outside

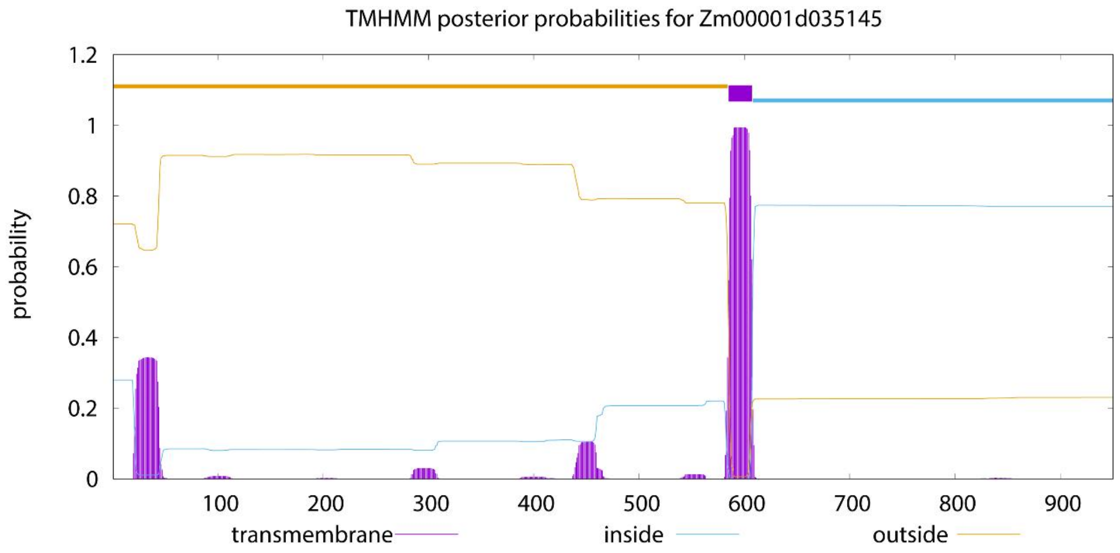

TMHMM posterior probabilities for Zm00001d035145
probability
1.2
1
0.8
0.6
0.4
0.2
0
100
200
300
400
500
600
700
800
900
transmembrane
inside
outside

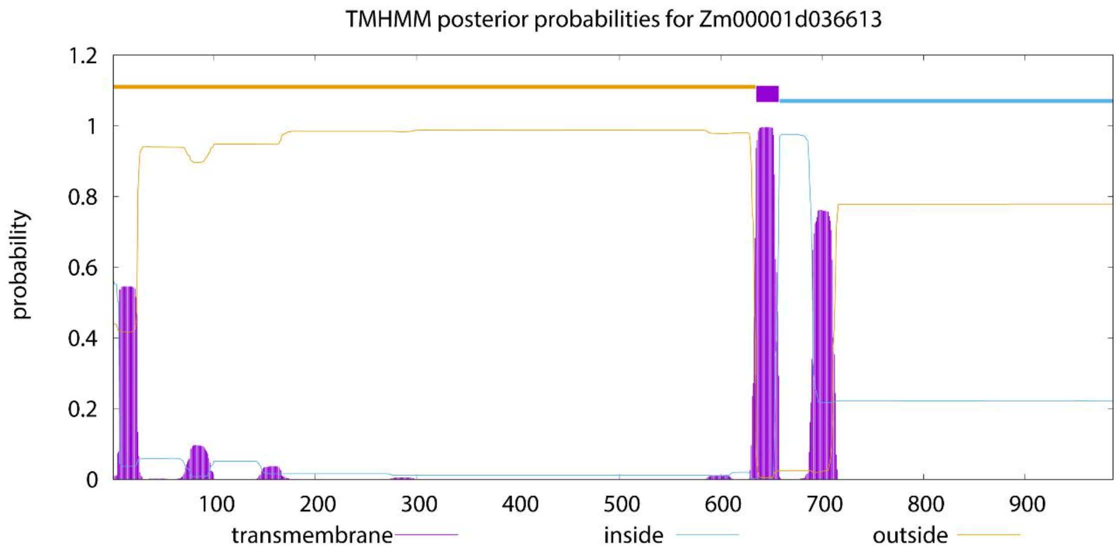

TMHMM posterior probabilities for Zm00001d036613
probability
1.2
1
0.8
0.6
0.4
0.2
0
100
200
300
400
500
600
700
800
900
transmembrane
inside
outside

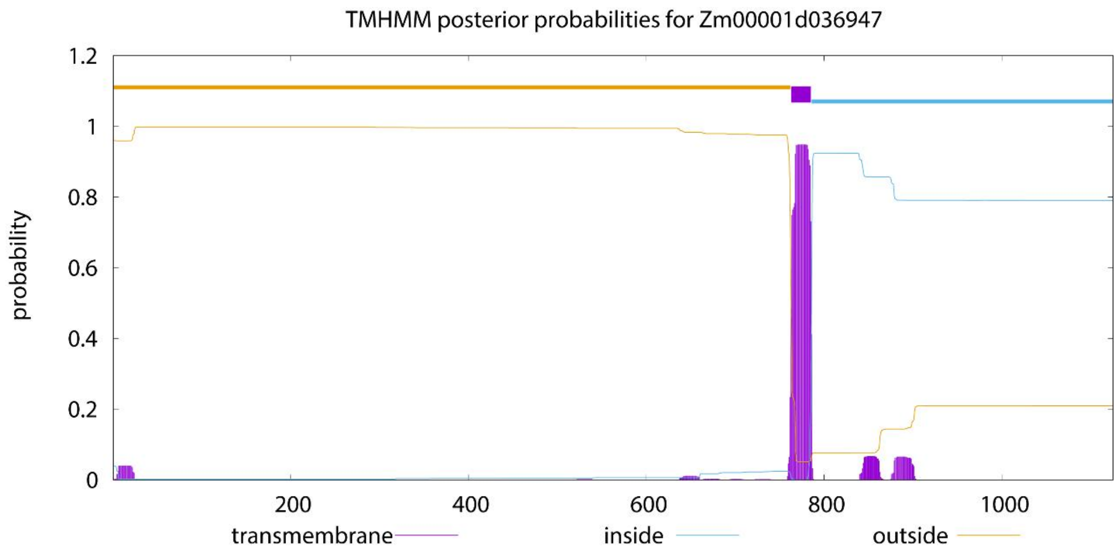

TMHMM posterior probabilities for Zm00001d036947
probability
1.2
1
0.8
0.6
0.4
0.2
0
200
400
600
800
1000
transmembrane
inside
outside

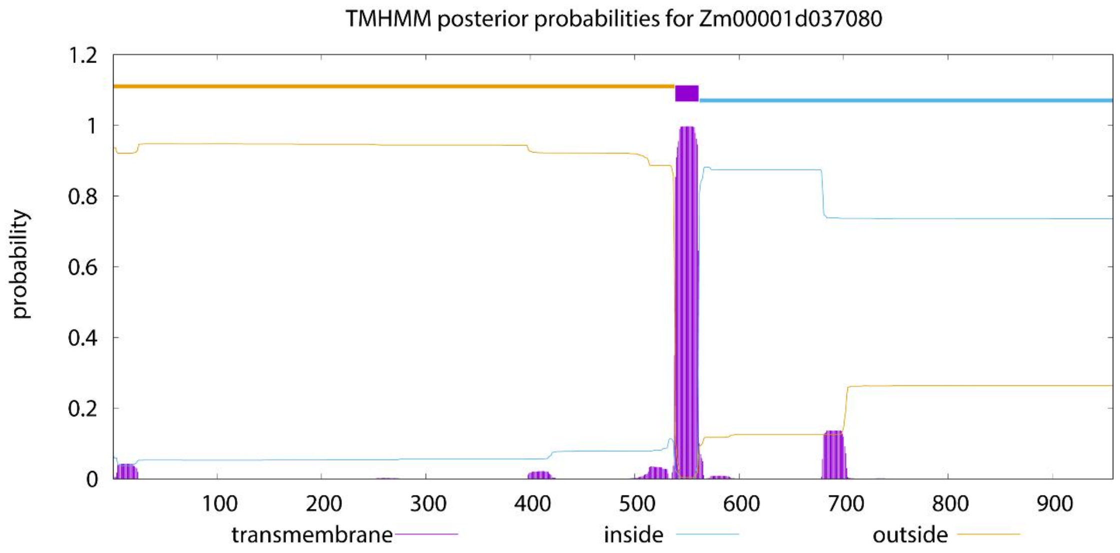
TMHMM posterior probabilities for Zm00001d037080
probability
1.2
1
0.8
0.6
0.4
0.2
0
100
200
300
400
500
600
700
800
900
transmembrane
inside
outside

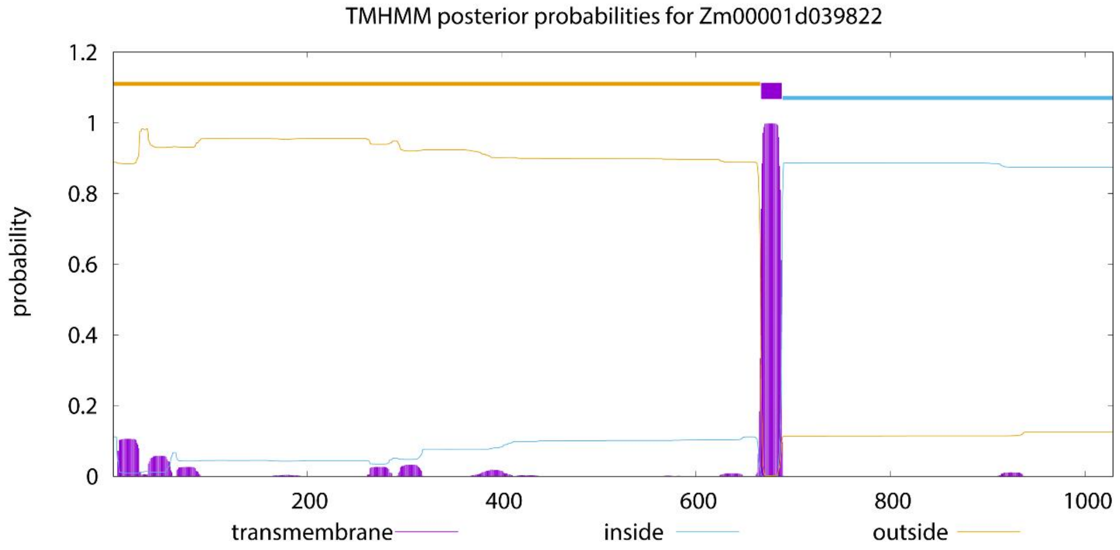
TMHMM posterior probabilities for Zm00001d039822
probability
1.2
1
0.8
0.6
0.4
0.2
0
200
400
600
800
1000
transmembrane
inside
outside

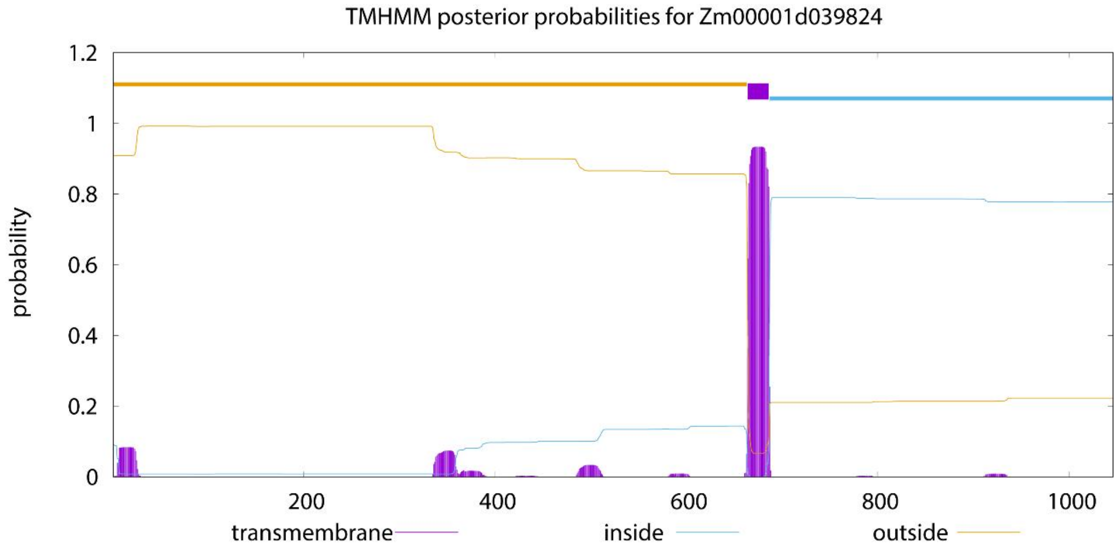
TMHMM posterior probabilities for Zm00001d039824
probability
1.2
1
0.8
0.6
0.4
0.2
0
200
400
600
800
1000
transmembrane
inside
outside

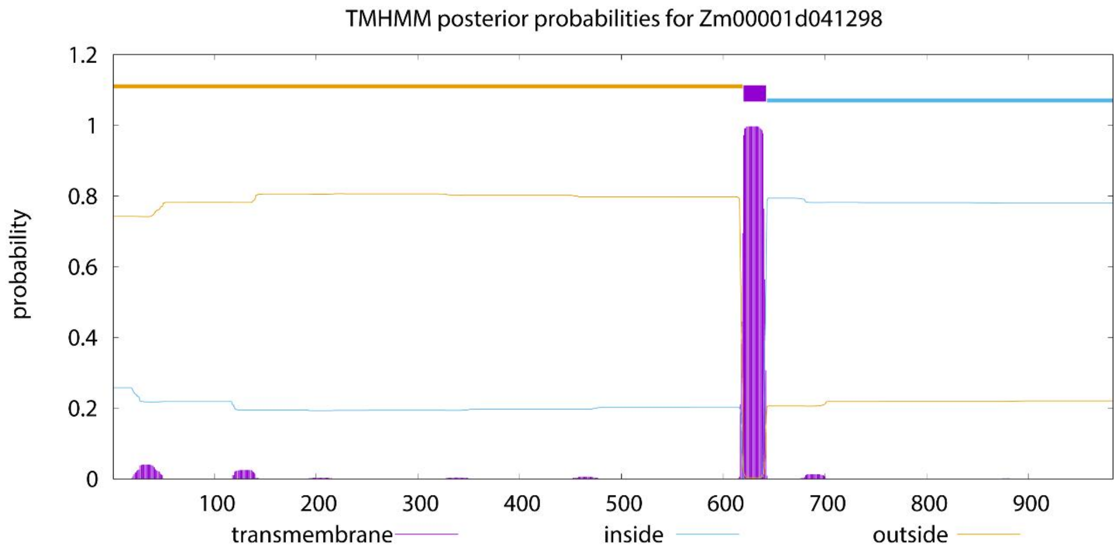
TMHMM posterior probabilities for Zm00001d041298
probability
1.2
1
0.8
0.6
0.4
0.2
0
100
200
300
400
500
600
700
800
900
transmembrane
inside
outside

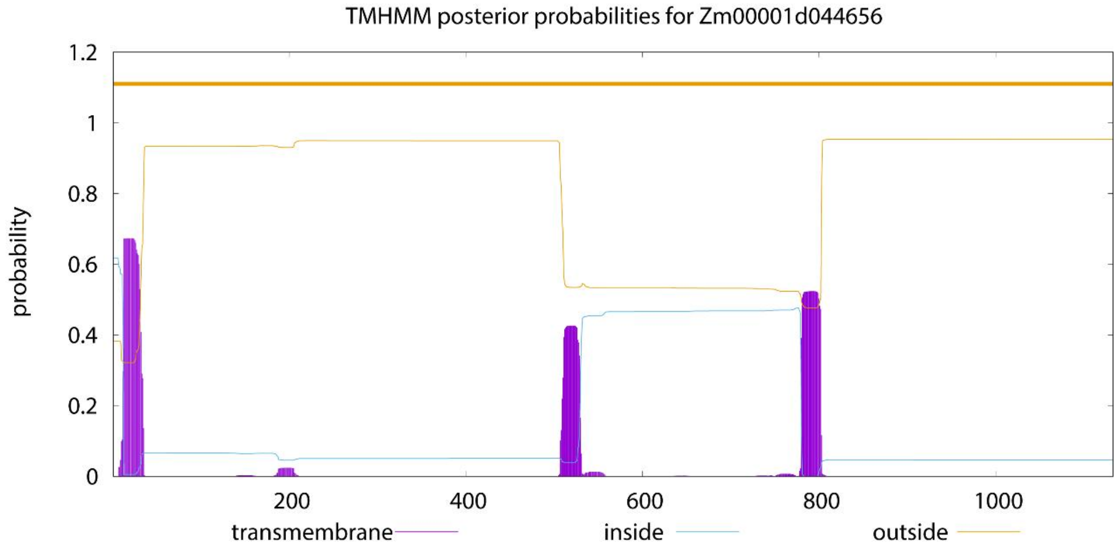
TMHMM posterior probabilities for Zm00001d044656
probability
1.2
1
0.8
0.6
0.4
0.2
0
200
400
600
800
1000
transmembrane
inside
outside

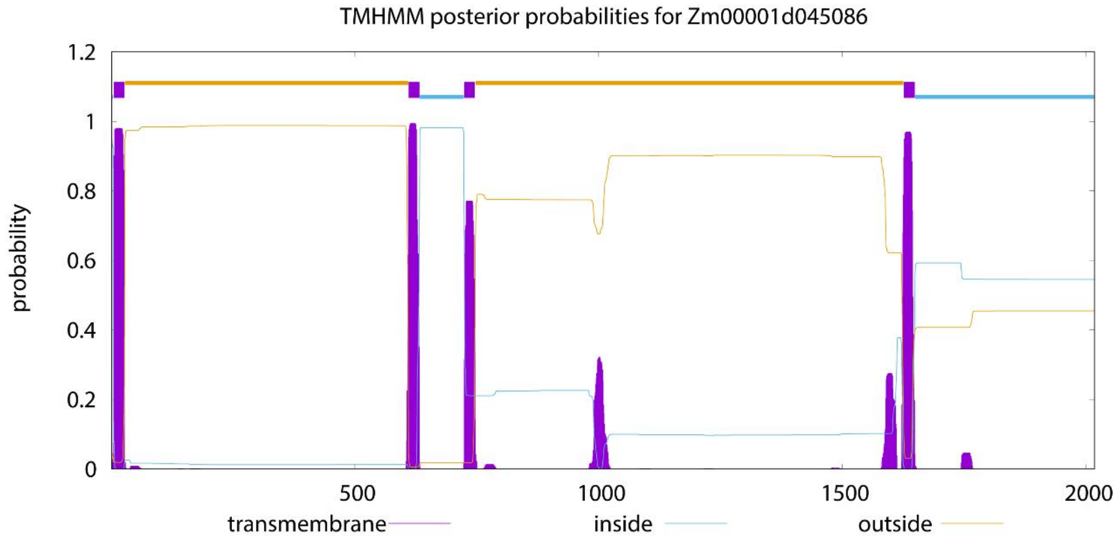
TMHMM posterior probabilities for Zm00001d045086
probability
1.2
1
0.8
0.6
0.4
0.2
0
500
1000
1500
2000
transmembrane
inside
outside

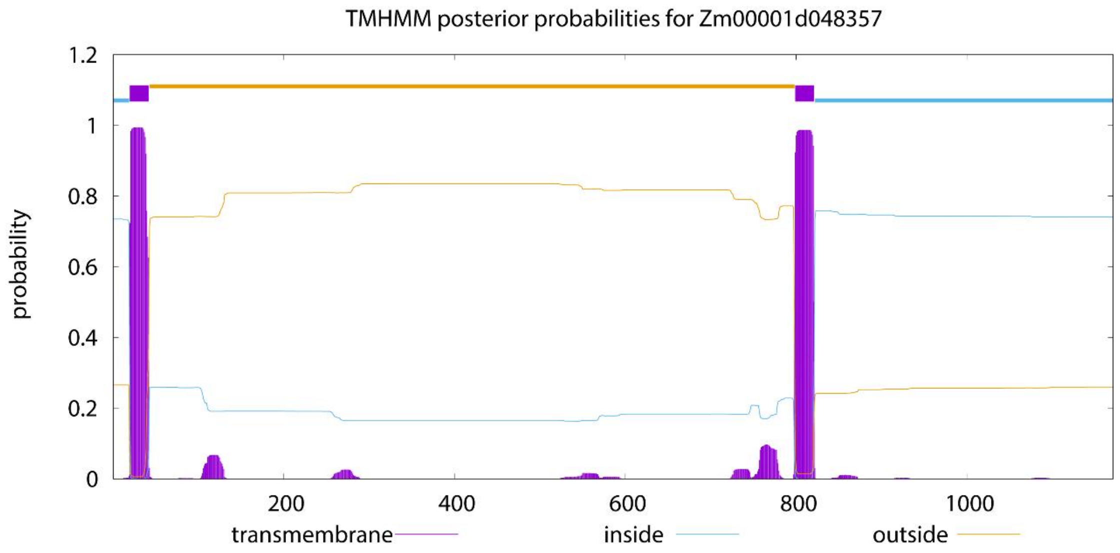
TMHMM posterior probabilities for Zm00001d048357
probability
1.2
1
0.8
0.6
0.4
0.2
0
200
400
600
800
1000
transmembrane
inside
outside

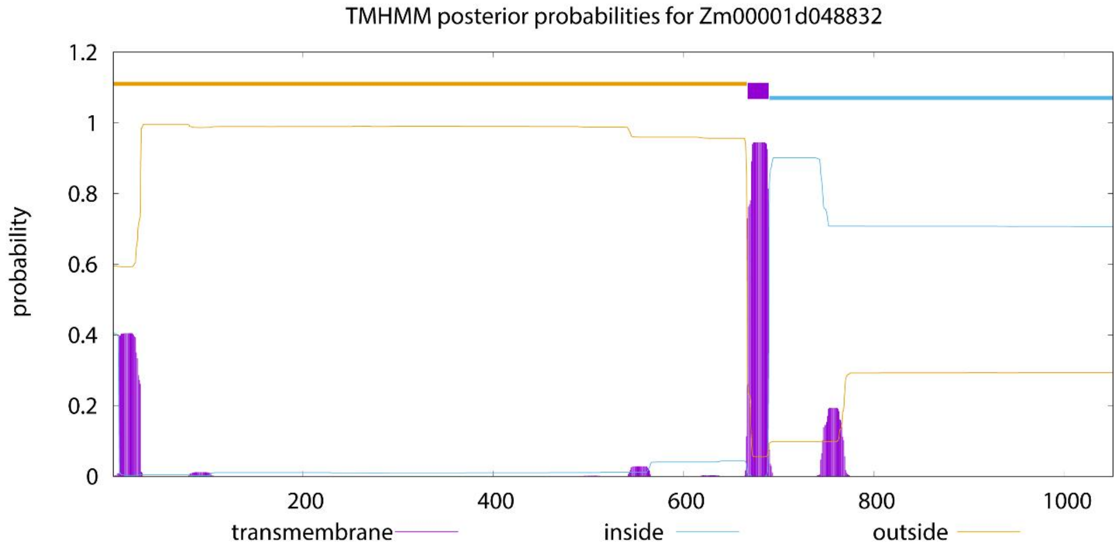
TMHMM posterior probabilities for Zm00001d048832
probability
1.2
1
0.8
0.6
0.4
0.2
0
200
400
600
800
1000
transmembrane
inside
outside

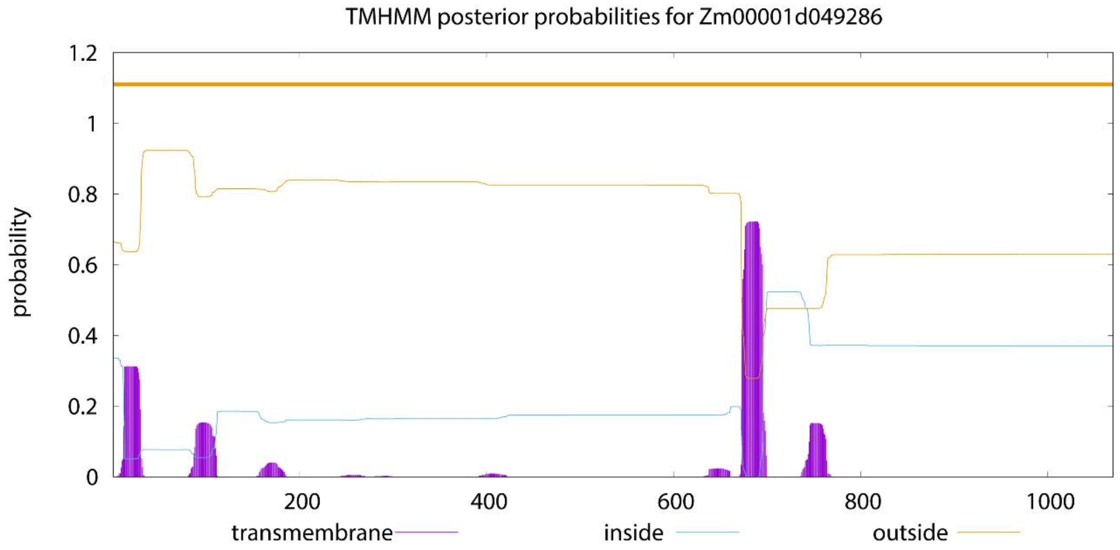
TMHMM posterior probabilities for Zm00001d049286
probability
1.2
1
0.8
0.6
0.4
0.2
0
200
400
600
800
1000
transmembrane
inside
outside

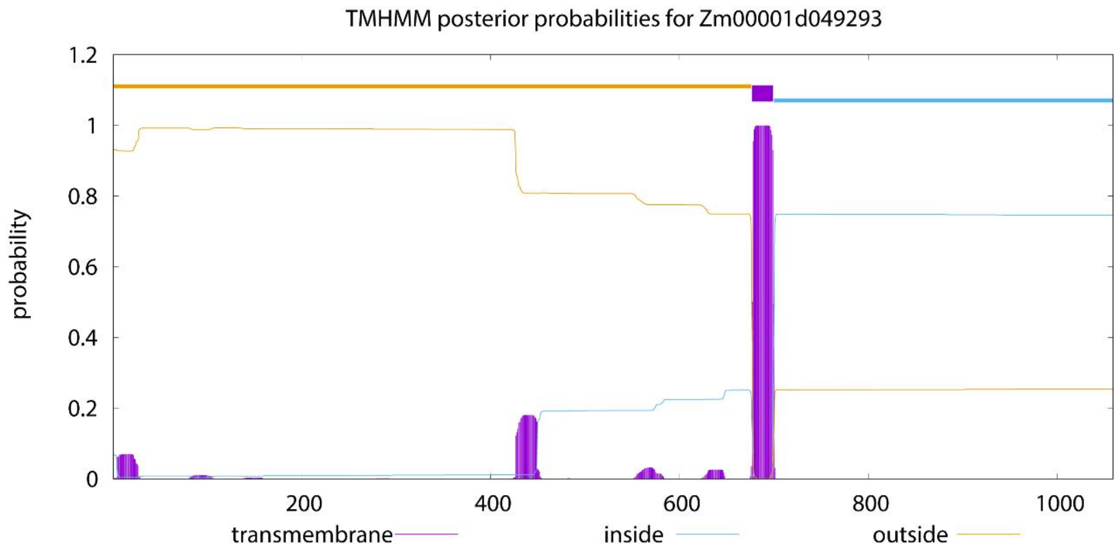
TMHMM posterior probabilities for Zm00001d049293
probability
1.2
1
0.8
0.6
0.4
0.2
0
200
400
600
800
1000
transmembrane
inside
outside

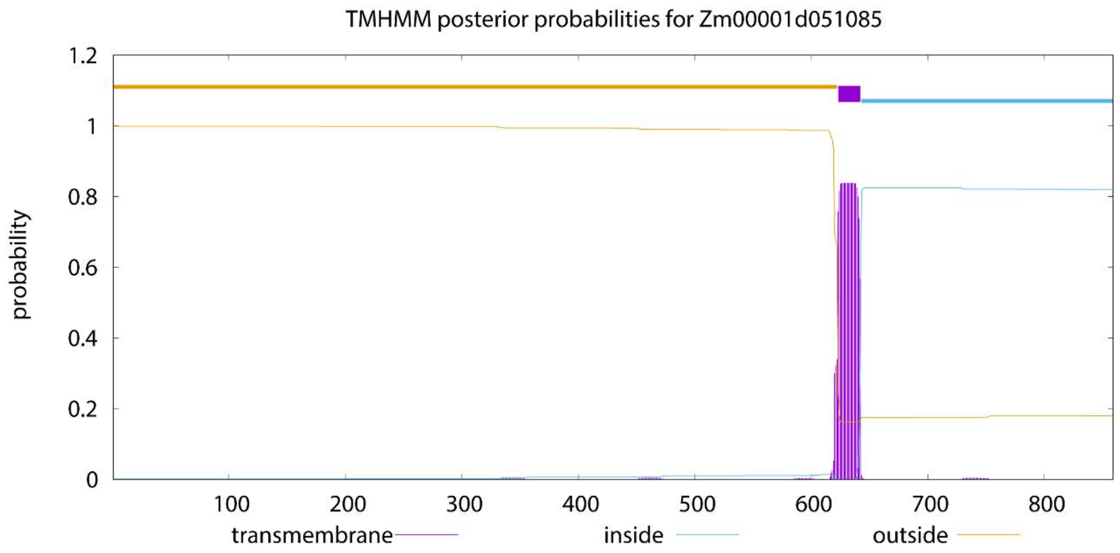
TMHMM posterior probabilities for Zm00001d051085
probability
1.2
1
0.8
0.6
0.4
0.2
0
100
200
300
400
500
600
700
800
transmembrane
inside
outside

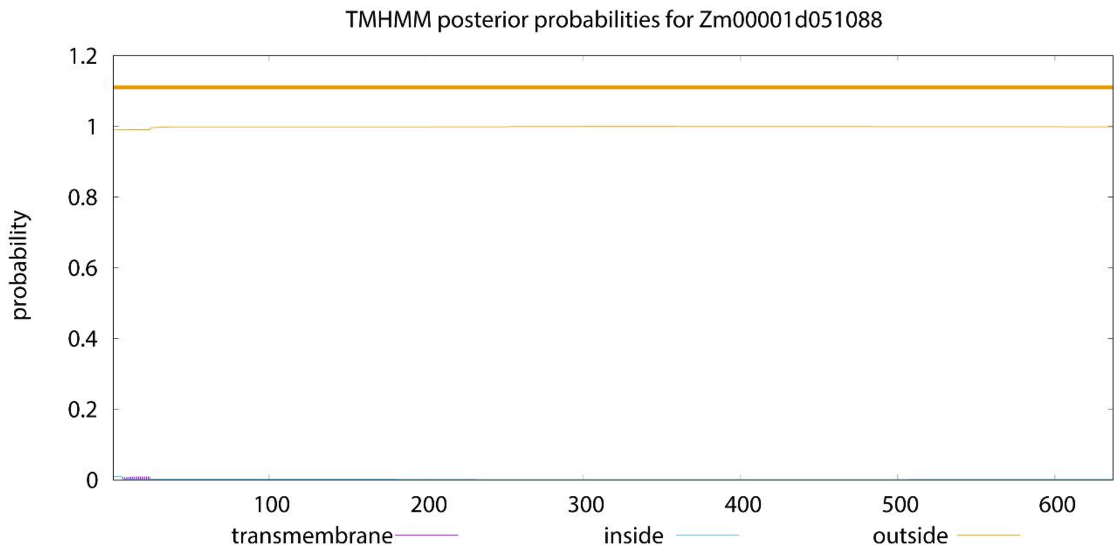
TMHMM posterior probabilities for Zm00001d051088
probability
1.2
1
0.8
0.6
0.4
0.2
0
100
200
300
400
500
600
transmembrane
inside
outside

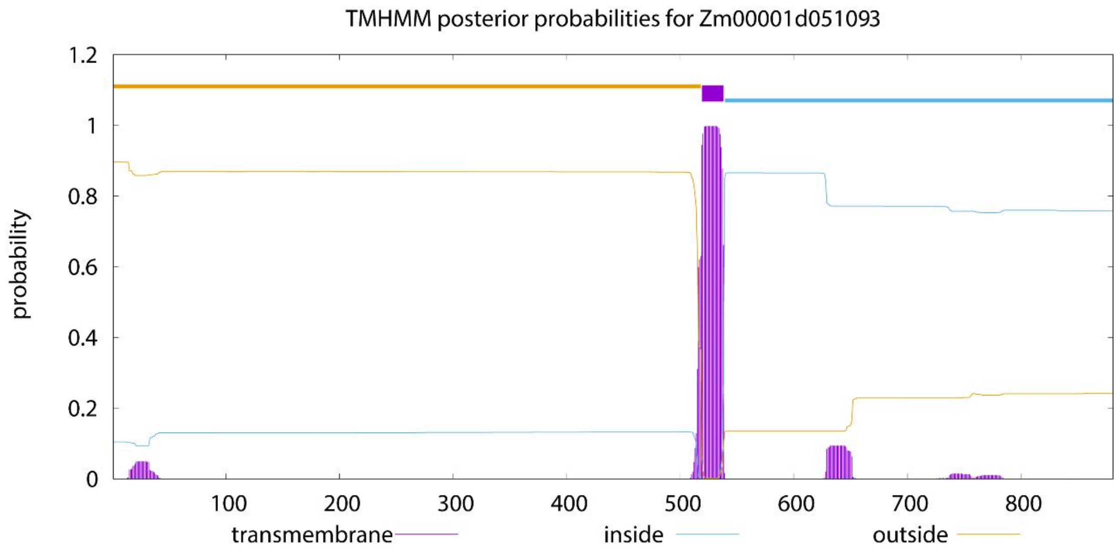
TMHMM posterior probabilities for Zm00001d051093
probability
1.2
1
0.8
0.6
0.4
0.2
0
100
200
300
400
500
600
700
800
transmembrane
inside
outside

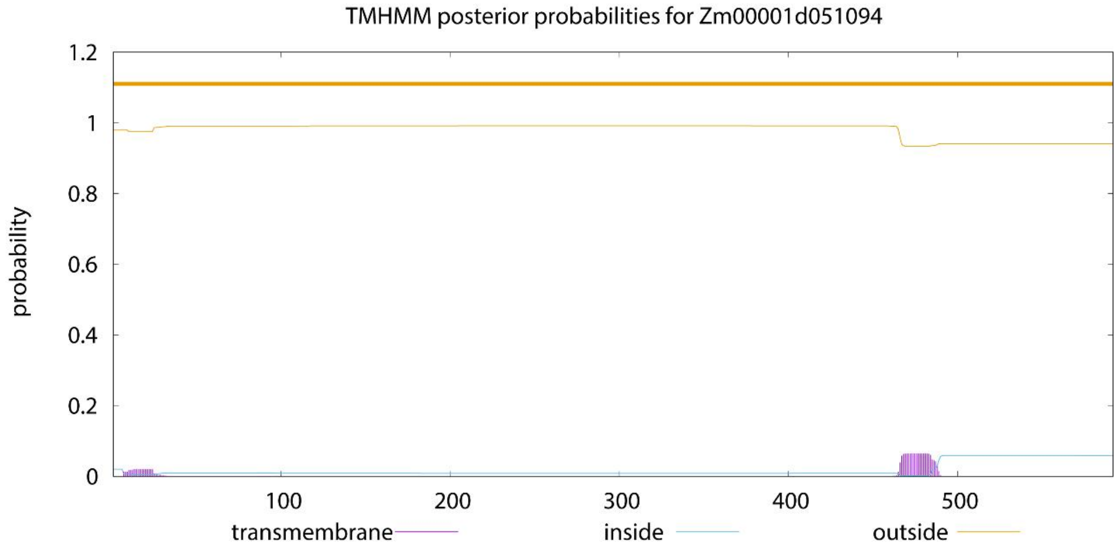
TMHMM posterior probabilities for Zm00001d051094
probability
1.2
1
0.8
0.6
0.4
0.2
0
100
200
300
400
500
transmembrane
inside
outside

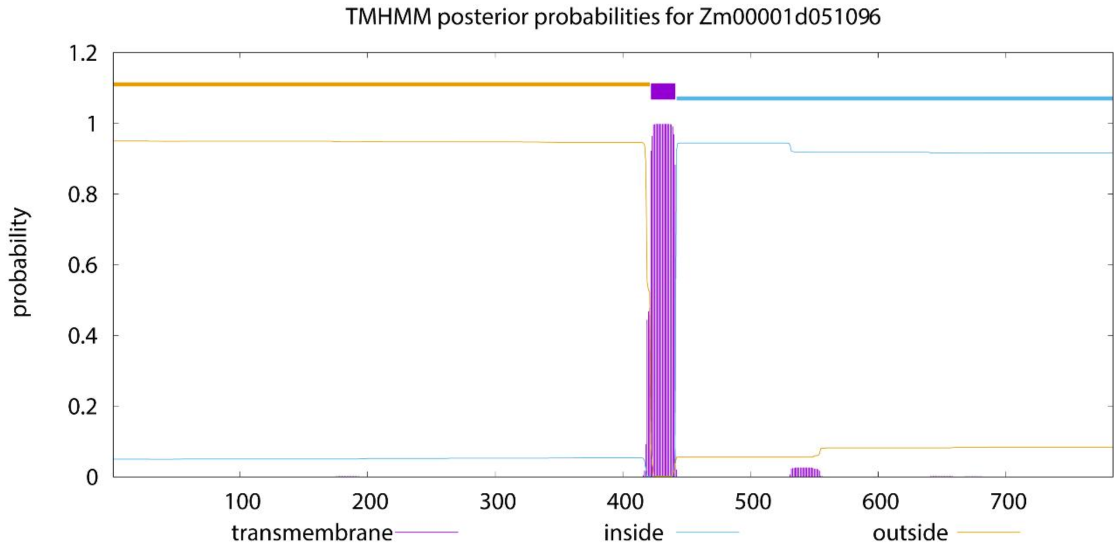
TMHMM posterior probabilities for Zm00001d051096
probability
1.2
1
0.8
0.6
0.4
0.2
0
100
200
300
400
500
600
700
transmembrane
inside
outside

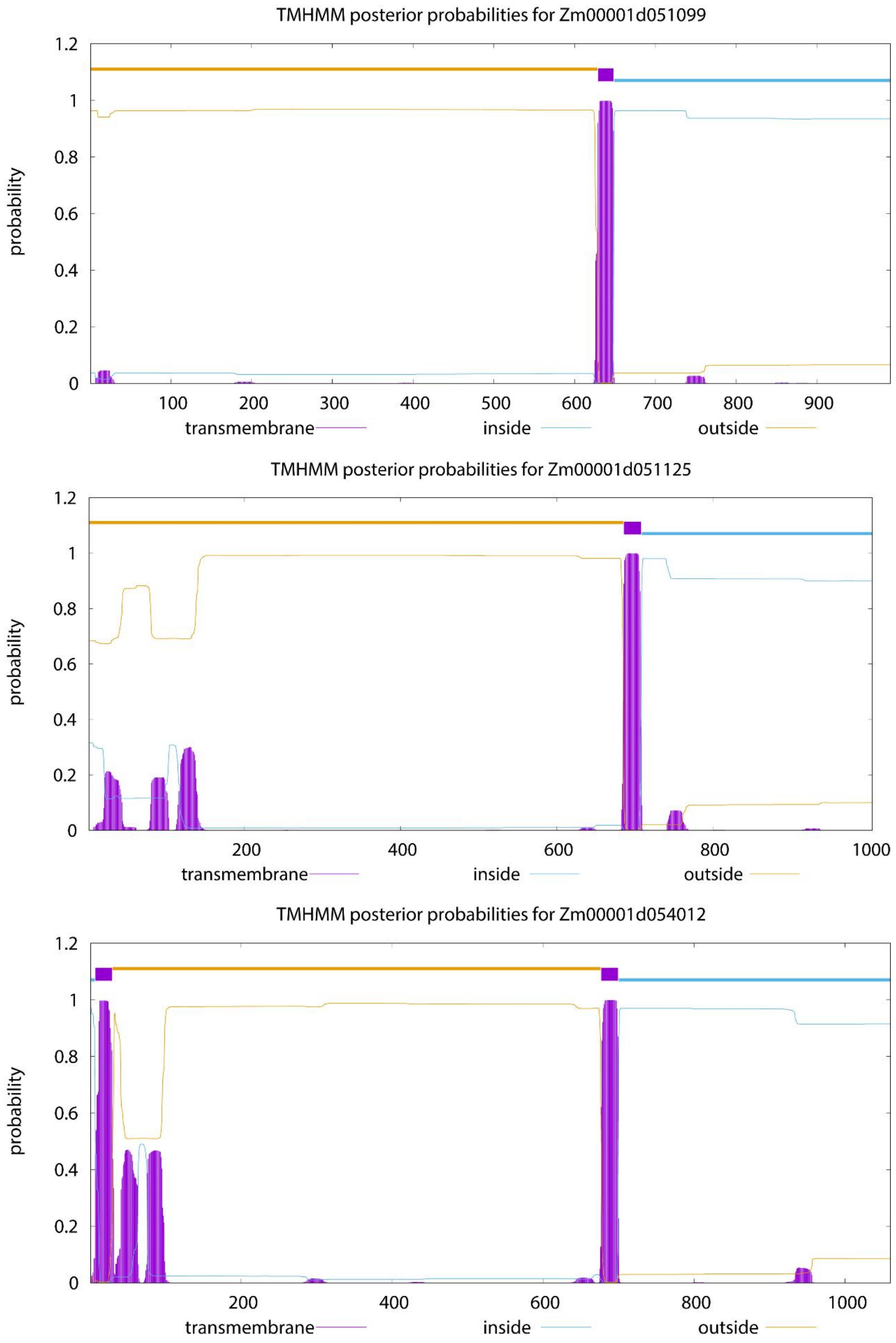

Transmembrane structure domain of subfamily XII.

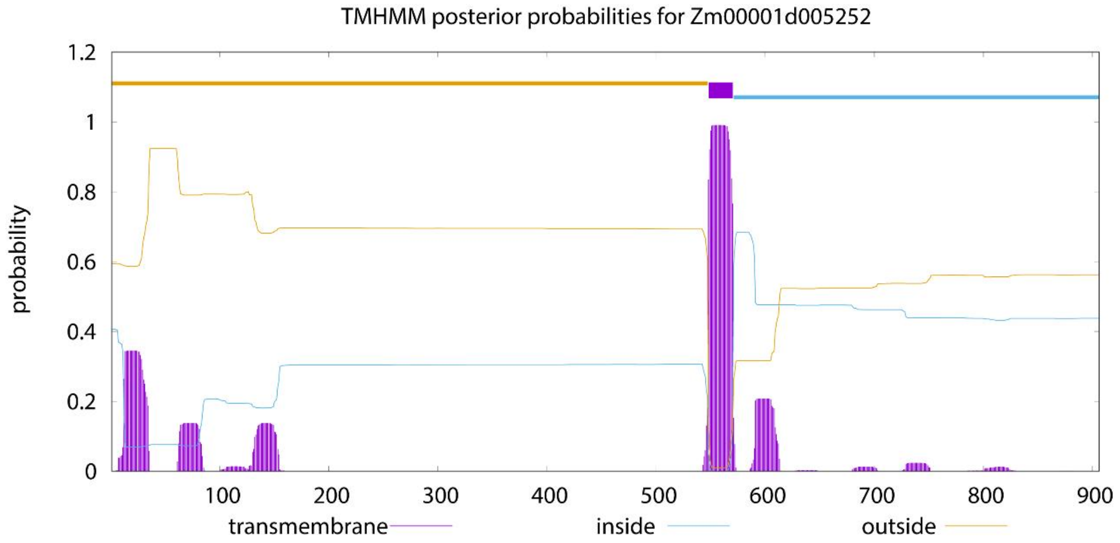

TMHMM posterior probabilities for Zm00001d005252
probability
1.2
1
0.8
0.6
0.4
0.2
0
100
200
300
400
500
600
700
800
900
transmembrane
inside
outside

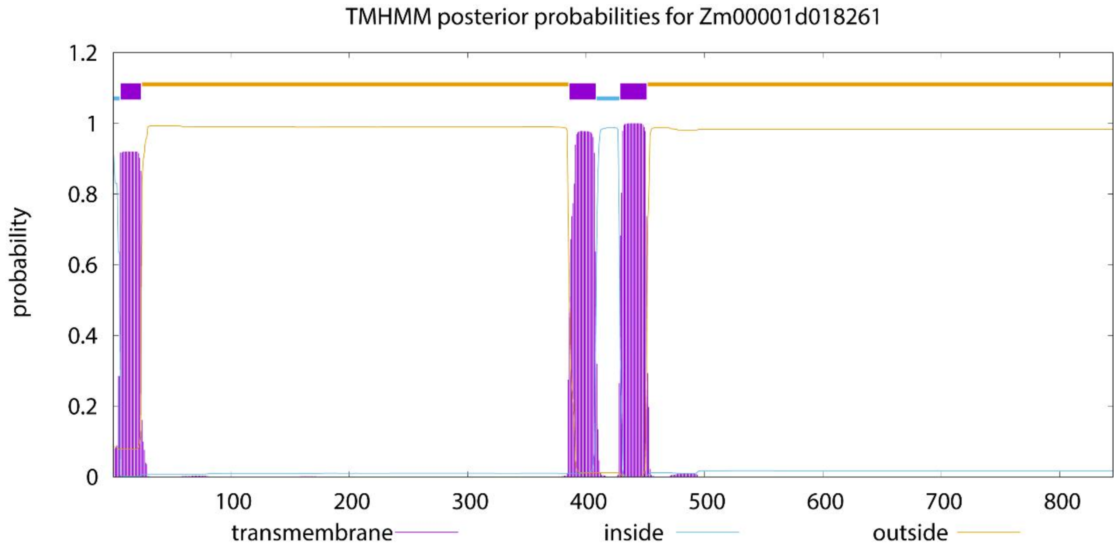

TMHMM posterior probabilities for Zm00001d018261
probability
1.2
1
0.8
0.6
0.4
0.2
0
100
200
300
400
500
600
700
800
transmembrane
inside
outside

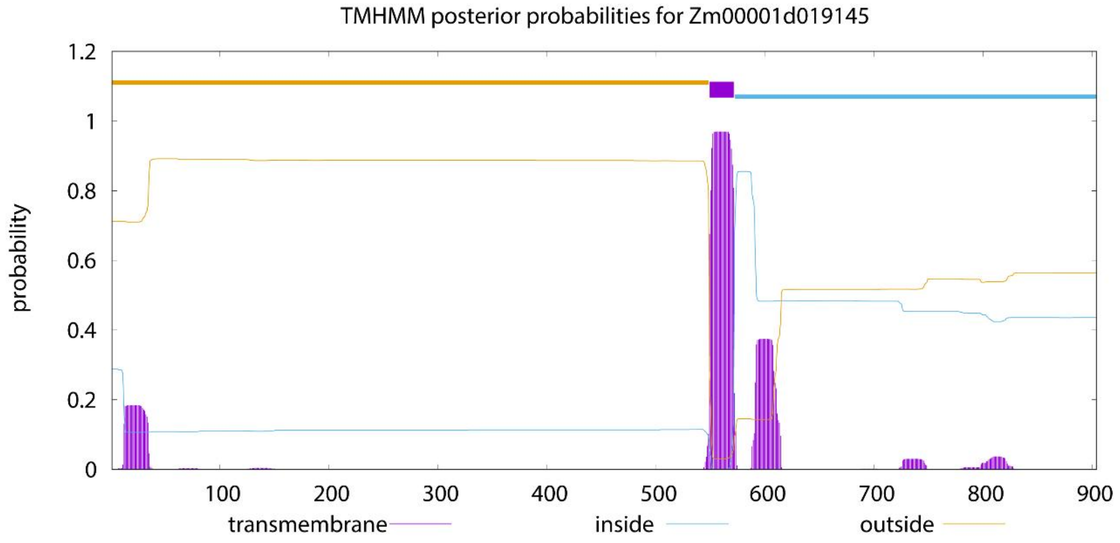

TMHMM posterior probabilities for Zm00001d019145
probability
1.2
1
0.8
0.6
0.4
0.2
0
100
200
300
400
500
600
700
800
900
transmembrane
inside
outside

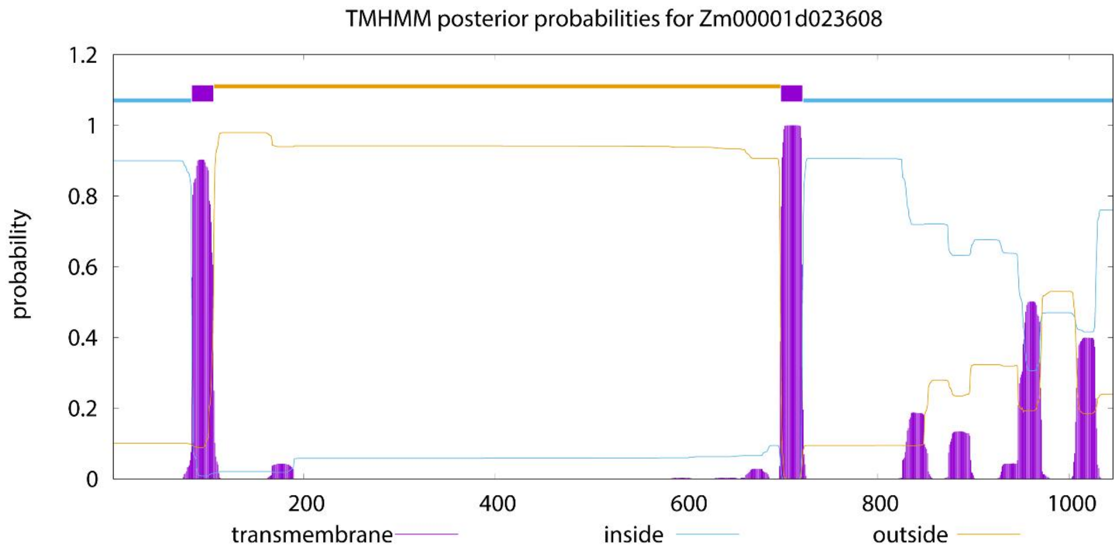
TMHMM posterior probabilities for Zm00001d023608
probability
1.2
1
0.8
0.6
0.4
0.2
0
200
400
600
800
1000
transmembrane
inside
outside

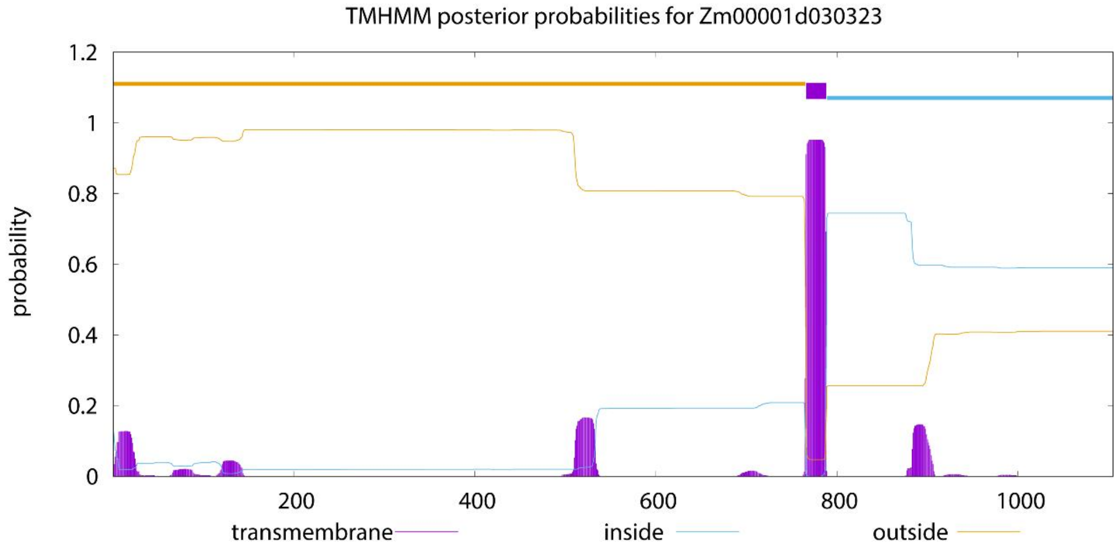
TMHMM posterior probabilities for Zm00001d030323
probability
1.2
1
0.8
0.6
0.4
0.2
0
200
400
600
800
1000
transmembrane
inside
outside

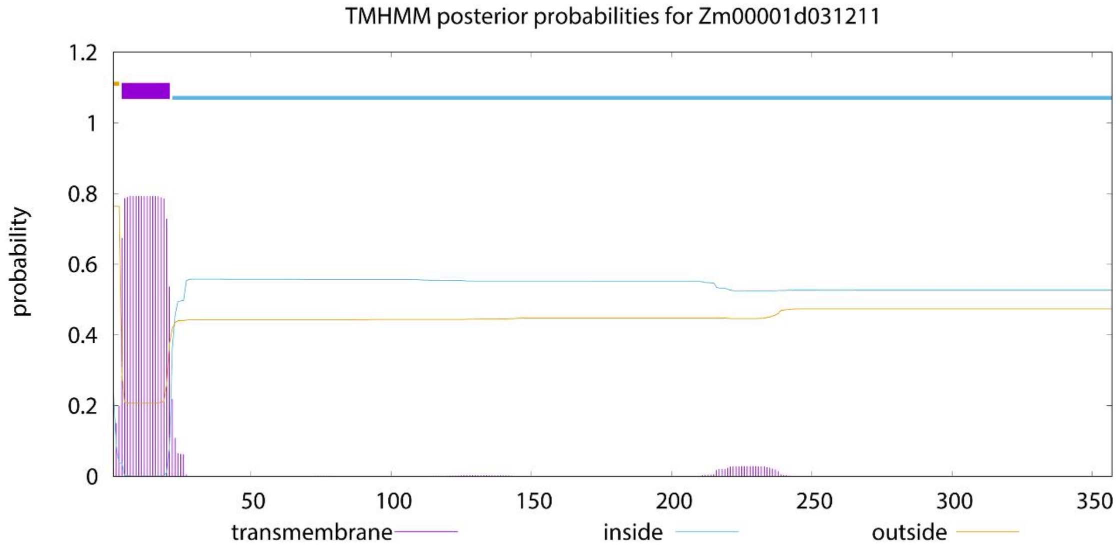
TMHMM posterior probabilities for Zm00001d031211
probability
1.2
1
0.8
0.6
0.4
0.2
0
50
100
150
200
250
300
350
transmembrane
inside
outside

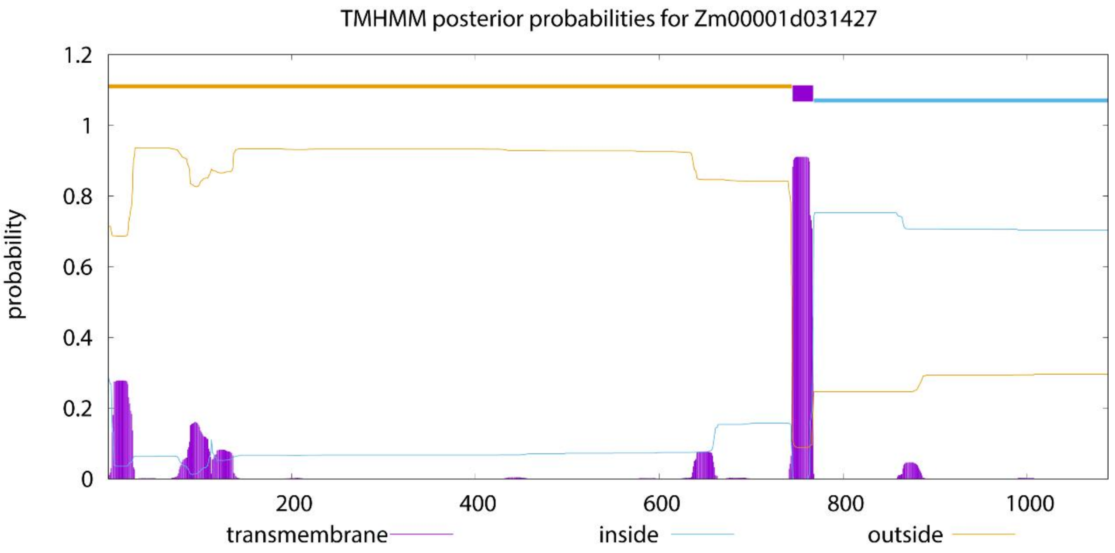
TMHMM posterior probabilities for Zm00001d031427
probability
1.2
1
0.8
0.6
0.4
0.2
0
200
400
600
800
1000
transmembrane
inside
outside

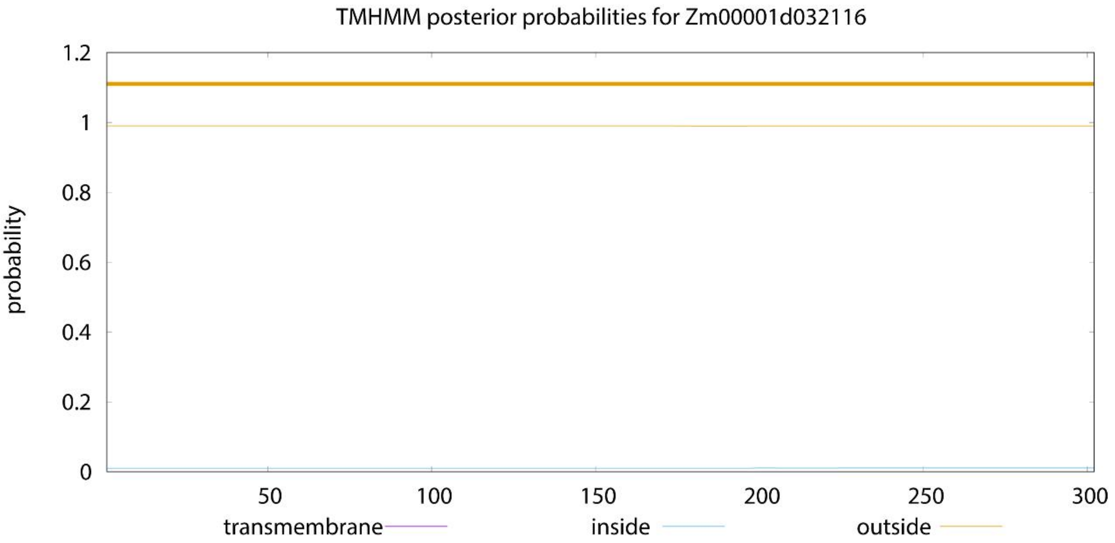
TMHMM posterior probabilities for Zm00001d032116
probability
1.2
1
0.8
0.6
0.4
0.2
0
50
100
150
200
250
300
transmembrane
inside
outside

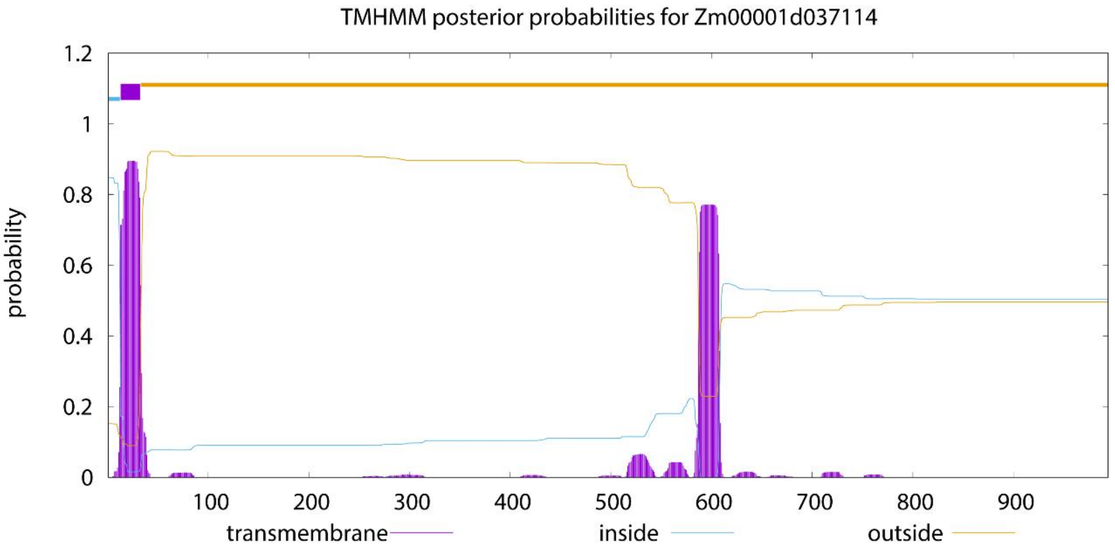
TMHMM posterior probabilities for Zm00001d037114
probability
1.2
1
0.8
0.6
0.4
0.2
0
100
200
300
400
500
600
700
800
900
transmembrane
inside
outside

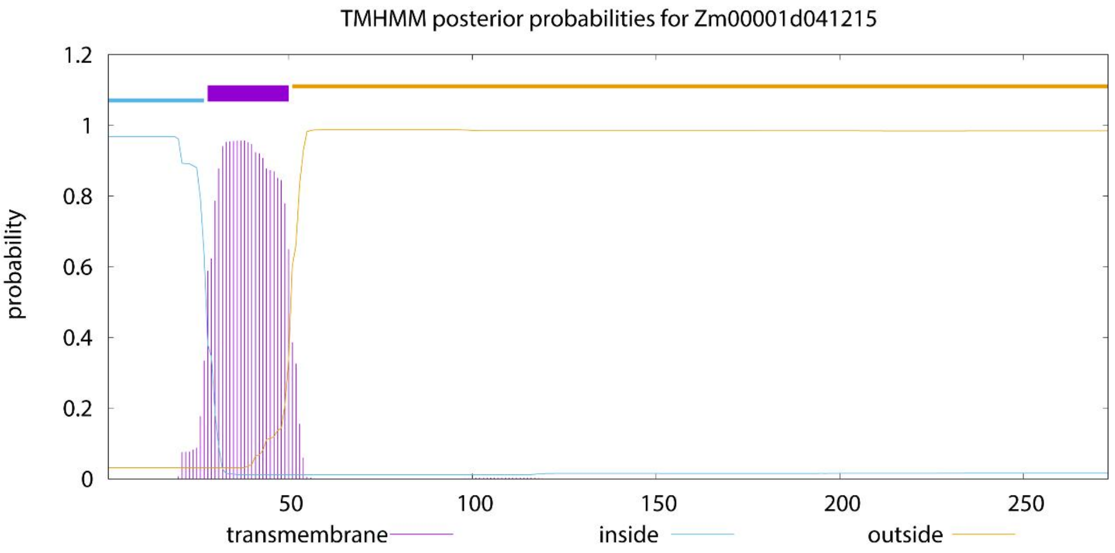

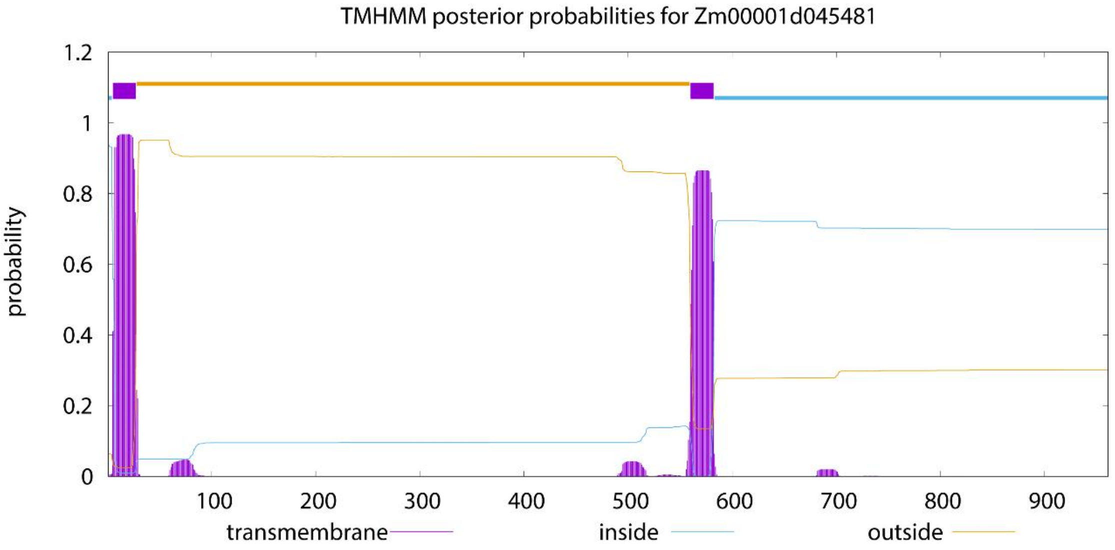

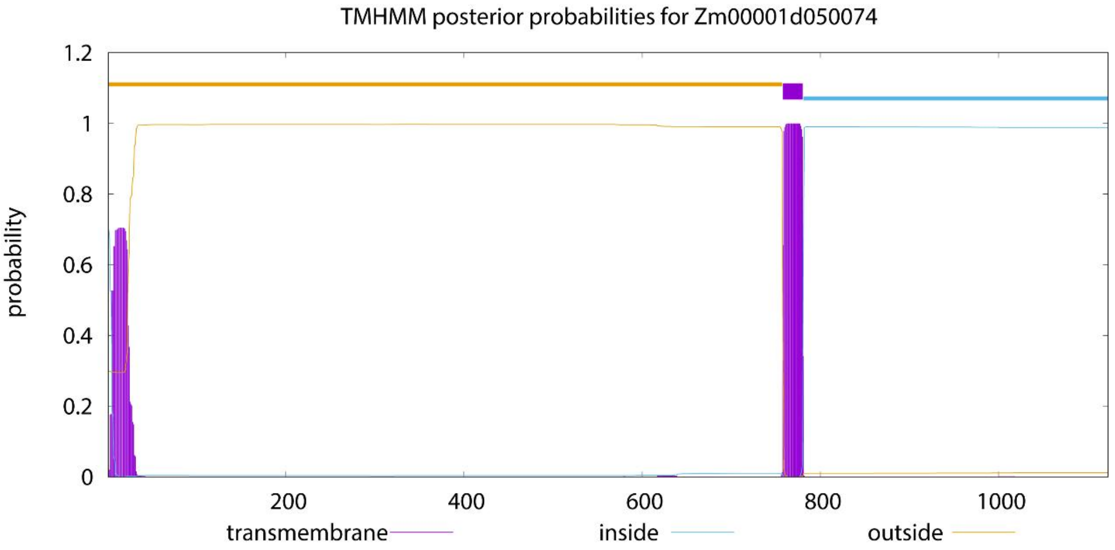

Transmembrane structure domain of subfamily XIII.

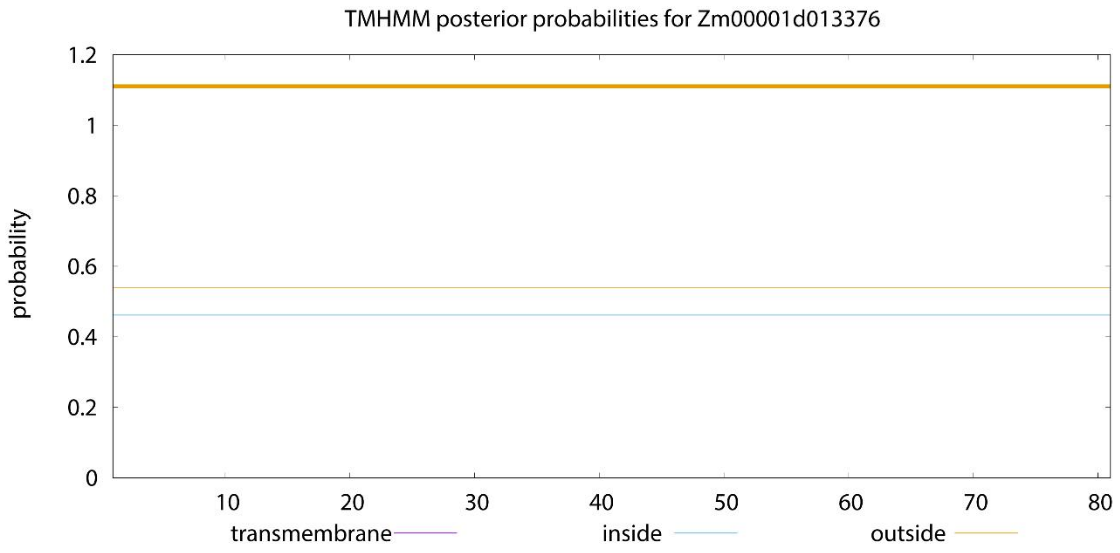

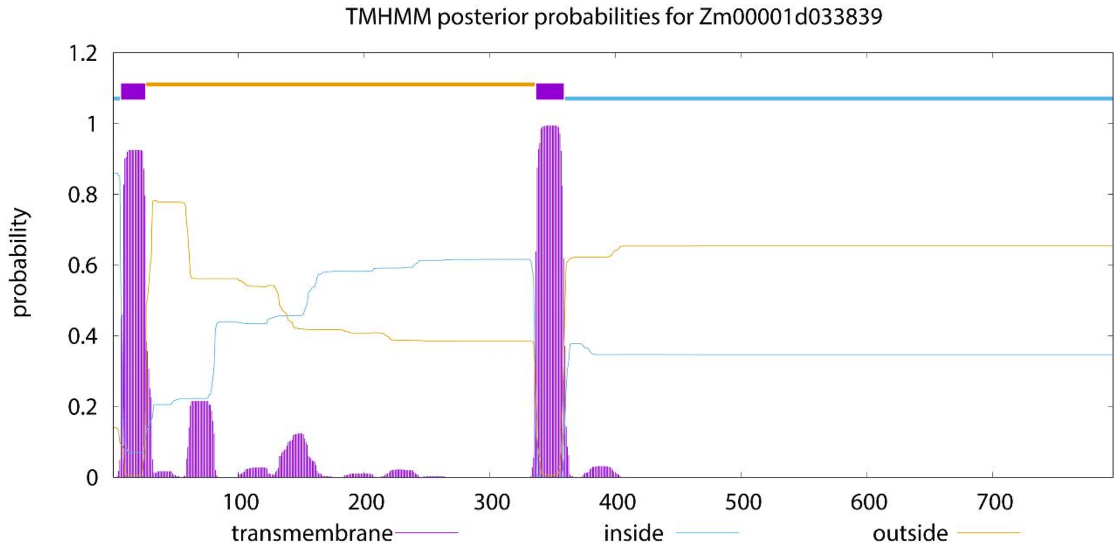

Transmembrane structure domain of subfamily XIV.

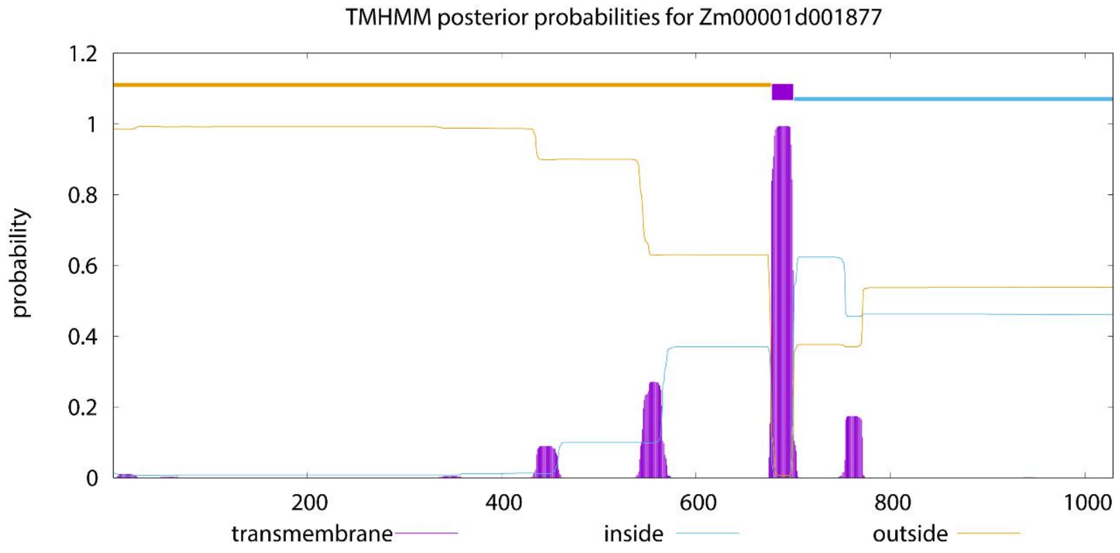

TMHMM posterior probabilities for Zm00001d013251

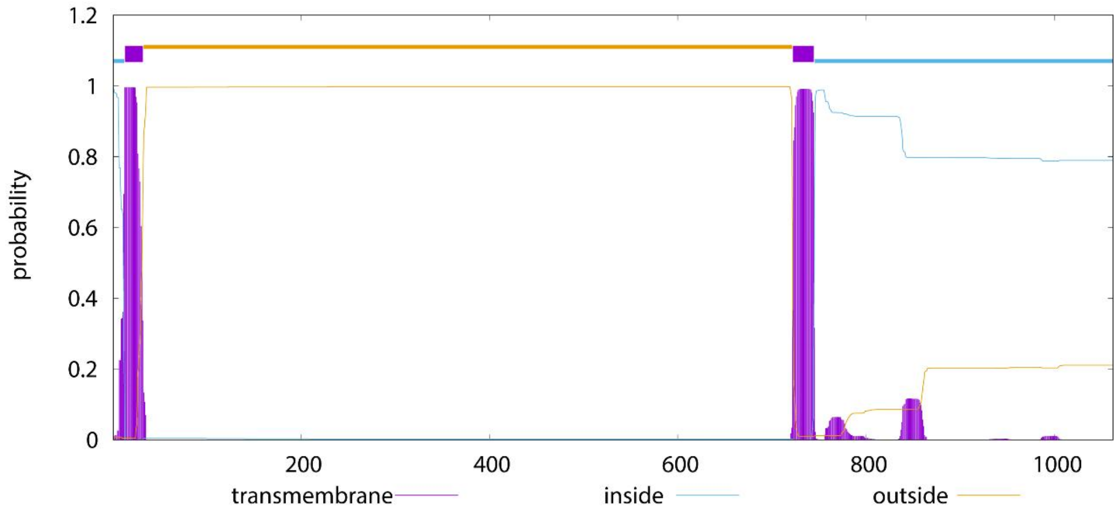

TMHMM posterior probabilities for Zm00001d014555

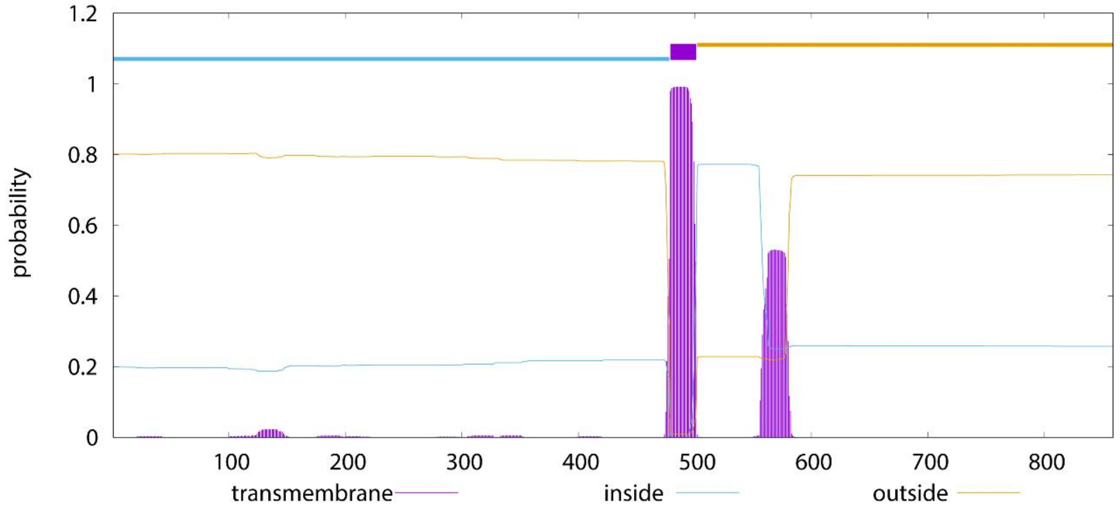

TMHMM posterior probabilities for Zm00001d014557

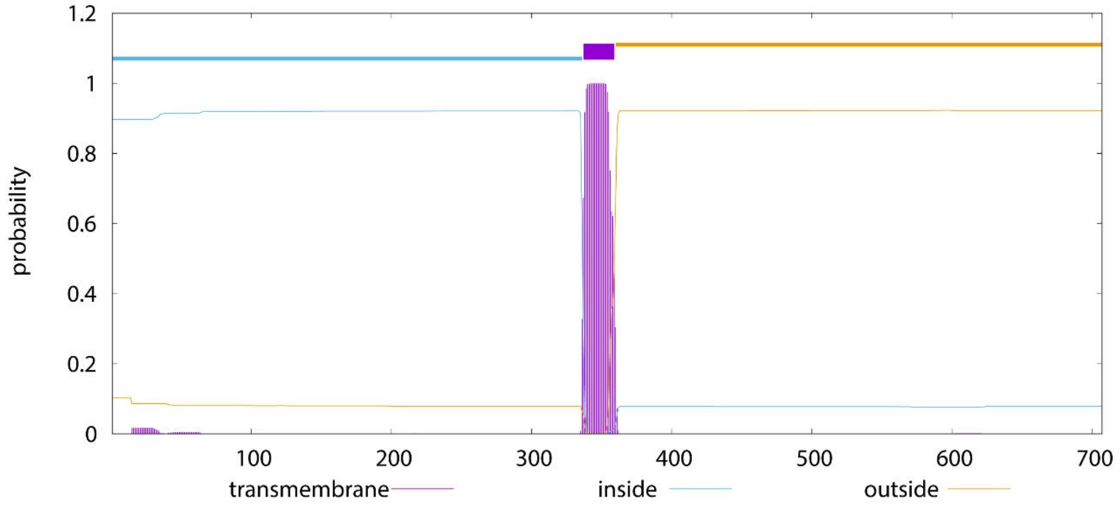

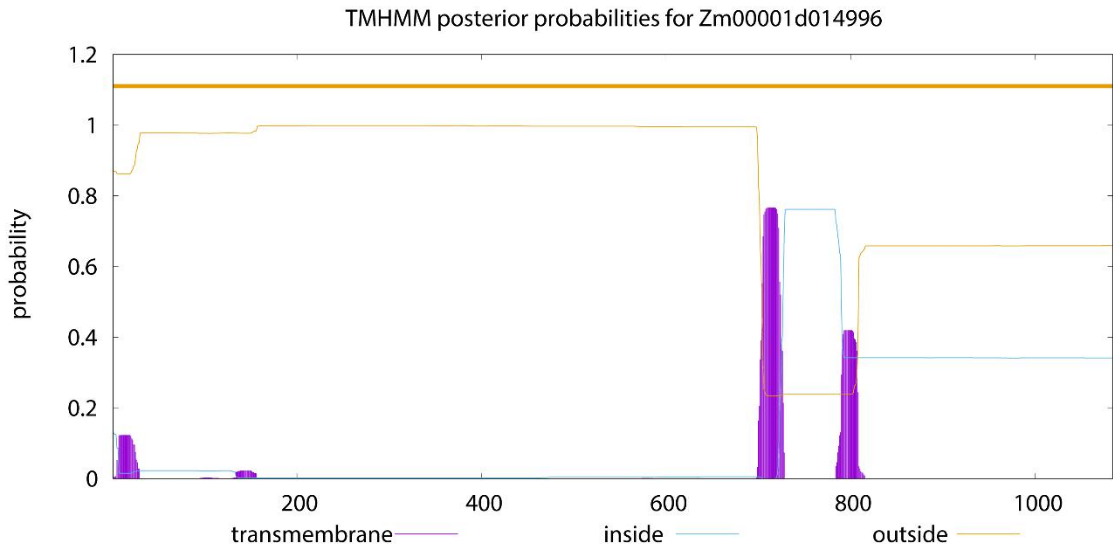

TMHMM posterior probabilities for Zm00001d014996
probability
1.2
1
0.8
0.6
0.4
0.2
0
200
400
600
800
1000
transmembrane
inside
outside

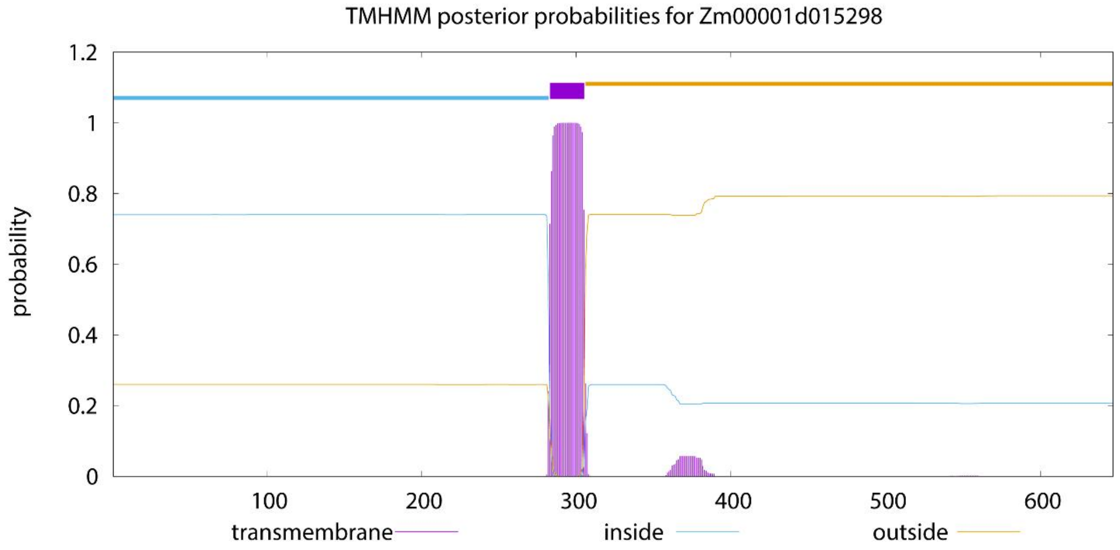

TMHMM posterior probabilities for Zm00001d015298
probability
1.2
1
0.8
0.6
0.4
0.2
0
100
200
300
400
500
600
transmembrane
inside
outside

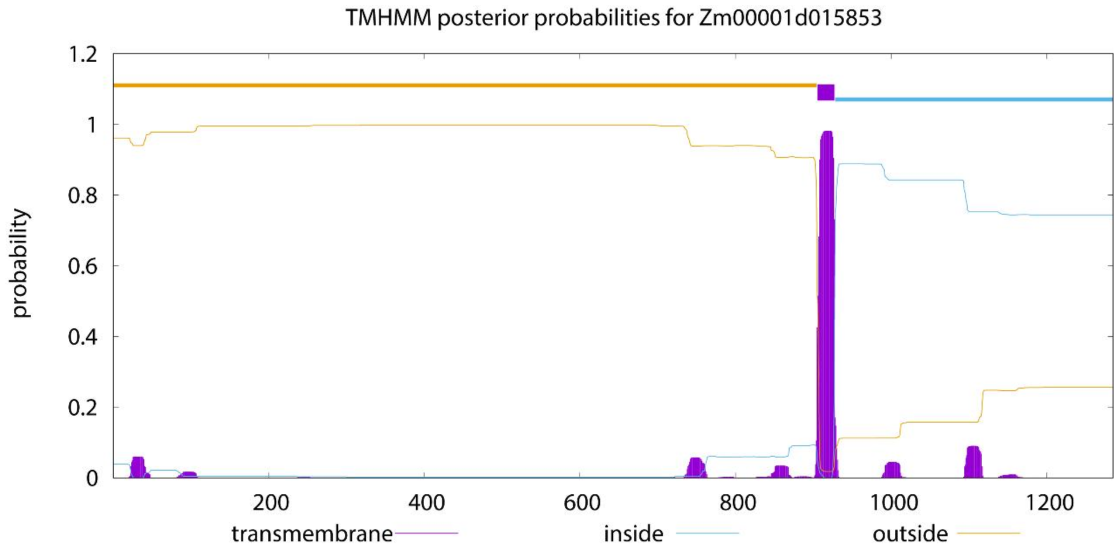

TMHMM posterior probabilities for Zm00001d015853
probability
1.2
1
0.8
0.6
0.4
0.2
0
200
400
600
800
1000
1200
transmembrane
inside
outside

TMHMM posterior probabilities for Zm00001d017291

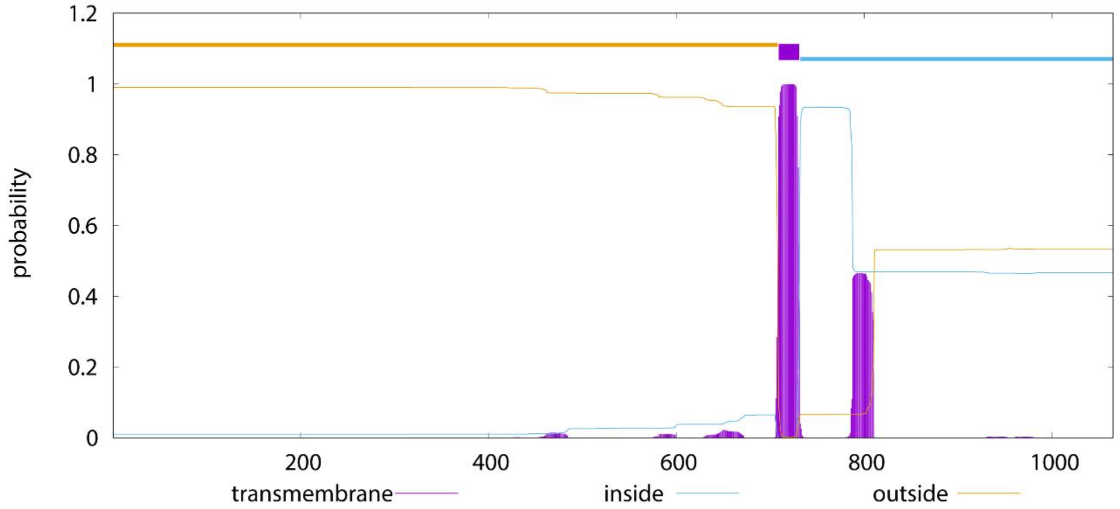

TMHMM posterior probabilities for Zm00001d018635

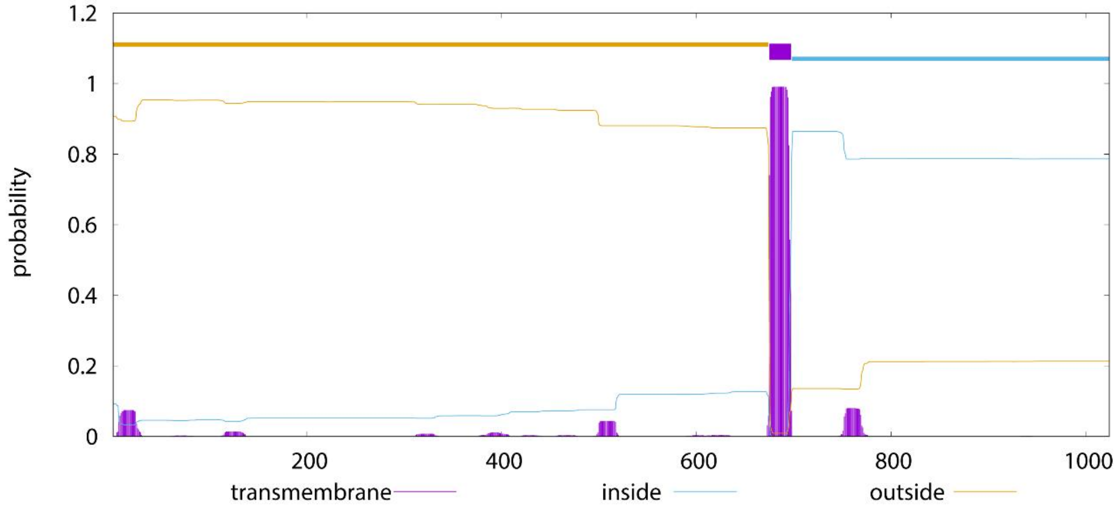

TMHMM posterior probabilities for Zm00001d018665

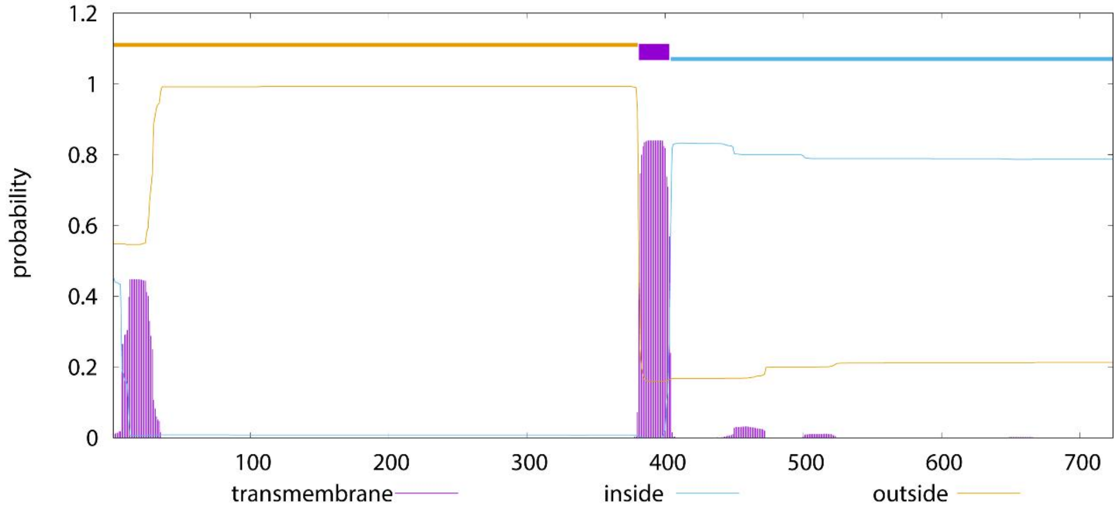

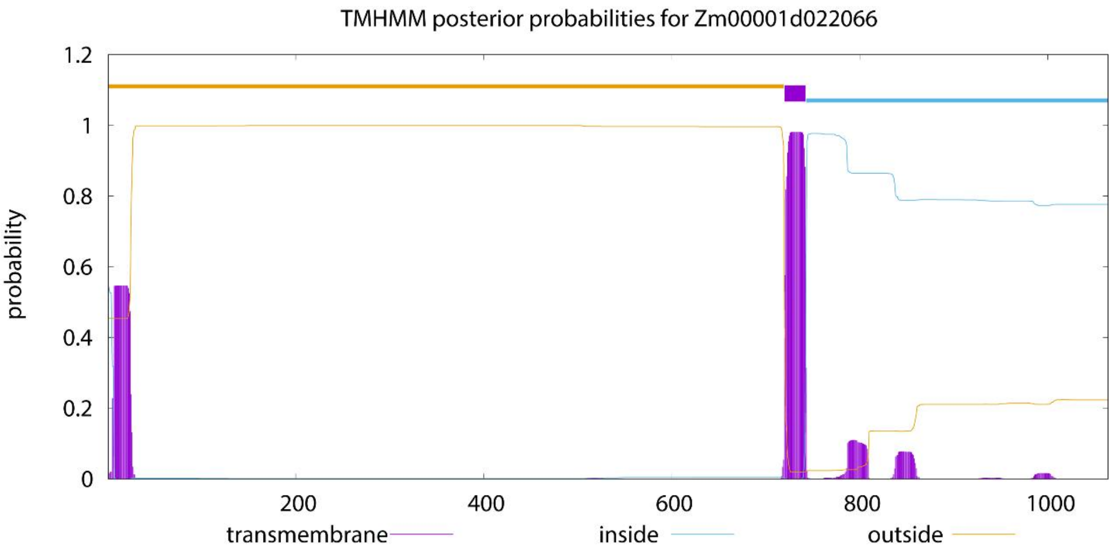
TMHMM posterior probabilities for Zm00001d022066
probability
1.2
1
0.8
0.6
0.4
0.2
0
200
400
600
800
1000
transmembrane
inside
outside

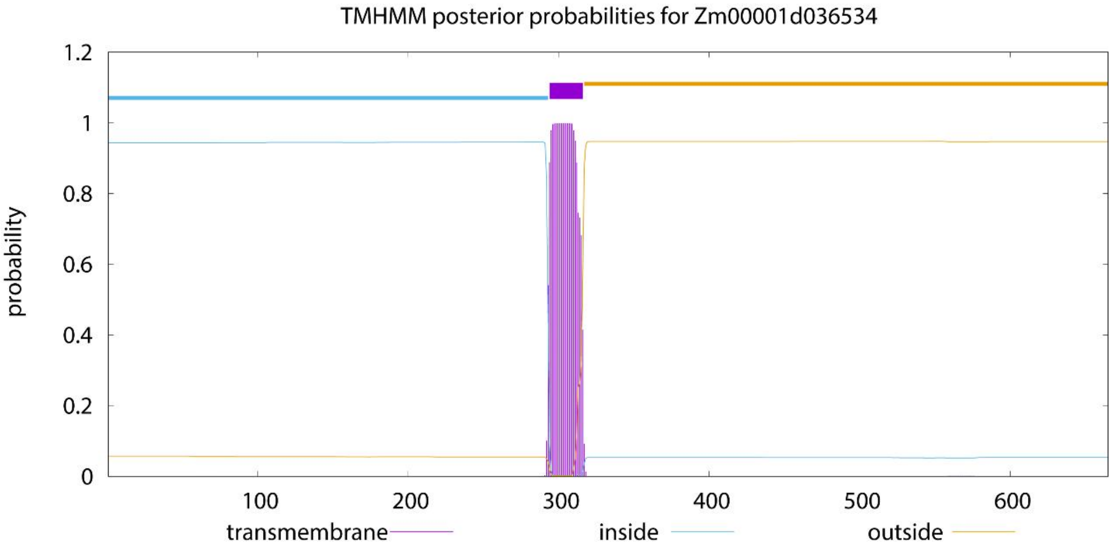
TMHMM posterior probabilities for Zm00001d036534
probability
1.2
1
0.8
0.6
0.4
0.2
0
100
200
300
400
500
600
transmembrane
inside
outside

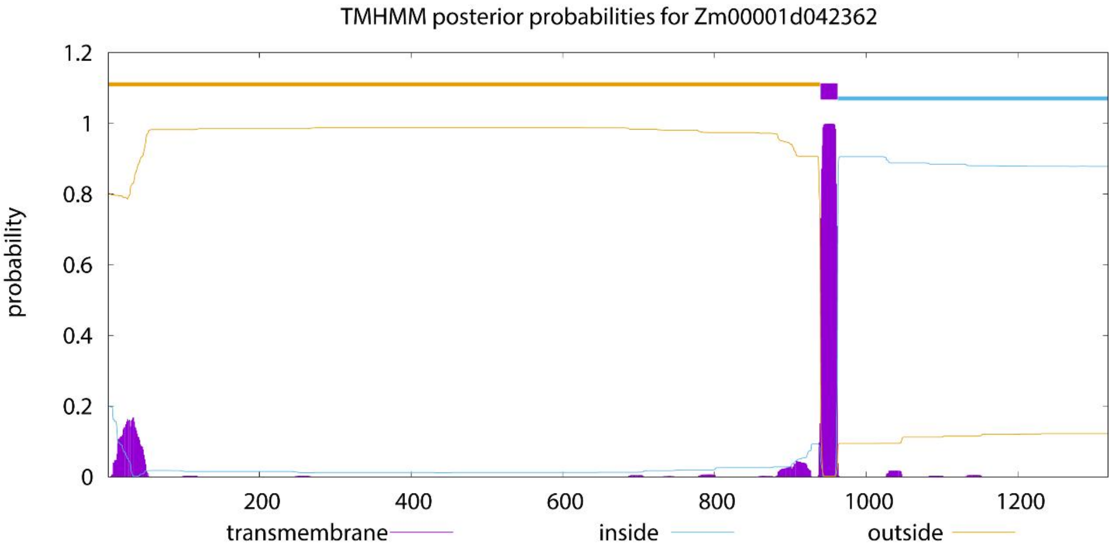
TMHMM posterior probabilities for Zm00001d042362
probability
1.2
1
0.8
0.6
0.4
0.2
0
200
400
600
800
1000
1200
transmembrane
inside
outside

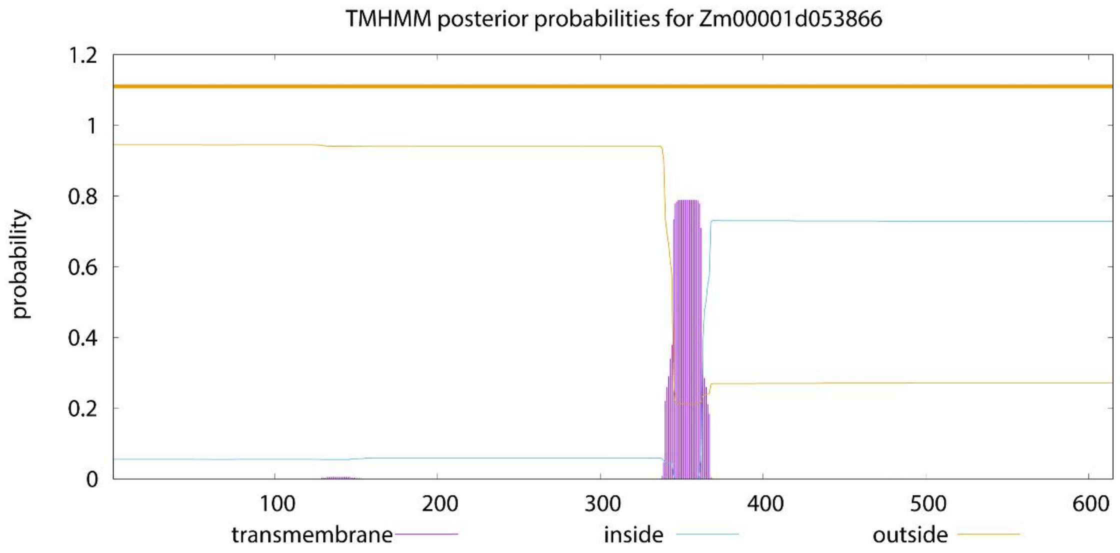

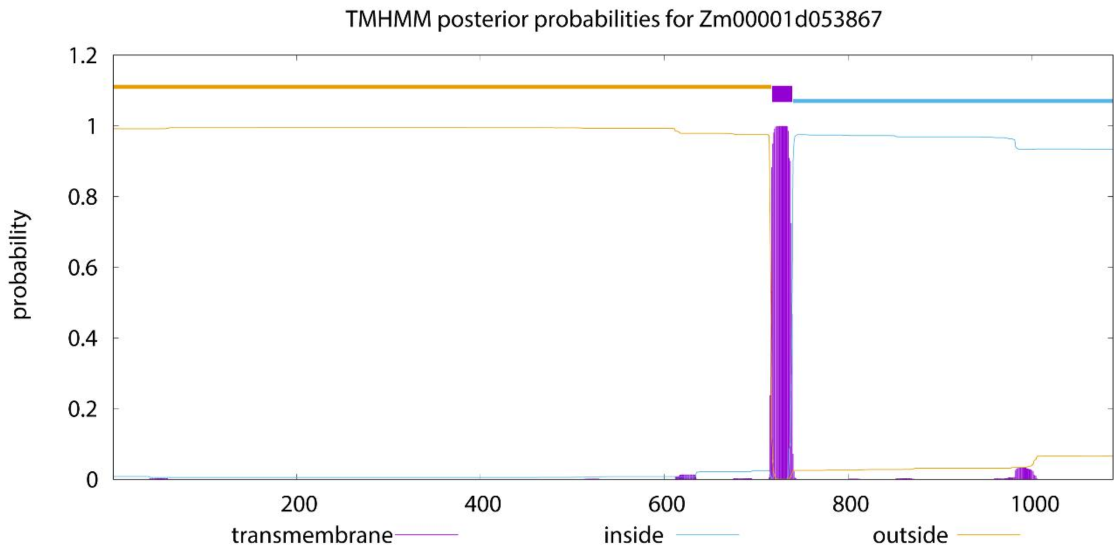

Transmembrane structure domain of subfamily XV.
